# Denitrogenative Suzuki and carbonylative Suzuki coupling reactions of benzotriazoles with boronic acids†
†Electronic supplementary information (ESI) available. See DOI: 10.1039/c7sc00367f



**DOI:** 10.1039/c7sc00367f

**Published:** 2017-03-15

**Authors:** Yuanhao Wang, Yunfei Wu, Yuanhe Li, Yefeng Tang

**Affiliations:** a School of Pharmaceutical Sciences , Tsinghua University , Beijing 100084 , China . Email: yefengtang@tsinghua.edu.cn; b Collaborative Innovation Center for Biotherapy , State Key Laboratory of Biotherapy and Cancer Center , West China Medical School , Sichuan University , Chengdu 610041 , China

## Abstract

Unprecedented palladium-catalyzed denitrogenative Suzuki and carbonylative Suzuki coupling reactions of benzotriazoles with boronic acids have been realized, which afforded structurally diverse *ortho*-amino-substituted biaryl and biaryl ketone derivatives. The key to this success is due to the development of a rationally designed strategy to achieve the ring opening of benzotriazoles with a synergistic activating–stabilizing effect, which enables the *in situ* generation of the corresponding *ortho*-amino-arenediazonium species. The present work opens up a new avenue to utilize benzotriazoles as synthetic equivalents of *ortho*-amino-arenediazoniums, which otherwise could not be directly accessed by existing synthetic methods.

## Abstract 2

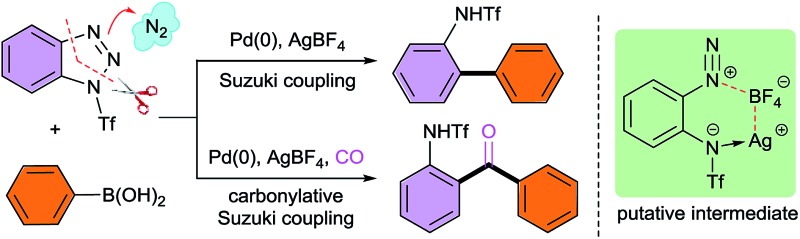
Palladium-catalyzed denitrogenative Suzuki and carbonylative Suzuki reactions of benzotriazoles with boronic acids have been achieved through the *in situ* generation of *ortho*-amino-arenediazonium species.

## Introduction

**Scheme 1:**
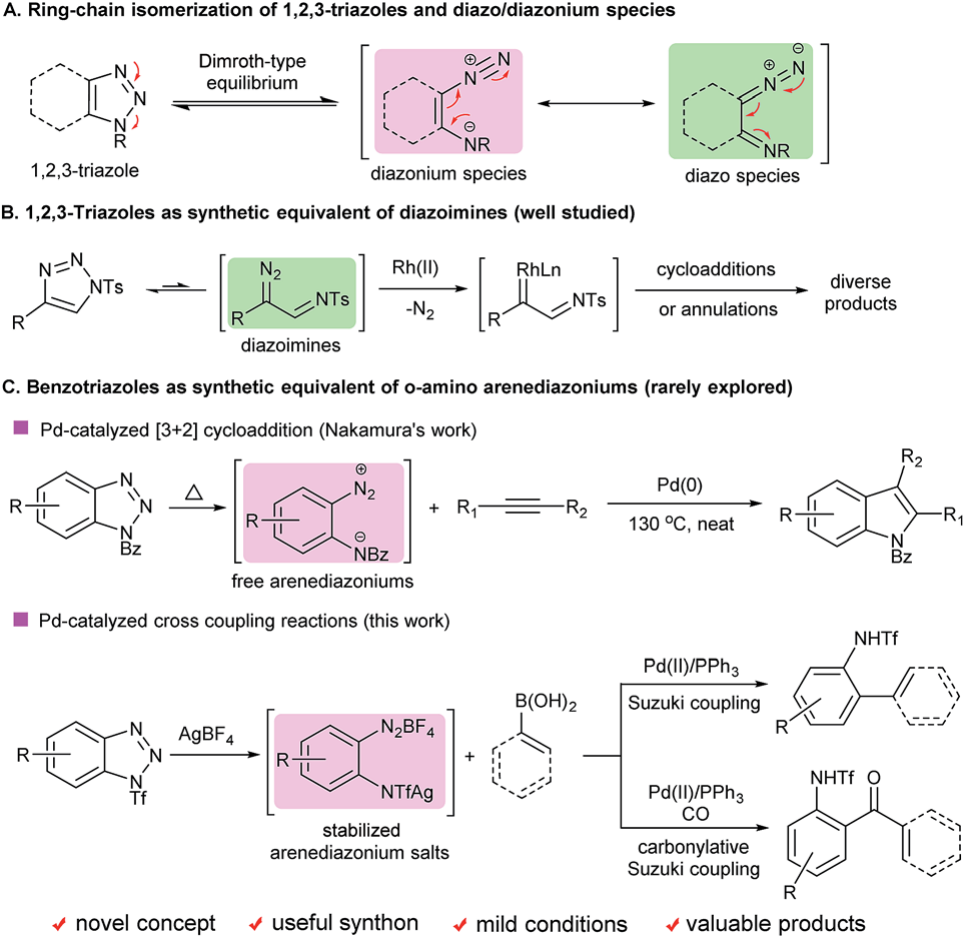
Ring-opening chemistry of 1,2,3-triazoles and benzotriazoles.

1,2,3-Triazoles are among the most important structural elements in modern chemical, biological and material sciences.^1^ One of their unique chemical properties is that they can undergo ring-chain isomerization to form the corresponding diazonium or diazo species *via* a Dimroth-type equilibrium (Scheme 1A).^2^ Considerable efforts have been devoted to the development of novel transformations based on this unique reactivity. As a paradigm, recently significant advances have been made in the applications of 1-sulfonyl-1,2,3-triazoles as synthetic equivalents of diazoimines in a broad range of intriguing reactions (Scheme 1B).^3^ In contrast, the ring-opening chemistry of benzotriazoles, a subset of 1,2,3-triazoles well known for their versatile reactivity,^4^ has remained underdeveloped, mainly due to their high stability and innate reluctance to undergo the ring-opening process.^5^ Historically, it was reported that benzotriazoles could undergo ring opening followed by denitrogenative cyclization upon photolysis^6^ or pyrolysis.^7^ However, those transformations require forcing conditions, and suffer from narrow substrate scope and moderate efficiency, and thus have rarely found applications in organic synthesis.

In 2009, Nakamura and co-workers reported a novel palladium-catalyzed denitrogenative formal [3 + 2] cycloaddition of *N*-aroylbenzotriazoles with internal alkynes (Scheme 1C).^8^ It was assumed that the reaction proceeded *via* an *ortho*-amino-arenediazonium intermediate that was generated *in situ* through the ring opening of *N*-aroylbenzotriazole. This seminal discovery shed light on the feasibility of implementing the ring opening of benzotriazoles and transition-metal-catalyzed denitrogenative transformations in one pot. However, such potential has been overlooked by the synthetic community over the past several years, probably because both the demanding reaction conditions and moderate efficiency of the reaction make it less attractive from a practical point of view. Thus, the development of a more general, efficient and robust method to achieve the ring-opening chemistry of benzotriazoles remains an unmet challenge.

Our laboratory has been working on the development of novel reactions based on the ring-opening chemistry of 1,2,3-triazoles.^9^ Given that this type of chemistry remained underdeveloped for benzotriazoles, we initiated a program to confront this challenge. Notably, when our manuscript was in preparation, Glorius and co-workers published an elegant study on the subject, in which the first visible-light-promoted denitrogenative functionalization of benzotriazoles *via* aryl radical intermediates was realized.^10^ We herein report a different strategy to achieve the ring opening of benzotriazoles with a synergistic activating–stabilizing effect, which enables the *in situ* generation of an *ortho*-amino-arenediazonium species. As proof-of-concept cases, both palladium-catalyzed denitrogenative Suzuki and carbonylative Suzuki coupling reactions of benzotriazoles with boronic acids have been developed, giving rise to diverse *ortho*-amino-substituted biaryl and biaryl ketone derivatives. The present work opens up a new avenue to utilize benzotriazoles as synthetic equivalents of *ortho*-amino-arenediazoniums, which otherwise could not be accessed by existing synthetic methods.^11^

## Results and discussion

**Scheme 2:**
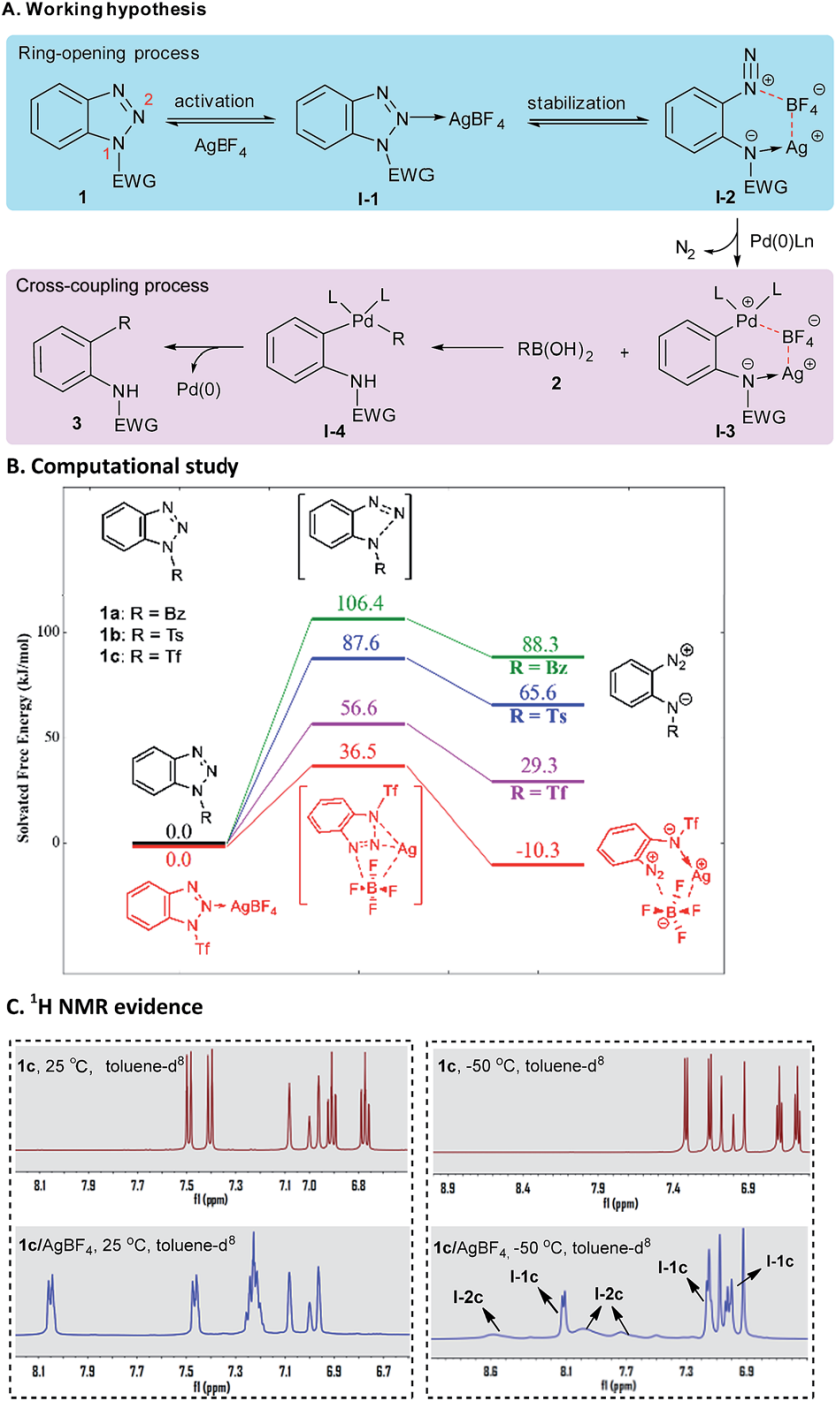
(A) Working hypothesis; (B) computational study; (C) NMR evidence.

At the outset of our study, we were aware of two challenges associated with the project: (1) how to effect the ring opening of benzotriazoles under mild conditions and (2) how to combine the ring-opening process with other synthetically useful transformations. In terms of the first question, it is known that an electron-withdrawing N1-substituent could facilitate the ring opening of benzotriazoles.^12^ For example, Ziegler and co-workers reported that 1-nonafluorobutanesulfonyl-benzotriazole^12*c*^ and 1-nitrobenzotriazole^12*f*^ could undergo ring opening upon treatment with strong nucleophiles such as amines and deprotonated phenols. Nakamura and co-workers^8^ and Glorius and co-workers^10^ utilized 1-aroylbenzotriazoles as effective substrates in their studies. To obtain deeper insight into the role of the N1-substituent, we conducted a computational study with three representative benzotriazoles (**1a–c**) as model substrates (Scheme 2B). The results revealed that the electron-withdrawing N1-substituent exerts a beneficial effect on the ring opening of benzotriazoles by lowering both the activation free energy (Δ*G* ^≠^) and Gibbs free energy difference (Δ*G*). Not surprisingly, such an activating effect positively correlates with the electron-withdrawing capability of N1-substituents (Tf > Ts > Bz). Nevertheless, in all cases, the ring opening of benzotriazoles is a thermodynamically unfavorable process, and thus the resulting zwitterionic diazoniums readily return to their ring-closed forms. To overcome this problem, we planned to use an additive such as AgBF_4_ to further promote the ring-opening process of benzotriazole by (1) activating the N1–N2 bond through the formation of a complex of benzotriazole–AgBF_4_ (**I-1**), and (2) stabilizing the ring-opening product through the formation of an arenediazonium tetrafluoroborate species (**I-2**). Encouragingly, the synergistic activating–stabilizing effect of AgBF_4_ was supported by the calculation results when using **1c** as the model substrate. Indeed, both Δ*G* ^≠^ and Δ*G* of the ring-opening process notably decreased in the presence of AgBF_4_. As a result, the ring-opening/ring-closing equilibrium shifted in the desired direction. More convincing evidence was obtained from the extensive spectroscopic study performed by us. As shown in Scheme 2C, the chemical shifts of **1c** (toluene-d^8^, 25 °C) notably move downfield in the presence of equimolar AgBF_4_, thus implying that there exists a strong coordination effect between AgBF_4_ and **1c**. Moreover, the variable temperature ^1^H NMR experiments showed that a new group of broad signals appeared when the ^1^H NMR spectrum was recorded at –50 °C, which might be due to the putative ring-opening species **I-2c**. Of note is the fact that a similar phenomenon was also observed in the variable temperature ^19^F NMR experiments (for details, see ESI†).

As to the second question, we envisaged that since arenediazonium tetrafluoroborates have been proven to be versatile precursors in organic synthesis,^13^ it was feasible to combine the ring-opening chemistry of benzotriazoles with various other synthetically useful transformations, such as the palladium-catalyzed denitrogenative Suzuki coupling reaction. Indeed, Bohle *et al.* proved that the ring-opening forms of benzotriazoles could be trapped by coordination to a suitable organometallic complex.^14^ In the current scenario, the *in situ* generated *ortho*-amino-arenediazonium tetrafluoroborate (**I-2**) would undergo oxidative addition with Pd(0) to give an organopalladium complex (**I-3**) which, after transmetalation with boronic acid (**2**) and reductive elimination, could advance to the final product (**3**) (Scheme 2A).

We commenced the study by treating 1-benzoylbenzotriazole (**1a**) and phenylboronic acid (**2a**) with AgBF_4_ (2.5 equiv.) and Pd(OAc)_2_ (0.05 equiv.) in toluene at 100 °C for 12 h. To our disappointment, no reaction occurred under the reaction conditions (entry 1, Table 1). We further evaluated benzotriazoles **1b** and **1c** in the reaction. Gratifyingly, while **1b** also failed to give a promising result (entry 2), **1c** displayed superior reactivity by affording the desired product **3a** in 58% isolated yield (entry 3). Encouraged by this outcome, we proceeded to optimize the reaction using **1c** as the substrate. First of all, several other additives, including LiBF_4_, AgSbF_6_ and AgOTf, were utilized in the reactions, however, none of them gave improved results over AgBF_4_ (entries 4–6), which indicated that both of the counter ions of AgBF_4_ played crucial roles in promoting the reaction. Next, a simple evaluation of the solvent effect was conducted, however it failed to give promising outcomes (entries 7–9). Gratifyingly, we found that the usage of PPh_3_ as an additive could notably improve the reaction by affording **3a** in an excellent yield (entry 10). Comparably, the other commonly used phosphine ligand, dppf, afforded only a moderate yield (entry 11). Interestingly, although Pd(PPh_3_)_4_ proved to be an effective catalyst for the transformation, it gave a decreased yield (entry 12). Also of note is the fact that lowering the reaction temperature to 80 °C had no side-effects on the reaction (entry 12). However, a poor yield of **3a** was obtained when the reaction was performed with reduced equivalents of or in the absence of AgBF_4_ (entries 14–15).

**Table 1:** Condition optimization^*a*^ ^,^ ^*b*^

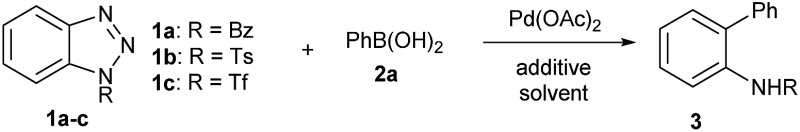					
Entry	Triazole	Solvent	Additive	*T* (°C)	Yield of **3**
1	**1a**	Toluene	AgBF_4_	100	n.r.
2	**1b**	Toluene	AgBF_4_	100	n.r.
3	**1c**	Toluene	AgBF_4_	100	58%
4	**1c**	Toluene	LiBF_4_	100	n.r.
5	**1c**	Toluene	AgSbF_6_	100	18%
6	**1c**	Toluene	AgOTf	100	27%
7	**1c**	CH_3_CN	AgBF_4_	100	17%
8	**1c**	1,4-Dioxane	AgBF_4_	100	25%
9	**1c**	DMF	AgBF_4_	100	n.r.
10	**1c**	Toluene	AgBF_4_/PPh_3_	100	92%
11	**1c**	Toluene	AgBF_4_/dppf	100	39%
12^*c*^	**1c**	Toluene	AgBF_4_/PPh_3_	100	68%
13	**1c**	Toluene	AgBF_4_/PPh_3_	80	94%
14^*d*^	**1c**	Toluene	AgBF_4_/PPh_3_	80	22%
15	**1c**	Toluene	PPh_3_	80	13%

^*a*^Reaction conditions: **1a–c** (0.30 mmol), **2a** (0.45 mmol), Pd(OAc)_2_ (0.015 mmol), PPh_3_ (0.09 mmol) and AgBF_4_ (0.75 mmol) in the solvent (3.0 mL).

^*b*^Isolated yield.

^*c*^Pd(PPh_3_)_4_ (5 mol%) was used.

^*d*^1.0 equiv. of AgBF_4_ (0.30 mmol) was used. n.r. = no reaction. Bz = benzoyl, Ts = *p*-toluenesulfonyl, Tf = trifluoromethanesulfonyl, dppf = 1,1′-bis(diphenylphosphino)ferrocene.

Having determined the optimal conditions, we next investigated the substrate scope of the reaction. First, a variety of substituted benzotriazoles were evaluated using **2a** as a reactant. Pleasingly, all of the examined benzotriazoles bearing either electron-donating or -withdrawing substituents were proven to be suitable substrates by affording the corresponding products (**3b–3i**, Table 2) in good to excellent yields. Generally, the 5- and 6-substituted benzotriazoles gave slightly higher yields than the 4-substituted one (*e.g.* **3b** and **3c** *vs.* **3i**). Moreover, the naphtha[2,3-*d*]-1,2,3-triazole was also amenable to the reaction, thus indicating that the reaction could be extended to the use of other aromatic ring-fused 1,2,3-triazole derivatives. Next, the scope of boronic acids was evaluated using **1c** as a reactant. As shown, an array of substituted phenylboronic acids worked well to efficiently give the corresponding products, regardless of the steric or electronic bias imposed by the substrates. Also of note is the fact that the ester, amide and cyano functional groups remained unchanged during the reactions, thus demonstrating good functionality tolerance. Some other aryl boronic acids were also amenable to the reactions, as witnessed by the reactions leading to **3w** and **3x**. Not surprisingly, the transformation could be applied to the synthesis of biaryl derivatives containing two substituted aromatic rings (*e.g.* **3y–ab**). Last but not least, vinylboronic acids were also proven to be effective substrates for the reactions leading to **3ac–af**, thus demonstrating their application in the synthesis of *ortho*-amino-substituted styrene derivatives.

**Table 2:** Substrate scope of the Suzuki reaction^*a*^ ^,^ ^*b*^

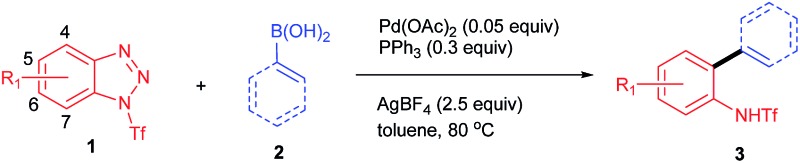
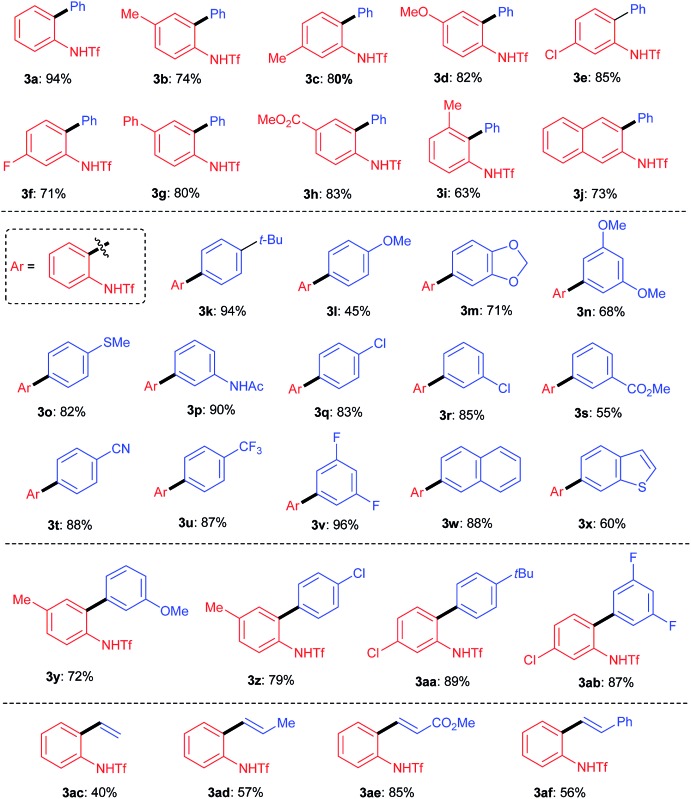

^*a*^Conditions: **1** (0.30 mmol), **2** (0.45 mmol), Pd(OAc)_2_ (0.015 mmol), PPh_3_ (0.09 mmol) and AgBF_4_ (0.75 mmol) in toluene (3.0 mL).

^*b*^Isolated yield.

To further demonstrate the synthetic utility of the ring-opening chemistry, we successfully developed an intriguing carbonylative Suzuki coupling reaction, which provided a new method to access *ortho*-amino-substituted biaryl ketone derivatives. As shown in Table 3, a slightly different catalytic system (AgBF_4_, Pd(PPh_3_)_2_Cl_2_, toluene, CO, 80 °C) was utilized in the carbonylative Suzuki coupling reactions. Generally, the transformations exhibited moderate substrate scopes with regard to both the benzotriazole and aryl boronic acid reactants. Most of the reactions proceeded smoothly to furnish the corresponding products in good to excellent yields, regardless of the electronic properties and substituent patterns of the substrates. However, different from the aforementioned Suzuki coupling reactions, the vinyl boronic acids failed to give the desired products (*e.g.* **4s**) under the studied conditions.

**Table 3:** Scope of the carbonylative Suzuki coupling reaction^*a*^ ^,^ ^*b*^

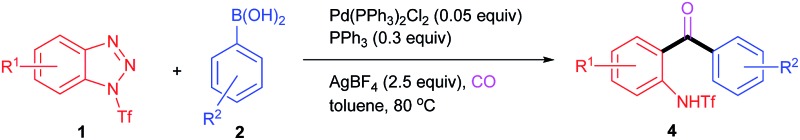
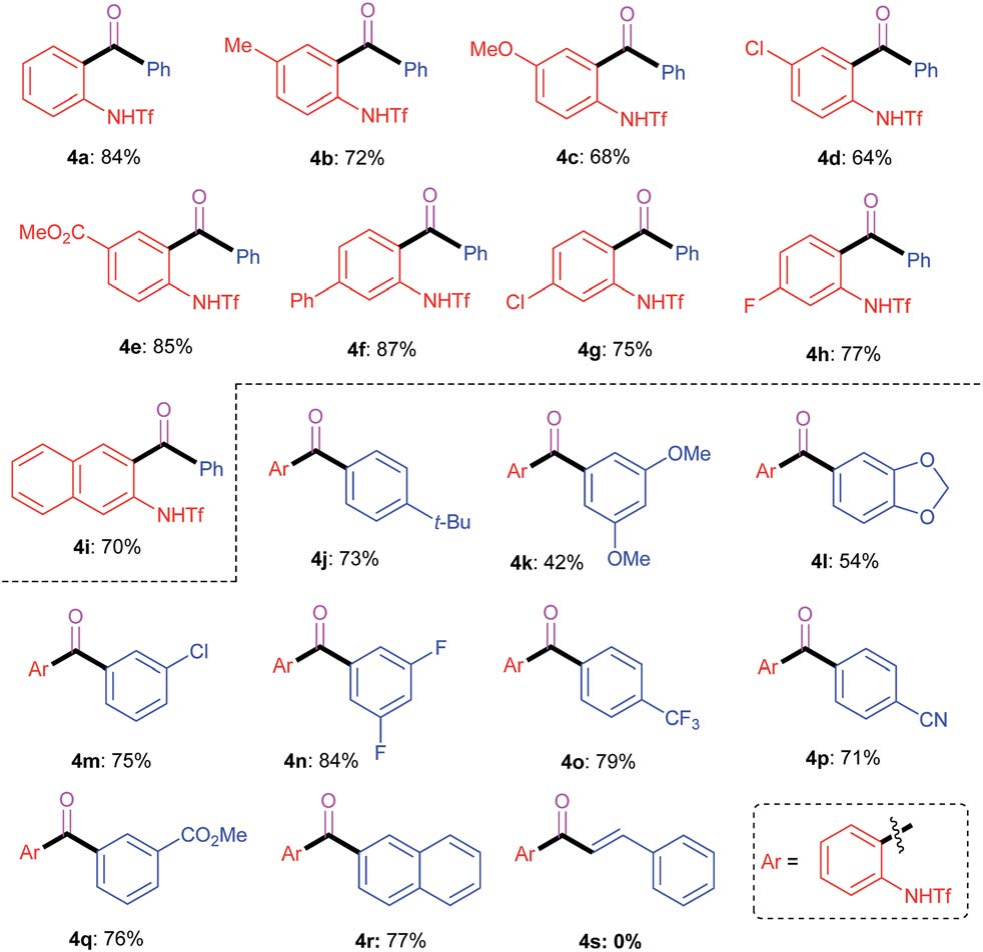

^*a*^Conditions: **1** (0.30 mmol), **2** (0.45 mmol), Pd(PPh_3_)_2_Cl_2_ (0.015 mmol), PPh_3_ (0.09 mmol) and AgBF_4_ (0.75 mmol) in toluene (3.0 mL) with a CO balloon (1 atm).

^*b*^Isolated yield.

**Scheme 3:**
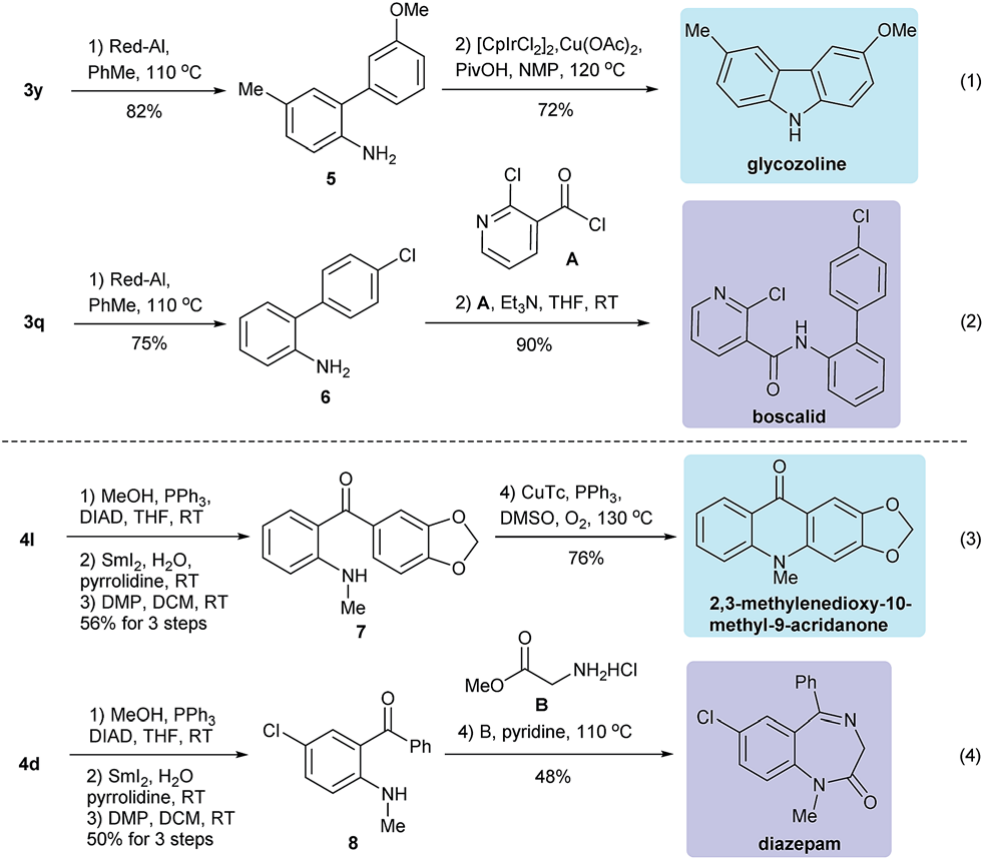
Applications in natural product and drug synthesis.

To exemplify the power of the above reactions, we converted some of the resulting products into bioactive natural products and drugs. For example, the Suzuki coupling product **3y** could undergo deprotection (Red-Al, toluene, reflux, 82%) followed by an intramolecular Ir-catalyzed annulation^15^ to give the carbazole alkaloid glycozoline,^16^ which exhibits antibiotic and antifungal properties (eqn (1), Scheme 3). Similarly, **3q** could undergo sequential deprotection and condensation with 2-chloronicotinoyl chloride to provide boscalid,^17^ a fungicide marketed by BASF company (eqn (2)). For the carbonylative Suzuki coupling product **4l**, it could be converted to **7** in three steps (methylation, SmI_2_ mediated reductive deprotection and Dess–Martin oxidation), which then underwent Cu-catalyzed intramolecular C–H bond activation/C–N bond formation^18^ to give the alkaloid 2,3-methylenedioxy-10-methyl-9-acridanone (eqn (3)).^19^ The same protocol was also utilized to convert **4d** to **8**, which, after condensation with glycine methyl ester, gave diazepam, a well-known drug for treating anxiety and epilepsy (eqn (4)).^20^

## Conclusions

In summary, we developed a new strategy to achieve the ring opening of benzotriazole with a synergistic activating–stabilizing effect, which enables the facile generation of a versatile *ortho*-amino-arenediazonium species. As proof-of-concept examples, palladium-catalyzed denitrogenative Suzuki and carbonylative Suzuki coupling reactions of benzotriazoles with boronic acids have been achieved. The great synthetic potential of the developed chemistry was demonstrated by its application in the synthesis of bioactive natural products and drugs. We anticipated that the novel concept presented in this work may inspire the development of more mechanistically interesting and synthetically useful transformations. Related studies on this subject are currently underway in our laboratory and the progress will be communicated in due course.
