# Supplementary material for: Denitrogenative Suzuki and carbonylative Suzuki coupling reactions of benzotriazoles with boronic acids
Source: Chem Sci. 2017 Mar 15;8(5):3852–7. doi: 10.1039/c7sc00367f (PMC5578364; doi:10.1039/c7sc00367f)
Supplement: Supplementary file 1 [file SC-008-C7SC00367F-s001.pdf]

Electronic Supplementary Material (ESI) for Chemical Science.
This journal is © The Royal Society of Chemistry 2017

Supporting Information

Denitrogenative Suzuki and Carbonylative Suzuki Coupling Reactions of Benzotriazoles with Boronic Acids

Yuanhao Wang[a] , Yunfei Wu,[b] Yuanhe Li,[a] and Yefeng Tang*[a,b]

[a]*School of Pharmaceutical Sciences, Tsinghua University, Beijing 100084, China.*
[b]*Collaborative Innovation Center for Biotherapy, West China Hospital, Sichuan University, Chengdu 610041, P.R. China.*

Table of Contents

1. General Information 2

2. Variable Temperature NMR Studies 3

3. Preparation of 1-Trifluoromethylsulfonyl benzotriazoles 6

4. General Procedures for Suzuki and Carbonylative Suzuki Coupling Reactions 8

5. Analysis Data of 1-trifluoromethylsulfonyl Benzotriazoles 8

6. Analysis Data of Suzuki and Carbonylative Suzuki Coupling Products 10

7. NMR Spectra of 1-trifluoromethylsulfonyl Benzotriazoles 26

8. NMR Spectra of Suzuki and Carbonylative Suzuki Coupling Products 38

9. Computational Studies 96

1. General Information

NMR spectra were recorded on Bruker AV400 and AV500 instrument. TMS was used as internal standard for ^{1}H NMR ($CDCl_3$, 0 ppm), and solvent signal was used as reference for ^{1}H NMR (toluene-d^8, 2.08, 6.96, 7.00, 7.08 ppm) and ^{13}C NMR ($CDCl_3$, 77.16 ppm). The following abbreviations were used to explain the multiplicities: s = singlet, d = doublet, t = triplet, q = quartet, td = triple doublet, qd = quarter doublet, m = multiplet. High-resolution mass spectra (HRMS) were recorded on a Waters Xevo G2 QTOF MS.

Reactions were monitored by Thin Layer Chromatography on plates (GF_{254}) supplied by Yantai Chemicals (China) using UV light as visualizing agent and an ethanolic solution of Potassium permanganate, and heat as developing agents. If not specially mentioned, flash column chromatography uses silica gel (200-300 mesh) supplied by Tsingtao Haiyang Chemicals (China).

Solvent purification was conducted according to Purification of Laboratory Chemicals (Peerrin, D. D.; Armarego, W. L. and Perrins, D. R., Pergamon Press: Oxford, 1980). Yields refer to chromatographically and spectroscopically (^{1}H NMR) homogeneous materials.

2. Variable Temperature NMR Studies

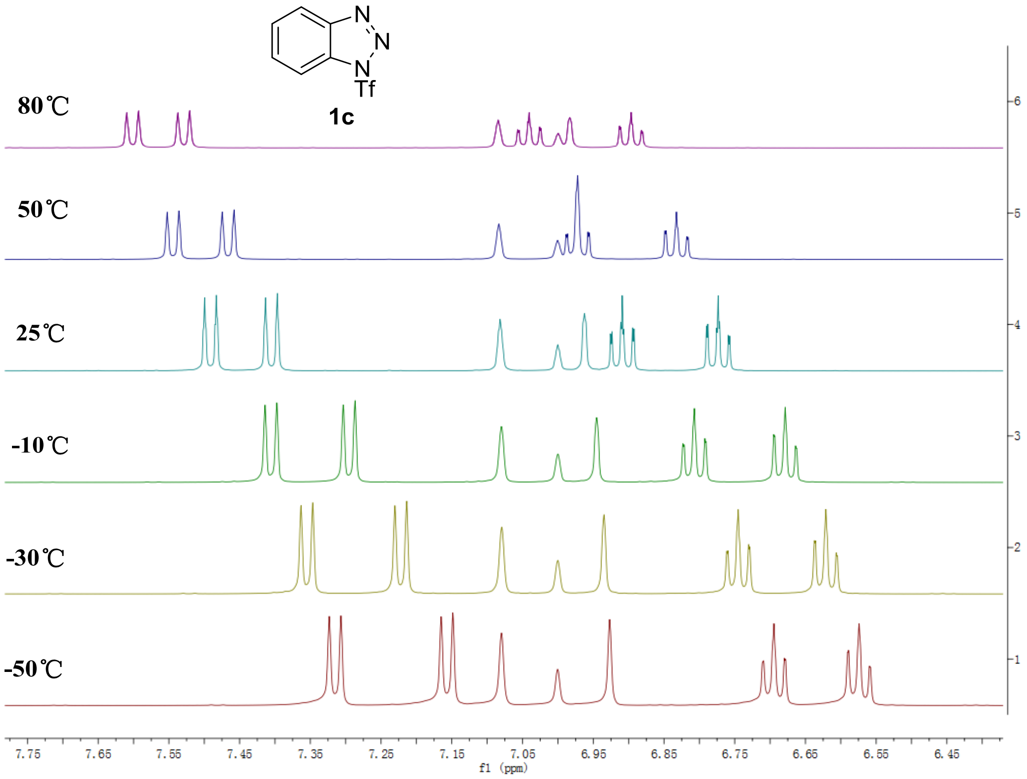

Figure S-1. Variable temperature ^{1}H NMR spectrum of **1c** (toluene-d^{8}, 500 MHz)

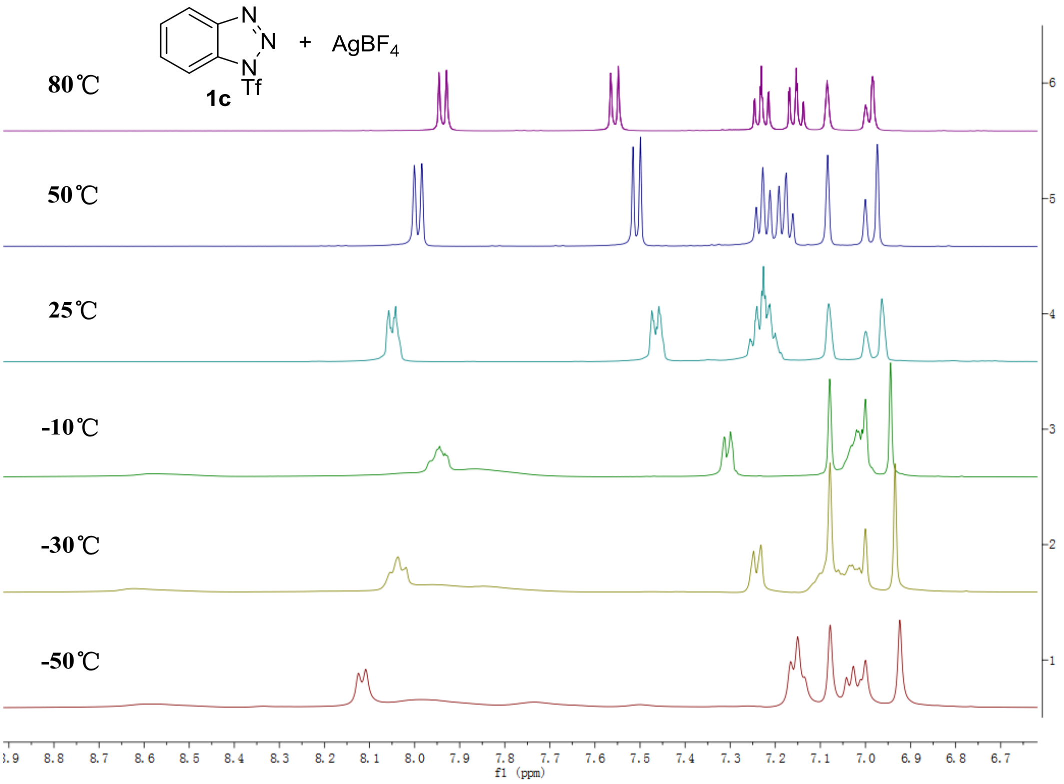

Figure S-2. Variable temperature ^{1}H NMR spectrum of **1c**/$AgBF_4$ (toluene-d^{8}, 500 MHz)

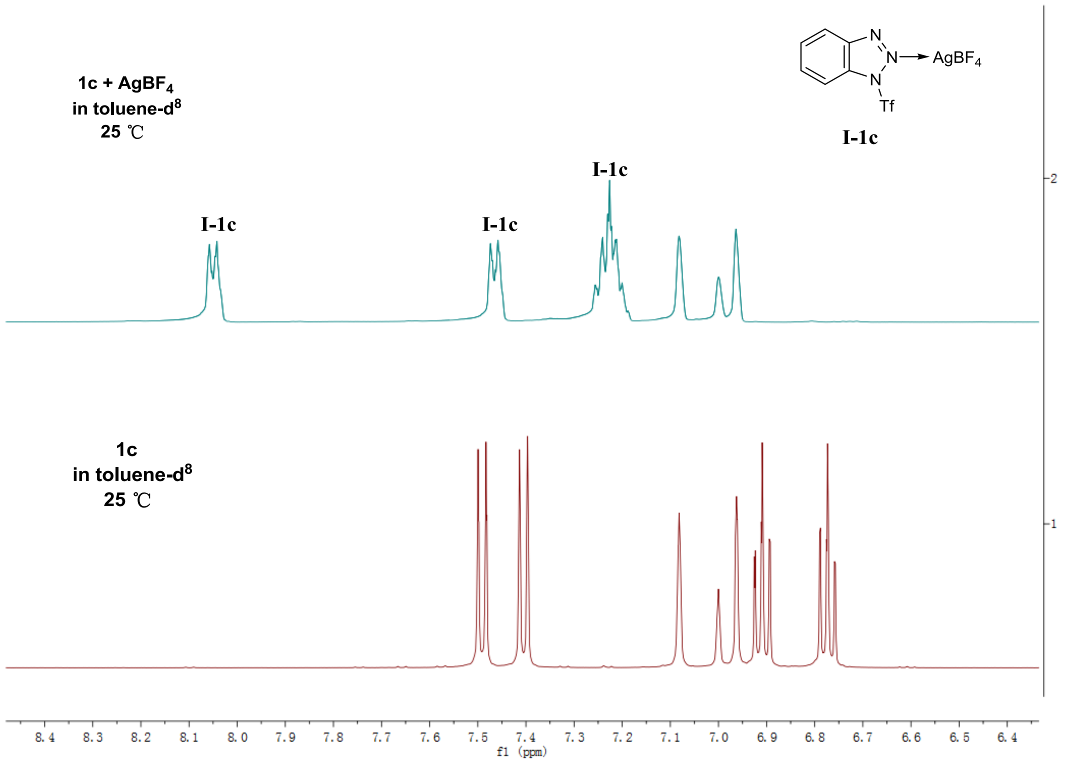

Figure S-3. Comparison of ^{1}H NMR Spectrum of **1c** and **1c**/$AgBF_4$ at 25℃

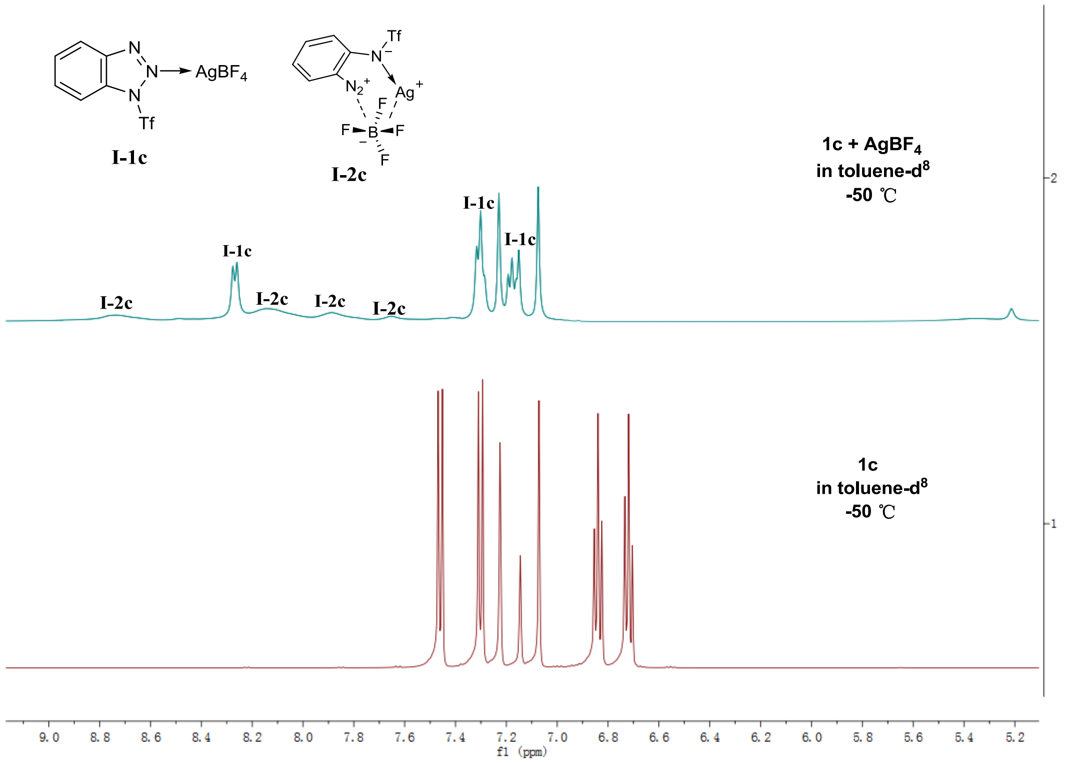

Figure S-4. Comparison of ^{1}H NMR Spectrum of **1c** and **1c**/$AgBF_4$ at -50℃

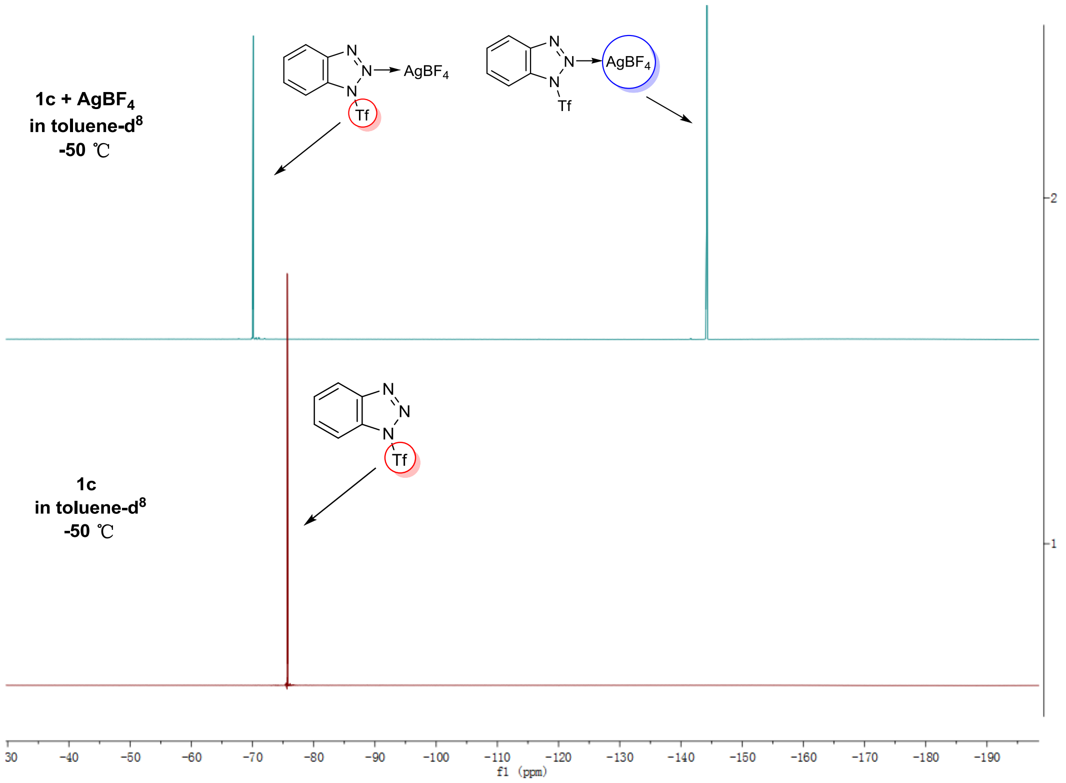

Figure S-6. Comparison of ^{19}F NMR Spectrum of **1c** and **1c**/$AgBF_4$ at 25℃

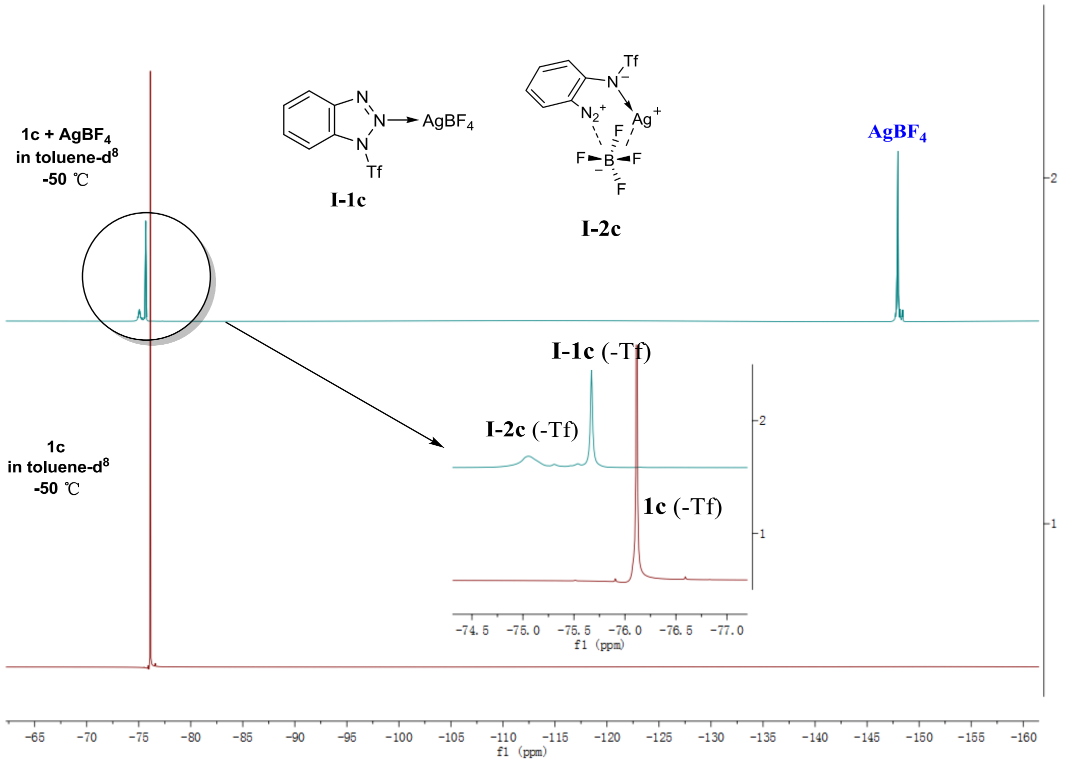

Figure S-6. Comparison of ^{19}F NMR Spectrum of **1c** and **1c**/$AgBF_4$ at -50℃

3. Preparation of 1-Trifluoromethylsulfonyl-benzotriazoles

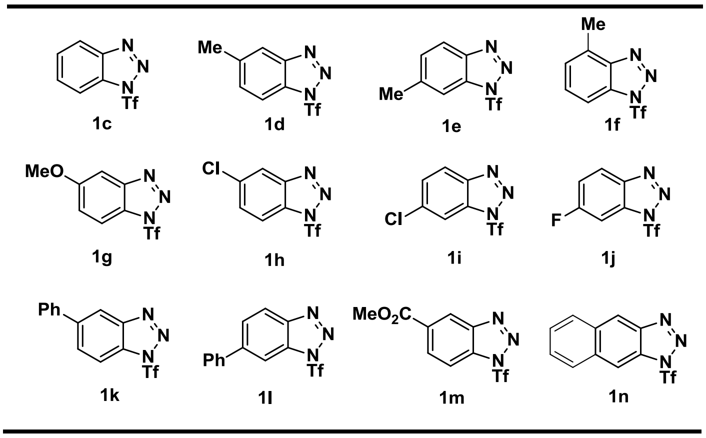

1) Procedure A (for 1c-1i, 1m and 1n)[1]

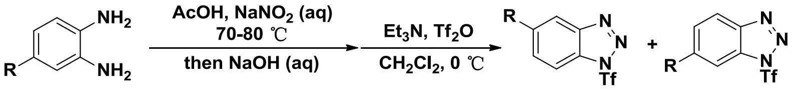

To a solution of benzene-1,2-diamine (5.0 mmol) in AcOH (10 mL) was added $NaNO_2$ aq solution (25 mL, 1M) and the mixture was stirred at 70-80 ℃ for 1h. The pH value of the solution was then adjusted to 4.4-4.6 by 40% NaOH aq solution and 1M HCl. The precipitated product was collected by filtration, washed with 5% ice-cold NaCl aq solution and recrystallized (CH_2Cl_2/MeOH) to afford the corresponding benzotriazoles as white powder.

To a solution of aforementioned benzotriazole (5.0 mmol) in CH_2Cl_2 (50 mL) was sequentially added Et_3N (6.0 mmol, 1.2 eq) and Tf_2O (6.5 mmol, 1.3 eq) at 0℃. The reaction was stirred at the same temperature for 0.5-1 h and then water (20 mL) was added. The organic layer was concentrated in vacuo and the residue was purified by flash chromatography to give the corresponding 1-trifluoromethanesulfonyl-benzotriazoles as a mixture of regioisomers.

2) Procedure B (for 1j, 1k, 1l)[2]

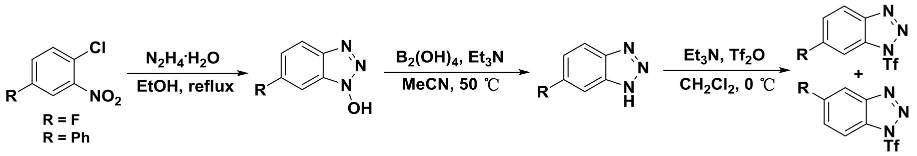

A mixture of the appropriate *ortho*-chloronitrobenzene (7.85 mmol) and hydrazine hydrate (0.77 mL, 15.70 mmol) in absolute EtOH (10 mL) was refluxed for 36 h. After removal of the solvent

---

[1] J. Fu, Y. Yang, X. Zhang, W. Mao, Z. Zhang, H. Zhu. *Bioorg. Med. Chem.* **2010**, *18*, 8457-8462.

[2] V. Gurram, H. K. Akula, R. Garlapati, N. Pottabathini, M. K. Lakshman. *Adv. Synth. Catal.* **2015**, *357*, 451-462.

under reduced pressure, the residue was dissolved in 10% aqueous Na_2CO_3 (20 mL). The solution was extracted with Et_2O and then acidified with concentrated HCl. The precipitated product was filtered, washed with water, and dried to obtain the corresponding 1-hydroxy-1*H*-benzotriazole as an off-white solid.

The prepared 1-hydroxy-1*H*-benzotriazole was dissolved in MeCN. The Et_3N (1.2 eq) was added and the reaction mixture was stirred at room temperature for 30 min. Then $B_2(OH)_4$ (1.2 eq) was added and the resulting reaction mixture was stirred for another 30 min at 50℃. After completion of the reaction, the mixture was concentrated and crude material was purified by chromatography to give the corresponding benzotriazole. Subsequently, the trifluoromethanesulfonylation of above-mentioned benzotriazole was conducted according to the same procedure as described in Procedure A.

Note: Most of the benzotriazole derivatives were obtained as a mixture of regioisomers using above methods. Their structures were assigned based on the following methods:

a) The benzotriazole itself is known compounds, such as the case of **1c**[3].

b) The benzotriazoles themselves are new compounds, but their coupling products could be converted to the known compounds after deprotection, such as the case of **1g**[4], **1k**[5], **1j** and **1m**[6].

c) The benzotriazoles themselves are new compounds, but they could be converted to known natural products and drugs, such as the case of **1d**[7] and **1h**[8].

d) The benzotriazoles themselves are new compounds, but their corresponding regioisomer could be unambiguously confirmed by above-mentioned methods. As a result, their structure could be deduced, such as the case of **1e**, **1i** and **1l.**

[3] V. I. Meshcheryakov, B. A. Shainyan, L. L. Tolstikova, A. I. Albanov, *Russ. J. Org. Chem.* **2003**, *39*, 1517.
[4] I. T. Alt, B. Plietker, *Angew. Chem. Int. Ed.* **2016**, *55*, 1519.
[5] T. Truong, O. Daugulis, *Org. Lett.* **2012**, *14*, 5964.
[6] J. Rong, L. Deng, P. Tan, C. Ni, Y. Gu, J. Hu, *Angew. Chem. Int. Ed.* **2016**, *55*, 2743.
[7] S. W. Youn, J. H. Bihn, B. S. Kim, *Org. Lett.* **2011**, *13*, 3738.
[8] Y. Wu, L. Sun, Y. Chen, Q. Zhou, J. Huang, H. Miao, H. Luo, *J. Org. Chem.* **2016**, *81*, 1244.

4. General Procedures for Suzuki and Carbonylative Suzuki Coupling Reactions

1) Procedure A (for Suzuki products 3a-3af)

A bottom of flask was sequentially charged with N-Tf-benzotriazole (0.30 mmol, 1.0 eq), phenyl/vinylboronic acid (0.45 mmol, 1.5 eq), $Pd(OAc)_2$ (3.3 mg, 0.015 mmol, 0.05 eq), PPh_3 (24 mg, 0.09 mmol, 0.3 eq) and $AgBF_4$ (145 mg, 0.75 mmol, 2.5 eq) at N_2 atmosphere. The reaction was added freshly distilled toluene (3.0 mL) and then placed in an oil bath preheated to 80 ℃. The resulting solution was heated at this temperature for 3-8 hours before being cooled to room temperature and concentrated in vacuo. The residue was directly purified by flash chromatography (SiO_2, hexanes/EtOAc) to give the corresponding Suzuki product**.**

2) Procedure B (for carbonylative Suzuki coupling products 4a-4s)

A bottom of flask was sequentially charged with *N*-Tf-benzotriazole (0.30 mmol, 1.0 eq), phenyl/vinylboronic acid (0.45 mmol, 1.5 eq), $Pd(PPh_3)_2Cl_2$ (10.4 mg, 0.015 mmol, 0.05 eq), PPh_3 (24 mg, 0.09 mmol, 0.3 eq) and $AgBF_4$ (145 mg, 0.75 mmol, 2.5 eq) at carbon monoxide (CO) atmosphere. The reaction was added freshly distilled toluene (3.0 mL) and then placed in an oil bath preheated to 80 ℃. The resulting solution was heated at this temperature for 8-12 hours before being cooled to room temperature and concentrated in vacuo. The residue was directly purified by flash chromatography (SiO_2, hexanes/EtOAc) to give the corresponding Suzuki carbonylation product**.**

5. Analysis Data of 1-trifluoromethylsulfonyl Benzotriazoles

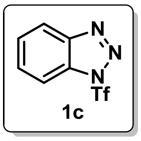

1-((trifluoromethyl)sulfonyl)-1*H*-benzo[d][1,2,3]triazole (1c): The product was obtained as a yellow solid. Yield: 82%; ^{1}H NMR (400 MHz, $CDCl_3$) δ 7.64 (dt, *J* = 7.6 Hz, *J* = 0.8 Hz, 1H), 7.80 (dt, *J* = 7.6 Hz, *J* = 0.8 Hz, 1H), 7.96 (d, *J* = 8.0 Hz, 1H), 8.24 (d, *J* = 8.0 Hz, 1H); ^{13}C (100 MHz, $CDCl_3$) δ 111.9, 119.4 (q, *J* = 321.6 Hz), 121.7, 127.4, 132.1, 132.1, 145.6.

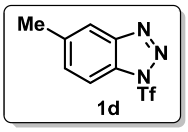

5-methyl-1-((trifluoromethyl)sulfonyl)-1*H*-benzo[d][1,2,3]triazole (1d): The product was obtained as a light yellow solid. Yield: 39% (one isomer); ^{1}H

NMR (400 MHz, $CDCl_3$) δ 2.58 (s, 3H), 7.59 (dd, *J* = 8.4 Hz, *J* = 1.2 Hz, 1H), 7.82 (d, *J* = 8.4 Hz, 1H), 7.99 (s, 1H); ^{13}C (100 MHz, $CDCl_3$) δ 21.5, 111.3, 119.4 (q, *J* = 321.6 Hz), 120.8, 130.4, 133.8, 138.0, 146.2.

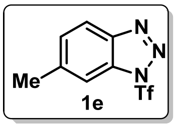

6-methyl-1-((trifluoromethyl)sulfonyl)-1H-benzo[d][1,2,3]triazole (1e): The product was obtained as an orange solid. Yield: 44% (one isomer); ^1H NMR (400 MHz, $CDCl_3$) δ 2.62 (s, 3H), 7.44 (d, *J* = 8.4 Hz, 1H), 7.72 (s, 1H), 8.07 (d, *J* = 8.4 Hz, 1H); ^{13}C (100 MHz, $CDCl_3$) δ 22.3, 111.4, 119.4 (q, *J* = 320.2 Hz), 121.0, 129.2, 132.5, 143.8, 144.1.

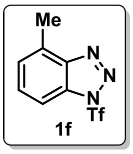

4-methyl-1-((trifluoromethyl)sulfonyl)-1*H*-benzo[d][1,2,3]triazole (1f): The product was obtained as an orange solid. Yield: 75%; ^1H NMR (400 MHz, $CDCl_3$) δ 2.87 (s, 3H), 7.40 (d, *J* = 7.2 Hz, 1H), 7.66 (t, *J* = 7.6 Hz, 1H), 7.74 (d, *J* = 8.4 Hz, 1H); ^{13}C (100 MHz, $CDCl_3$) δ 16.7, 109.0, 119.4 (q, *J* = 321.5 Hz), 127.6, 131.9, 132.1, 133.2, 145.4.

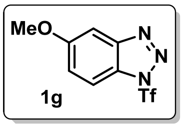

6-methoxy-1-((trifluoromethyl)sulfonyl)-1*H*-benzo[d][1,2,3]triazole (1g): The product was obtained as a white solid. Yield: 49% (one isomer); ^1H NMR (400 MHz, $CDCl_3$) δ 3.93 (s, 3H), 7.37 (dd, *J* = 9.2 Hz, *J* = 2.0 Hz, 1H), 7.52 (d, *J* = 2.0 Hz, 1H), 7.77 (d, *J* = 9.2 Hz, 1H); ^{13}C (100 MHz, $CDCl_3$) δ 56.1, 101.0, 112.2, 119.2 (q, *J* = 321.5 Hz), 123.5, 126.8, 146.9, 159.3.

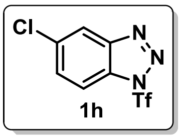

5-chloro-1-((trifluoromethyl)sulfonyl)-1H-benzo[d][1,2,3]triazole (1h): The product was obtained as a yellow solid. Yield: 30% (one isomer); ^1H NMR (400 MHz, $CDCl_3$) δ 7.75 (dd, *J* = 8.8 Hz, *J* = 1.6 Hz, 1H), 7.89 (d, *J* = 8.8 Hz, 1H), 8.22 (d, *J* = 1.6 Hz, 1H); ^{13}C (100 MHz, $CDCl_3$) δ 112.7, 119.3 (q, *J* = 321.7 Hz), 121.2, 130.8, 132.8, 133.6, 146.4.

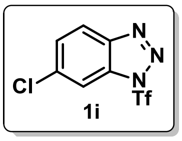

6-chloro-1-((trifluoromethyl)sulfonyl)-1*H*-benzo[d][1,2,3]triazole (1i): The product was obtained as a yellow solid. Yield: 38% (one isomer); ^1H NMR (400 MHz, $CDCl_3$) δ 7.61 (dd, *J* = 8.8 Hz, *J* = 1.6 Hz, 1H), 7.96 (d, *J* = 1.6 Hz, 1H), 8.16 (d, *J* = 8.8 Hz, 1H); ^{13}C (100 MHz, $CDCl_3$) δ 112.1, 119.3 (q, *J* = 321.7 Hz), 122.4, 128.6, 132.8, 139.2, 144.1.

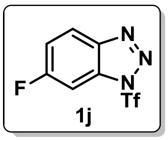

6-fluoro-1-((trifluoromethyl)sulfonyl)-1*H*-benzo[d][1,2,3]triazole (1j): The

product was obtained as a yellow solid. Yield: 47% (one isomer); ^{1}H NMR (400 MHz, $CDCl_3$) δ 7.40 (dt, *J* = 8.8 Hz, *J* = 0.8 Hz, 1H), 7.64 (dd, *J* = 7.2 Hz, *J* = 0.8 Hz, 1H), 8.22 (dd, *J* = 8.8 Hz, *J* = 4.8 Hz, 1H); ^{13}C (100 MHz, $CDCl_3$) δ 99.2 (d, *J* = 29.9 Hz), 117.0 (d, *J* = 26.2 Hz), 119.3 (q, *J* = 321.5 Hz), 123.3 (d, *J* = 10.9 Hz), 133.1 (d, *J* = 14.4 Hz), 142.1, 164.9 (d, *J* = 254.6 Hz).

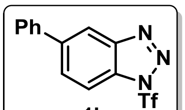

1k

6-phenyl-1-((trifluoromethyl)sulfonyl)-1*H*-benzo[d][1,2,3]triazole (1k): The product was obtained as a yellow solid. Yield: 42% (one isomer); ^{1}H NMR (400 MHz, $CDCl_3$) δ 7.48 (t, *J* = 7.2 Hz, 1H), 7.55 (t, *J* = 7.2 Hz, 2H), 7.67 (d, *J* = 7.6 Hz, 2H), 8.00-8.05 (m, 2H), 8.40 (s, 1H); ^{13}C (100 MHz, $CDCl_3$) δ 112.0, 119.4, 119.4 (q, *J* = 321.6 Hz), 127.7, 128.6, 129.4, 131.3, 131.9, 139.1, 141.5, 146.5.

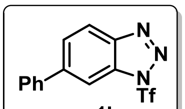

1l

5-phenyl-1-((trifluoromethyl)sulfonyl)-1*H*-benzo[d][1,2,3]triazole (1l): The product was obtained as a yellow solid. Yield: 25% (one isomer); ^{1}H NMR (400 MHz, $CDCl_3$) δ 7.48-7.58 (m, 3H), 7.69 (d, *J* = 7.6 Hz, 2H), 7.89 (d, *J* = 8.4 Hz, 1H), 8.11 (s, 1H), 8.30 (d, *J* = 8.8 Hz, 1H); ^{13}C (100 MHz, $CDCl_3$) δ 109.8, 119.4 (q, *J* = 321.7 Hz), 121.7, 127.4, 128.0, 129.1, 129.4, 132.8, 139.2, 144.8, 146.0.

Methyl 1-((trifluoromethyl)sulfonyl)-1*H*-benzo[d][1,2,3]triazole-5-carboxylate (1m): The

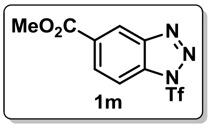

1m

product was obtained as a yellow solid. Yield: 35% (one isomer); ^{1}H NMR (400 MHz, $CDCl_3$) δ 4.04 (s, 3H), 8.30 (d, *J* = 10.0 Hz, 1H), 8.33 (d, *J* = 10.0 Hz, 1H), 8.62 (s, 1H); ^{13}C (100 MHz, $CDCl_3$) δ 53.2, 113.6, 119.3 (q, *J* = 321.7 Hz), 121.6, 128.4, 132.0, 133.7, 147.6, 165.3.

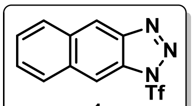

1n

1-((trifluoromethyl)sulfonyl)-1*H*-naphtho[2,3-d][1,2,3]triazole (1n): The product was obtained as a white solid. Yield: 78%; ^{1}H NMR (400 MHz, $CDCl_3$) δ 7.64 (t, *J* = 7.2 Hz, 1H), 7.71 (t, *J* = 7.2 Hz, 1H), 8.07 (d, *J* = 8.4 Hz, 1H), 8.14 (d, *J* = 8.4 Hz, 1H), 8.33 (s, 1H), 8.77 (s, 1H); ^{13}C (100 MHz, $CDCl_3$) δ 108.8, 119.5 (q, *J* = 319.7 Hz), 121.1, 127.0, 128.4, 128.6, 129.3, 129.8, 131.8, 134.9, 144.5.

6. Analysis Data of Suzuki and Carbonylative Suzuki Coupling Products

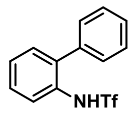

3a

***N*-([1,1'-biphenyl]-2-yl)-1,1,1-trifluoromethanesulfonamide (3a):** The product was obtained as a colorless oil. Yield: 94%; ^{1}H NMR (400 MHz, $CDCl_3$) δ 6.76 (s, 1H), 7.35-7.38 (m, 4H), 7.42-7.57 (m, 4H), 7.68 (d, *J* = 8.0 Hz, 1H); ^{13}C (100 MHz, $CDCl_3$) δ 119.6 (q, *J* = 320.6 Hz), 121.8, 126.8, 128.8, 129.1, 129.2, 129.5, 130.9, 131.7,

135.0, 136.9; IR ν_{max} (film): 3332.66, 2947.78, 2835.97, 1646.12, 1448.98, 1412.62, 1203.93, 1112.81, 1014.93, 509.91 cm^{-1}; HRMS m/z calcd for $C_{13}H_9F_3NO_2S$ [M-H]$^+$: 300.0306; found: 300.0309.

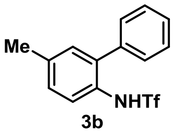

3b

1,1,1-trifluoro-N-(5-methyl-[1,1'-biphenyl]-2-yl)methanesulfonamide (3b): The product was obtained as a colorless oil. Yield: 74%; ^{1}H NMR (400 MHz, CDCl$_3$) δ 2.38 (s, 3H), 7.12 (s, 1H), 7.20 (d, *J* = 8.0 Hz, 1H), 7.30 (d, *J* = 7.2 Hz, 2H), 7.42-7.51 (m, 4H); ^{13}C (100 MHz, CDCl$_3$) δ 21.0, 119.7 (q, *J* = 320.6 Hz), 122.2, 128.7, 129.0, 129.2, 129.4, 129.6, 131.5, 135.3, 136.9, 137.2; IR ν_{max} (film): 3373.47, 2942.86, 2830.61, 1488.58, 1421.46, 1365.15, 1233.78, 1206.12, 1142.46, 1022.45 cm^{-1}; HRMS m/z calcd for $C_{14}H_{11}F_3NO_2S$ [M-H]$^+$: 314.0463; found: 314.0466.

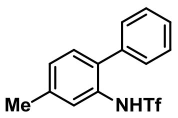

3c

1,1,1-trifluoro-*N*-(4-methyl-[1,1'-biphenyl]-2-yl)methanesulfonamide (3c): The product was obtained as a colorless oil. Yield: 80%; ^{1}H NMR (400 MHz, CDCl$_3$) δ 2.45 (s, 3H), 6.68 (s, 1H), 7.15 (d, *J* = 7.6 Hz, 1H), 7.21 (d, *J* = 7.6 Hz, 1H), 7.33 (d, *J* = 7.6 Hz, 2H), 7.45-7.54 (m, 4H); ^{13}C (100 MHz, CDCl$_3$) δ 21.5, 119.7 (q, *J* = 320.8 Hz), 122.1, 127.5, 128.6, 129.3, 129.5, 130.6, 131.5, 132.1, 136.9, 139.3; IR ν_{max} (film): 3324.49, 2987.54, 2899.75, 1648.98, 1451.02, 1228.63, 1116.33, 1065.91, 1016.33, 567.35 cm^{-1}; HRMS m/z calcd for $C_{14}H_{11}F_3NO_2S$ [M-H]$^+$: 314.0463; found: 314.0462.

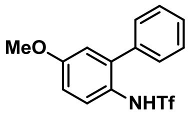

3d

1,1,1-trifluoro-*N*-(5-methoxy-[1,1'-biphenyl]-2-yl)methanesulfonamide (3d): The product was obtained as a white solid. Yield: 82%; ^{1}H NMR (400 MHz, CDCl$_3$) δ 3.86 (s, 3H), 6.55 (s, 1H), 6.87 (d, *J* = 2.8 Hz, 1H), 6.95 (dd, *J* = 8.8 Hz, *J* = 2.8 Hz, 1H), 7.34 (d, *J* = 7.6 Hz, 2H), 7.45-7.54 (m, 4H); ^{13}C (100 MHz, CDCl$_3$) δ 55.7, 114.2, 114.8, 119.6 (q, *J* = 320.7 Hz), 124.1, 125.6, 128.7, 129.1, 129.3, 137.3, 138.4, 158.5; IR ν_{max} (film): 3674.11, 2987.47, 2900.08, 1607.88, 1508.96, 1486.27, 1409.58, 1393.55, 1367.75, 1201.30, 1141.13, 1065.87, 879.04, 762.28 cm^{-1}; HRMS m/z calcd for $C_{14}H_{11}F_3NO_3S$ [M-H]$^+$: 330.0412; found: 330.0418.

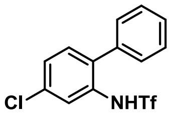

3e

***N*-(4-chloro-[1,1'-biphenyl]-2-yl)-1,1,1-trifluoromethanesulfonamide (3e):** The product was obtained as a colorless oil. Yield: 85%; ^{1}H NMR (400 MHz, CDCl$_3$) δ 6.70 (s, 1H), 7.26 (d, *J* = 8.0 Hz, 1H), 7.31 (d, *J* = 7.2 Hz, 3H), 7.50-7.57 (m, 3H), 7.70 (s, 1H); ^{13}C (100 MHz, CDCl$_3$) δ 119.6 (q, *J* = 320.7 Hz), 121.3, 126.8, 129.1, 129.2, 129.8, 131.8, 132.8, 132.8, 134.8, 135.7; IR ν_{max} (film): 3674.23, 3359.18, 2987.28,

2900.10, 1480.65, 1405.23, 1393.59, 1379.22, 1231.71, 1139.85, 1065.81, 1056.75, 1016.33, 891.61, 606.12 cm^{-1}; HRMS m/z calcd for $C_{13}H_8ClF_3NO_2S$ $[M-H]^+$: 333.9916; found: 333.9920.

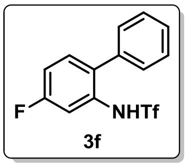

1,1,1-trifluoro-*N*-(4-fluoro-[1,1'-biphenyl]-2-yl)methanesulfonamide (3f): The product was obtained as a colorless oil. Yield: 71%; ^{1}H NMR (400 MHz, $CDCl_3$) δ 6.69 (s, 1H), 7.01 (dt, *J* = 8.4 Hz, *J* = 2.4 Hz, 1H), 7.24-7.30 (m, 3H), 7.43 (dd, *J* = 10.0 Hz, *J* = 2.4 Hz, 1H), 7.47-7.54 (m, 3H); ^{13}C (100 MHz, $CDCl_3$) δ 108.3 (d, *J* = 27.1 Hz), 113.4 (d, *J* = 21.3 Hz), 119.6 (q, *J* = 320.7 Hz), 129.1, 129.3, 129.8, 129.9 (d, *J* = 3.5 Hz), 132.1 (d, *J* = 9.0 Hz), 133.0 (d, *J* = 10.9 Hz), 135.8, 162.5 (d, *J* = 246.4 Hz); IR ν_{max} (film): 3674.32, 3334.69, 2987.32, 2900.09, 1655.10, 1405.60, 1393.45, 1380.15, 1230.96, 1065.81, 1056.77, 1014.29, 891.78, 612.24 cm^{-1}; HRMS m/z calcd for $C_{13}H_8F_4NO_2S$ $[M-H]^+$: 318.0212; found: 318.0209.

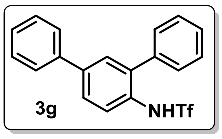

***N*-([1,1':3',1''-terphenyl]-4'-yl)-1,1,1-trifluoromethanesulfonamide (3g):** The product was obtained as a colorless oil. Yield: 80%; ^{1}H NMR (400 MHz, $CDCl_3$) δ 6.68 (s, 1H), 7.35-7.39 (m, 3H), 7.45 (t, *J* = 7.2 Hz, 2H), 7.48-7.55 (m, 4H), 7.59 (d, *J* = 8.0 Hz, 2H), 7.62 (dd, *J* = 8.4 Hz, *J* = 0.8 Hz, 1H), 7.71 (d, *J* = 8.4 Hz, 1H); ^{13}C (100 MHz, $CDCl_3$) δ 119.7 (q, *J* = 320.8 Hz), 122.0, 127.2, 127.6, 128.0, 129.0, 129.1, 129.2, 129.5, 129.7, 130.9, 135.2, 136.9, 139.7, 139.7; IR ν_{max} (film): 3674.30, 3336.73, 2987.26, 2900.12, 1653.06, 1405.40, 1393.48, 1249.68, 1065.80, 1056.73, 1012.24, 891.84 cm^{-1}; HRMS m/z calcd for $C_{19}H_{13}F_3NO_2S$ $[M-H]^+$: 376.0619; found: 376.0623.

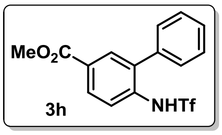

Methyl 6-(trifluoromethylsulfonamido)-[1,1'-biphenyl]-3-carboxylate (3h): The product was obtained as a white solid. Yield: 83%; ^{1}H NMR (400 MHz, $CDCl_3$) δ 3.92 (s, 3H), 6.83 (s, 1H), 7.33 (d, *J* = 7.6 Hz, 2H), 7.48-7.56 (m, 3H), 7.75 (d, *J* = 8.8 Hz, 1H), 8.00 (s, 1H), 8.07 (dd, *J* = 8.8 Hz, *J* = 1.6 Hz, 1H); ^{13}C (100 MHz, $CDCl_3$) δ 52.5, 119.6 (q, *J* = 320.9 Hz), 119.7, 127.9, 129.2, 129.4, 129.9, 130.5, 132.3, 133.5, 135.6, 135.9, 166.1; IR ν_{max} (film): 3674.34, 3344.90, 2987.37, 2900.08, 1727.82, 1506.71, 1393.43, 1378.49, 1231.41, 1141.28, 1065.84, 891.83 cm^{-1}; HRMS m/z calcd for $C_{15}H_{11}F_3NO_4S$ $[M-H]^+$: 358.0361; found: 358.0372.

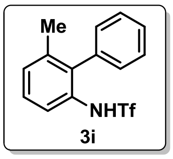

1,1,1-trifluoro-*N*-(6-methyl-[1,1'-biphenyl]-2-yl)methanesulfonamide (3i): The product was obtained as a colorless oil. Yield: 63%; ^{1}H NMR (400 MHz,

$CDCl_3$) δ 2.04 (s, 3H), 6.00 (s, 1H), 7.16-7.19 (m, 3H), 7.29 (t, *J* = 7.6 Hz, 1H), 7.44-7.54 (m, 4H); ^{13}C (100 MHz, $CDCl_3$) δ 20.8, 118.3, 119.7 (q, *J* = 321.1 Hz), 128.1, 128.6, 128.9, 129.4, 129.9, 132.4, 134.1, 135.5, 138.0; IR ν_{max} (film): 3308.16, 2944.90, 2830.61, 1446.94, 1118.37, 1020.41, 624.49 cm^{-1}; HRMS m/z calcd for $C_{14}H_{11}F_3NO_2S$ $[M-H]^+$: 314.0463; found: 314.0460.

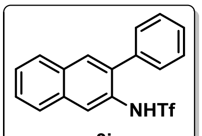

3j

1,1,1-trifluoro-*N*-(3-phenylnaphthalen-2-yl)methanesulfonamide (3j): The product was obtained as a white solid. Yield: 73%; ^1H NMR (400 MHz, $CDCl_3$) δ 7.43 (d, *J* = 7.6 Hz, 2H), 7.53-7.60 (m, 5H), 7.81 (s, 1H), 7.85 (d, *J* = 7.6 Hz, 1H), 7.92 (d, *J* = 7.6 Hz, 1H), 8.13 (s, 1H); ^{13}C (100 MHz, $CDCl_3$) δ 119.3, 119.8 (q, *J* = 321.0 Hz), 126.9, 127.3, 127.8, 128.0, 129.0, 129.5, 129.6, 129.7, 130.2, 131.5, 133.1, 133.5, 136.8; IR ν_{max} (film): 3674.35, 3336.73, 2987.22, 2900.14, 1651.02, 1405.49, 1393.45, 1249.69, 1229.00, 1065.79, 1056.71, 1012.24 cm^{-1}; HRMS m/z calcd for $C_{17}H_{11}F_3NO_2S$ $[M-H]^+$: 350.0463; found: 350.0471.

***N*-(4'-(tert-butyl)-[1,1'-biphenyl]-2-yl)-1,1,1-trifluoromethanesulfonamide (3k):** The product

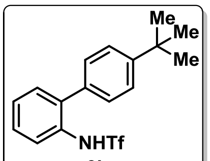

3k

was obtained as a colorless oil. Yield: 94%; ^1H NMR (400 MHz, $CDCl_3$) δ 1.38 (s, 9H), 7.24-7.30 (m, 4H), 7.36-7.40 (m, 1H), 7.52 (d, *J* = 8.4 Hz, 2H), 7.64 (d, *J* = 8.4 Hz, 1H); ^{13}C (100 MHz, $CDCl_3$) δ 31.4, 34.9, 119.7 (q, *J* = 320.8 Hz), 121.0, 126.5, 126.6, 128.9, 128.9, 131.0, 131.9, 133.7, 134.4, 151.9; IR ν_{max} (film): 3706.04, 3679.87, 2980.51, 2921.79, 2864.42, 2843.33, 1454.04, 1345.73, 1054.47, 1032.57, 1010.05 cm^{-1}; HRMS m/z calcd for $C_{17}H_{17}F_3NO_2S$ $[M-H]^+$: 356.0932; found: 356.0931.

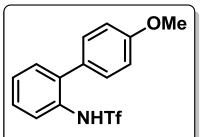

3l

1,1,1-trifluoro-*N*-(4’-methoxy-[1,1’-biphenyl]-2-yl)methanesulfonamide (3l): The product was obtained as a colorless oil. Yield: 45%; ^1H NMR (400 MHz, $CDCl_3$) δ 3.90 (s, 3H), 7.05 (d, *J* = 8.8 Hz, 2H), 7.26-7.31 (m, 4H), 7.39-7.42 (m, 1H), 7.65 (d, *J* = 8.4 Hz, 1H); ^{13}C (100 MHz, $CDCl_3$) δ 55.6, 115.0, 119.7 (q, *J* = 320.7 Hz), 121.1, 126.5, 128.8, 128.9, 130.4, 131.1, 132.0, 134.3, 160.0; IR ν_{max} (film): 3674.23, 3340.82, 2987.33, 2900.08, 1405.47, 1393.48, 1140.87, 1065.81, 1056.74, 891.68, 602.04 cm^{-1}; HRMS m/z calcd for $C_{14}H_{11}F_3NO_3S$ $[M-H]^+$: 330.0412; found: 330.0419.

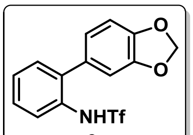

3m

***N*-(2-(benzo[d][1,3]dioxol-5-yl)phenyl)-1,1,1-trifluoromethanesulfonamide (3m):** The product was obtained as a colorless oil. Yield: 71%; ^1H NMR (400 MHz, $CDCl_3$) δ 6.06 (s, 2H), 6.75-6.77 (m, 3H), 6.93 (d, *J* = 8.0 Hz, 1H), 7.26-7.28 (m, 1H), 7.36-7.40 (m, 1H), 7.62 (d, *J* = 8.4 Hz, 1H); ^{13}C (100 MHz, $CDCl_3$) δ 101.7,

109.2, 109.6, 119.7 (q, *J* = 320.9 Hz), 121.1, 122.7, 126.5, 129.0, 130.3, 131.0, 132.0, 134.2, 148.2, 148.7; IR ν_{max} (film): 3417.50, 3186.02, 2980.56, 2972.22, 2921.85, 2864.25, 2843.33, 1660.69, 1478.25, 1345.70, 1207.71, 1054.40, 1032.71, 1008.55 cm^{-1}; HRMS m/z calcd for $C_{14}H_9F_3NO_4S$ $[M-H]^+$: 344.0204; found: 344.0205.

***N*-(3',5'-dimethoxy-[1,1'-biphenyl]-2-yl)-1,1,1-trifluoromethanesulfonamide (3n):** The product

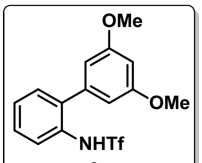

3n

was obtained as a colorless oil. Yield: 68%; ^1H NMR (400 MHz, $CDCl_3$) δ 3.82 (s, 6H), 6.43 (d, *J* = 2.0 Hz, 2H), 6.52 (t, *J* = 2.4 Hz, 1H), 6.84 (s, 1H), 7.28-7.31 (m, 2H), 7.37-7.42 (m, 1H), 7.63 (d, *J* = 8.0 Hz, 1H); ^{13}C (100 MHz, $CDCl_3$) δ 55.6, 100.7, 107.1, 119.7 (q, *J* = 320.8 Hz), 121.0, 126.4, 129.1, 130.5, 131.7, 134.4, 138.6, 161.7; IR ν_{max} (film): 3705.15, 3679.88, 2980.51, 2921.68, 2864.40, 2843.33, 1454.18, 1345.73, 1054.41, 1032.56, 1011.91 cm^{-1}; HRMS m/z calcd for $C_{15}H_{13}F_3NO_4S$ $[M-H]^+$: 360.0517; found: 360.0523.

1,1,1-trifluoro-*N*-(4'-(methylthio)-[1,1'-biphenyl]-2-yl)methanesulfonamide (3o): The product

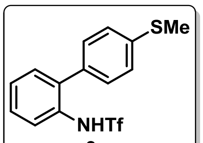

3o

was obtained as a colorless oil. Yield: 82%; ^1H NMR (400 MHz, $CDCl_3$) δ 2.54 (s, 3H), 7.24-7.42 (m, 7H), 7.62 (d, *J* = 8.4 Hz, 1H); ^{13}C (100 MHz, $CDCl_3$) δ 15.6, 119.7 (q, *J* = 320.6 Hz), 121.6, 126.7, 127.0, 129.1, 129.6, 130.9, 131.8, 133.2, 134.2, 139.9; IR ν_{max} (film): 3705.64, 3679.91, 2972.22, 2921.76, 2864.37, 2843.34, 1454.09, 1345.72, 1054.36, 1032.51, 1011.55 cm^{-1}; HRMS m/z calcd for $C_{14}H_{11}F_3NO_2S_2$ $[M-H]^+$: 346.0183; found: 346.0182.

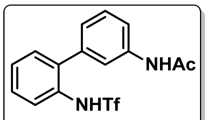

3p

***N*-(2'-(trifluoromethylsulfonamido)-[1,1'-biphenyl]-3-yl)acetamide (3p):** The product was obtained as a colorless oil. Yield: 90%; ^1H NMR (400 MHz, $CDCl_3$) δ 2.10 (s, 3H), 7.07 (d, *J* = 7.2 Hz, 1H), 7.26-7.31 (m, 2H), 7.36-7.40 (m, 2H), 7.45 (d, *J* = 8.0 Hz, 1H), 7.52 (s, 1H), 7.56 (d, *J* = 8.0 Hz, 1H), 7.65 (s, 1H); ^{13}C (100 MHz, $CDCl_3$) δ 24.5, 119.6 (q, *J* = 320.6 Hz), 119.8, 120.9, 123.7, 125.2, 127.2, 129.1, 129.9, 130.8, 131.6, 136.0, 138.1, 138.4, 169.2; IR ν_{max} (film): 3705.76, 3679.89, 2972.24, 2921.76, 2864.34, 2843.39, 1345.69, 1054.61, 1032.68, 1003.51 cm^{-1}; HRMS m/z calcd for $C_{15}H_{12}F_3N_2O_3S$ $[M-H]^+$: 357.0521; found: 357.0521.

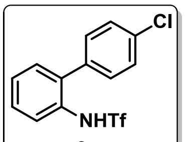

3q

***N*-(4'-chloro-[1,1'-biphenyl]-2-yl)-1,1,1-trifluoromethanesulfonamide (3q):** The product was obtained as a white solid. Yield: 83%; ^1H NMR (400 MHz, $CDCl_3$) δ 6.55 (s, 1H), 7.26-7.28 (m, 3H), 7.33 (t, *J* = 7.2 Hz, 1H), 7.42 (dt, *J* =

7.2 Hz, J = 1.6 Hz, 1H), 7.49 (d, J = 8.4 Hz, 2H), 7.61 (d, J = 8.4 Hz, 1H); ^{13}C (100 MHz, $CDCl_3$) δ 119.6 (q, J = 320.5 Hz), 122.6, 127.1, 129.4, 129.7, 130.6, 130.9, 131.6, 134.3, 135.1, 135.4; IR ν_{max} (film): 3418.03, 3187.38, 2980.62, 2864.18, 2843.46, 1648.93, 1477.95, 1372.21, 1205.21, 1145.74, 1054.95, 1006.55 cm^{-1}; HRMS m/z calcd for $C_{13}H_8F_3NO_2SCl$ $[M-H]^+$: 333.9916; found: 333.9924.

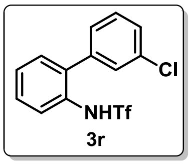

***N*-(3’-chloro-[1,1’-biphenyl]-2-yl)-1,1,1-trifluoromethanesulfonamide (3r):** The product was obtained as a colorless oil. Yield: 85%; ^{1}H NMR (400 MHz, $CDCl_3$) δ 6.64 (s, 1H), 7.25 (m, 1H), 7.31-7.39 (m, 3H), 7.44-7.48 (m, 3H), 7.66 (d, J = 8.4 Hz, 1H); ^{13}C (100 MHz, $CDCl_3$) δ 119.6 (q, J = 320.5 Hz), 122.7, 127.1, 127.3, 128.9, 129.5, 129.6, 130.6, 130.8, 131.5, 134.2, 135.4, 138.8; IR ν_{max} (film): 3674.29, 3336.73, 2987.21, 2900.15, 1655.10, 1405.72, 1393.47, 1380.99, 1249.67, 1065.79, 1056.68, 1012.24, 891.84, 604.08 cm^{-1}; HRMS m/z calcd for $C_{13}H_8ClF_3NO_2S$ $[M-H]^+$: 333.9916; found: 333.9919.

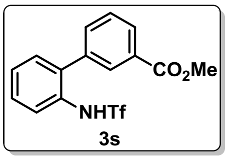

Methyl 2’-(trifluoromethylsulfonamido)-[1,1’-biphenyl]-3-carboxylate: (3s): The product was obtained as a white solid. Yield: 55%; ^{1}H NMR (400 MHz, $CDCl_3$) δ 3.96 (s, 3H), 6.72 (s, 1H), 7.33-7.40 (m, 2H), 7.46 (qt, J = 8.0 Hz, J = 1.2 Hz, 1H), 7.56-7.67 (m, 3H), 8.03 (s, 1H), 8.14 (d, J = 7.2 Hz, 1H); ^{13}C (100 MHz, $CDCl_3$) δ 52.6, 119.6 (q, J = 320.5 Hz), 123.2, 127.3, 129.5, 129.5, 129.8, 130.4, 131.0, 131.2, 131.5, 133.7, 134.9, 137.5, 166.6; IR ν_{max} (film): 3674.34, 3342.86, 2987.32, 2900.06, 1558.04, 1393.43, 1249.71, 1065.81, 1056.81, 891.72, 567.35 cm^{-1}; HRMS m/z calcd for $C_{15}H_{11}F_3NO_4S$ $[M-H]^+$: 358.0361; found: 358.0363.

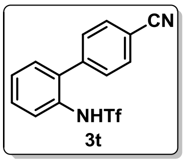

***N*-(4'-cyano-[1,1'-biphenyl]-2-yl)-1,1,1-trifluoromethanesulfonamide (3t):** The product was obtained as a colorless oil. Yield: 88%; ^{1}H NMR (400 MHz, $CDCl_3$) δ 6.73 (s, 1H), 7.31 (dd, J = 7.6 Hz, J = 1.6 Hz, 1H), 7.41 (dt, J = 7.6 Hz, J = 1.2 Hz, 1H), 7.46-7.51 (m, 3H), 7.60 (dd, J = 8.4 Hz, J = 0.8 Hz, 1H), 7.79 (d, J = 8.4 Hz, 2H); ^{13}C (100 MHz, $CDCl_3$) δ 112.5, 118.4, 119.5 (q, J = 320.2 Hz), 124.6, 127.9, 130.1, 130.3, 130.8, 131.1, 133.0, 135.1, 142.2; IR ν_{max} (film): 3679.90, 2980.57, 2864.26, 2843.45, 1649.25, 1371.93, 1208.74, 1143.98, 1054.59, 1032.74, 1003.89 cm^{-1}; HRMS m/z calcd for $C_{14}H_8F_3N_2O_2S$ $[M-H]^+$: 325.0259; found: 325.0256.

1,1,1-trifluoro-*N*-(4’-(trifluoromethyl)-[1,1’-biphenyl]-2-yl)methanesulfonamide (3u): The

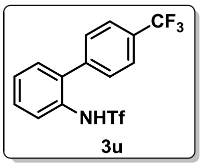

product was obtained as a colorless oil. Yield: 87%; ^{1}H NMR (400 MHz, $CDCl_3$) δ 6.62 (s, 1H), 7.34 (d, *J* = 7.6 Hz, 1H), 7.41 (t, *J* = 7.2 Hz, 1H), 7.47-7.51 (m, 3H), 7.66 (d, *J* = 8.4 Hz, 1H), 7.80 (d, *J* = 7.6 Hz, 2H); ^{13}C (100 MHz, $CDCl_3$) δ 119.6 (q, *J* = 320.3 Hz), 123.4, 124.0 (q, *J* = 270.6 Hz), 126.3 (q, *J* = 3.7 Hz), 127.5, 129.8, 129.8, 130.9, 131.0 (q, *J* = 32.7 Hz), 131.3, 134.6, 140.8; IR ν_{max} (film): 3674.24, 3338.78, 2987.33, 2900.07, 1393.46, 1249.66, 1065.87, 1056.80, 1014.29, 891.79, 585.71 cm^{-1}; HRMS m/z calcd for $C_{14}H_8F_6NO_2S$ $[M-H]^+$: 368.0180; found: 368.0178.

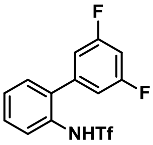

3v

***N*-(3',5'-difluoro-[1,1'-biphenyl]-2-yl)-1,1,1-trifluoromethanesulfonamide (3v):** The product was obtained as a colorless oil. Yield: 96%; ^{1}H NMR (400 MHz, $CDCl_3$) δ 6.87-6.93 (m, 3H), 7.29 (dd, *J* = 7.6 Hz, *J* = 1.6 Hz, 1H), 7.36 (dt, *J* = 7.6 Hz, *J* = 0.8 Hz, 1H), 7.45 (dt, *J* = 7.6 Hz, *J* = 1.6 Hz, 1H), 7.62 (dd, *J* = 8.0 Hz, *J* = 0.8 Hz, 1H); ^{13}C (100 MHz, $CDCl_3$) δ 104.3 (t, *J* = 24.9 Hz), 112.6 (dd, *J* = 18.3 Hz, *J* = 7.3 Hz), 119.6 (q, *J* = 320.5 Hz), 123.5, 127.4, 129.9, 130.7, 131.3, 133.8, 140.3 (t, *J* = 9.4 Hz), 163.4 (dd, *J* = 249.9 Hz, *J* = 12.9 Hz); IR ν_{max} (film): 3705.66, 3679.86, 2980.52, 2921.77, 2864.40, 2843.33, 1345.68, 1054.43, 1032.58, 1010.19 cm^{-1}; HRMS m/z calcd for $C_{13}H_7F_5NO_2S$ $[M-H]^+$: 336.0118; found: 336.0125.

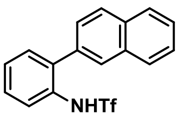

3w

1,1,1-trifluoro-*N*-(2-(naphthalen-2-yl)phenyl)methanesulfonamide (3w): The product was obtained as a white solid. Yield: 88%; ^{1}H NMR (400 MHz, $CDCl_3$) δ 6.78 (s, 1H), 7.37-7.50 (m, 4H), 7.61-7.63 (m, 2H), 7.73 (d, *J* = 8.0 Hz, 1H), 7.86 (s, 1H), 7.91-7.98 (m, 2H), 8.03 (d, *J* = 8.4 Hz, 1H); ^{13}C (100 MHz, $CDCl_3$) δ 119.7 (q, *J* = 320.7 Hz), 121.6, 126.6, 126.7, 127.1, 127.2, 128.0, 128.2, 128.5, 129.2, 129.5, 131.1, 131.9, 133.1, 133.5, 134.2, 134.8; IR ν_{max} (film): 3674.28, 3342.86, 2987.34, 2900.07, 1653.06, 1405.94, 1393.46, 1229.24, 1065.81, 1056.79, 891.87, 602.04 cm^{-1}; HRMS m/z calcd for $C_{17}H_{11}F_3NO_2S$ $[M-H]^+$: 350.0463; found: 350.0465.

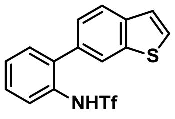

3x

***N*-(2-(benzo[b]thiophen-6-yl)phenyl)-1,1,1-trifluoromethanesulfonamide (3x):** The product was obtained as a colorless oil. Yield: 60%; ^{1}H NMR (400 MHz, $CDCl_3$) δ 7.29 (dd, *J* = 8.4 Hz, *J* = 1.6 Hz, 1H), 7.32-7.45 (m, 4H), 7.58 (d, *J* = 5.2 Hz, 1H), 7.67 (d, *J* = 8.0 Hz, 1H), 7.78 (d, *J* = 1.2 Hz, 1H), 8.02 (d, *J* = 8.4 Hz, 1H); ^{13}C (100 MHz, $CDCl_3$) δ 119.7 (q, *J* = 320.6 Hz), 121.2, 123.7, 124.0, 124.2, 125.0, 126.5, 128.4, 129.1, 131.2, 132.1, 132.8, 134.5, 140.1, 140.4; IR ν_{max} (film): 3706.00, 3679.89, 2980.51, 2972.23, 2921.83, 2864.43, 2843.34, 1454.05, 1345.73, 1054.47, 1032.58, 1010.32 cm^{-1}; HRMS

m/z calcd for $C_{15}H_9F_3NO_2S_2$ $[M-H]^+$: 356.0027; found: 356.0029.

1,1,1-trifluoro-*N*-(3'-methoxy-5-methyl-[1,1'-biphenyl]-2-yl)methanesulfonamide (3y): The

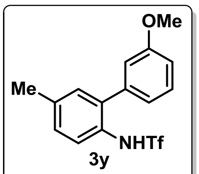

3y

product was obtained as a colorless oil. Yield: 72%; ^{1}H NMR (400 MHz, $CDCl_3$) δ 2.37 (s, 3H), 3.83 (s, 3H), 6.48 (s, 1H), 6.83 (s, 1H), 6.86 (d, *J* = 7.2 Hz, 1H), 6.96 (dd, *J* = 8.4 Hz, *J* = 2.4 Hz, 1H), 7.12 (s, 1H), 7.18 (d, *J* = 8.4 Hz, 1H), 7.39 (t, *J* = 8.0 Hz, 1H), 7.49 (d, *J* = 8.4 Hz, 1H); ^{13}C (100 MHz, $CDCl_3$) δ 21.0, 55.5, 114.3, 114.7, 121.3, 122.1, 122.9 (q, *J* = 320.4 Hz), 129.0, 129.6, 130.5, 131.3, 135.1, 136.8, 138.5, 160.3; IR ν_{max} (film): 3302.26, 2942.63, 2830.57, 1448.39, 1210.57, 1115.45, 1021.10, 611.83 cm^{-1}; HRMS m/z calcd for $C_{15}H_{13}F_3NO_3S$ $[M-H]^+$: 344.0568; found: 344.0563.

***N*-(4'-chloro-5-methyl-[1,1'-biphenyl]-2-yl)-1,1,1-trifluoromethanesulfonamide (3z):** The

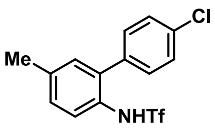

3z

product was obtained as a colorless oil. Yield: 79%; ^{1}H NMR (400 MHz, $CDCl_3$) δ 2.38 (s, 3H), 6.44 (s, 1H), 7.09 (s, 1H), 7.21 (d, *J* = 8.4 Hz, 1H), 7.25 (d, *J* = 8.4 Hz, 2H), 7.46-7.48 (m, 3H); ^{13}C (100 MHz, $CDCl_3$) δ 21.0, 119.6 (q, *J* = 320.6 Hz), 123.2, 128.8, 129.6, 130.0, 130.6, 131.5, 134.7, 134.9, 135.7, 137.4; IR ν_{max} (film): 3379.59, 2944.90, 2832.65, 1487.01, 1418.79, 1365.42, 1233.55, 1205.22, 1142.46, 1091.88, 1024.49, 907.51, 732.34, 621.72 cm^{-1}; HRMS m/z calcd for $C_{14}H_{10}F_3NO_2SCl$ $[M-H]^+$: 348.0073; found: 348.0071.

***N*-(4'-(tert-butyl)-4-chloro-[1,1'-biphenyl]-2-yl)-1,1,1-trifluoromethanesulfonamide (3aa):**

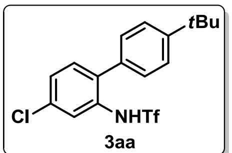

3aa

The product was obtained as a colorless oil. Yield: 89%; ^{1}H NMR (400 MHz, $CDCl_3$) δ 1.38 (s, 9H), 7.21-7.23 (m, 3H), 7.27 (d, *J* = 6.4 Hz, 1H), 7.53 (d, *J* = 8.0 Hz, 2H), 7.67 (s, 1H); ^{13}C (100 MHz, $CDCl_3$) δ 31.4, 34.9, 119.6 (q, *J* = 320.6 Hz), 120.7, 126.6, 126.8, 128.8, 131.9, 132.4, 132.6, 132.9, 134.6, 152.4; IR ν_{max} (film): 3377.55, 2963.27, 2830.61, 1602.04, 1486.86, 1431.91, 1408.80, 1365.65, 1233.17, 1204.08, 1141.56, 1024.49, 954.35, 906.54, 732.57, 607.91 cm^{-1}; HRMS m/z calcd for $C_{17}H_{16}F_3NO_2SCl$ $[M-H]^+$: 390.0542; found: 390.0543.

***N*-(4-chloro-3',5'-difluoro-[1,1'-biphenyl]-2-yl)-1,1,1-trifluoromethanesulfonamide (3ab):** The

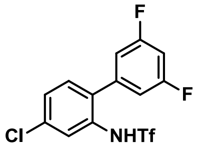

3ab

product was obtained as a colorless oil. Yield: 87%; ^{1}H NMR (400 MHz, $CDCl_3$) δ 6.85 (dd, *J* = 12.8 Hz, *J* = 7.2 Hz, 2H), 6.94 (t, *J* = 12.8 Hz, 1H), 7.22 (d, *J* = 8.0 Hz, 1H), 7.33 (d, *J* = 8.0 Hz, 1H), 7.66 (s, 1H); ^{13}C (100

MHz, $CDCl_3$) δ 104.8 (t, *J* = 24.8 Hz), 112.5 (dd, *J* = 18.3 Hz, *J* = 7.5 Hz), 119.6 (q, *J* = 320.5 Hz), 122.8, 127.4, 131.4, 131.6, 132.5, 135.8, 139.1 (t, *J* = 9.5 Hz), 163.6 (dd, *J* = 250.8 Hz, *J* = 12.9 Hz); IR ν_{max} (film): 3283.73, 3093.88, 2830.61, 1622.07, 1595.14, 1497.74, 1462.10, 1435.73, 1399.38, 1369.15, 1236.57, 1218.07, 1199.02, 1140.13, 1122.01, 1020.41, 990.54, 957.80, 866.03, 732.19, 601.96 cm^{-1}; HRMS m/z calcd for $C_{13}H_6F_5NO_2SCl$ $[M-H]^+$: 369.9728; found: 369.9728.

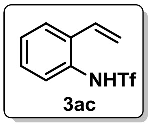

1,1,1-trifluoro-*N*-(2-vinylphenyl)methanesulfonamide (3ac): The product was obtained as a colorless oil. Yield: 40%; ^1H NMR (400 MHz, $CDCl_3$) δ 5.51 (d, *J* = 10.8 Hz, 1H), 5.76 (d, *J* = 17.6 Hz, 1H), 6.92 (dd, *J* = 17.6 Hz, *J* = 10.8 Hz, 1H), 7.32-7.35 (m, 2H), 7.42-7.44 (m, 1H), 7.53-7.56 (m, 1H); ^{13}C (100 MHz, $CDCl_3$) δ 119.5, 119.9 (q, *J* = 320.3 Hz), 126.4, 127.2, 128.6, 129.1, 130.7, 131.1, 134.3; IR ν_{max} (film): 3674.16, 3326.53, 2987.14, 2900.15, 1655.10, 1405.30, 1393.49, 1249.69, 1229.22, 1065.76, 1056.66, 1012.24, 891.72 cm^{-1}; HRMS m/z calcd for $C_9H_7F_3NO_2S$ $[M-H]^+$: 250.0150; found: 250.0151.

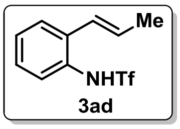

(*E*)-1,1,1-trifluoro-*N*-(2-(prop-1-en-1-yl)phenyl)methanesulfonamide (3ad): The product was obtained as a colorless oil. Yield: 57%; ^1H NMR (400 MHz, $CDCl_3$) δ 1.97 (d, *J* = 6.8 Hz, 3H), 6.24 (dq, *J* = 15.6 Hz, *J* = 6.8 Hz, 1H), 6.58 (d, *J* = 15.6 Hz, 1H), 6.60 (s, 1H), 7.29-7.33 (m, 1H), 7.43-7.48 (m, 2H); ^{13}C (100 MHz, $CDCl_3$) δ 19.1, 119.9 (q, *J* = 320.4 Hz), 124.9, 125.8, 127.5, 128.2, 128.3, 130.3, 132.1, 134.1; IR ν_{max} (film): 3674.24, 3336.73, 2987.20, 2900.15, 1653.06, 1405.45, 1393.48, 1381.32, 1249.69, 1229.00, 1065.31, 1012.24, 891.84 cm^{-1}; HRMS m/z calcd for $C_{10}H_9F_3NO_2S$ $[M-H]^+$: 264.0306; found: 264.0310.

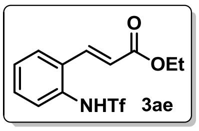

(*E*)-ethyl 3-(2-(trifluoromethylsulfonamido)phenyl)acrylate (3ae): The product was obtained as a colorless oil. Yield: 85%; ^1H NMR (400 MHz, $CDCl_3$) δ 1.34 (t, *J* = 7.2 Hz, 3H), 4.28 (q, *J* = 7.2 Hz, 2H), 6.49 (d, *J* = 16.0 Hz, 1H), 7.40 (t, *J* = 7.6 Hz, 1H), 7.48 (t, *J* = 7.6 Hz, 1H), 7.56 (d, *J* = 7.6 Hz, 1H), 7.68 (d, *J* = 7.6 Hz, 1H), 8.12 (d, *J* = 16.0 Hz, 1H); ^{13}C (100 MHz, $CDCl_3$) δ 14.3, 61.4, 119.9 (q, *J* = 320.5 Hz), 121.8, 127.5, 128.0, 128.8, 131.1, 131.4, 132.8, 139.0, 167.3; IR ν_{max} (film): 3674.15, 3320.41, 2987.13, 2900.16, 1655.10, 1405.32, 1393.49, 1381.16, 1065.76, 1056.65, 1010.20, 891.70 cm^{-1}; HRMS m/z calcd for $C_{12}H_{11}F_3NO_4S$ $[M-H]^+$: 322.0361; found: 322.0368.

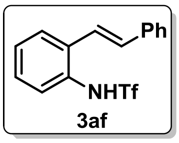

(*E*)-1,1,1-trifluoro-*N*-(2-styrylphenyl)methanesulfonamide (3af): The product was obtained as a white solid. Yield: 56%; ^1H NMR (400 MHz, $CDCl_3$)

δ 6.69 (s, 1H), 7.12 (d, *J* = 16.0 Hz, 1H), 7.29 (d, *J* = 6.8 Hz, 1H), 7.34-7.44 (m, 5H), 7.48 (d, *J* = 7.6 Hz, 1H), 7.55 (d, *J* = 7.6 Hz, 2H), 7.71 (d, *J* = 7.6 Hz, 1H); ^{13}C (100 MHz, $CDCl_3$) δ 119.9 (q, *J* = 320.3 Hz), 121.9, 127.0, 127.0, 127.2, 128.7, 128.8, 128.9, 129.0, 130.8, 133.8, 134.3, 136.6; IR ν_{max} (film): 3674.26, 2987.09, 2900.17, 1651.02, 1405.56, 1393.47, 1381.39, 1249.70, 1228.65, 1065.76, 1056.63, 1008.16, 891.69 cm^{-1}; HRMS m/z calcd for $C_{15}H_{11}F_3NO_2S$ $[M-H]^+$: 326.0463; found: 326.0469.

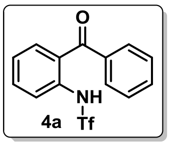

***N*-(2-benzoylphenyl)-1,1,1-trifluoromethanesulfonamide (4a):** The product was obtained as a white solid. Yield: 84%; ^{1}H NMR (400 MHz, $CDCl_3$) δ 7.31 (t, *J* = 7.6 Hz, 1H), 7.55 (t, *J* = 7.6 Hz, 2H), 7.64-7.69 (m, 3H), 7.75 (d, *J* = 7.6 Hz, 2H), 7.85 (d, *J* = 8.4 Hz, 1H), 11.0 (s, 1H); ^{13}C (100 MHz, $CDCl_3$) δ 119.9 (q, *J* = 321.2 Hz), 121.8, 124.8, 125.5, 128.7, 130.1, 133.3, 134.0, 134.6, 137.5, 137.5, 199.4; IR ν_{max} (film): 3674.37, 3330.61, 2987.20, 2900.14, 1653.06, 1405.40, 1393.48, 1249.72, 1229.49, 1065.78, 1056.69, 1014.29, 893.88 cm^{-1}; HRMS m/z calcd for $C_{14}H_9F_3NO_3S$ $[M-H]^+$: 328.0255; found: 328.0257.

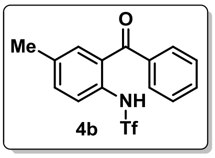

***N*-(2-benzoyl-4-methylphenyl)-1,1,1-trifluoromethanesulfonamide (4b):** The product was obtained as a white solid. Yield: 84%; ^{1}H NMR (400 MHz, $CDCl_3$) δ 2.48 (s, 3H), 7.09 (d, *J* = 8.0 Hz, 1H), 7.51-7.57 (m, 3H), 7.64-7.66 (m, 2H), 7.71 (d, *J* = 7.6 Hz, 2H), 11.2 (s, 1H); ^{13}C (100 MHz, $CDCl_3$) δ 22.2, 119.9 (q, *J* = 321.2 Hz), 122.0, 122.6, 125.5, 128.6, 130.0, 133.0, 134.2, 137.9, 137.9, 146.4, 199.3; IR ν_{max} (film): 3674.29, 3324.49, 2987.18, 2900.16, 1655.10, 1405.63, 1393.47, 1381.38, 1249.74, 1229.27, 1065.78, 1056.68, 1008.16, 893.88 cm^{-1}; HRMS m/z calcd for $C_{15}H_{11}F_3NO_3S$ $[M-H]^+$: 342.0412; found: 342.0415.

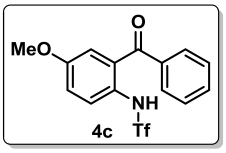

***N*-(2-benzoyl-4-methoxyphenyl)-1,1,1-trifluoromethanesulfonamide (4c):** The product was obtained as a white solid. Yield: 68%; ^{1}H NMR (400 MHz, $CDCl_3$) δ 3.81 (s, 3H), 7.11 (d, *J* = 2.8 Hz, 1H), 7.17 (dd, *J* = 8.8 Hz, *J* = 2.8 Hz, 1H), 7.55 (t, *J* = 7.6 Hz, 2H), 7.68 (t, *J* = 7.6 Hz, 1H), 7.73 (d, *J* = 9.2 Hz, 1H), 7.79 (d, *J* = 7.6 Hz, 2H), 10.01 (s, 1H); ^{13}C (100 MHz, $CDCl_3$) δ 55.9, 118.8, 119.0, 119.9 (q, *J* = 321.7 Hz), 125.3, 128.7, 128.7, 129.1, 130.2, 133.5, 137.2, 156.8, 198.3; IR ν_{max} (film): 3674.28, 3338.78, 2987.15, 2900.17, 1405.90, 1393.47, 1381.38, 1065.77, 1056.66, 1010.20, 891.69 cm^{-1}; HRMS m/z calcd for $C_{15}H_{11}F_3NO_4S$ $[M-H]^+$: 358.0361; found: 358.0359.

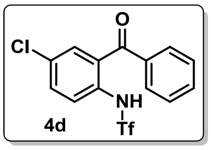

***N*-(2-benzoyl-4-chlorophenyl)-1,1,1-trifluoromethanesulfonamide (4d):** The product was obtained as a white solid. Yield: 64%; ^{1}H NMR (400 MHz, $CDCl_3$) δ 7.53-7.60 (m, 4H), 7.69 (t, J = 7.6 Hz, 1H), 7.72-7.78 (m, 3H), 10.58 (s, 1H); ^{13}C (100 MHz, $CDCl_3$) δ 119.8 (q, J = 321.2 Hz), 123.8, 127.2, 128.9, 130.2, 130.8, 133.1, 133.8, 134.3, 135.7, 136.7, 197.9; IR ν_{max} (film): 3334.69, 2942.86, 2830.61, 1642.19, 1596.21, 1482.92, 1421.46, 1293.17, 1230.07, 1205.44, 1140.96, 1022.45, 924.54, 702.81, 609.52 cm^{-1}; HRMS m/z calcd for $C_{14}H_8F_3NO_3SCl$ $[M-H]^+$: 361.9866; found: 361.9867.

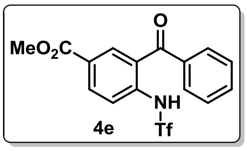

Methyl 3-benzoyl-4-(trifluoromethylsulfonamido)benzoate (4e): The product was obtained as a white solid. Yield: 85%; ^{1}H NMR (400 MHz, $CDCl_3$) δ 3.99 (s, 3H), 7.54 (t, J = 7.6 Hz, 2H), 7.66-7.75 (m, 4H), 7.94 (d, J = 8.0 Hz, 1H), 8.43 (s, 1H), 10.51 (s, 1H); ^{13}C (100 MHz, $CDCl_3$) δ 53.1, 119.8 (q, J = 321.0 Hz), 123.3, 125.9, 128.9, 129.2, 130.3, 133.5, 133.9, 135.3, 136.9, 137.2, 165.2, 198.2; IR ν_{max} (film): 3674.29, 3330.61, 2987.12, 2900.18, 1653.06, 1405.57, 1393.46, 1381.49, 1249.72, 1229.53, 1065.76, 1056.65, 1008.16, 891.69 cm^{-1}; HRMS m/z calcd for $C_{16}H_{11}F_3NO_5S$ $[M-H]^+$: 386.0310; found: 386.0311.

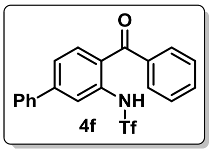

***N*-(4-benzoyl-[1,1'-biphenyl]-3-yl)-1,1,1-trifluoromethanesulfonamide (4f):** The product was obtained as a white solid. Yield: 87%; ^{1}H NMR (400 MHz, $CDCl_3$) δ 7.45-7.55 (m, 6H), 7.64-7.67 (m, 3H), 7.72 (d, J = 8.4 Hz, 1H), 7.75 (d, J = 7.6 Hz, 2H), 8.06 (s, 1H); ^{13}C (100 MHz, $CDCl_3$) δ 120.0, 120.0 (q, J = 321.2 Hz), 123.1, 123.7, 127.5, 128.7, 129.2, 129.3, 130.0, 133.2, 134.6, 137.8, 138.4, 138.7, 147.7, 199.2; IR ν_{max} (film): 3674.28, 3338.78, 2987.14, 2900.17, 1657.14, 1405.97, 1393.49, 1381.46, 1249.81, 1229.78, 1065.77, 1056.65, 1014.29, 891.70, 697.96, 597.96 cm^{-1}; HRMS m/z calcd for $C_{20}H_{13}F_3NO_3S$ $[M-H]^+$: 404.0568; found: 404.0568.

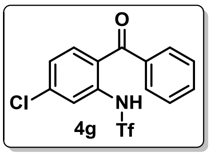

***N*-(2-benzoyl-5-chlorophenyl)-1,1,1-trifluoromethanesulfonamide (4g):** The product was obtained as a white solid. Yield: 75%; ^{1}H NMR (400 MHz, $CDCl_3$) δ 7.27 (d, J = 8.8 Hz, 1H), 7.56 (t, J = 7.6 Hz, 2H), 7.62 (d, J = 8.4 Hz, 1H), 7.67-7.72 (m, 3H), 7.87 (s, 1H); ^{13}C (100 MHz, $CDCl_3$) δ 119.8 (q, J = 320.9 Hz), 121.6, 123.3, 125.0, 128.8, 130.0, 133.5, 135.0, 137.3, 139.0, 141.2, 198.7; IR ν_{max} (film): 3674.28, 3326.53, 2987.12, 2900.18, 1653.06, 1405.60, 1393.47, 1381.45, 1249.77, 1229.60, 1065.76, 1056.64, 1006.12, 891.68 cm^{-1}; HRMS m/z calcd for $C_{14}H_8ClF_3NO_3S$ $[M-H]^+$: 361.9866; found:

361.9876.

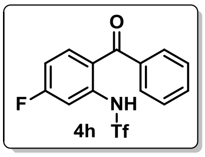

***N*-(2-benzoyl-5-fluorophenyl)-1,1,1-trifluoromethanesulfonamide (4h):** The product was obtained as a white solid. Yield: 87%; ^{1}H NMR (400 MHz, $CDCl_3$) δ 6.97 (t, *J* = 8.0 Hz, 1H), 7.56 (t, *J* = 8.0 Hz, 2H), 7.62 (d, *J* = 10.0 Hz, 1H), 7.66-7.75 (m, 4H), 11.54 (s, 1H); ^{13}C (100 MHz, $CDCl_3$) δ 108.7 (d, *J* = 27.7 Hz), 111.8 (d, *J* = 21.8 Hz), 119.8 (q, *J* = 320.8 Hz), 120.8 (d, *J* = 3.0 Hz), 128.8, 129.9, 133.3, 136.8 (d, *J* = 10.5 Hz), 137.6, 140.7 (d, *J* = 12.1 Hz), 165.9 (d, *J* = 256.5 Hz), 198.7; IR ν_{max} (film): 3674.30, 3338.78, 2987.10, 2900.18, 1657.14, 1405.66, 1393.47, 1381.44, 1249.74, 1230.29, 1065.76, 1056.65, 1008.16, 891.69 cm^{-1}; HRMS m/z calcd for $C_{14}H_8F_4NO_3S$ $[M-H]^+$: 346.0161; found: 346.0162.

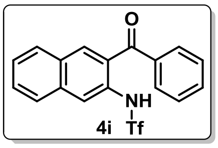

***N*-(3-benzoylnaphthalen-2-yl)-1,1,1-trifluoromethanesulfonamide (4i):** The product was obtained as a white solid. Yield: 70%; ^{1}H NMR (400 MHz, $CDCl_3$) δ 7.53-7.58 (m, 3H), 7.69 (m, 2H), 7.80-7.83 (m, 3H), 7.90 (d, *J* = 8.4 Hz, 1H), 8.17 (d, *J* = 8.0 Hz, 2H), 10.49 (s, 1H); ^{13}C (100 MHz, $CDCl_3$) δ 120.0 (q, *J* = 321.6 Hz), 121.0, 125.7, 127.4, 128.0, 128.8, 129.3, 129.6, 130.3, 130.4, 132.3, 133.5, 135.5, 136.3, 137.7, 199.1; IR ν_{max} (film): 3674.25, 3326.53, 2987.10, 2900.18, 1653.06, 1405.52, 1393.47, 1381.52, 1249.72, 1229.31, 1065.75, 1056.63, 1006.12, 891.69 cm^{-1}; HRMS m/z calcd for $C_{18}H_{11}F_3NO_3S$ $[M-H]^+$: 378.0412; found: 378.0414.

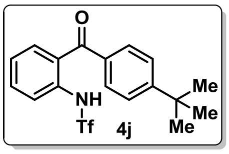

***N*-(2-(4-(tert-butyl)benzoyl)phenyl)-1,1,1-trifluoromethanesulfonamide (4j):** The product was obtained as a colorless oil. Yield: 73%; ^{1}H NMR (400 MHz, $CDCl_3$) δ 1.38 (s, 9H), 7.28 (t, *J* = 7.2 Hz, 1H), 7.53 (d, *J* = 8.4 Hz, 2H), 7.62 (dt, *J* = 8.0 Hz, *J* = 1.6 Hz, 1H), 7.68 (d, *J* = 8.4 Hz, 3H), 7.82 (d, *J* = 8.4 Hz, 1H), 10.90 (s, 1H); ^{13}C (100 MHz, $CDCl_3$) δ 31.2, 35.4, 119.9 (q, *J* = 321.3 Hz), 121.9, 124.8, 125.7, 125.9, 130.3, 133.9, 134.4, 134.7, 137.4, 157.3, 198.9; IR ν_{max} (film):3705.66, 3679.89, 2980.51, 2972.21, 2921.76, 2864.35, 2843.33, 1670.85, 1454.10, 1345.73, 1054.42, 1032.59, 1010.18 cm^{-1}; HRMS m/z calcd for $C_{18}H_{17}F_3NO_3S$ $[M-H]^+$: 384.0881; found: 384.0881.

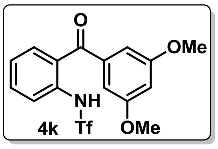

***N*-(2-(3,5-dimethoxybenzoyl)phenyl)-1,1,1-trifluoromethanesulfonamide (4k):** The product was obtained as a colorless oil. Yield: 42%; ^{1}H NMR (400 MHz, $CDCl_3$) δ 3.84 (s, 6H), 6.71 (t, *J* = 2.4 Hz, 1H), 6.81 (d, *J* = 2.4 Hz, 2H), 7.27 (dt, *J* = 7.6 Hz, *J* = 0.8 Hz, 1H), 7.63 (dt, *J* = 7.6 Hz, *J* = 1.6 Hz, 1H), 7.69 (dd, *J* = 7.6

Hz, J = 1.6 Hz, 1H), 7.81 (dd, J = 8.4 Hz, J = 0.8 Hz, 1H), 10.83 (s, 1H); ^{13}C (100 MHz, $CDCl_3$) δ 55.8, 105.3, 108.0, 119.9 (q, J = 321.1 Hz), 121.8, 124.8, 125.5, 133.9, 134.7, 137.5, 139.3, 160.8, 199.0; IR ν_{max} (film): 3705.99, 3679.90, 3411.17, 2980.54, 2972.23, 2921.81, 2864.38, 2843.33, 1661.76, 1453.98, 1345.73, 1054.51, 1032.62, 1009.45 cm^{-1}; HRMS m/z calcd for $C_{16}H_{13}F_3NO_5S$ $[M-H]^+$: 388.0467; found: 388.0469.

***N*-(2-(benzo[d][1,3]dioxole-5-carbonyl)phenyl)-1,1,1-trifluoromethanesulfonamide (4l):** The

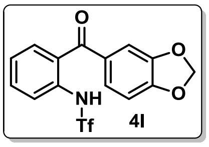

product was obtained as a colorless oil. Yield: 54%; ^{1}H NMR (400 MHz, $CDCl_3$) δ 6.11 (s, 2H), 6.90 (d, J = 8.0 Hz, 1H), 7.26-7.32 (m, 3H), 7.58-7.64 (m, 2H), 7.79 (d, J = 8.0 Hz, 1H); ^{13}C (100 MHz, $CDCl_3$) δ 102.3, 108.1, 110.0, 119.9 (q, J = 321.2 Hz), 122.5, 124.9, 126.8, 127.4, 131.6, 133.0, 134.0, 136.9, 148.3, 152.5, 196.8; IR ν_{max} (film): 3706.03, 3679.88, 2980.52, 2972.23, 2921.79, 2864.30, 2843.34, 1661.59, 1454.01, 1345.72, 1054.44, 1032.60, 1009.76 cm^{-1}; HRMS m/z calcd for $C_{15}H_9F_3NO_5S$ $[M-H]^+$: 372.0154; found: 372.0157.

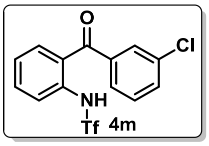

***N*-(2-(3-chlorobenzoyl)phenyl)-1,1,1-trifluoromethanesulfonamide (4m):** The product was obtained as a white solid. Yield: 75%; ^{1}H NMR (400 MHz, $CDCl_3$) δ 7.32 (t, J = 7.6 Hz, 1H), 7.49 (t, J = 7.6 Hz, 1H), 7.59-7.70 (m, 4H), 7.73 (s, 1H), 7.86 (d, J = 8.0 Hz, 1H), 10.82 (s, 1H); ^{13}C (100 MHz, $CDCl_3$) δ 119.9 (q, J = 321.2 Hz), 122.0, 125.0, 125.0, 128.2, 129.9, 130.0, 133.2, 133.8, 135.1, 135.1, 137.7, 139.2, 197.9; IR ν_{max} (film): 3674.24, 3332.65, 2987.09, 2900.18, 1653.06, 1405.52, 1393.47, 1381.50, 1249.74, 1229.53, 1065.76, 1056.62, 1006.12, 891.71 cm^{-1}; HRMS m/z calcd for $C_{14}H_8ClF_3NO_3S$ $[M-H]^+$: 361.9866; found: 361.9869.

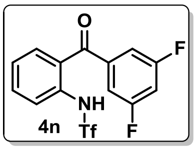

***N*-(2-(3,5-difluorobenzoyl)phenyl)-1,1,1-trifluoromethanesulfonamide (4n):** The product was obtained as a colorless oil. Yield: 84%; ^{1}H NMR (400 MHz, $CDCl_3$) δ 7.10 (tt, J = 8.4 Hz, J = 2.4 Hz, 1H), 7.23-7.25 (m, 2H), 7.32 (dt, J = 8.0 Hz, J = 0.8 Hz, 1H), 7.63 (dd, J = 8.0 Hz, J = 1.6 Hz, 1H), 7.68 (dt, J = 8.0 Hz, J = 1.6 Hz, 1H), 7.84 (d, J = 8.4 Hz, 1H); ^{13}C (100 MHz, $CDCl_3$) δ 108.6 (t, J = 25.0 Hz), 113.1 (dd, J = 19.0 Hz, J = 7.7 Hz), 119.9 (q, J = 320.9 Hz), 122.2, 124.6, 125.1, 133.6, 135.4, 137.7, 140.3 (t, J = 8.0 Hz), 162.8 (dd, J = 250.8 Hz, J = 11.7 Hz), 196.5; IR ν_{max} (film): 3418.45, 2980.53, 2972.23, 2921.80, 2864.33, 2843.32, 1666.76, 1453.94, 1345.73, 1054.48, 1032.59, 1010.40 cm^{-1}; HRMS m/z calcd for $C_{14}H_7F_5NO_3S$ $[M-H]^+$: 364.0067; found: 364.0069.

1,1,1-trifluoro-*N*-(2-(4-(trifluoromethyl)benzoyl)phenyl)methanesulfonamide (4o): The

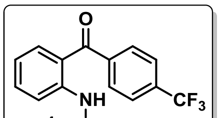
4o

product was obtained as a white solid. Yield: 79%; ^{1}H NMR (400 MHz, $CDCl_3$) δ 7.32 (t, *J* = 7.6 Hz, 1H), 7.62 (d, *J* = 7.6 Hz, 1H), 7.70 (t, *J* = 7.6 Hz, 1H), 7.81-7.89 (m, 5H), 10.90 (s, 1H); ^{13}C (100 MHz, $CDCl_3$) δ 119.9 (q, *J* = 321.1 Hz), 121.9, 123.6 (q, *J* = 271.2 Hz), 124.7, 125.0, 125.8 (q, *J* = 3.7 Hz), 130.2, 133.9, 134.6 (q, *J* = 32.7 Hz), 135.4, 137.9, 140.7, 198.4; IR ν_{max} (film): 3674.31, 3338.78, 2987.11, 2900.19, 1655.10, 1405.66, 1393.47, 1381.47, 1249.72, 1229.87, 1065.78, 1056.64, 1008.16, 891.68 cm^{-1}; HRMS m/z calcd for $C_{15}H_8F_6NO_3S$ [M-H]$^+$: 396.0129; found: 396.0132.

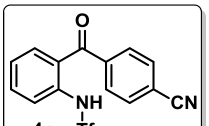
4p

***N*-(2-(4-cyanobenzoyl)phenyl)-1,1,1-trifluoromethanesulfonamide (4p):** The product was obtained as a colorless oil. Yield: 71%; ^{1}H NMR (400 MHz, $CDCl_3$) δ 7.31 (dt, *J* = 7.6 Hz, *J* = 0.8 Hz, 1H), 7.57 (dd, *J* = 8.4 Hz, *J* = 1.6 Hz, 1H), 7.69 (dt, *J* = 8.4 Hz, *J* = 1.6 Hz, 1H), 7.80-7.87 (m, 5H); ^{13}C (100 MHz, $CDCl_3$) δ 116.5, 117.8, 119.8 (q, *J* = 321.0 Hz), 122.0, 124.4, 125.0, 130.3, 132.5, 133.7, 135.6, 137.9, 141.1, 197.8; IR ν_{max} (film): 3406.32, 3186.44, 2980.57, 2972.24, 2921.75, 2864.27, 2843.33, 1666.66, 1454.03, 1345.74, 1054.56, 1032.66, 1009.44 cm^{-1}; HRMS m/z calcd for $C_{15}H_8F_3N_2O_3S$ [M-H]$^+$: 353.0208; found: 353.0213.

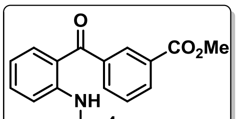
4q

Methyl 3-(2-(trifluoromethylsulfonamido)benzoyl)benzoate (4q): The product was obtained as a white solid. Yield: 76%; ^{1}H NMR (400 MHz, $CDCl_3$) δ 3.98 (s, 3H), 7.32 (t, *J* = 7.6 Hz, 1H), 7.63-7.70 (m, 3H), 7.87 (d, *J* = 8.0 Hz, 1H), 7.94 (d, *J* = 8.0 Hz, 1H), 8.33 (d, *J* = 7.6 Hz, 1H), 8.38 (s, 1H), 10.91 (s, 1H); ^{13}C (100 MHz, $CDCl_3$) δ 52.7, 119.9 (q, *J* = 321.1 Hz), 121.9, 124.9, 125.0, 129.0, 130.8, 131.0, 133.9, 134.0, 134.0, 135.1, 137.7, 137.9, 166.1, 198.5; IR ν_{max} (film): 3674.15, 3326.53, 2987.07, 2900.17, 1653.06, 1405.44, 1393.49, 1381.61, 1249.72, 1229.33, 1065.74, 1056.60, 1010.20, 891.69 cm^{-1}; HRMS m/z calcd for $C_{16}H_{11}F_3NO_5S$ [M-H]$^+$: 386.0310; found: 386.0321.

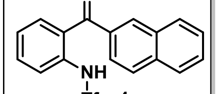
4r

***N*-(2-(2-naphthoyl)phenyl)-1,1,1-trifluoromethanesulfonamide (4r):** The product was obtained as a white solid. Yield: 77%; ^{1}H NMR (400 MHz, $CDCl_3$) δ 7.33 (t, *J* = 7.6 Hz, 1H), 7.61-7.71 (m, 3H), 7.75 (d, *J* = 8.0 Hz, 1H), 7.88 (t, *J* = 8.8 Hz, 2H), 7.96-8.02 (m, 3H), 8.23 (s, 1H), 10.91 (s, 1H); ^{13}C (100 MHz, $CDCl_3$) δ 119.9 (q, *J* = 294.2 Hz), 122.1, 124.9, 125.5, 125.9, 127.4, 128.0, 128.8, 129.1, 129.6, 132.2, 132.2, 133.9, 134.5, 134.7, 135.6, 137.5, 199.1; IR ν_{max} (film): 3674.30, 3338.78, 2987.14,

2900.17, 1655.10, 1405.77, 1393.47, 1381.47, 1249.69, 1229.00, 1065.77, 1056.65, 1010.20, 891.68 cm^{-1}; HRMS m/z calcd for $C_{18}H_{11}F_3NO_3S$ $[M-H]^+$: 378.0412; found: 378.0421.

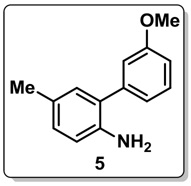

3'-methoxy-5-methyl-[1,1'-biphenyl]-2-amine (5): The product was obtained as a white solid. Yield: 82%; ^{1}H NMR (400 MHz, $CDCl_3$) δ 2.27 (s, 3H), 3.66 (s, 2H), 3.83 (s, 3H), 6.68 (d, *J* = 8.4 Hz, 1H), 6.88 (dd, *J* = 8.4 Hz, *J* = 1.6 Hz, 1H), 6.96-6.98 (m, 3H), 7.03 (d, *J* = 7.6 Hz, 1H), 7.34 (t, *J* = 8.0 Hz, 1H); ^{13}C (100 MHz, $CDCl_3$) δ 20.5, 55.4, 113.0, 114.6, 115.9, 121.5, 127.7, 127.9, 129.2, 129.9, 130.9, 141.1, 141.2, 160.0.

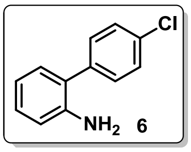

4'-chloro-[1,1'-biphenyl]-2-amine (6): The product was obtained as a white solid. Yield: 74%; ^{1}H NMR (400 MHz, $CDCl_3$) δ 3.65 (s, 2H), 6.69 (d, *J* = 7.6 Hz, 1H), 6.79 (t, *J* = 7.6 Hz, 1H), 7.05 (d, *J* = 7.6 Hz, 1H), 7.12 (t, *J* = 7.6 Hz, 1H), 7.33-7.38 (m, 4H); ^{13}C (100 MHz, $CDCl_3$) δ 115.8, 118.8, 126.3, 128.9, 129.0, 130.4, 130.5, 133.1, 138.0, 143.5.

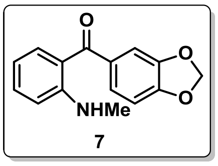

benzo[d][1,3]dioxol-5-yl(2-(methylamino)phenyl)methanone (7): The product was obtained as a light yellow solid. Yield: 56% (for three steps); ^{1}H NMR (400 MHz, $CDCl_3$) δ 2.94 (d, *J* = 4.0 Hz, 3H), 6.03 (s, 2H), 6.55 (t, *J* = 7.6 Hz, 1H), 6.74 (d, *J* = 8.0 Hz, 1H), 6.84 (d, *J* = 8.0 Hz, 1H), 7.16-7.26 (m, 2H), 7.39 (t, *J* = 8.0 Hz, 1H), 7.50 (d, *J* = 8.0 Hz, 1H), 8.18 (s, 1H); ^{13}C (100 MHz, $CDCl_3$) δ 29.6, 101.7, 107.7, 109.8, 111.2, 113.7, 117.7, 125.1, 134.6, 134.8, 135.0, 147.6, 150.2, 152.5, 197.7.

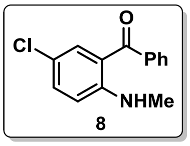

(5-chloro-2-(methylamino)phenyl)(phenyl)methanone (8): The product was obtained as a light yellow solid. Yield: 50% (for three steps); ^{1}H NMR (400 MHz, $CDCl_3$) δ 2.95 (d, *J* = 3.6 Hz, 3H), 6.71 (d, *J* = 9.2 Hz, 1H), 7.34 (dd, *J* = 9.2 Hz, *J* = 2.4 Hz, 1H), 7.43-7.49 (m, 3H), 7.52-7.60 (m, 3H), 8.47 (s, 1H); ^{13}C (100 MHz, $CDCl_3$) δ 29.7, 112.9, 118.0, 118.4, 128.4, 129.1, 131.3, 134.2, 135.0, 139.9, 151.3, 198.5.

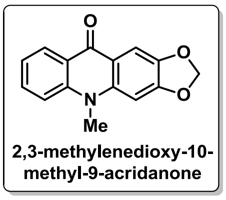

2,3-methylenedioxy-10-methyl-9-acridanone: The product was obtained as a white solid. Yield: 76%; ^{1}H NMR (400 MHz, $CDCl_3$) δ 3.84 (s, 3H), 6.08 (s, 2H), 6.94 (s, 1H), 7.28 (t, *J* = 7.6 Hz, 1H), 7.48 (d, *J* = 8.8 Hz, 1H), 7.68 (t, *J* = 7.6 Hz, 1H), 7.90 (s, 1H), 8.54 (d, *J* = 8.0 Hz, 1H); ^{13}C (100 MHz, $CDCl_3$) δ 34.5, 94.7, 102.2, 104.8, 114.7, 117.9, 121.4, 122.2, 127.8, 133.1, 140.6, 142.1, 143.8, 153.7, 176.5.

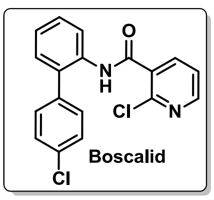

Boscalid: The product was obtained as a white solid. Yield: 77%; ^{1}H NMR

(400 MHz, $CDCl_3$) δ 7.27 (d, *J* = 4.8 Hz, 2H), 7.33-7.37 (m, 3H), 7.43-7.48 (m, 3H), 8.15 (d, *J* = 6.0 Hz, 2H), 8.42 (d, *J* = 8.0 Hz, 1H), 8.44 (dd, *J* = 4.8 Hz, *J* = 1.6 Hz, 1H); ^{13}C (100 MHz, $CDCl_3$) δ 122.2, 123.1, 125.5, 129.0, 129.5, 130.4, 130.9, 131.2, 132.4, 134.5, 134.6, 136.4, 140.3, 146.8, 151.5, 162.6.

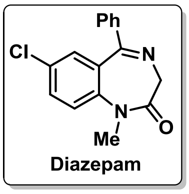

Diazepam: The product was obtained as a white solid. Yield: 48%; ^{1}H NMR (400 MHz, $CDCl_3$) δ 3.40 (s, 3H), 3.78 (d, *J* = 10.8 Hz, 1H), 4.84 (d, *J* = 10.8 Hz, 1H), 7.29 (s, 1H), 7.30 (d, *J* = 5.6 Hz, 1H), 7.40-7.44 (m, 2H), 7.47 (d, *J* = 7.2 Hz, 1H), 7.52 (dd, *J* = 8.8 Hz, *J* = 2.4 Hz, 1H), 7.59-7.61 (m, 2H); ^{13}C (100 MHz, $CDCl_3$) δ 35.0, 57.1, 122.7, 128.6, 129.4, 129.6, 130.1, 130.2, 130.9, 131.6, 138.3, 142.7, 169.1, 170.1.

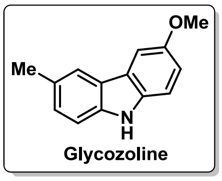

Glycozoline: The product was obtained as a white solid. Yield: 72%; ^{1}H NMR (400 MHz, $CDCl_3$) δ 2.52 (s, 3H), 3.92 (s, 3H), 7.04 (dd, *J* = 8.4 Hz, *J* = 2.4 Hz, 1H), 7.21 (d, *J* = 8.4 Hz, 1H), 7.29 (d, *J* = 8.0 Hz, 2H), 7.52 (d, *J* = 1.6 Hz, 1H), 7.80 (s, 1H), 7.83 (s, 1H); ^{13}C (100 MHz, $CDCl_3$) δ 21.6, 56.2, 103.3, 110.6, 111.4, 115.0, 120.3, 120.3, 123.7, 123.8, 127.3, 128.5, 134.9, 153.9.

7. NMR Spectra of 1-trifluoromethylsulfonyl Benzotriazoles

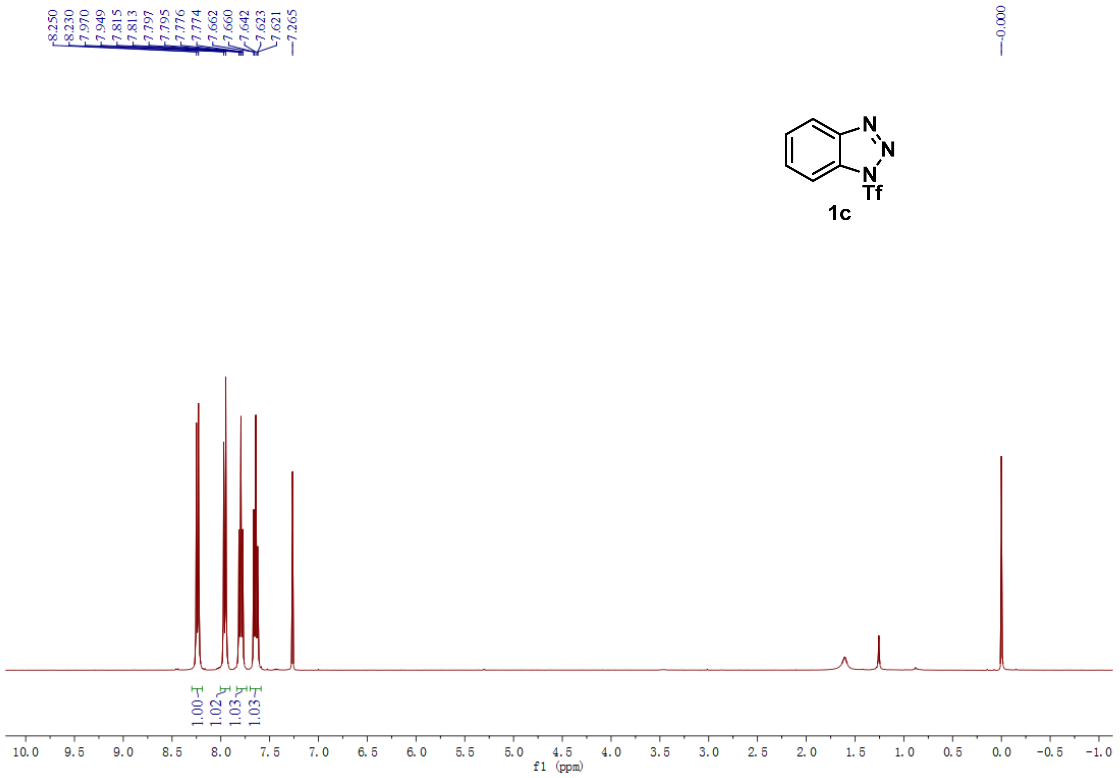

^{1}H NMR Spectrum for **1c** ($CDCl_3$, 400 MHz)

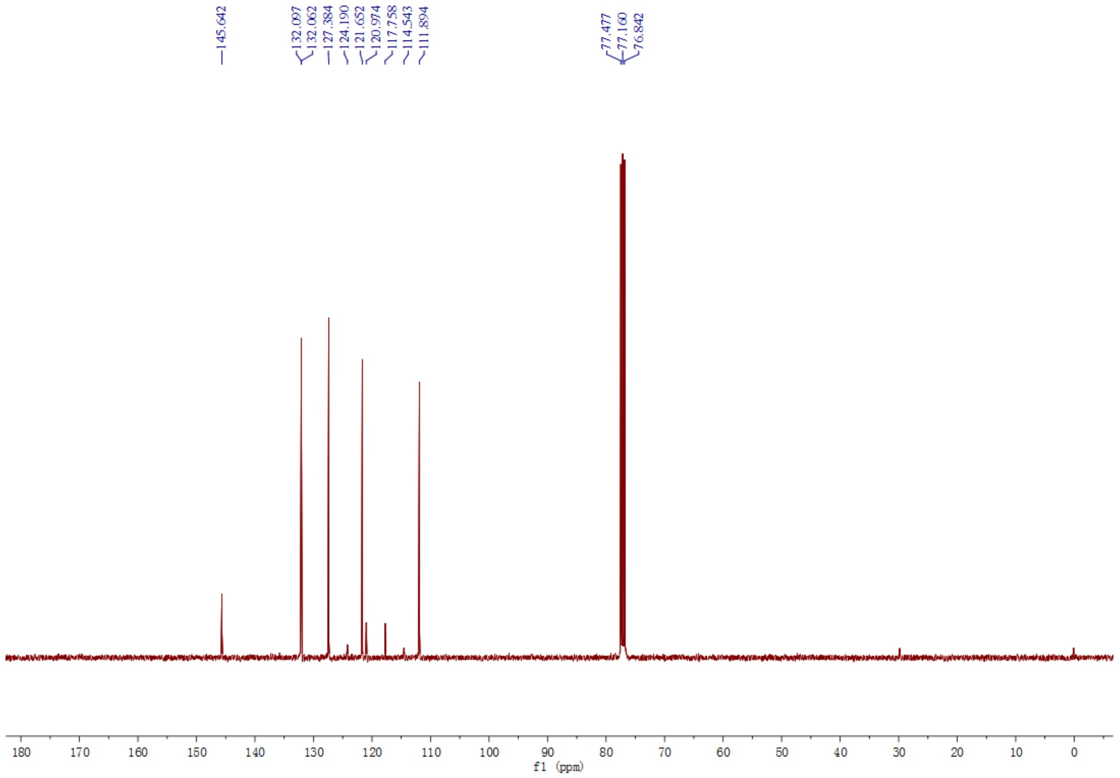

^{13}C NMR Spectrum for **1c** ($CDCl_3$, 100 MHz)

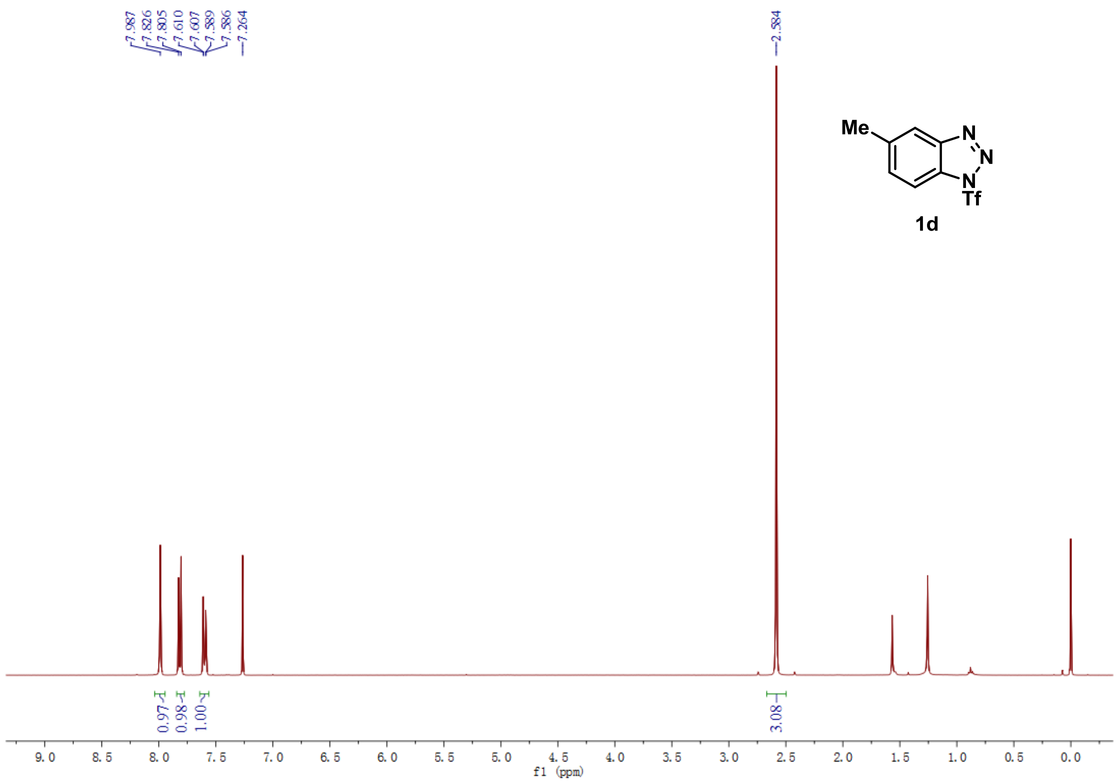

^{1}H NMR Spectrum for **1d** ($CDCl_3$, 400 MHz)

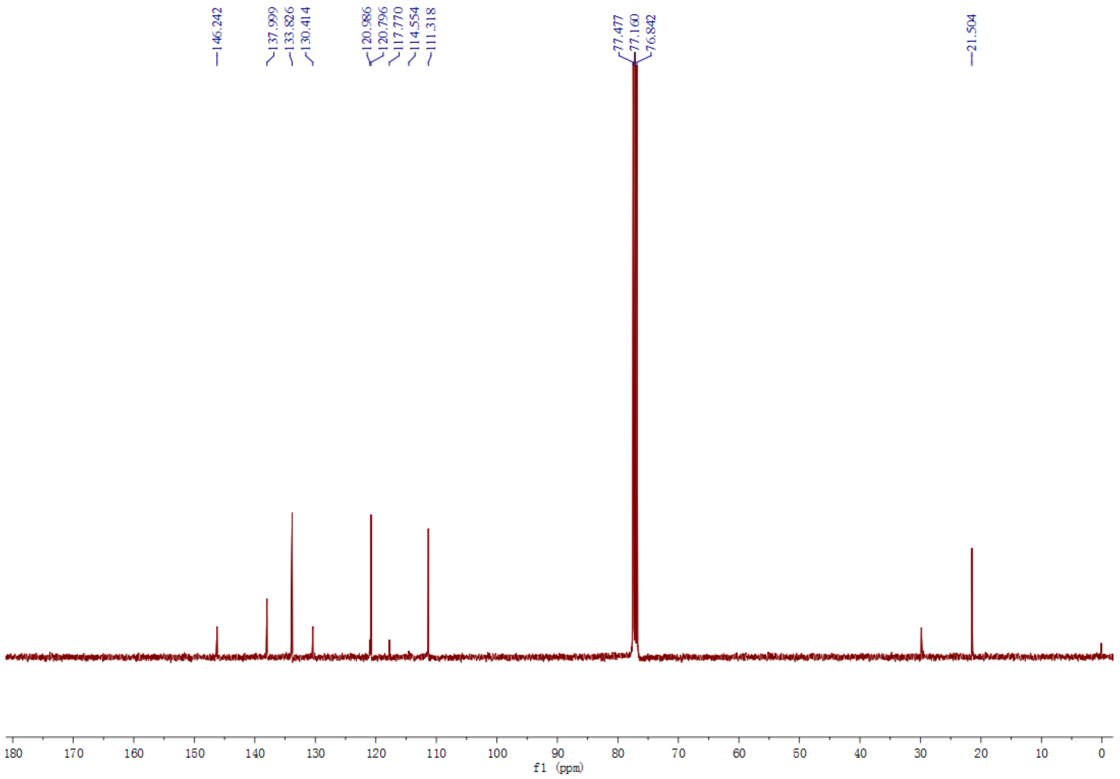

^{13}C NMR Spectrum for **1d** ($CDCl_3$, 100 MHz)

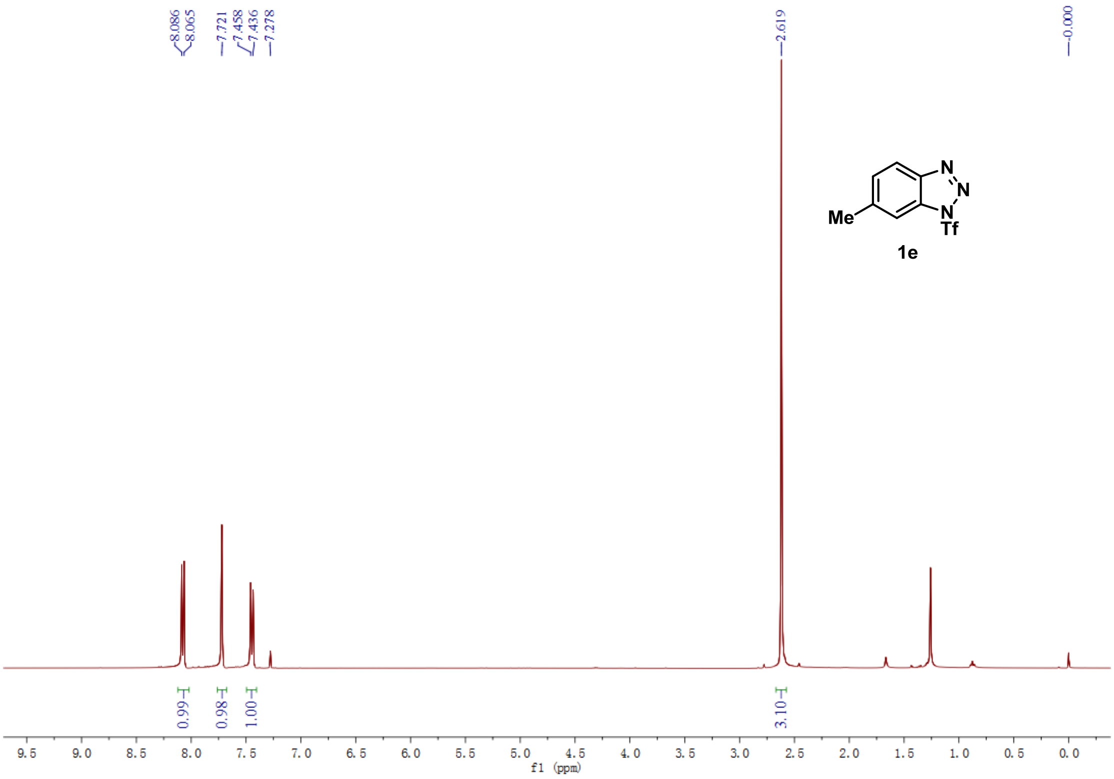

^{1}H NMR Spectrum for **1e** ($CDCl_3$, 400 MHz)

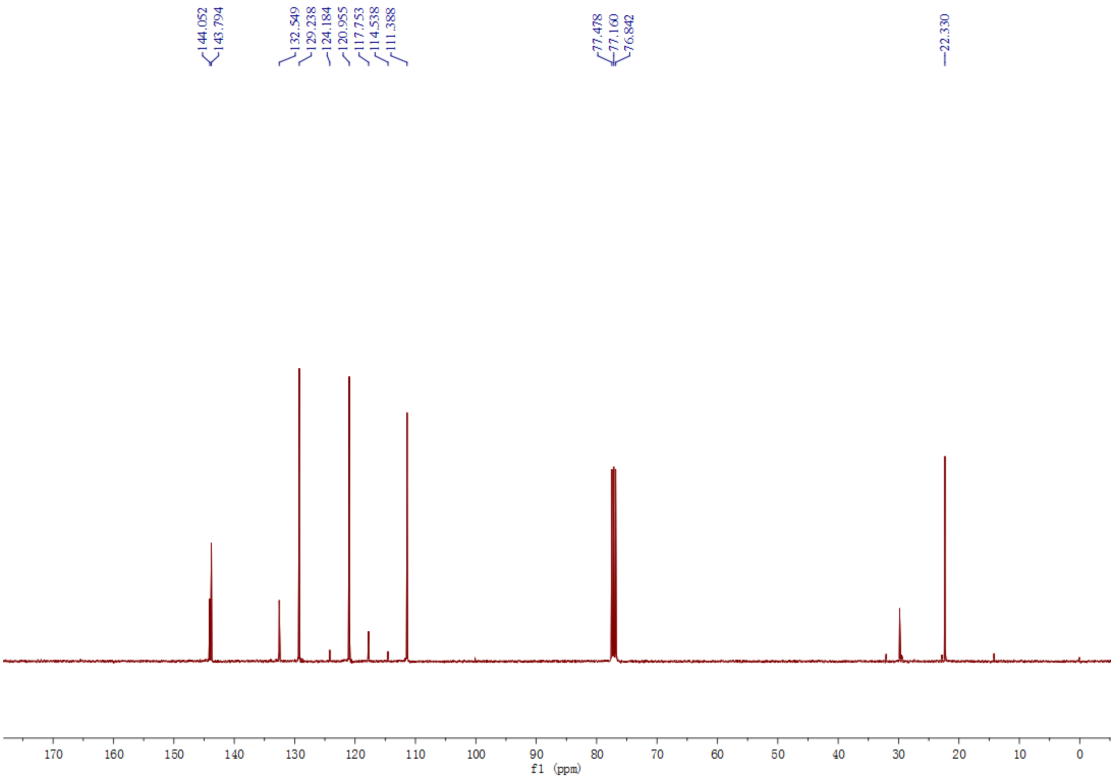

^{13}C NMR Spectrum for **1e** ($CDCl_3$, 100 MHz)

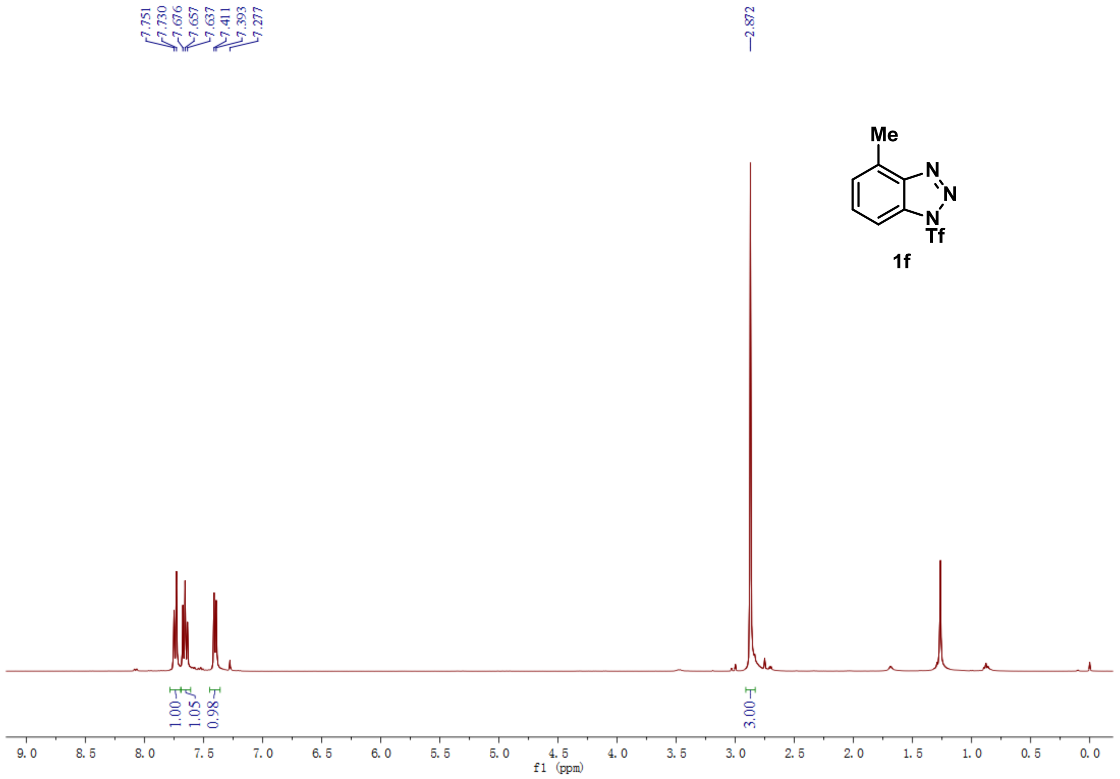

^{1}H NMR Spectrum for **1f** ($CDCl_3$, 400 MHz)

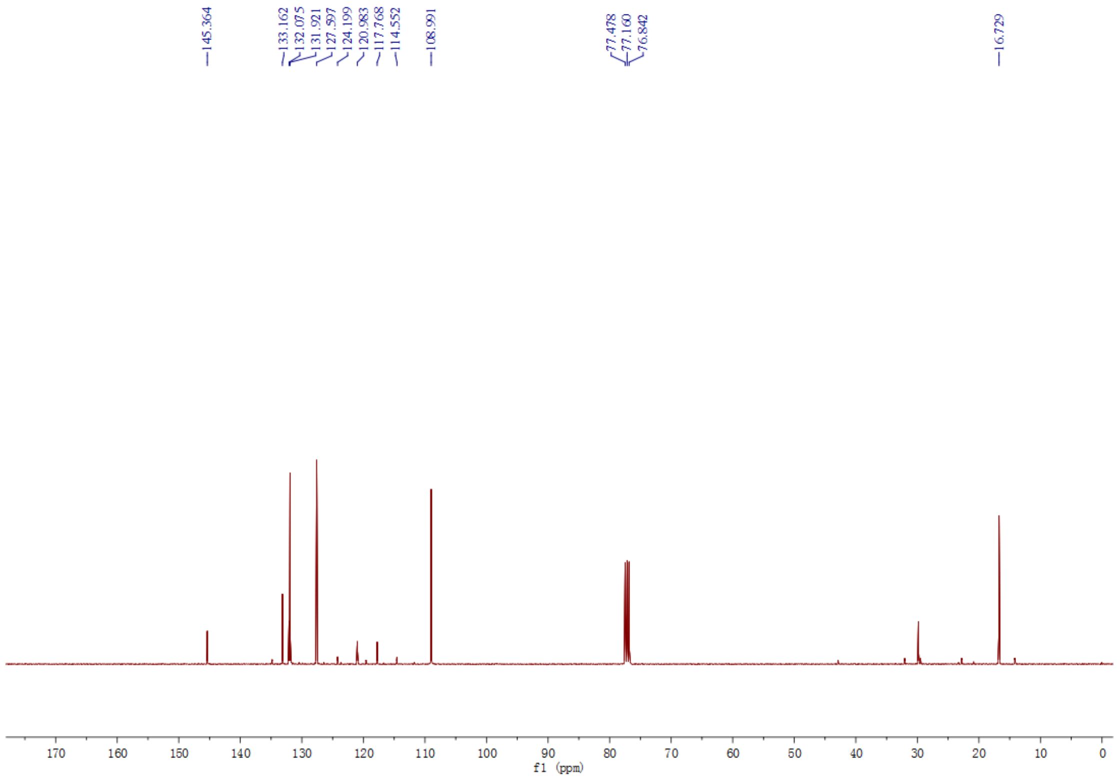

^{13}C NMR Spectrum for **1f** ($CDCl_3$, 100 MHz)

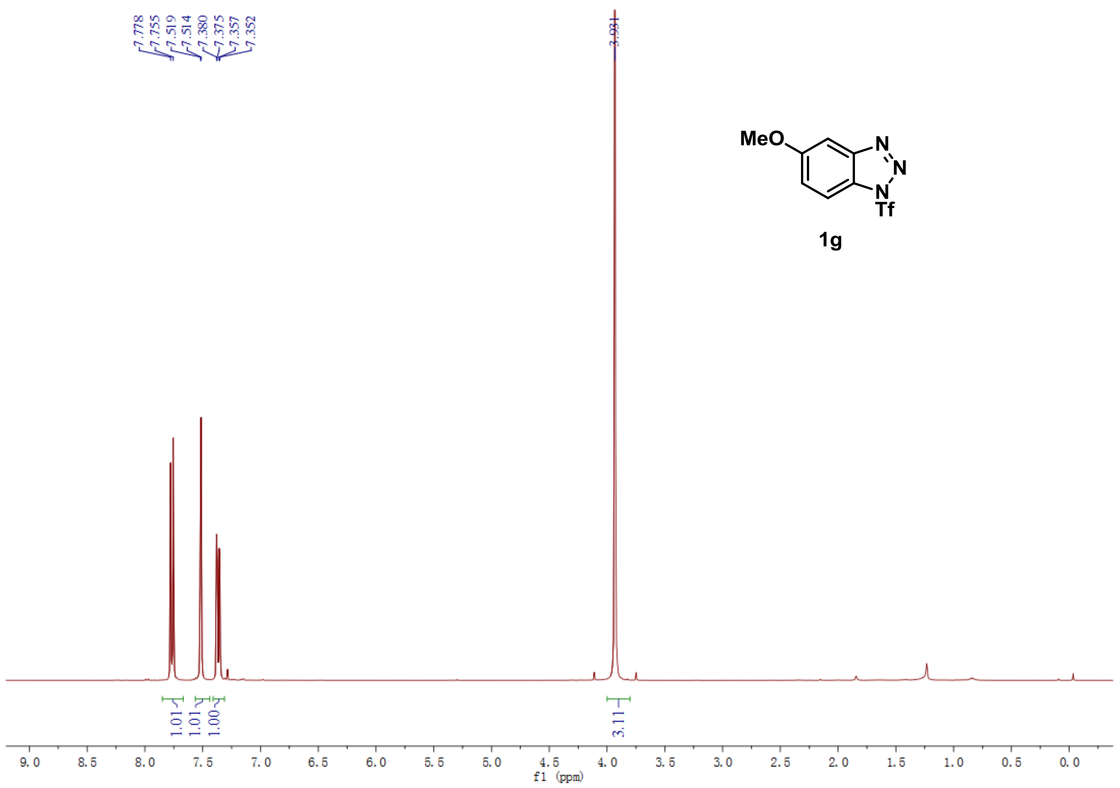

^{1}H NMR Spectrum for **1g** ($CDCl_3$, 400 MHz)

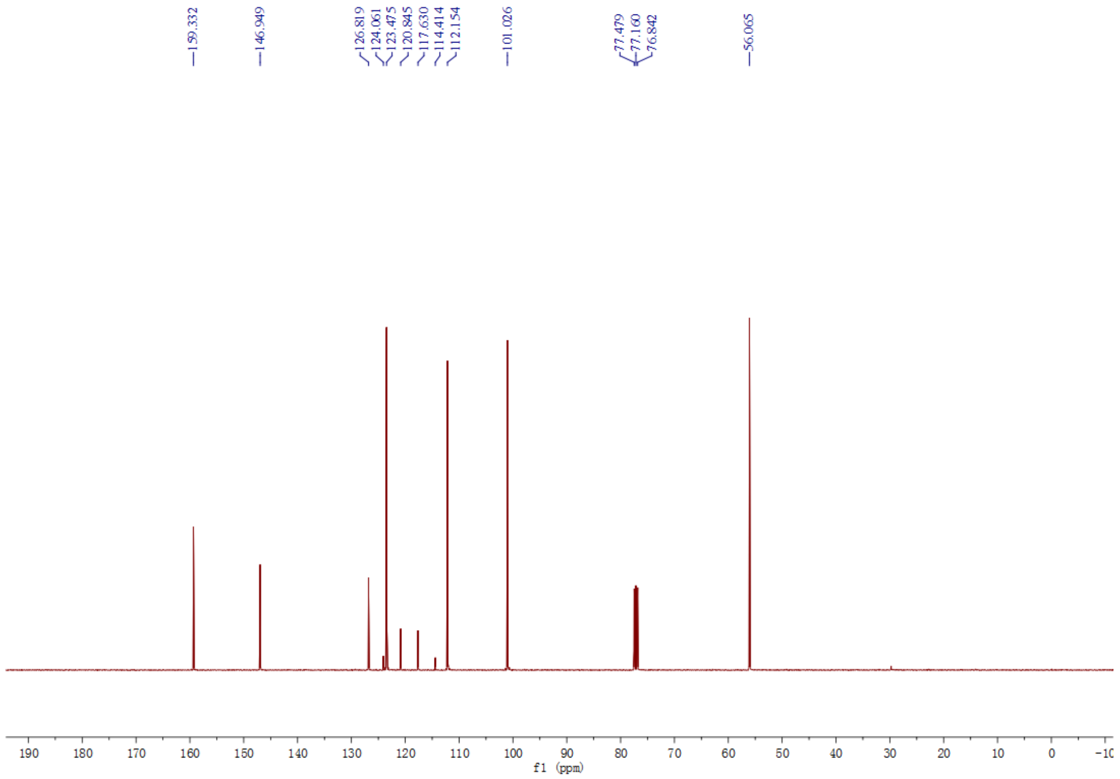

^{13}C NMR Spectrum for **1g** ($CDCl_3$, 100 MHz)

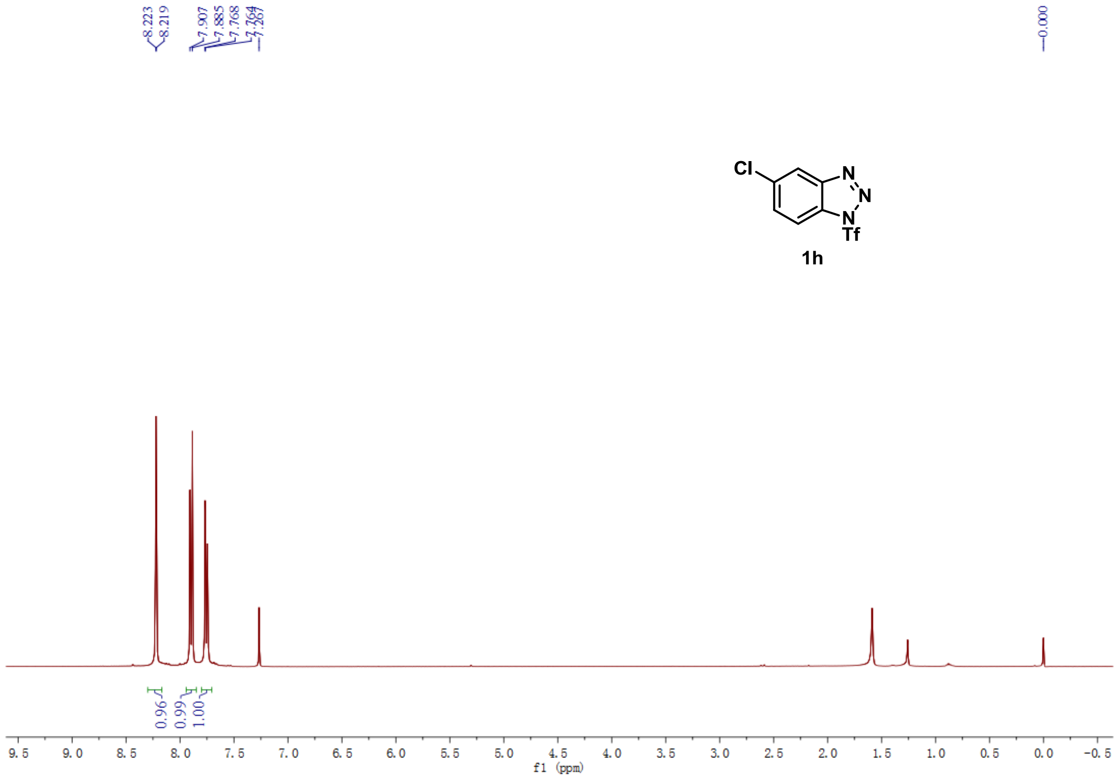

1h

^{1}H NMR Spectrum for **1h** ($CDCl_3$, 400 MHz)

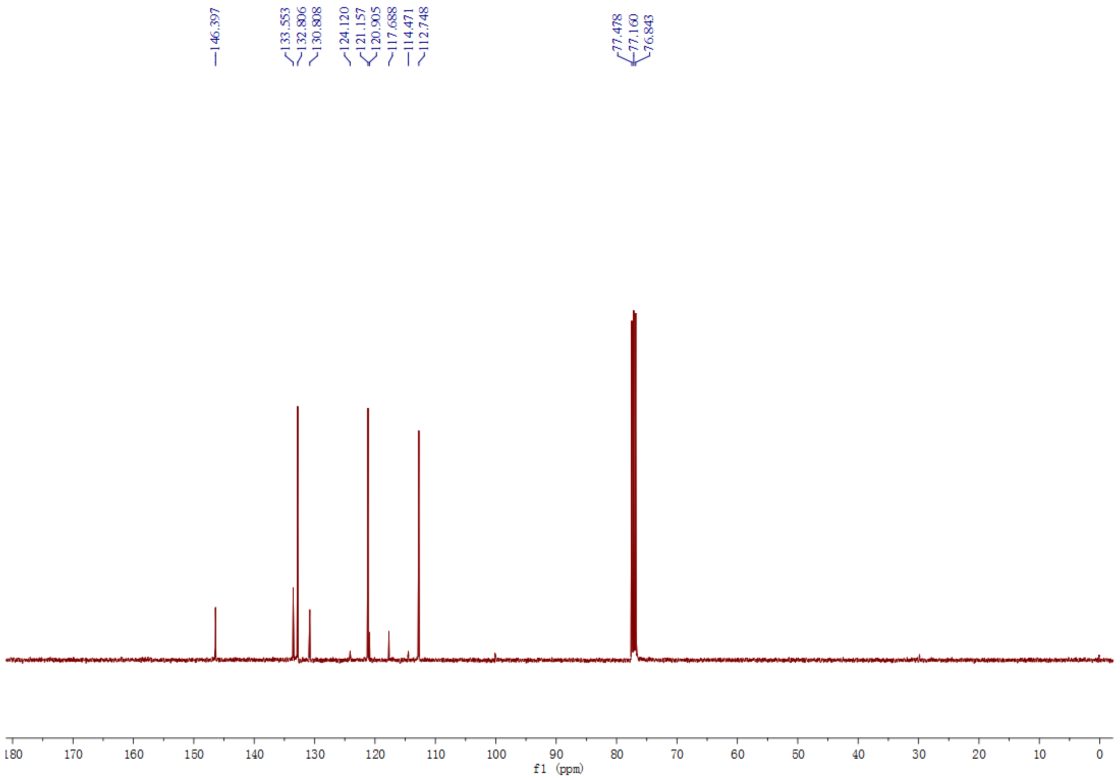

^{13}C NMR Spectrum for **1h** ($CDCl_3$, 100 MHz)

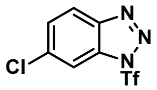

1i

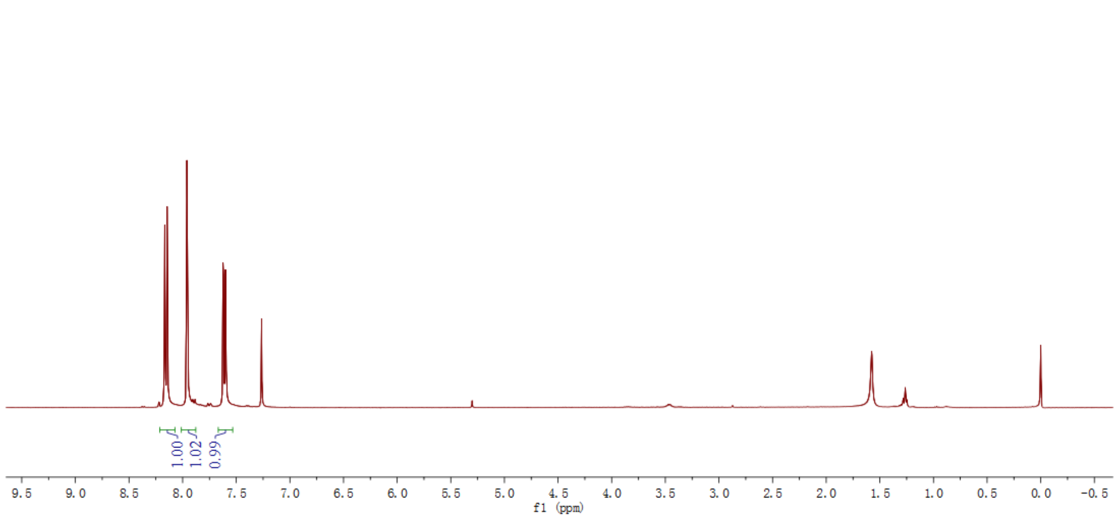

^{1}H NMR Spectrum for **1i** ($CDCl_3$, 400 MHz)

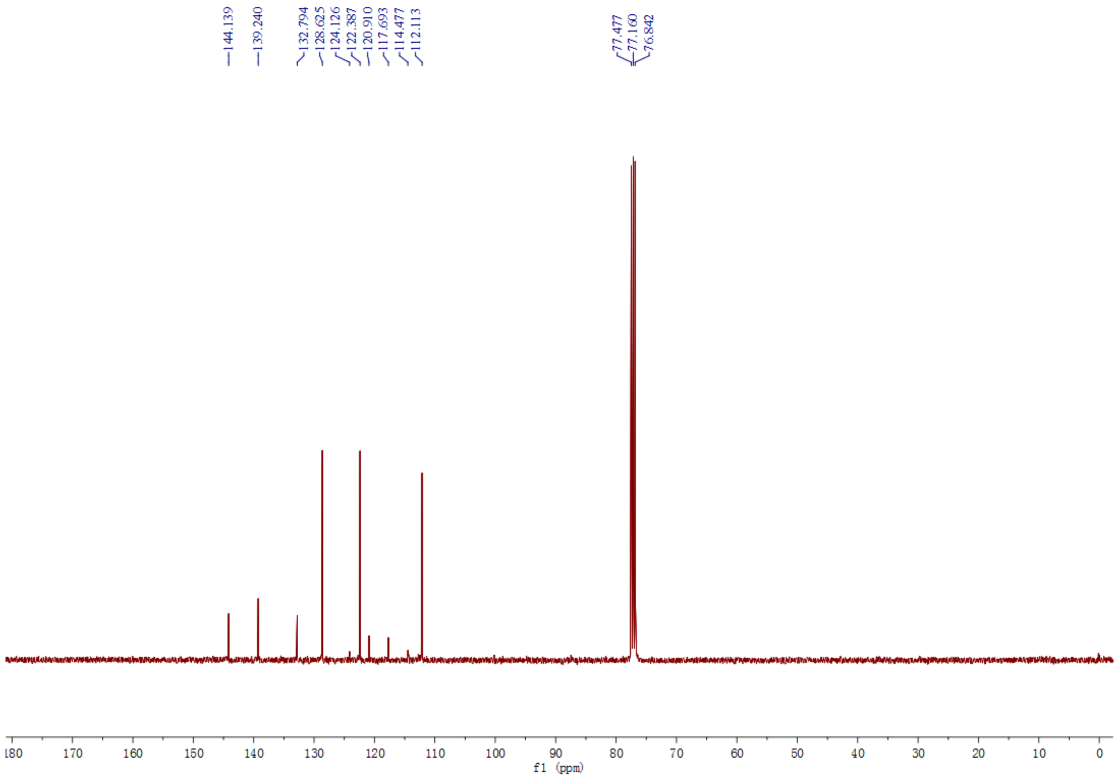

^{13}C NMR Spectrum for **1i** ($CDCl_3$, 100 MHz)

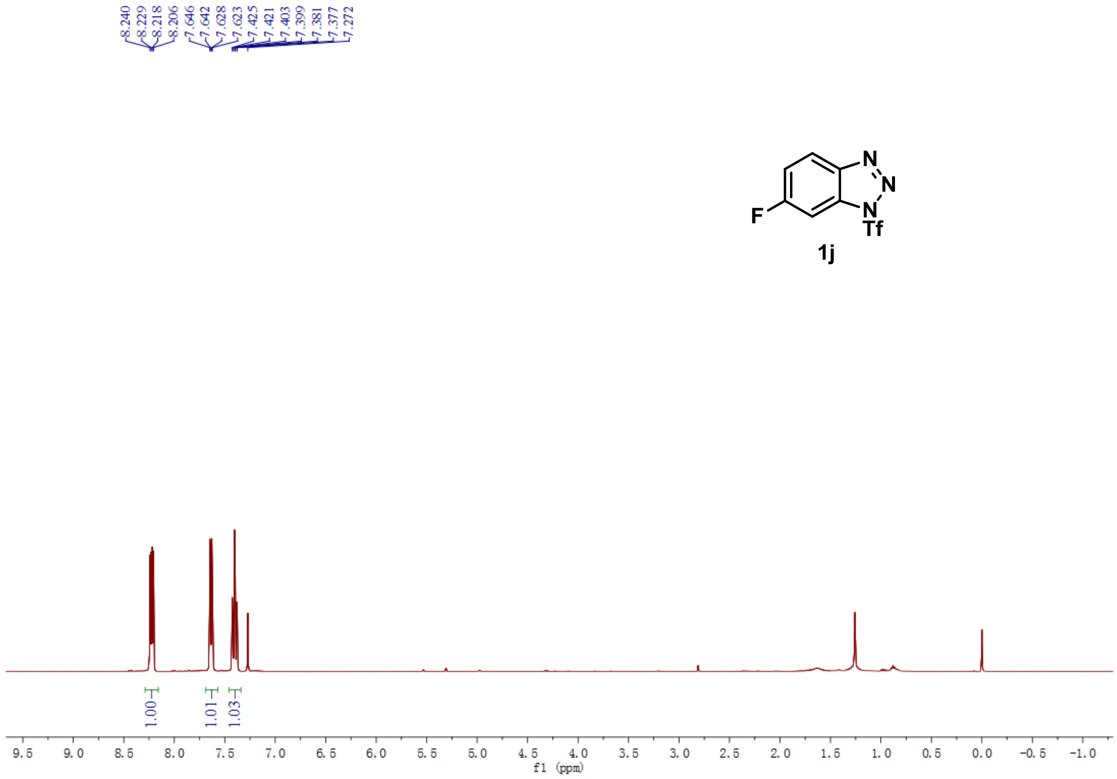

^{1}H NMR Spectrum for **1j** ($CDCl_3$, 400 MHz)

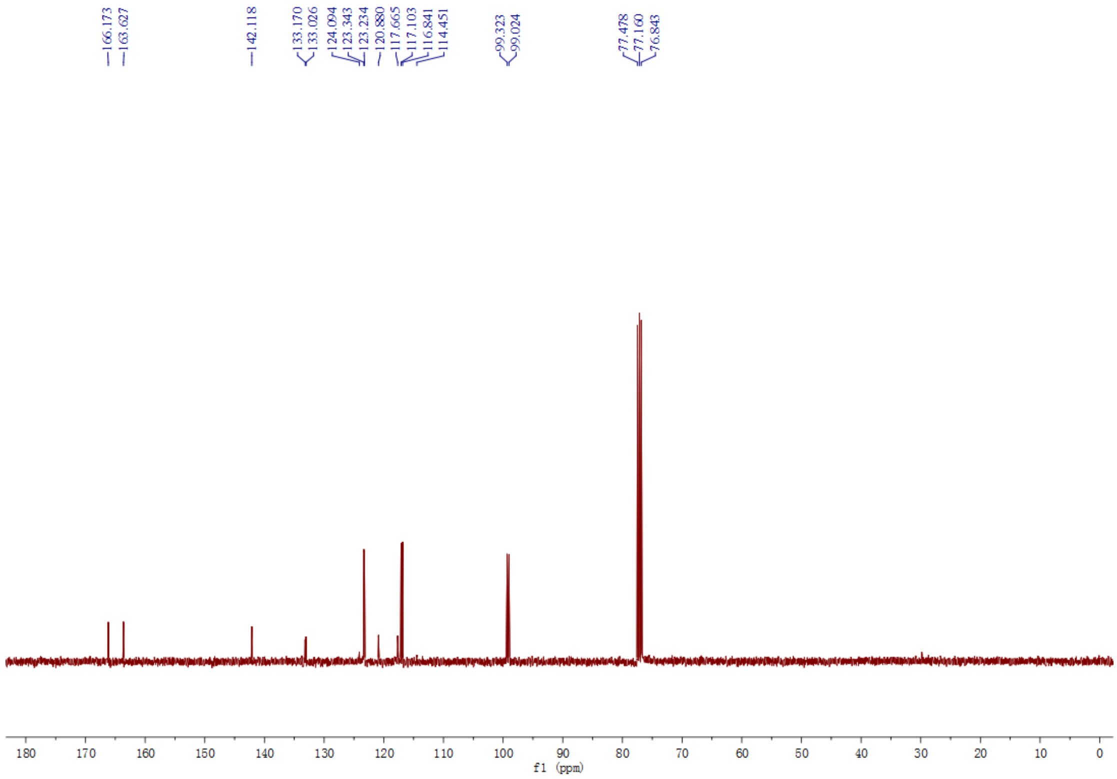

^{13}C NMR Spectrum for **1j** ($CDCl_3$, 100 MHz)

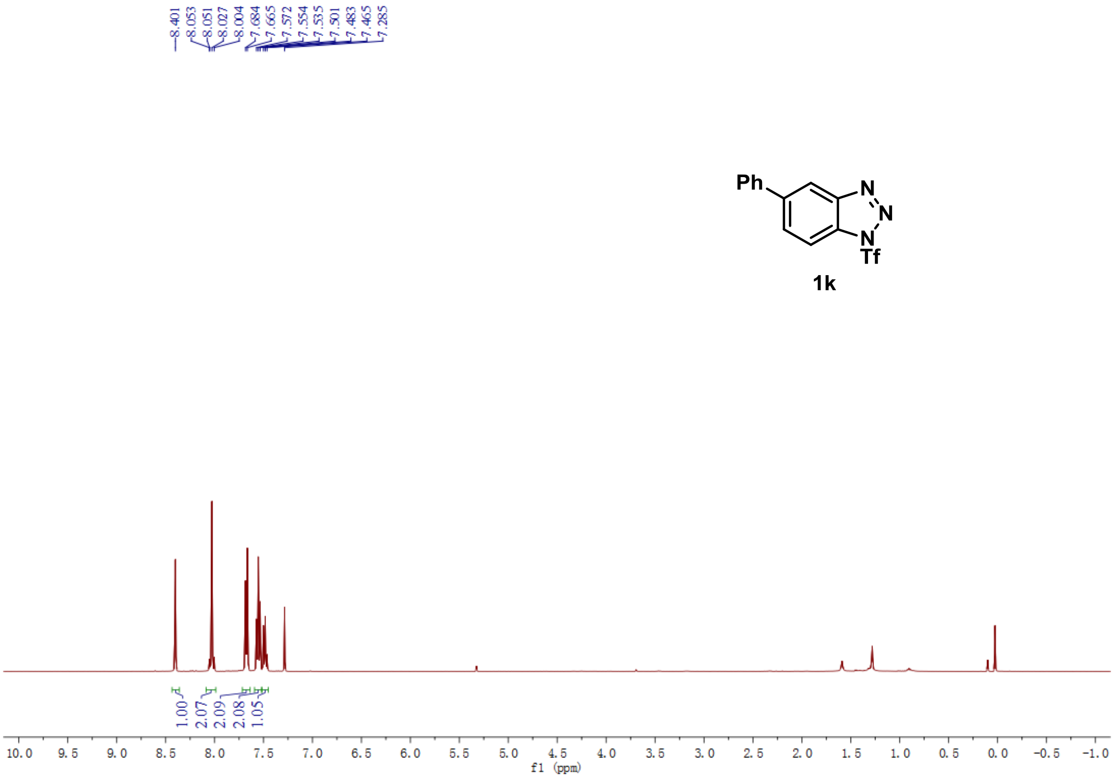

1k

^{1}H NMR Spectrum for **1k** ($CDCl_3$, 400 MHz)

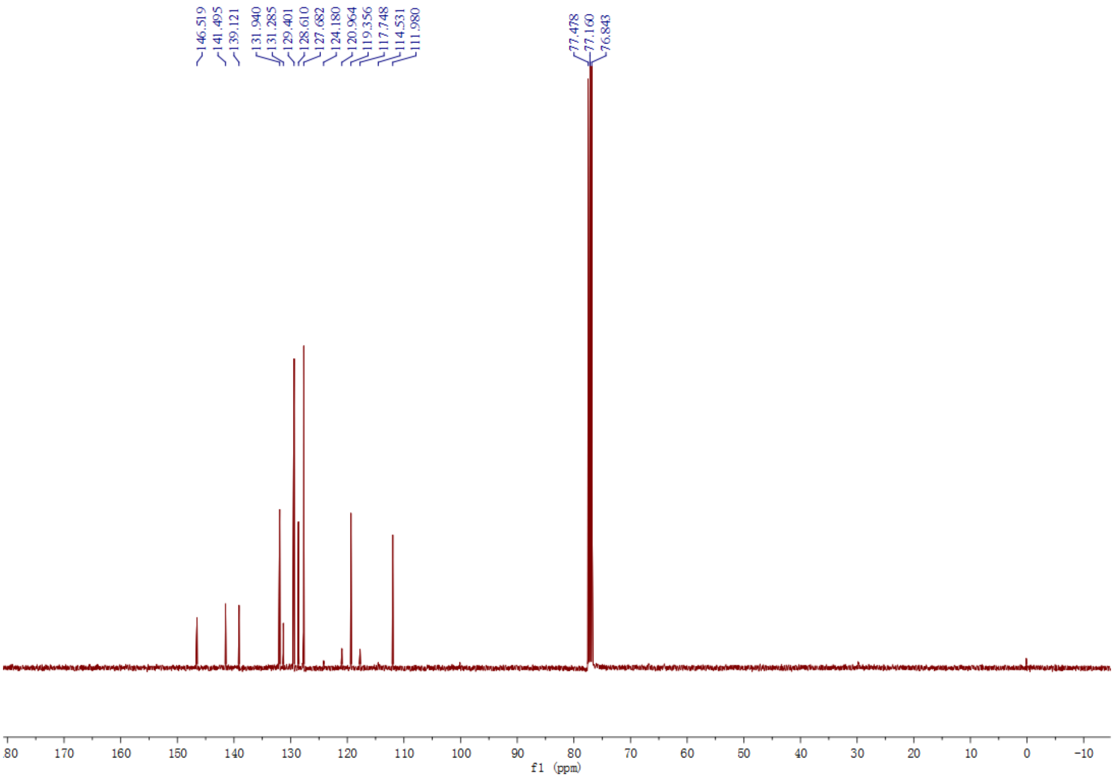

^{13}C NMR Spectrum for **1k** ($CDCl_3$, 100 MHz)

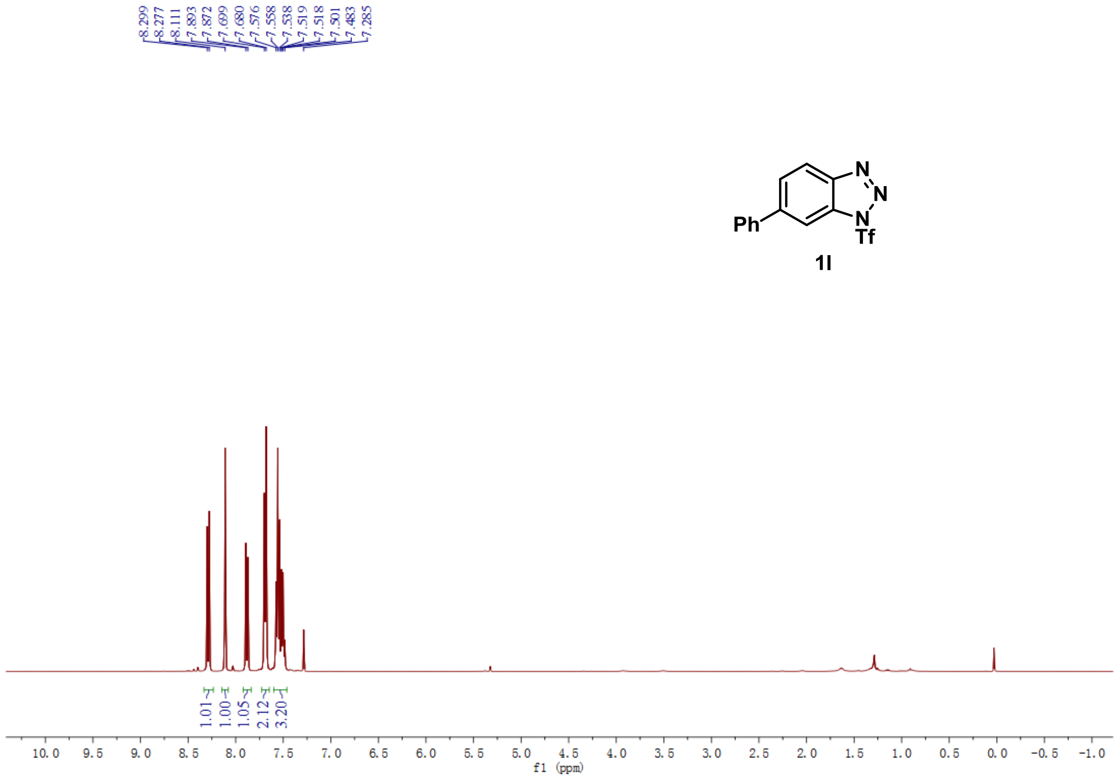

^{1}H NMR Spectrum for **1l** ($CDCl_3$, 400 MHz)

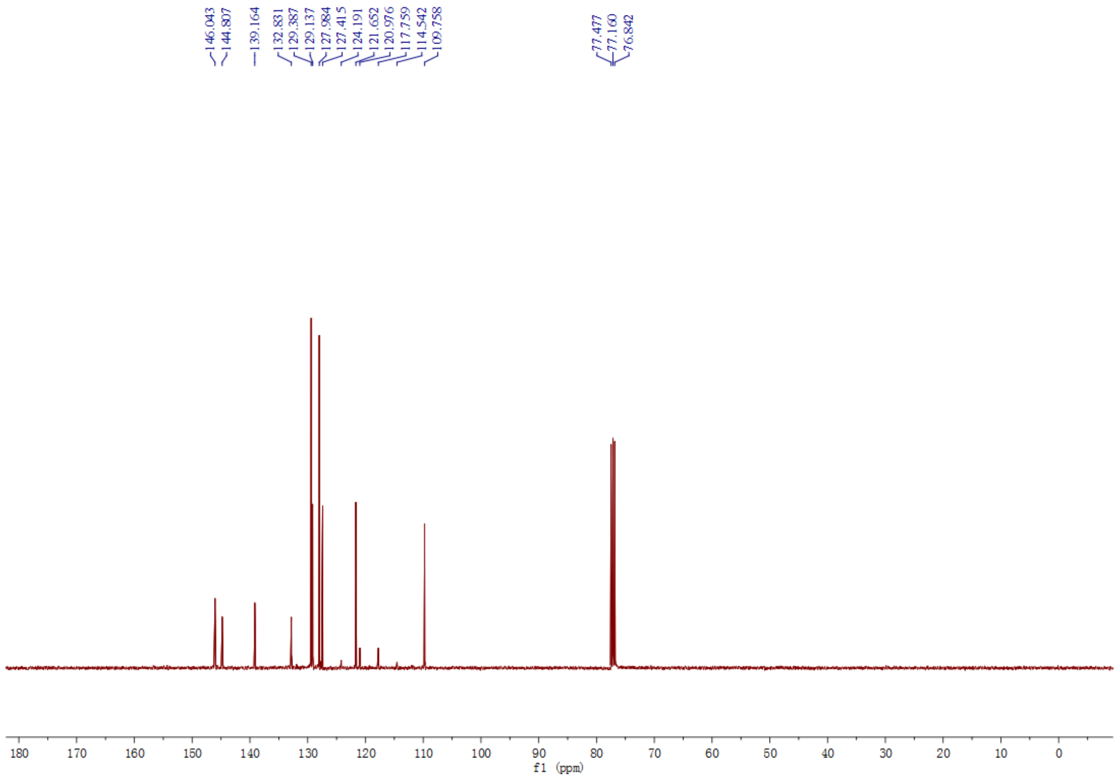

^{13}C NMR Spectrum for **1l** ($CDCl_3$, 100 MHz)

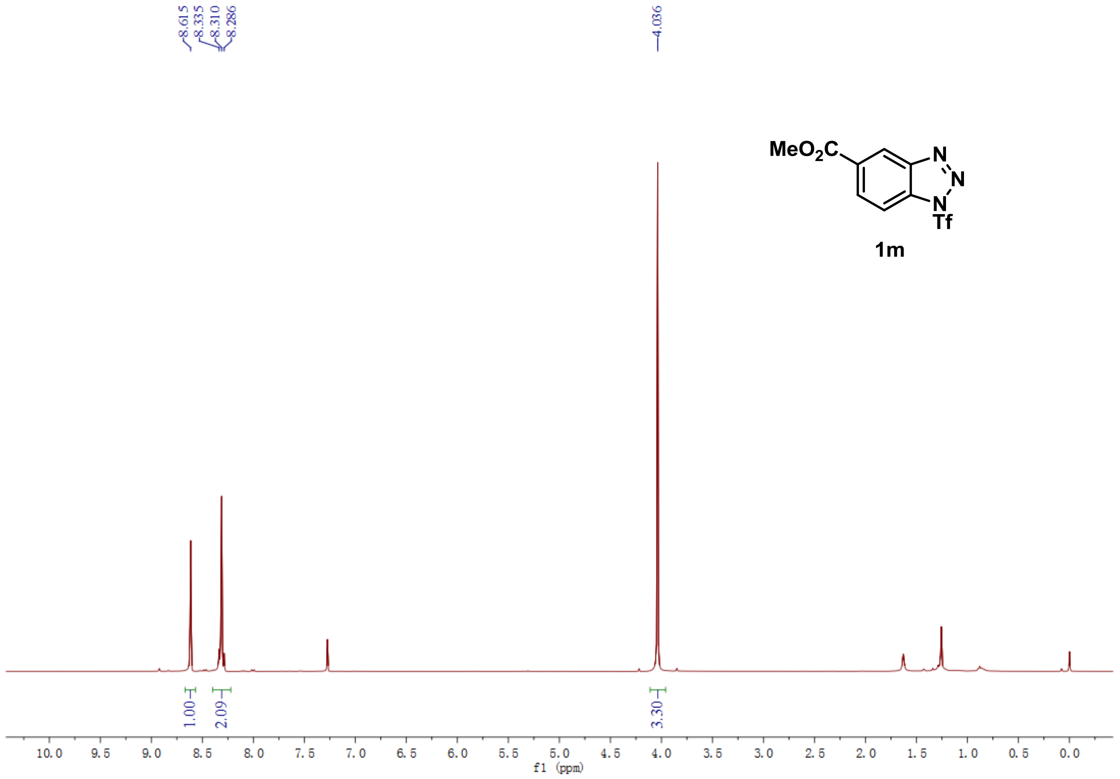

^{1}H NMR Spectrum for **1m** ($CDCl_3$, 400 MHz)

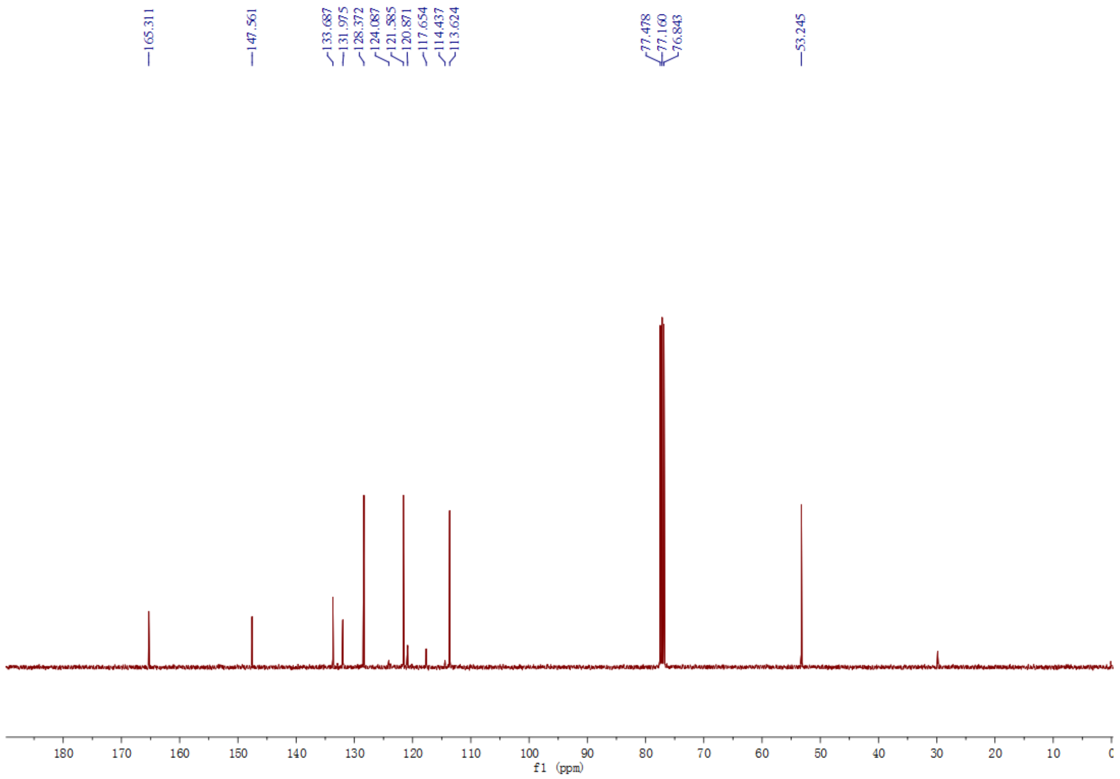

^{13}C NMR Spectrum for **1m** ($CDCl_3$, 100 MHz)

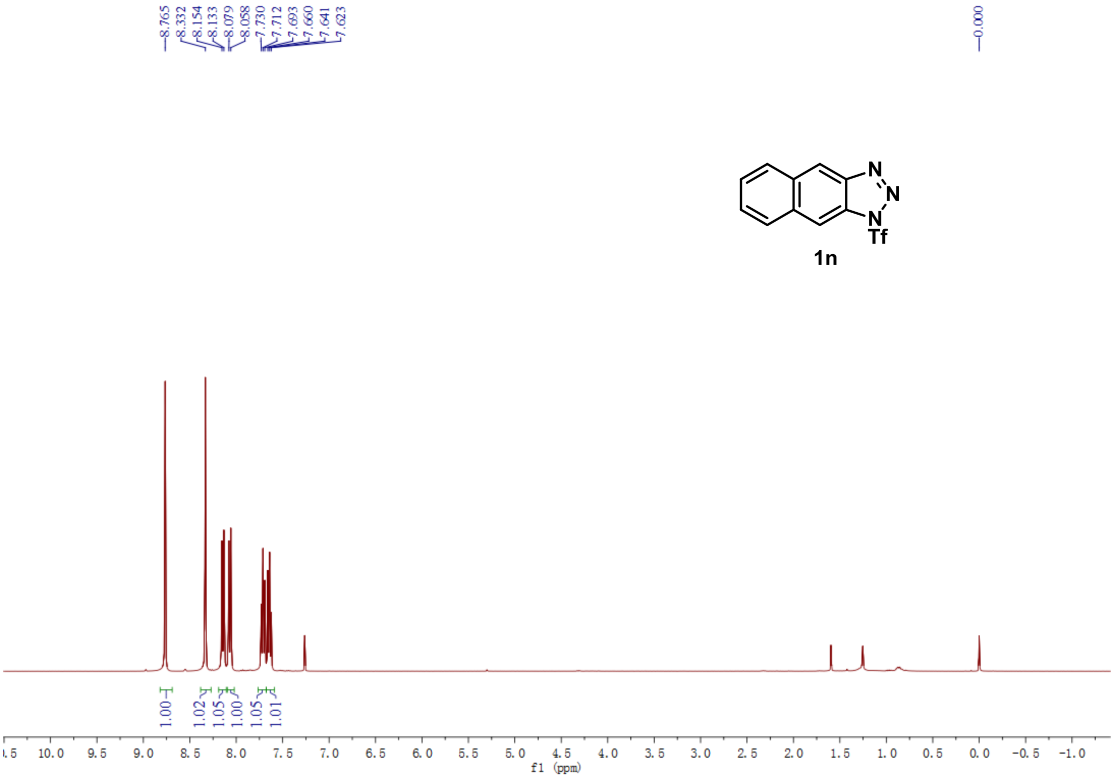

^{1}H NMR Spectrum for **1n** ($CDCl_3$, 400 MHz)

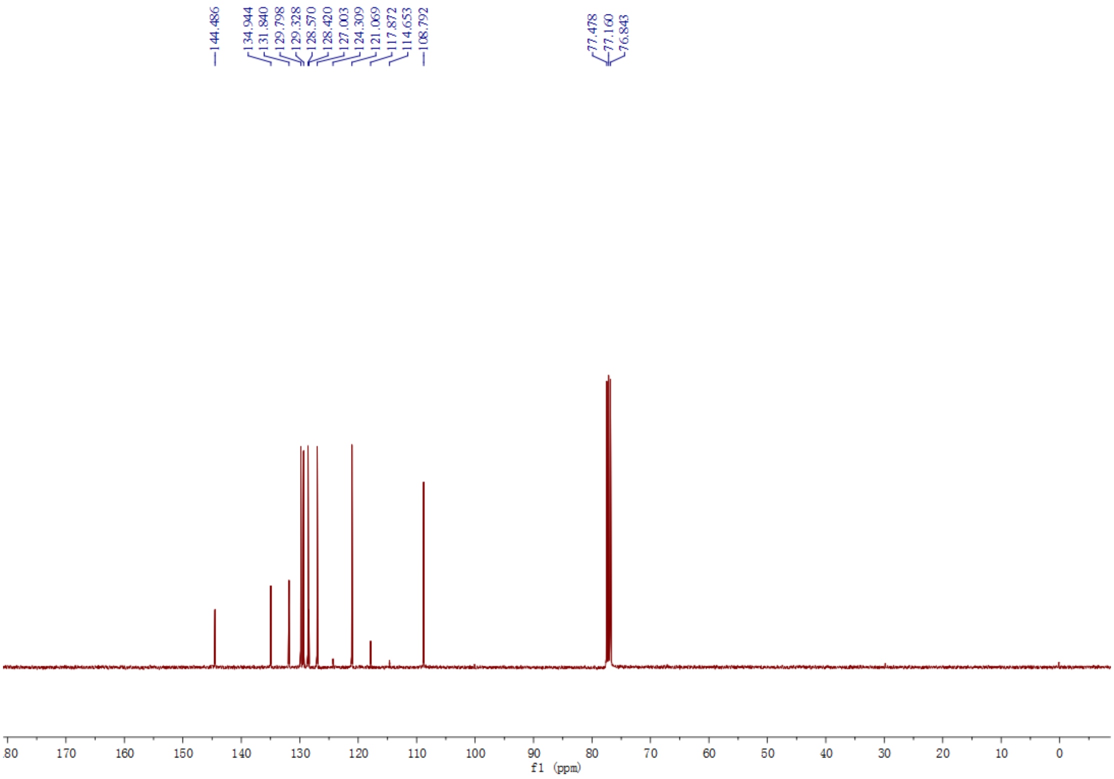

^{13}C NMR Spectrum for **1n** ($CDCl_3$, 100 MHz)

8. NMR Spectra of Suzuki and Carbonylative Suzuki Coupling Products

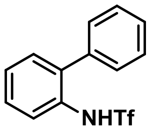

3a

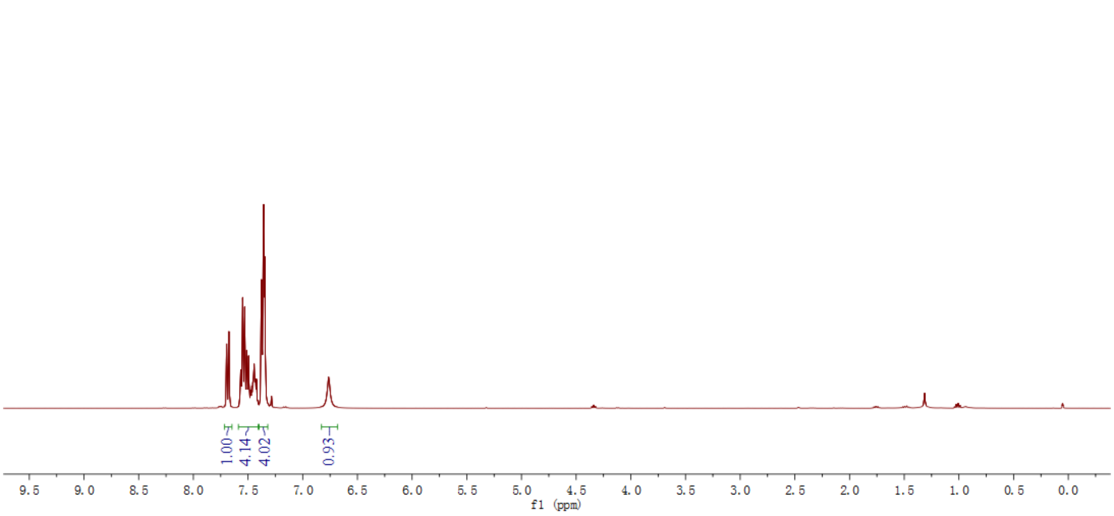

^{1}H NMR Spectrum for **3a** ($CDCl_3$, 400 MHz)

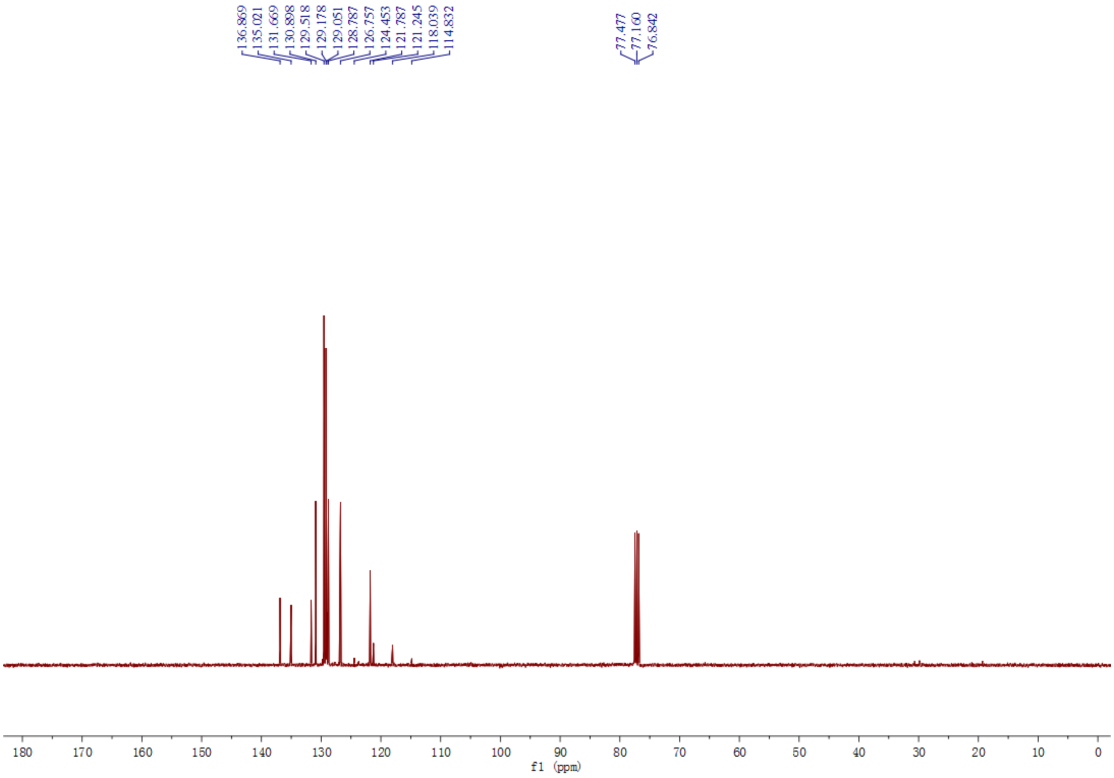

^{13}C NMR Spectrum for **3a** ($CDCl_3$, 100 MHz)

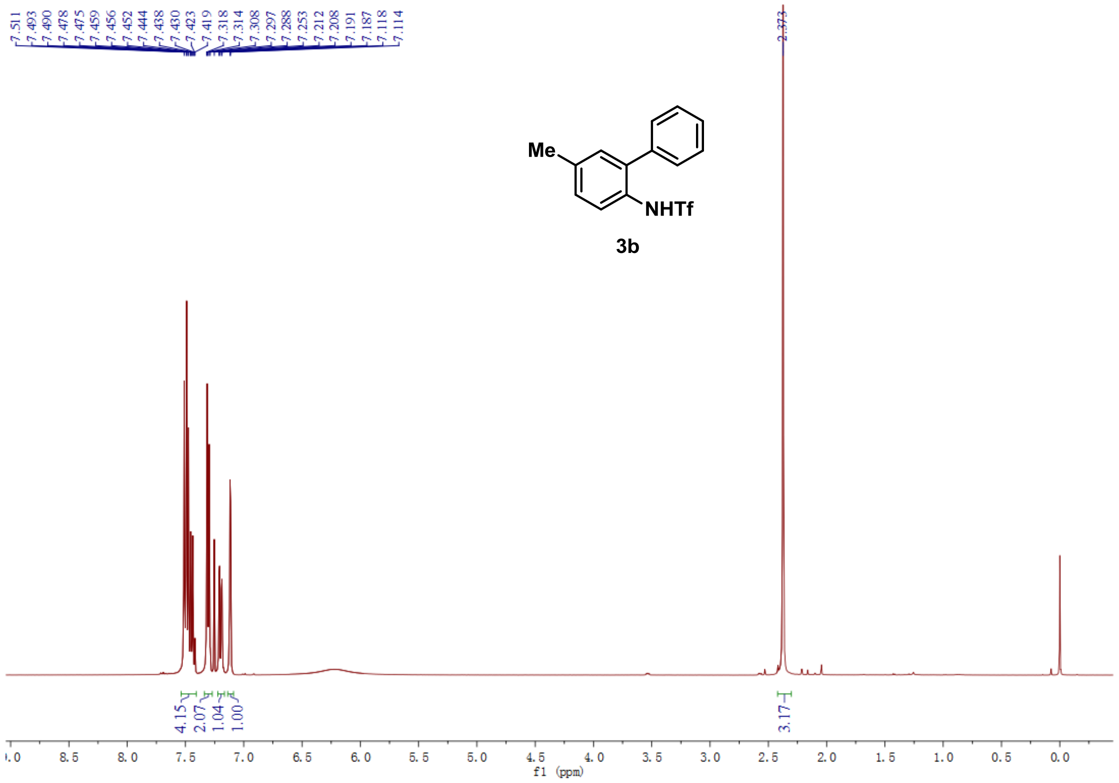

^{1}H NMR Spectrum for **3b** ($CDCl_3$, 400 MHz)

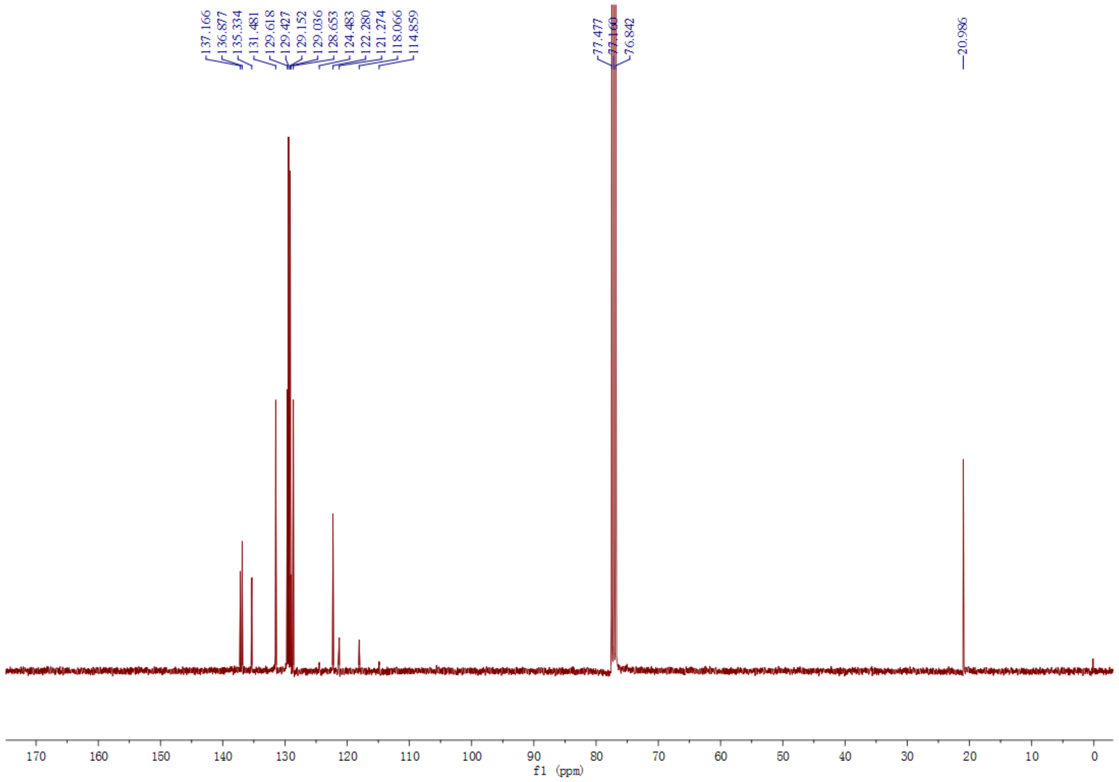

^{13}C NMR Spectrum for **3b** ($CDCl_3$, 100 MHz)

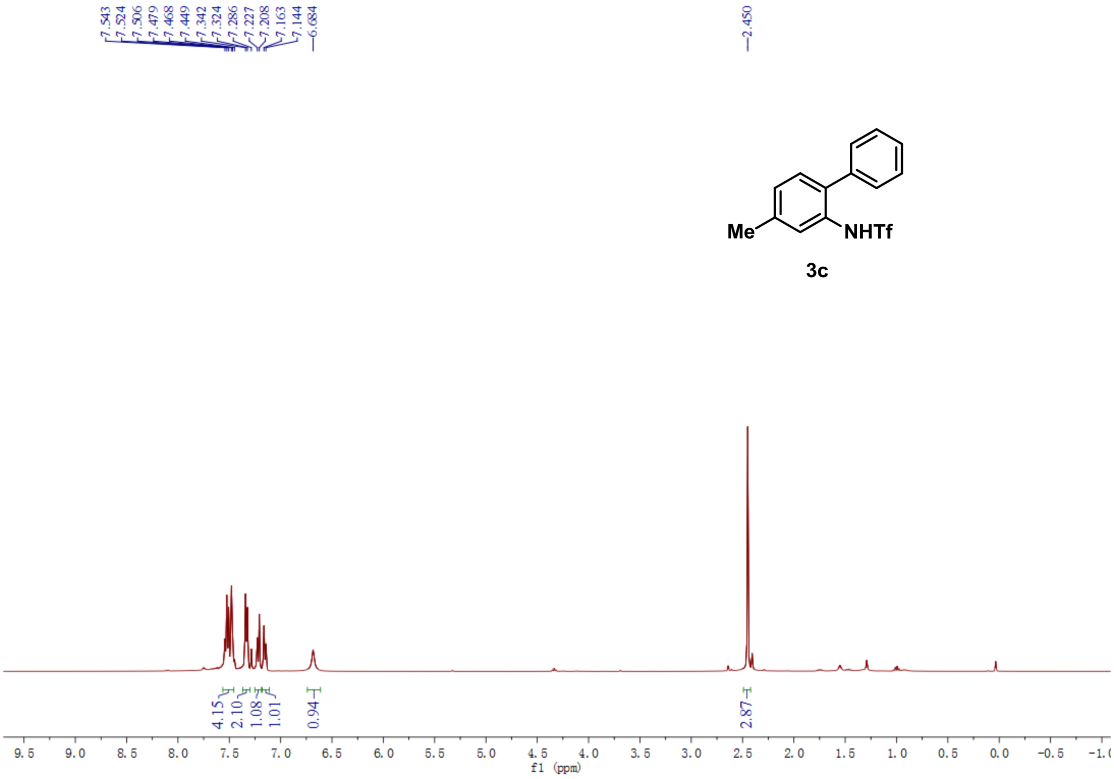

^{1}H NMR Spectrum for **3c** ($CDCl_3$, 400 MHz)

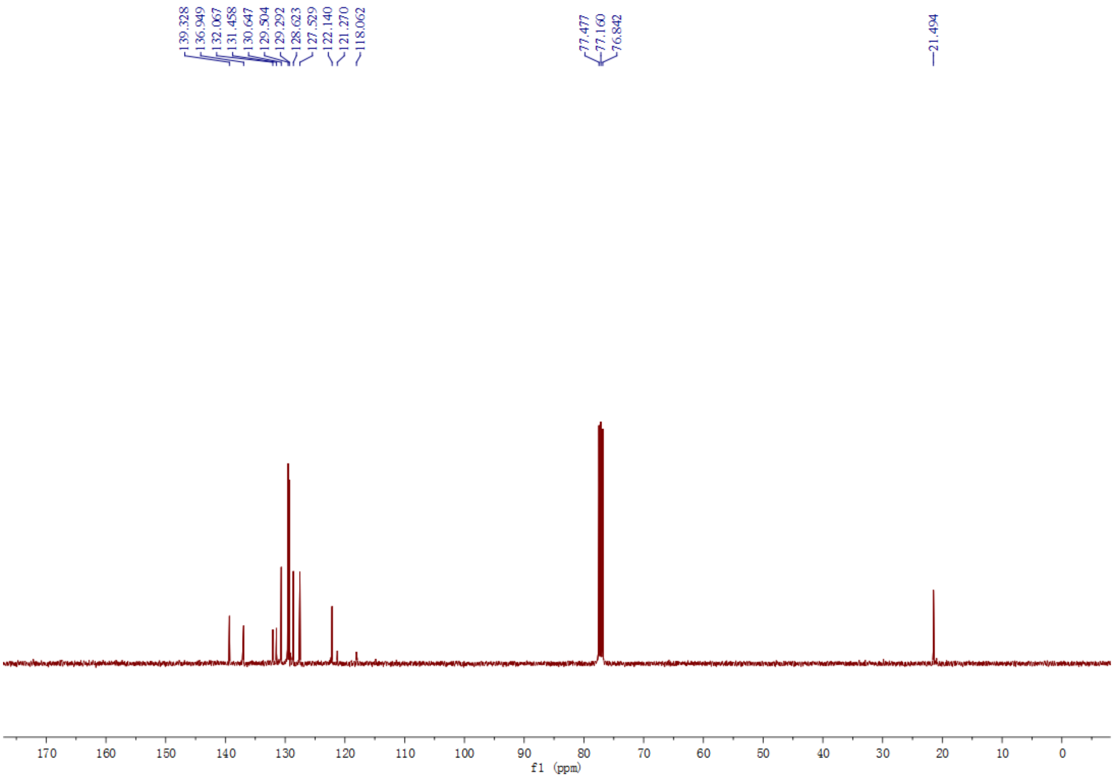

^{13}C NMR Spectrum for **3c** ($CDCl_3$, 100 MHz)

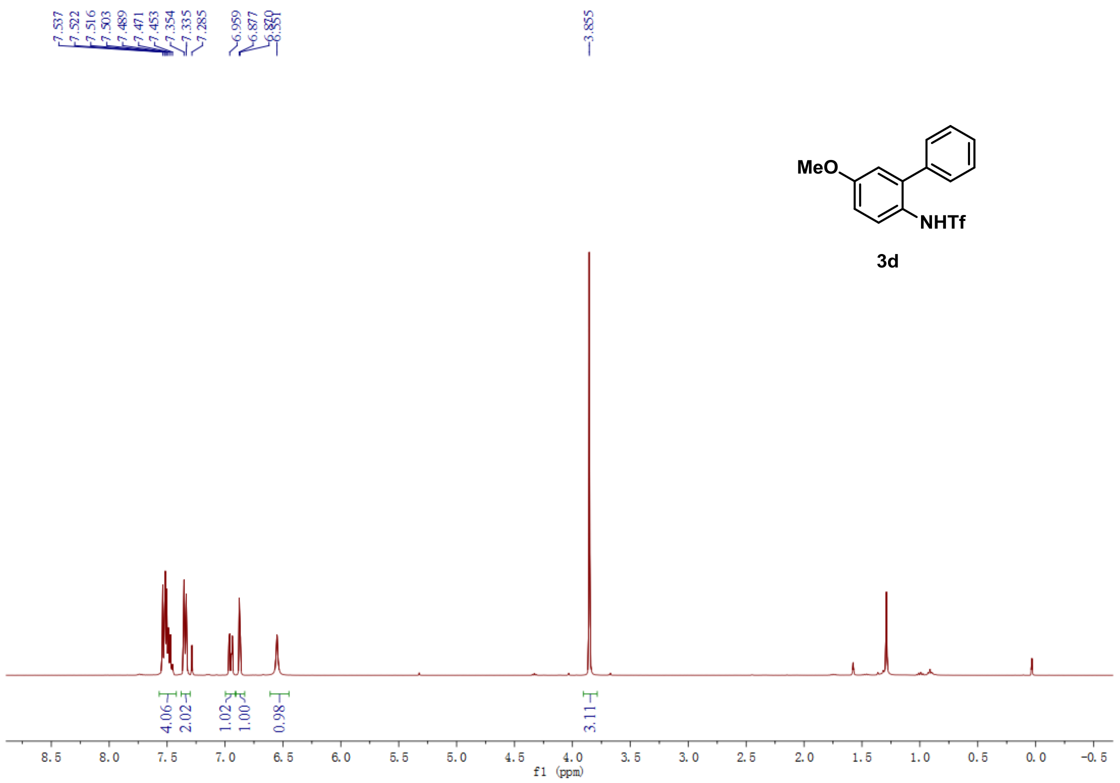

^{1}H NMR Spectrum for **3d** ($CDCl_3$, 400 MHz)

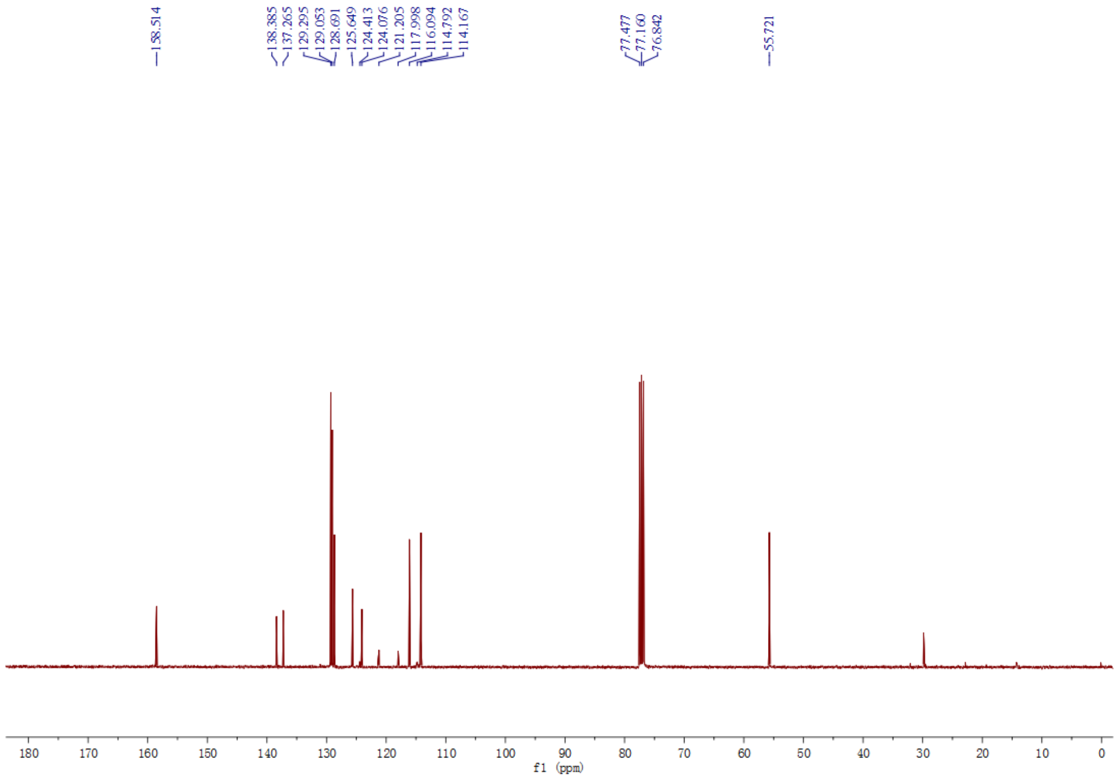

^{13}C NMR Spectrum for **3d** ($CDCl_3$, 100 MHz)

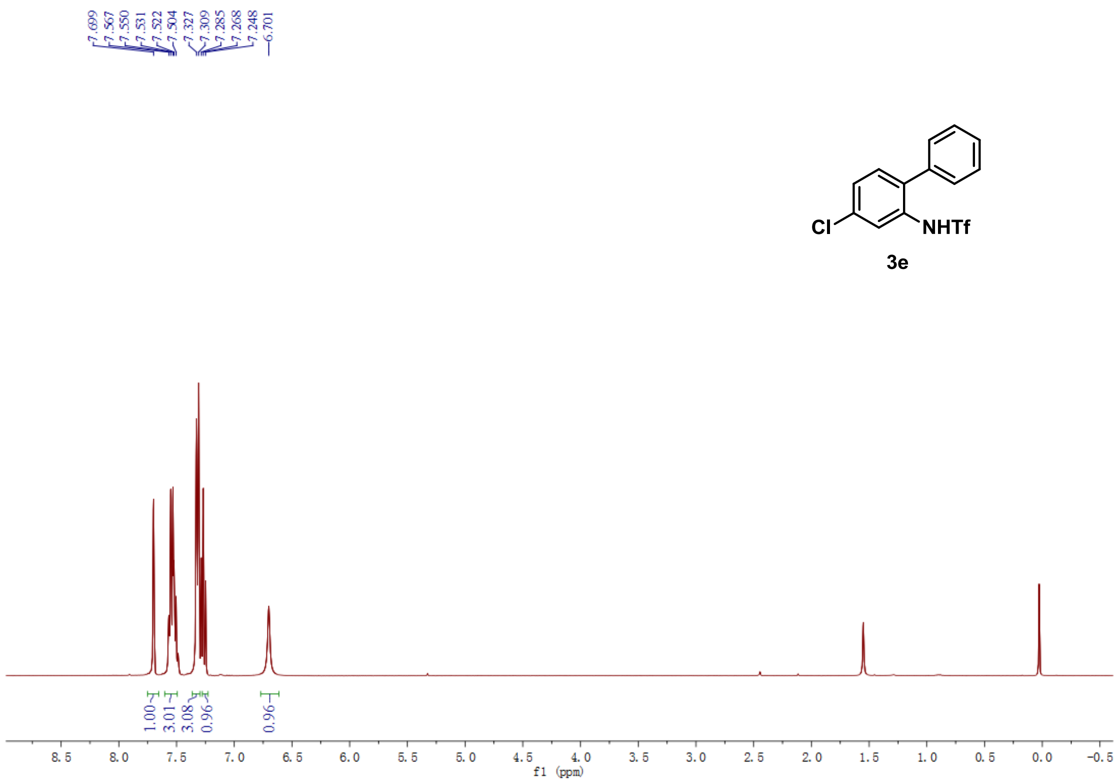

^{1}H NMR Spectrum for **3e** ($CDCl_3$, 400 MHz)

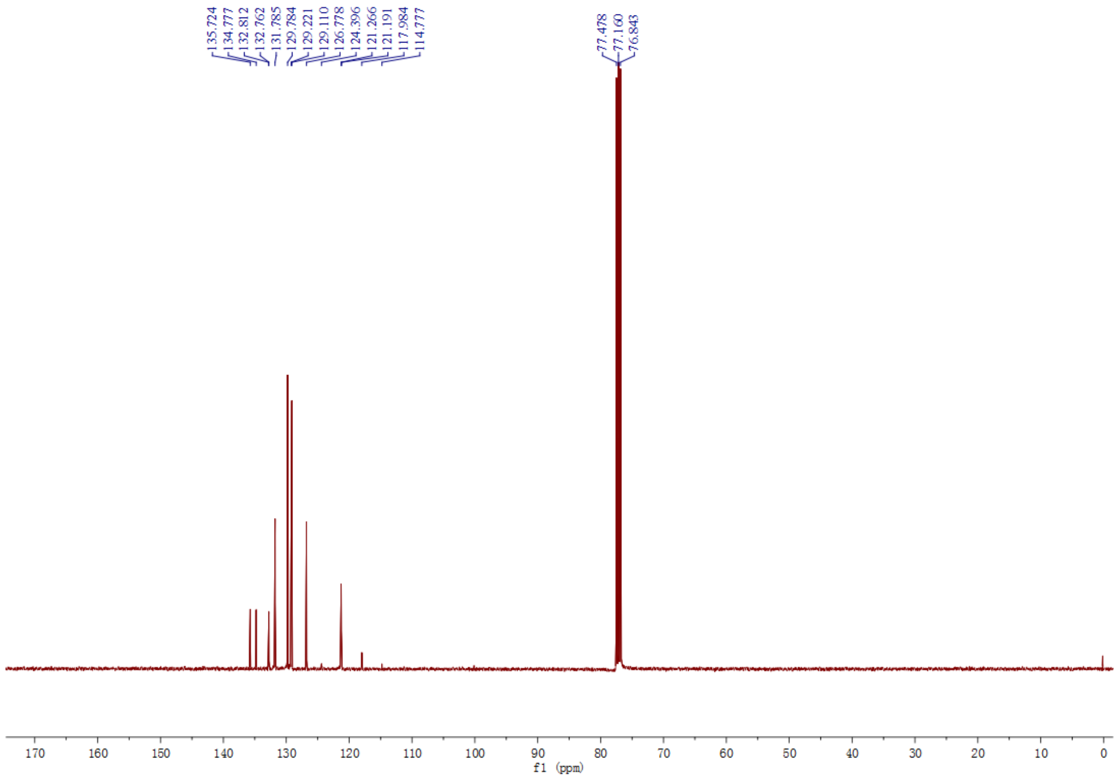

^{13}C NMR Spectrum for **3e** ($CDCl_3$, 100 MHz)

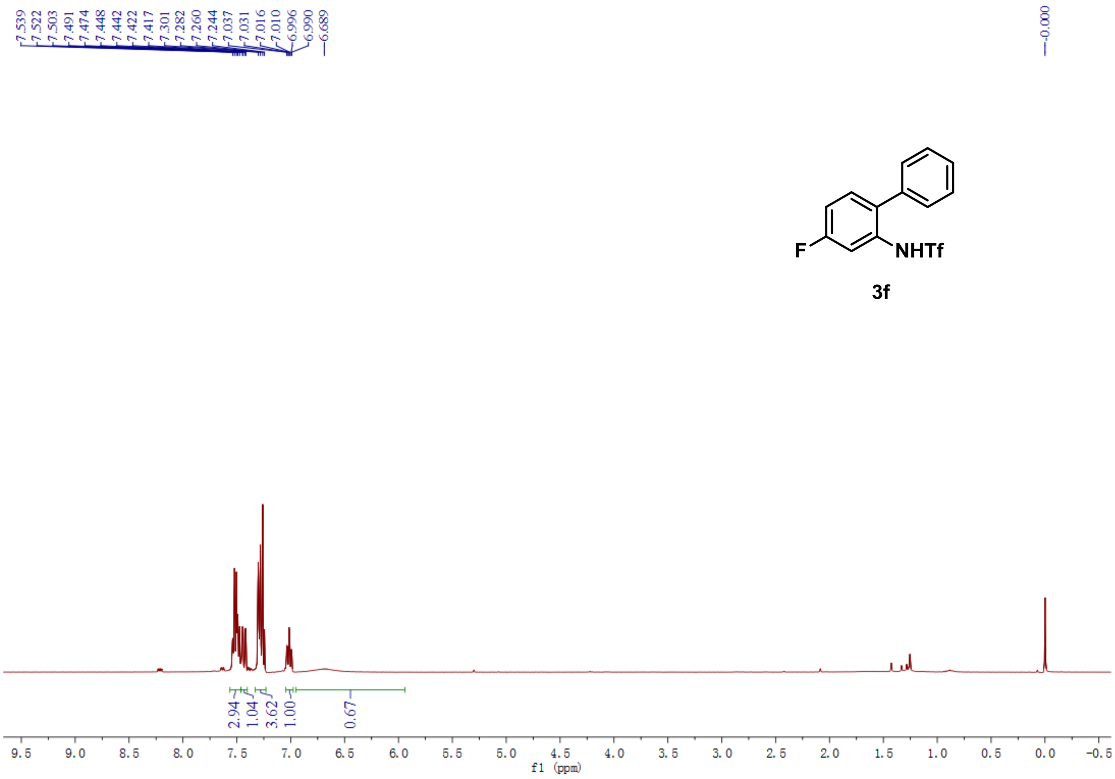

^{1}H NMR Spectrum for **3f** ($CDCl_3$, 400 MHz)

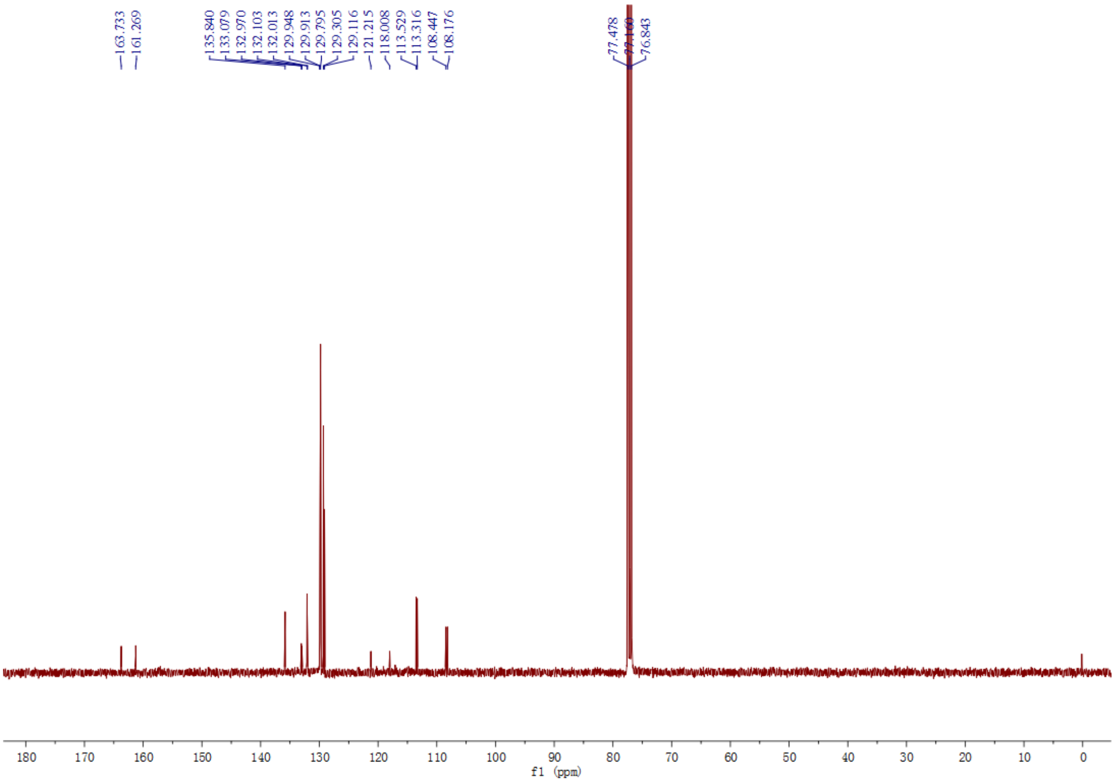

^{13}C NMR Spectrum for **3f** ($CDCl_3$, 100 MHz)

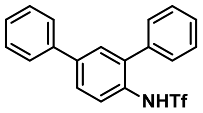

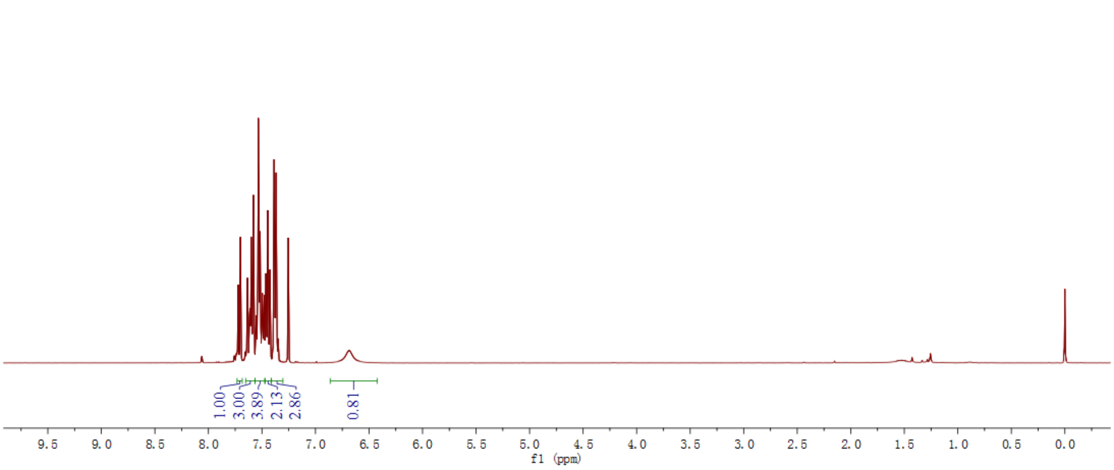

^{1}H NMR Spectrum for **3g** ($CDCl_3$, 400 MHz)

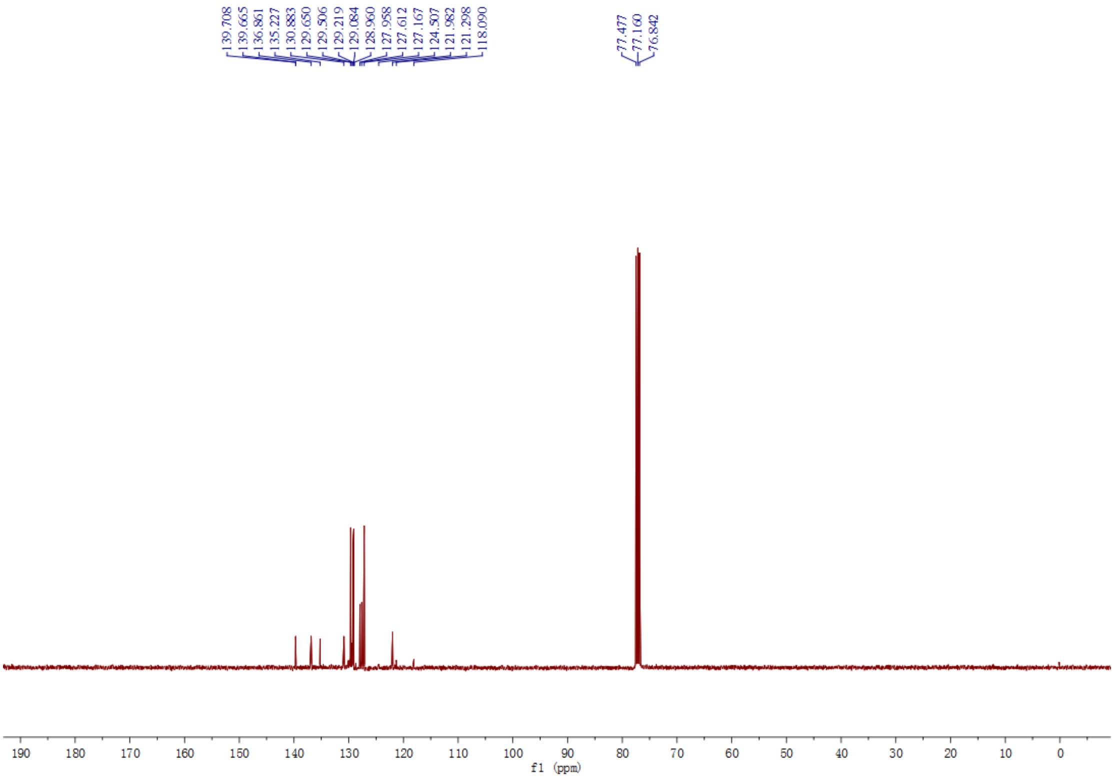

^{13}C NMR Spectrum for **3g** ($CDCl_3$, 100 MHz)

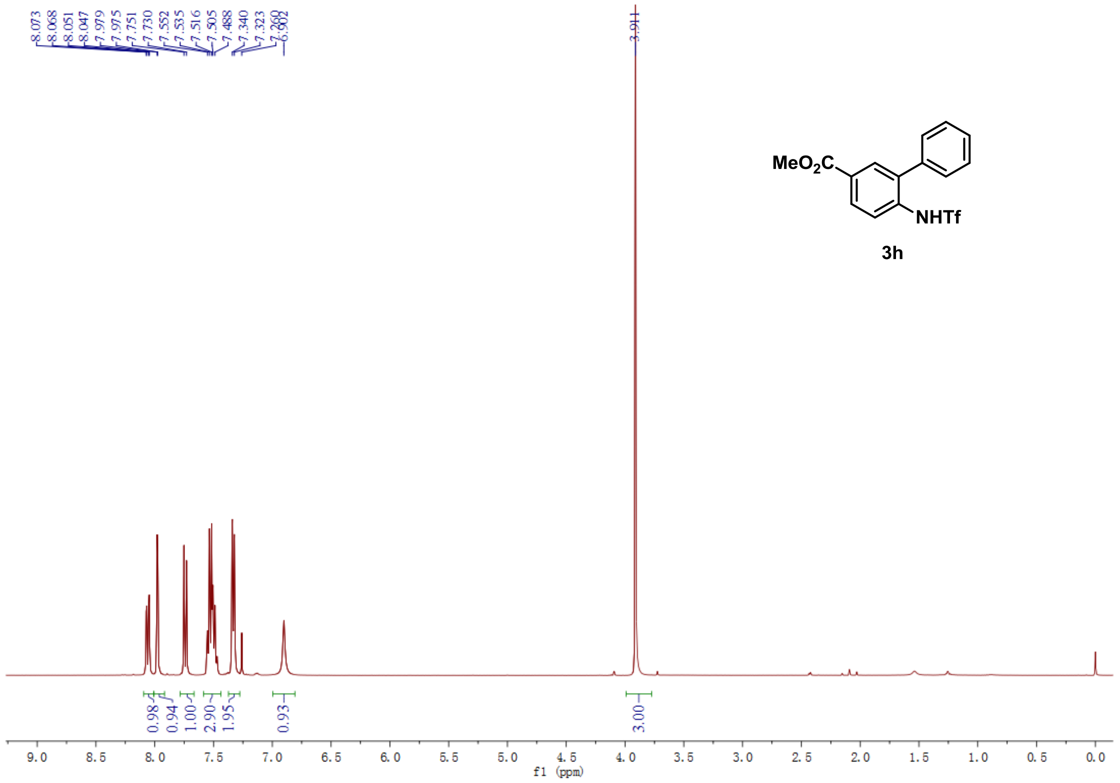

^{1}H NMR Spectrum for **3h** ($CDCl_3$, 400 MHz)

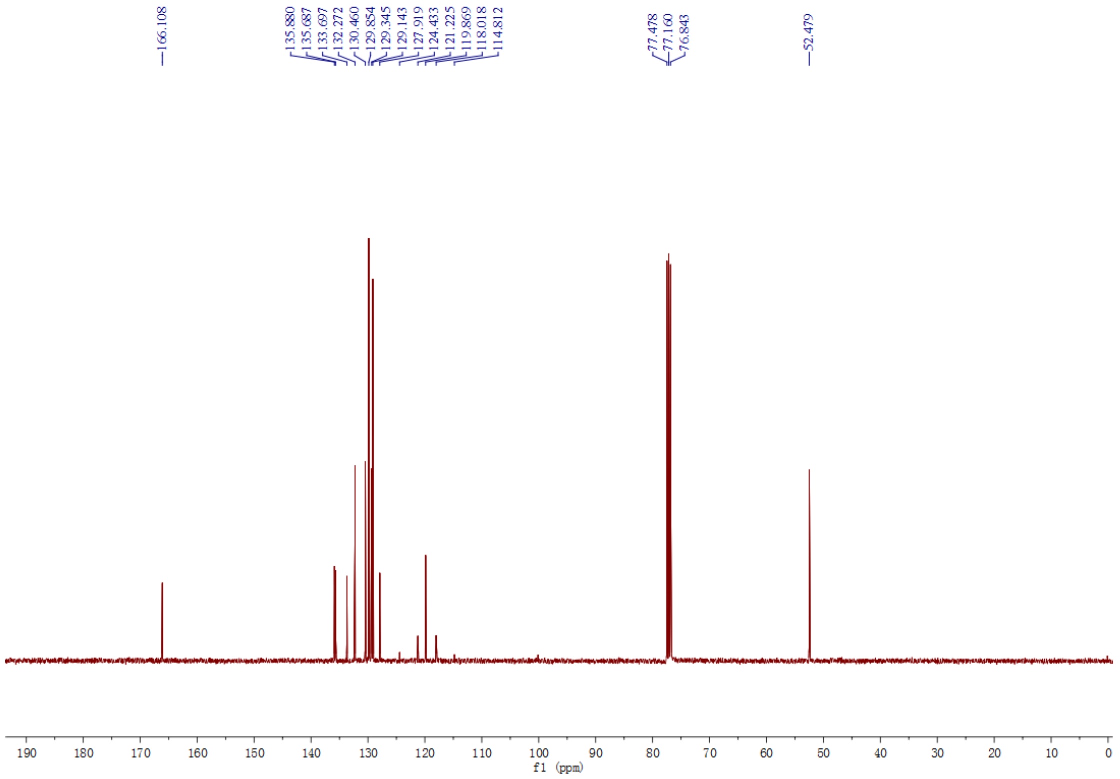

^{13}C NMR Spectrum for **3h** ($CDCl_3$, 100 MHz)

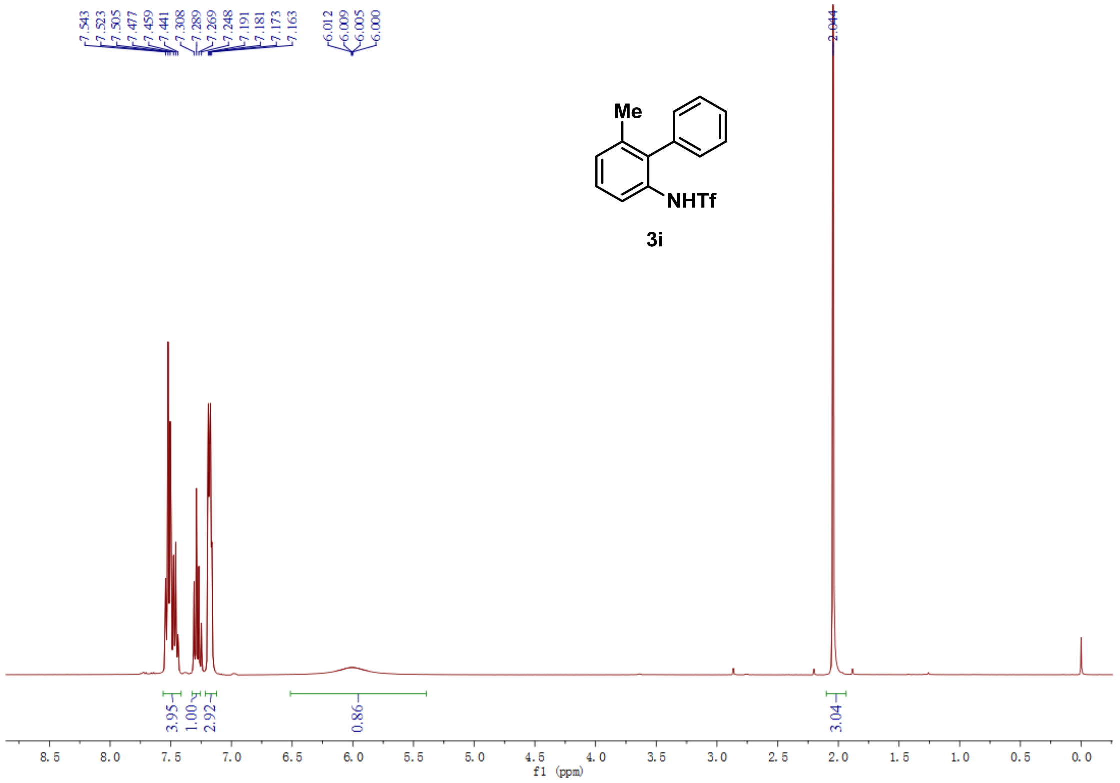

^{1}H NMR Spectrum for **3i** ($CDCl_3$, 400 MHz)

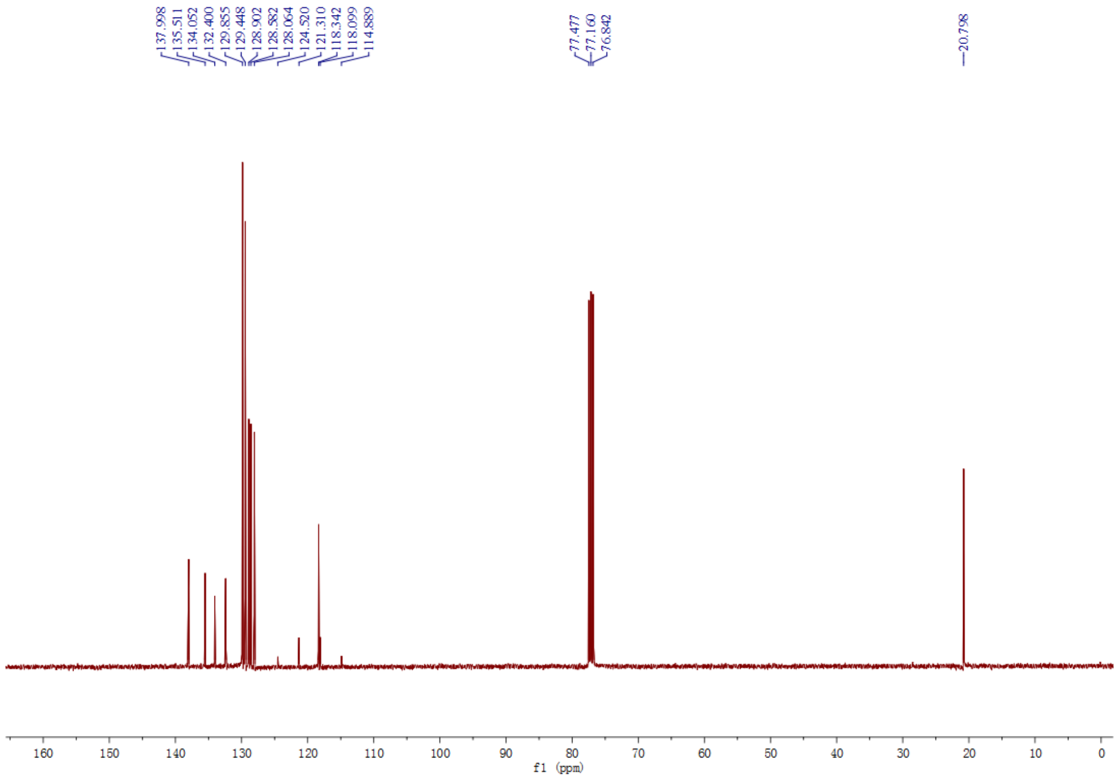

^{13}C NMR Spectrum for **3i** ($CDCl_3$, 100 MHz)

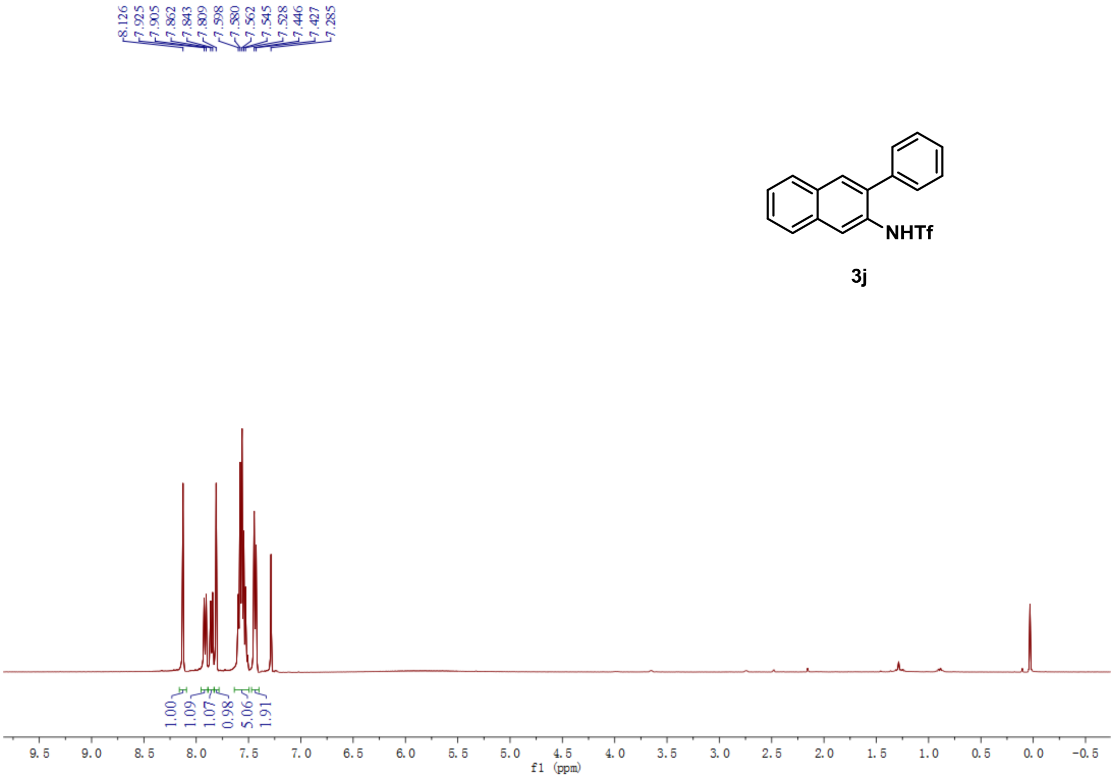

^{1}H NMR Spectrum for **3j** ($CDCl_3$, 400 MHz)

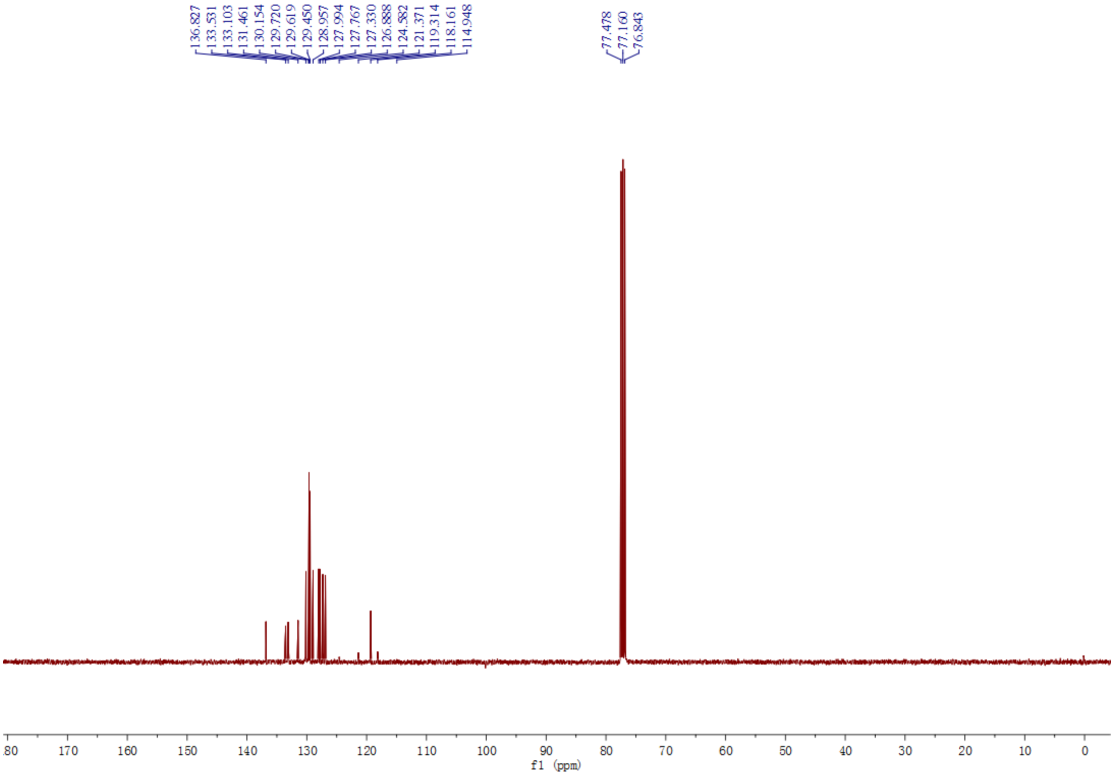

^{13}C NMR Spectrum for **3j** ($CDCl_3$, 100 MHz)

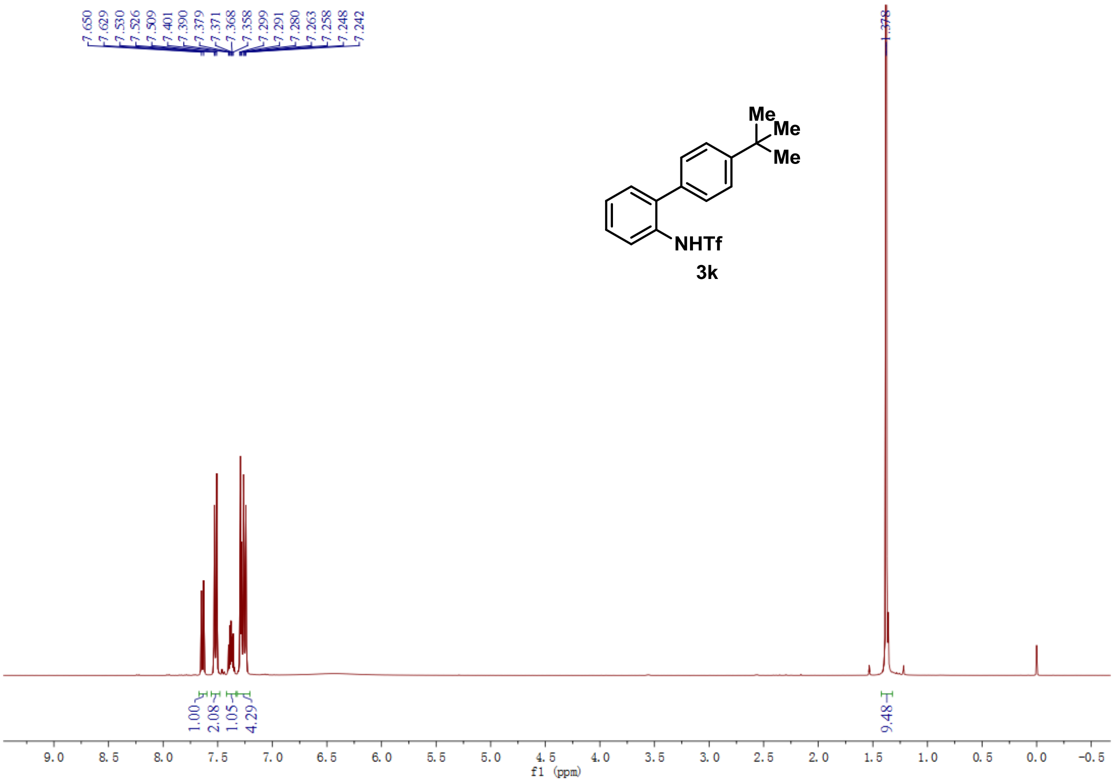

^{1}H NMR Spectrum for **3k** (CDCl$_3$, 400 MHz)

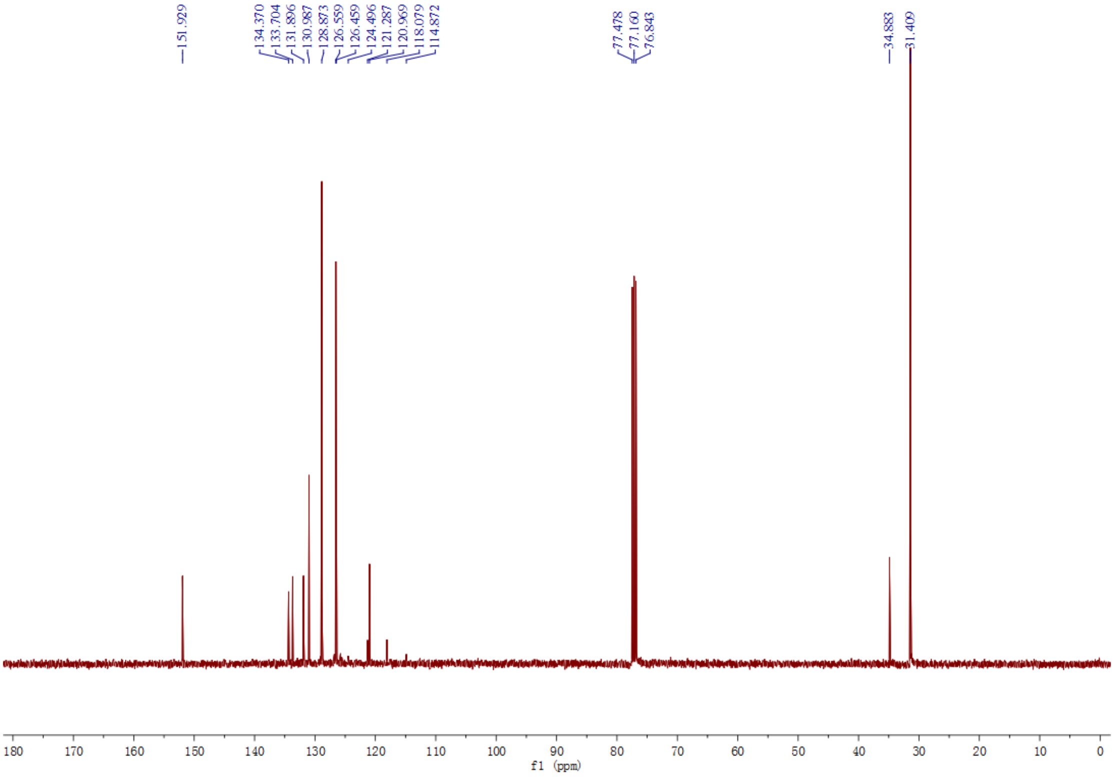

^{13}C NMR Spectrum for **3k** (CDCl$_3$, 100 MHz)

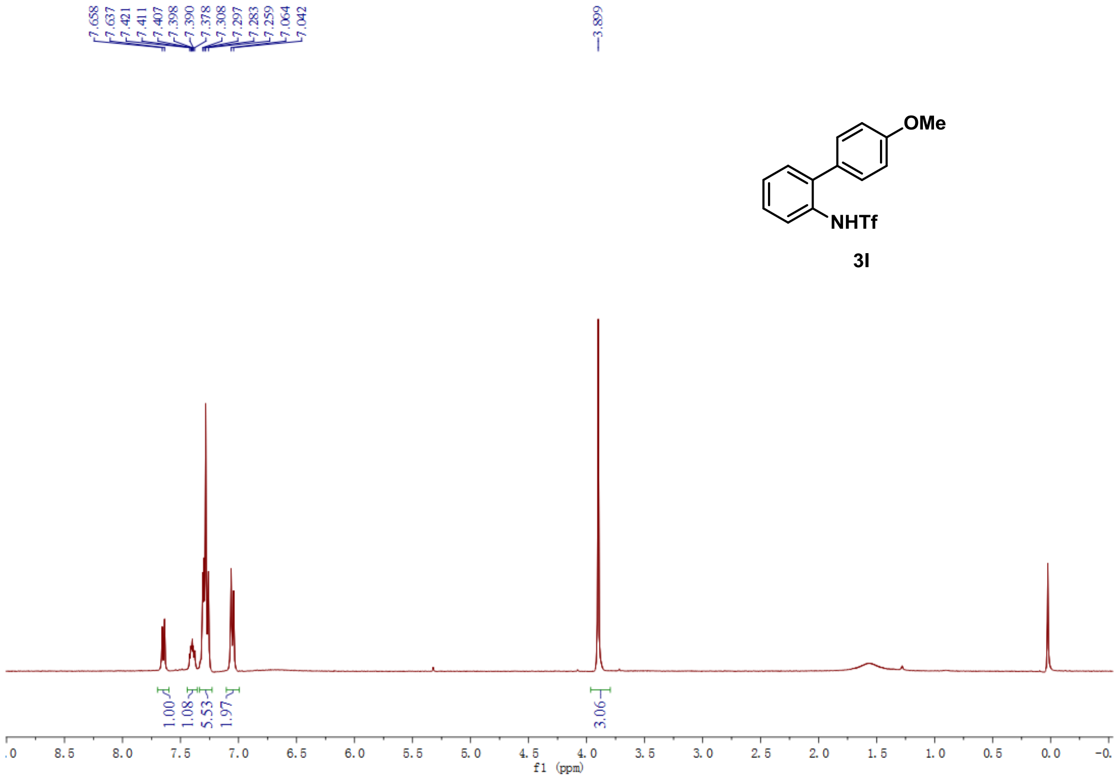

^{1}H NMR Spectrum for **3l** ($CDCl_3$, 400 MHz)

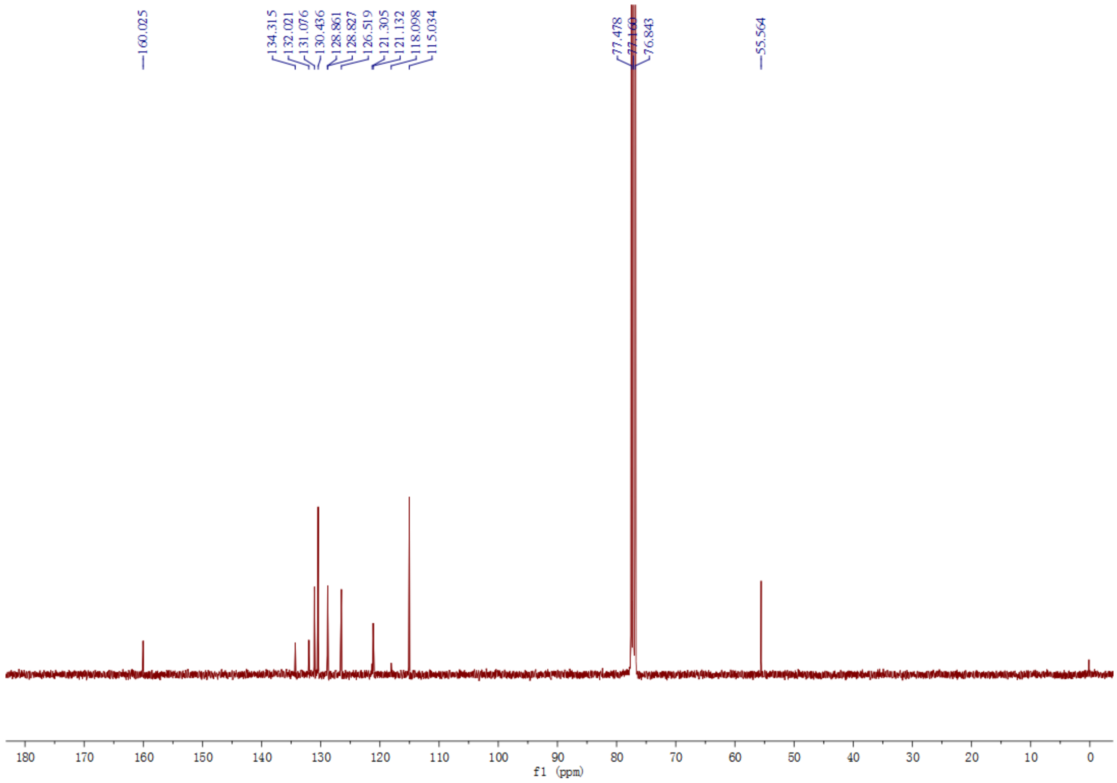

^{13}C NMR Spectrum for **3l** ($CDCl_3$, 100 MHz)

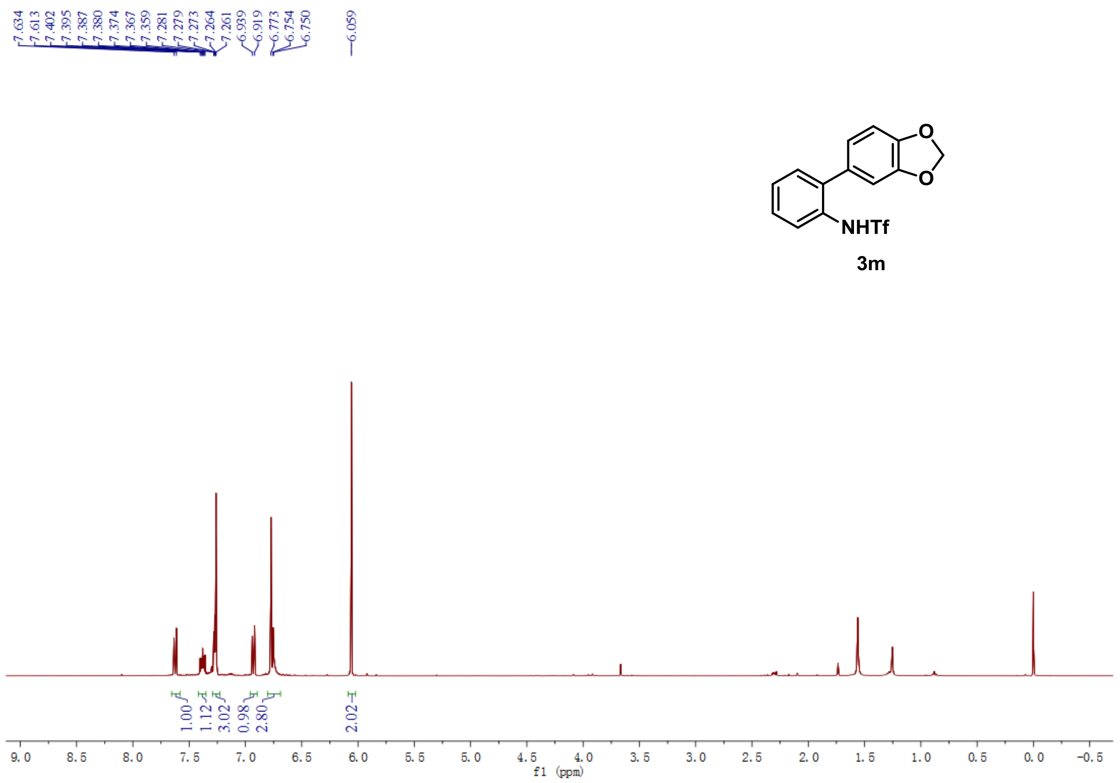

^{1}H NMR Spectrum for **3m** ($CDCl_3$, 400 MHz)

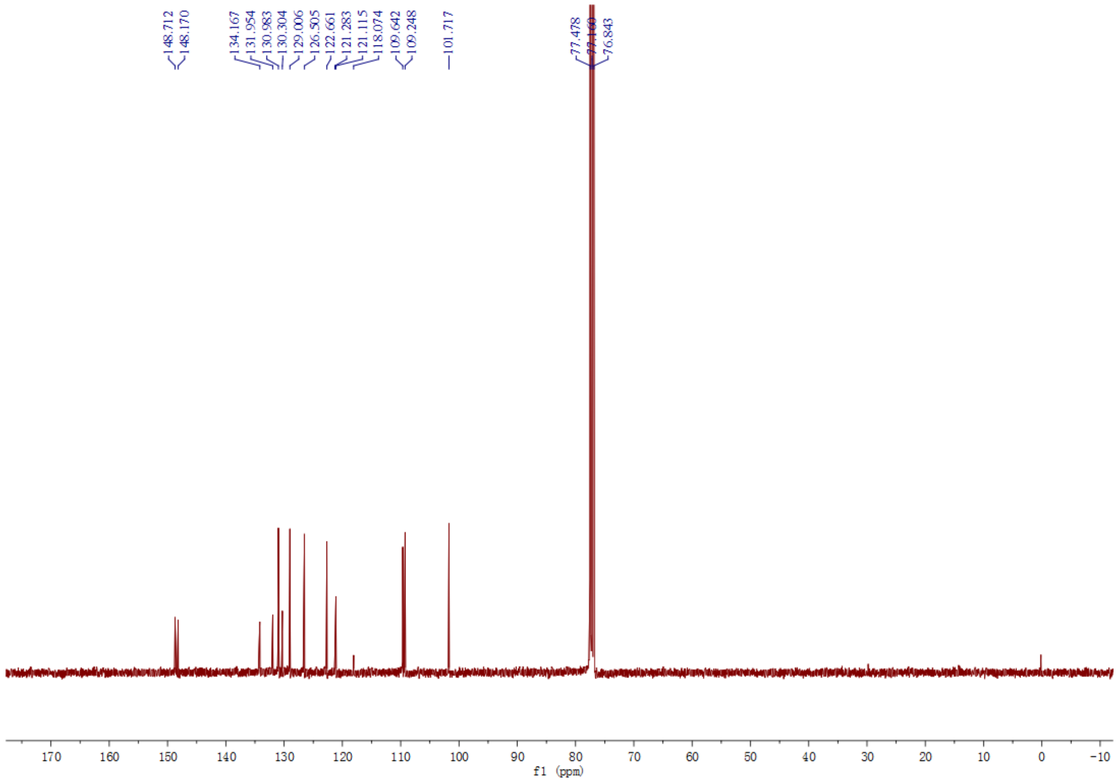

^{13}C NMR Spectrum for **3m** ($CDCl_3$, 100 MHz)

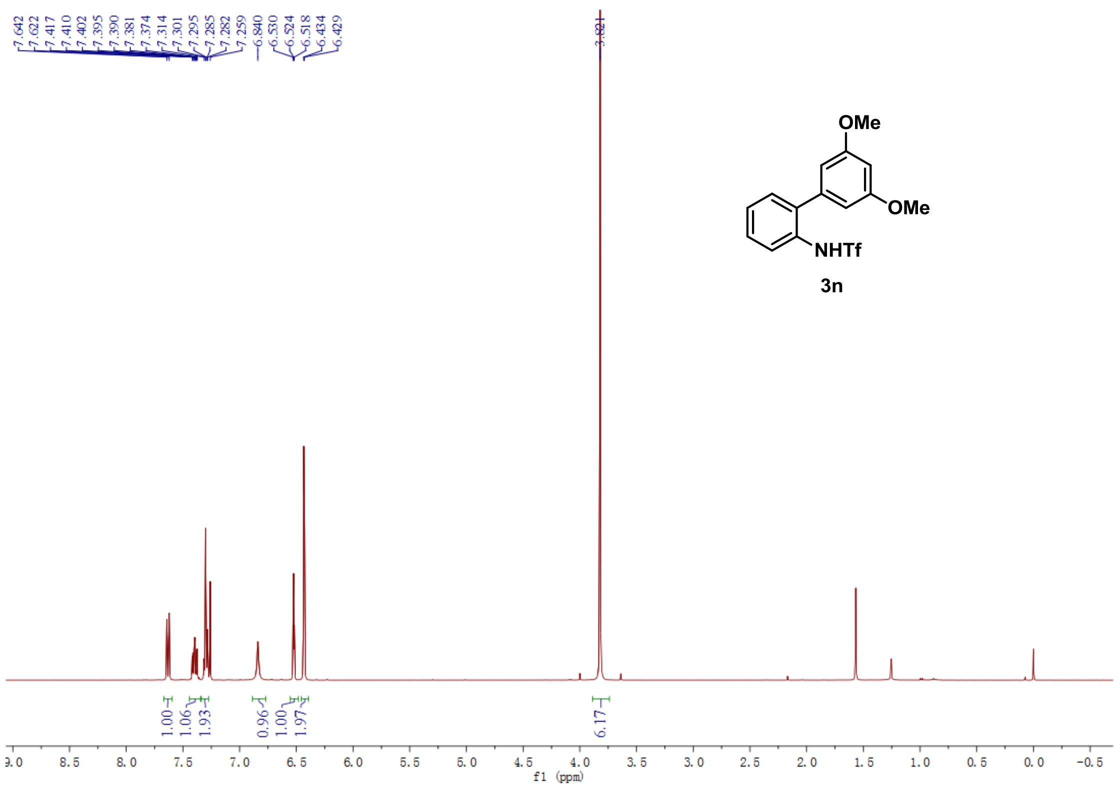

^{1}H NMR Spectrum for **3n** ($CDCl_3$, 400 MHz)

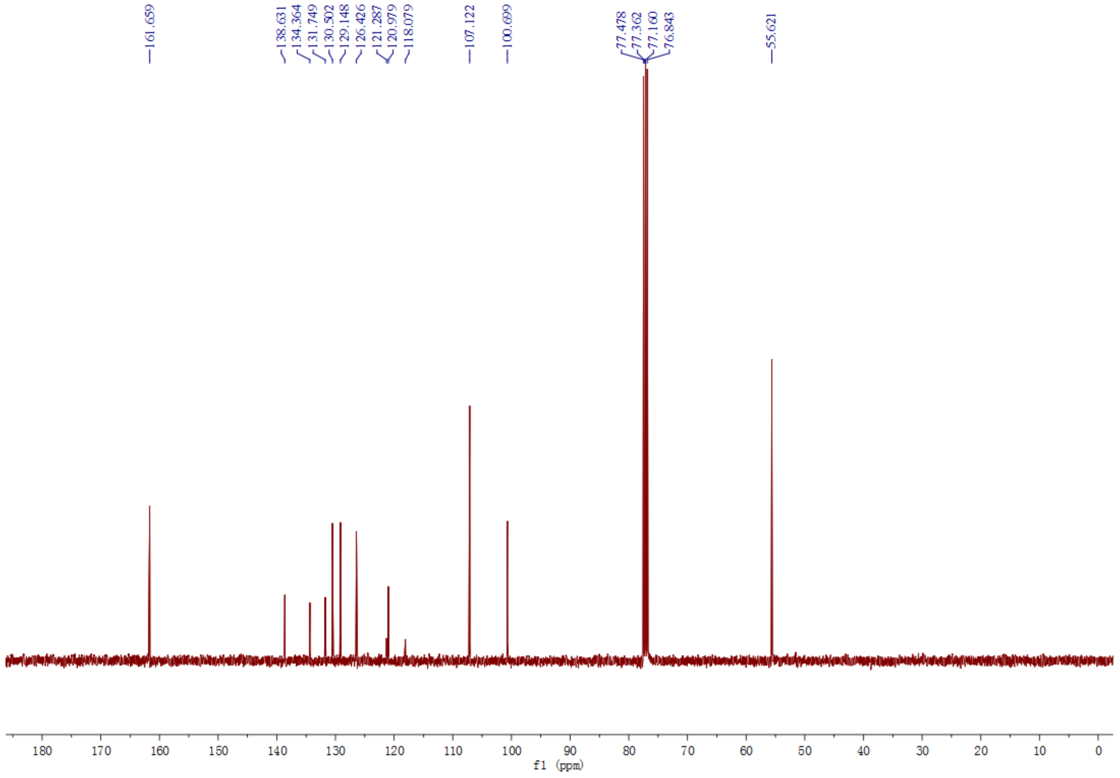

^{13}C NMR Spectrum for **3n** ($CDCl_3$, 100 MHz)

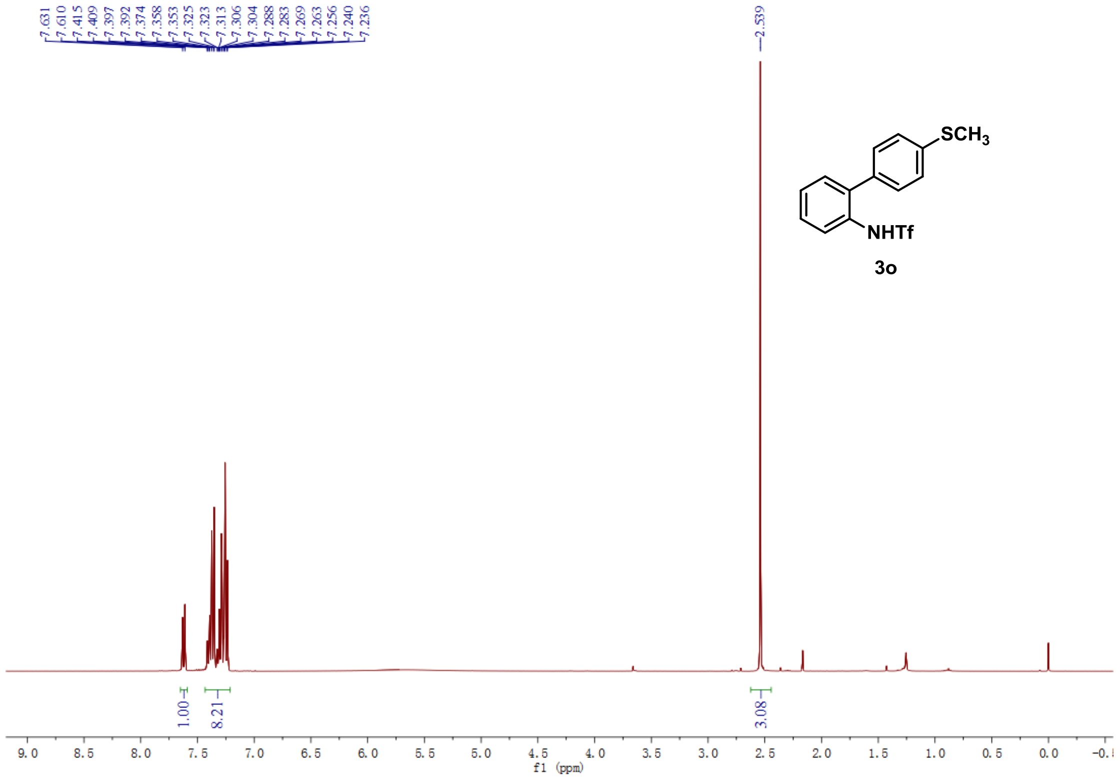

^{1}H NMR Spectrum for **3o** ($CDCl_3$, 400 MHz)

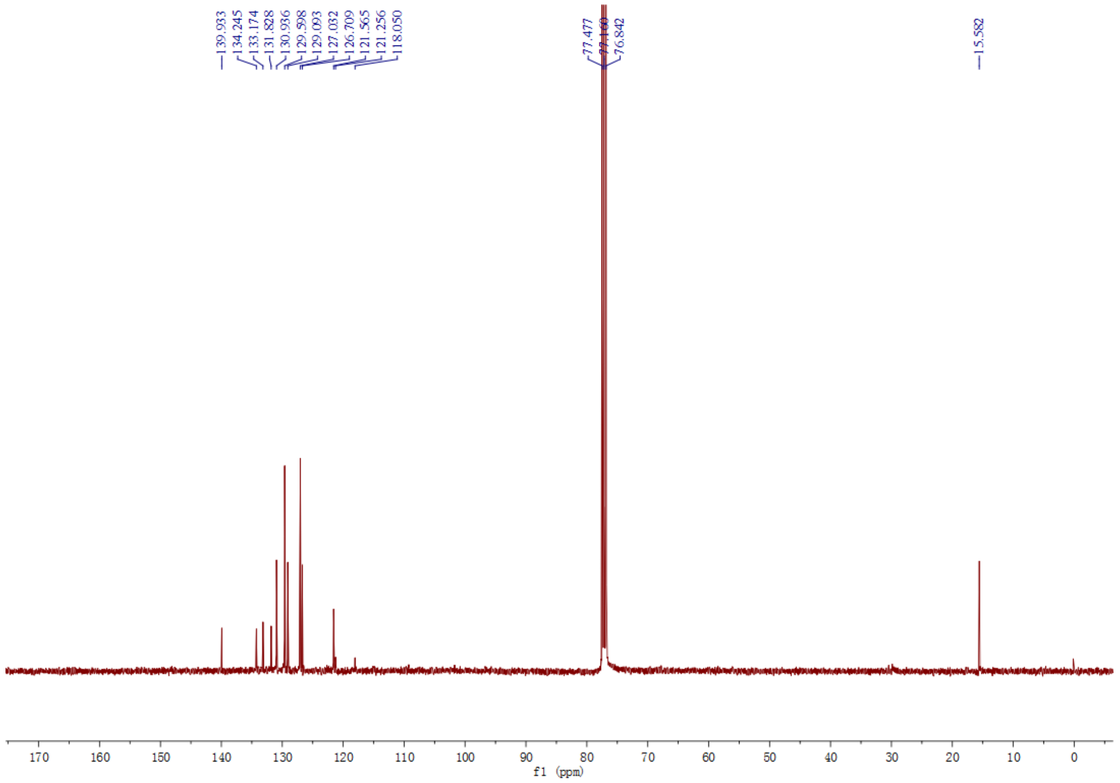

^{13}C NMR Spectrum for **3o** ($CDCl_3$, 100 MHz)

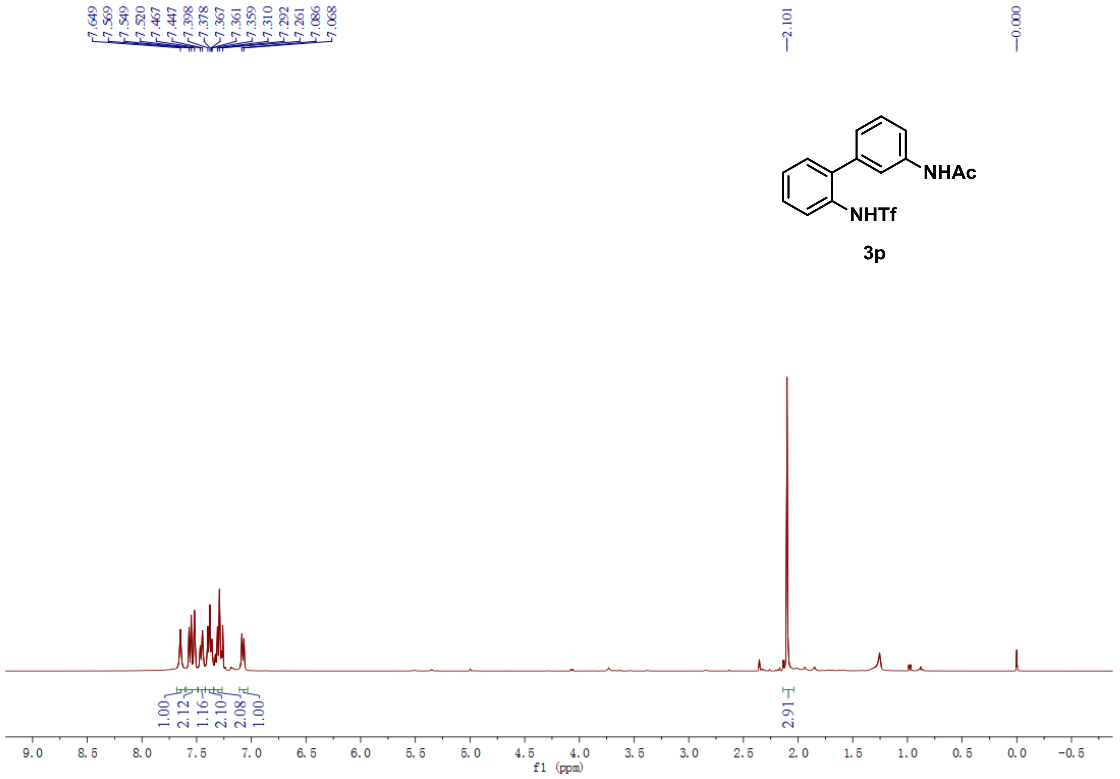

^{1}H NMR Spectrum for **3p** ($CDCl_3$, 400 MHz)

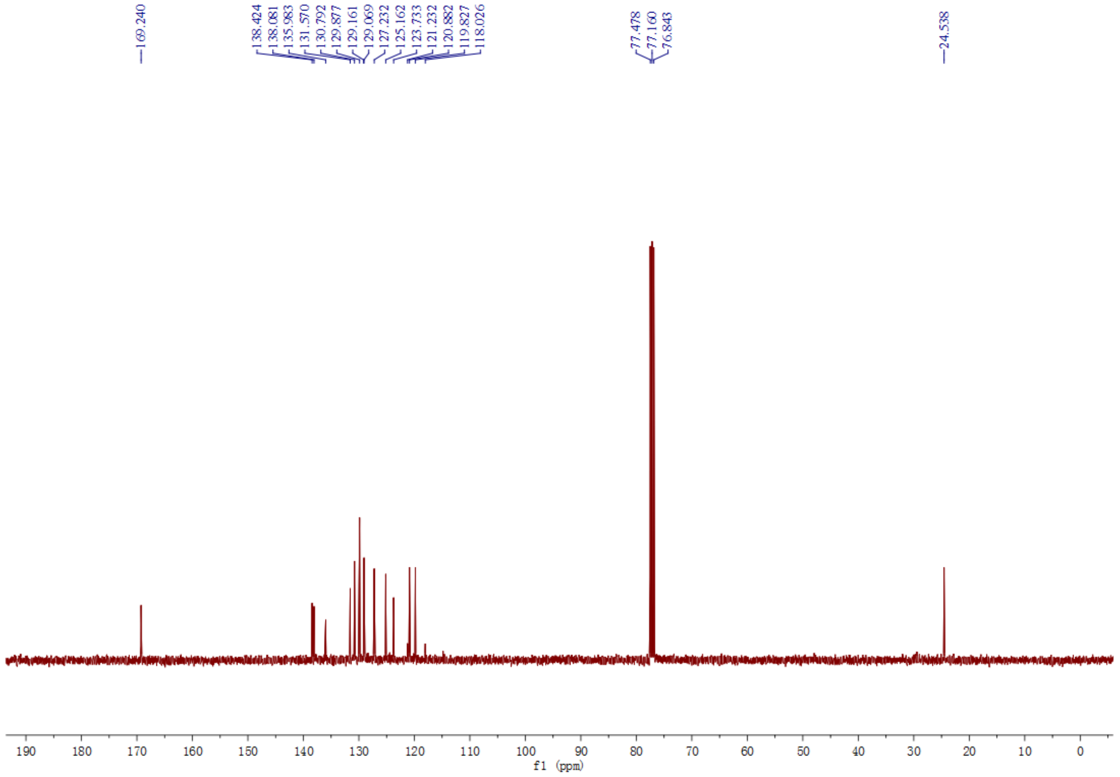

^{13}C NMR Spectrum for **3p** ($CDCl_3$, 100 MHz)

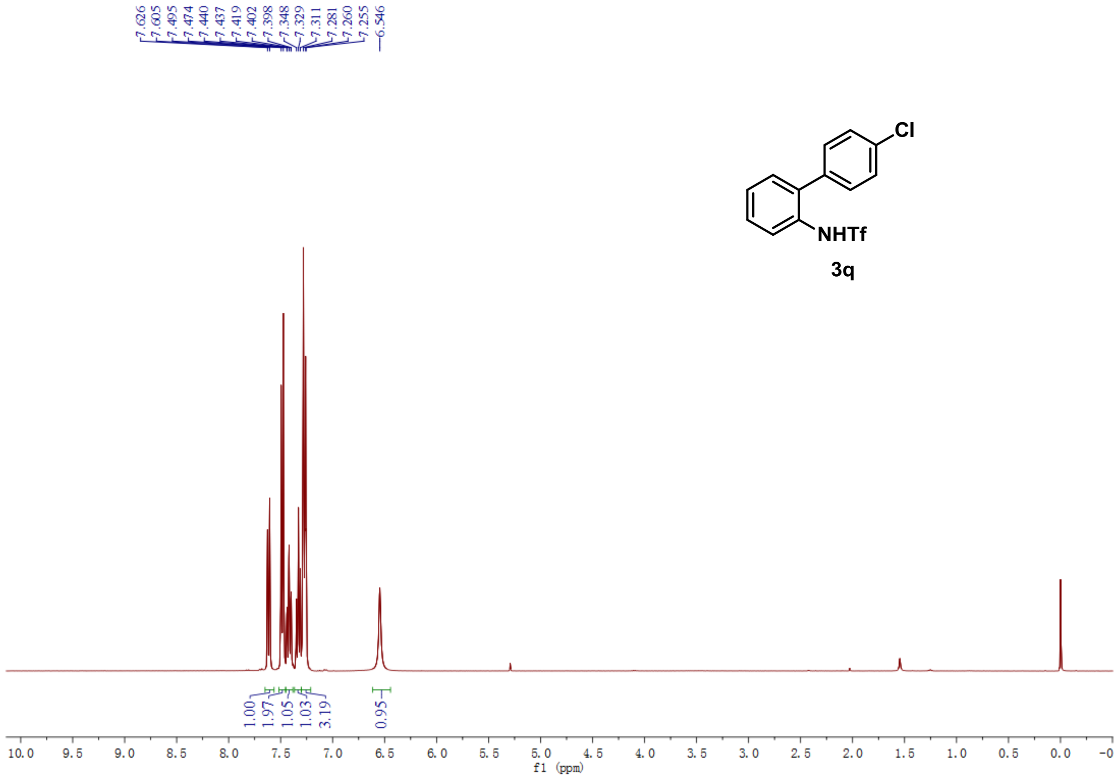

^{1}H NMR Spectrum for **3q** ($CDCl_3$, 400 MHz)

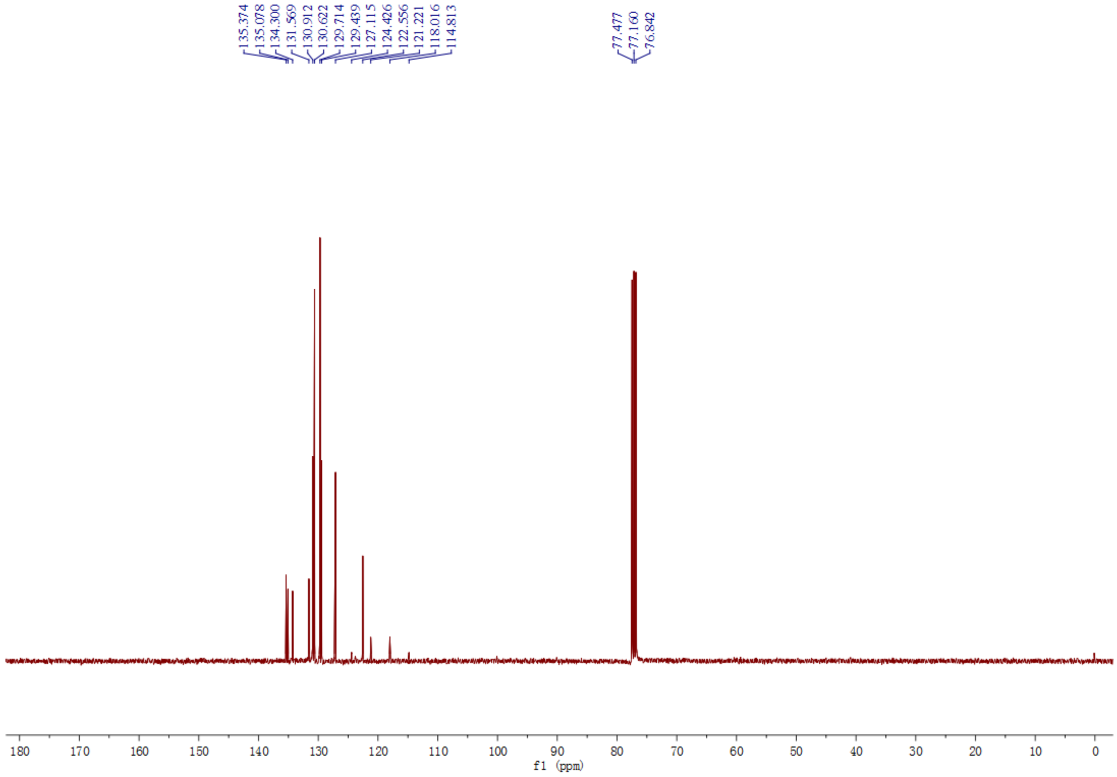

^{13}C NMR Spectrum for **3q** ($CDCl_3$, 100 MHz)

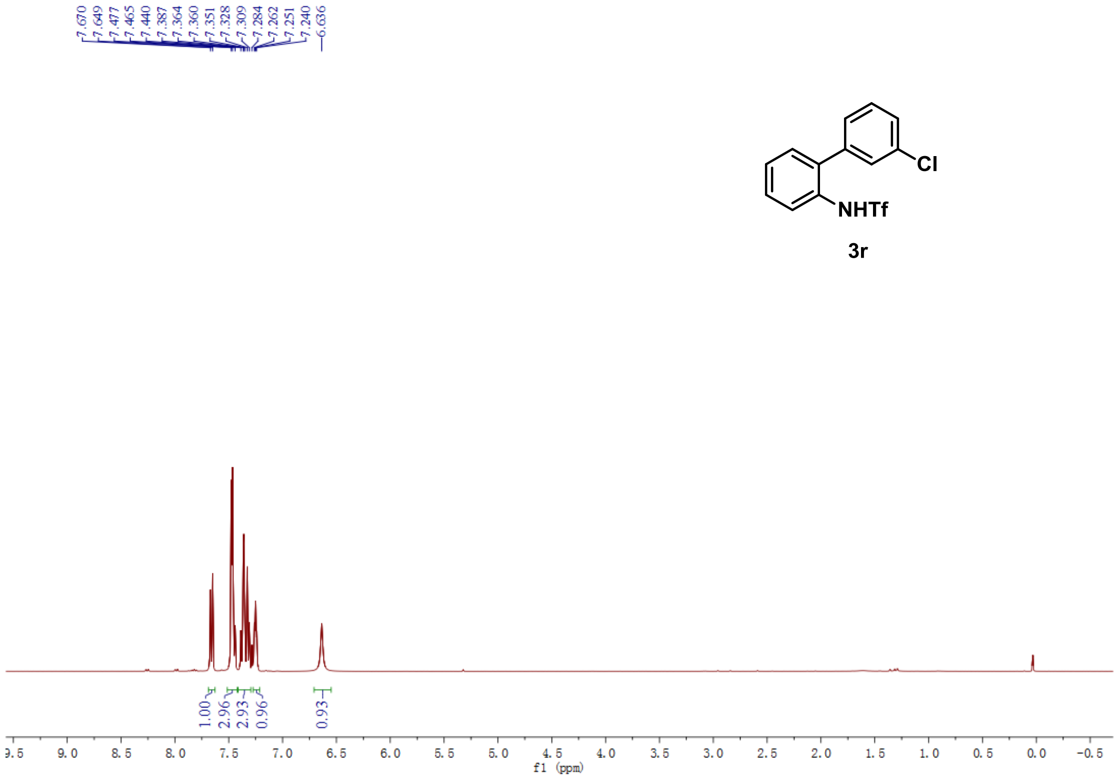

^{1}H NMR Spectrum for **3r** ($CDCl_3$, 400 MHz)

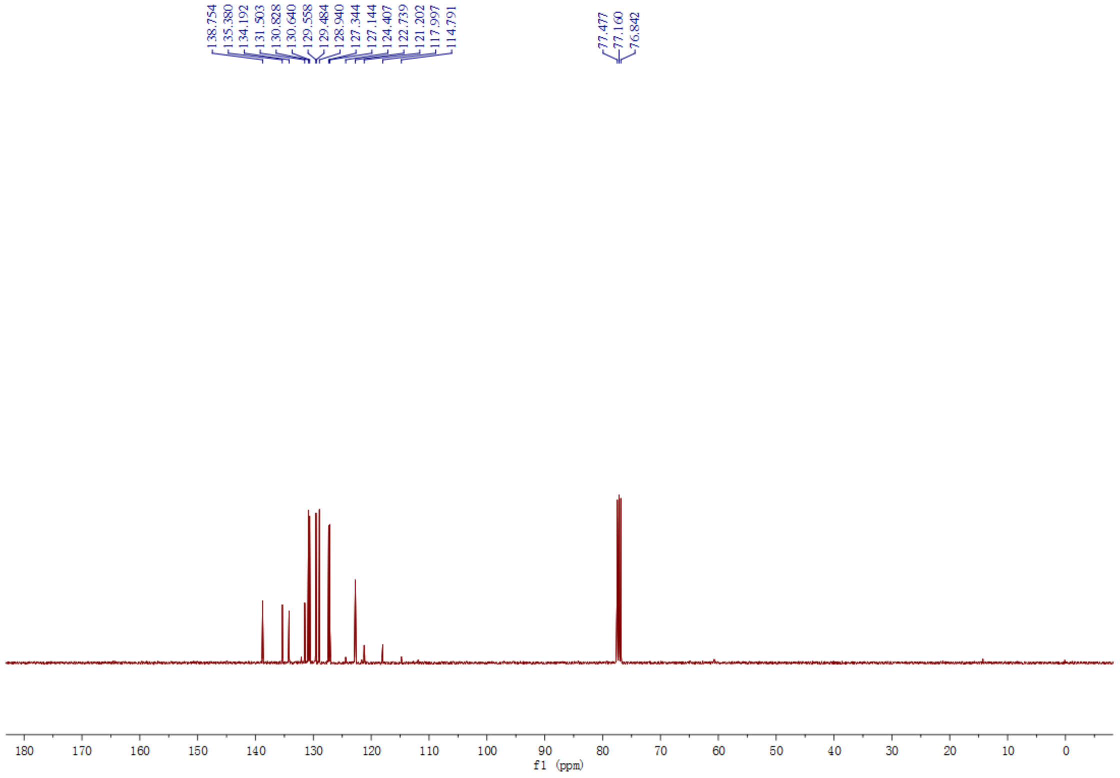

^{13}C NMR Spectrum for **3r** ($CDCl_3$, 100 MHz)

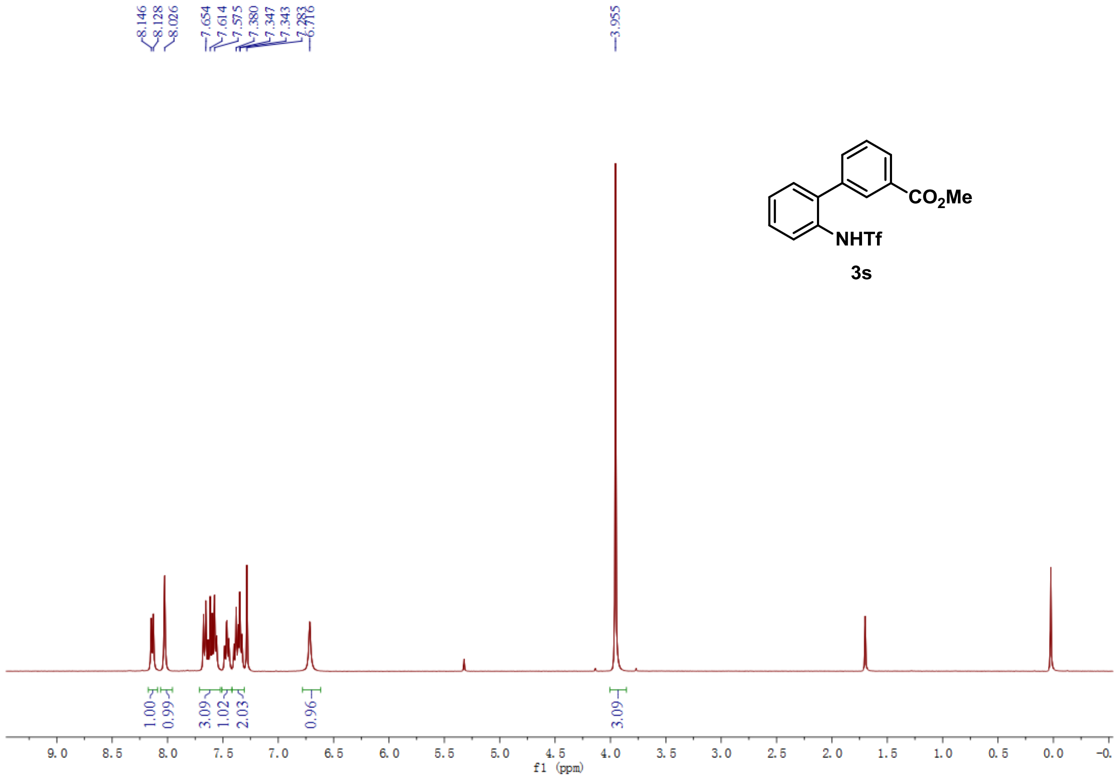

^{1}H NMR Spectrum for **3s** ($CDCl_3$, 400 MHz)

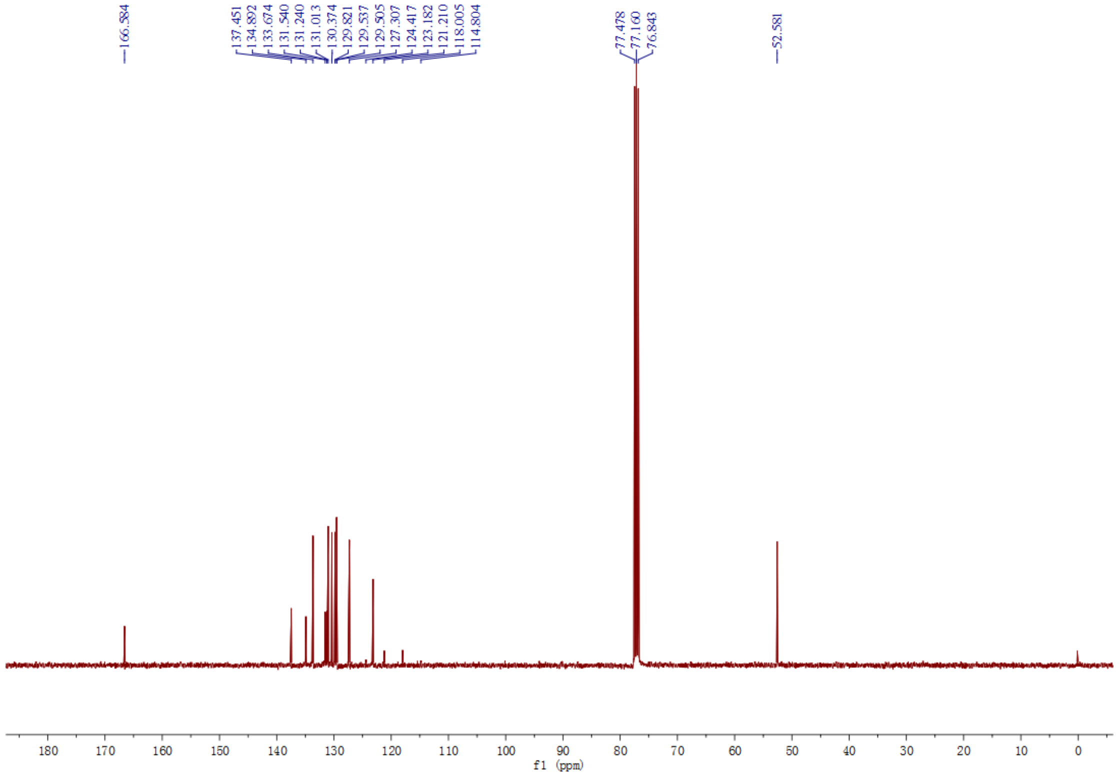

^{13}C NMR Spectrum for **3s** ($CDCl_3$, 100 MHz)

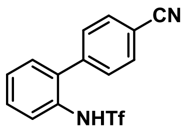

3t

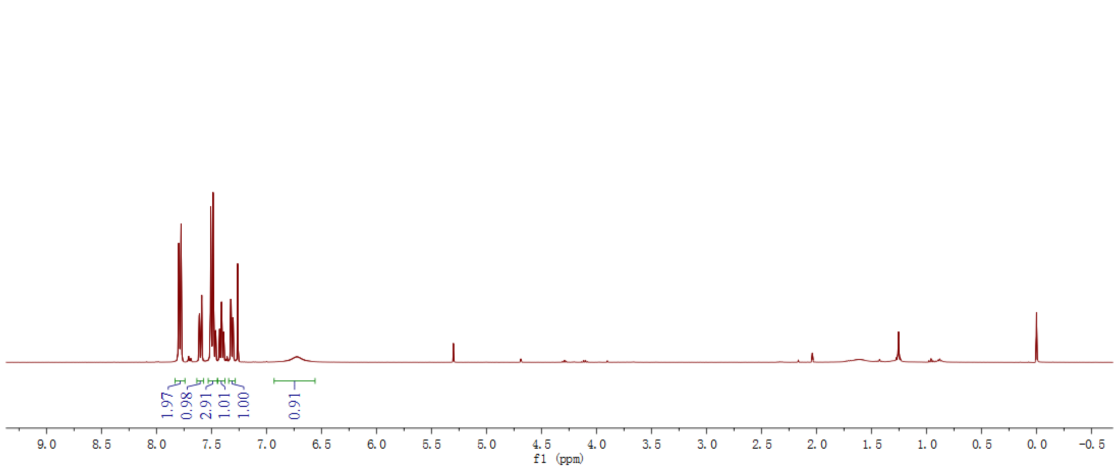

^{1}H NMR Spectrum for **3t** ($CDCl_3$, 400 MHz)

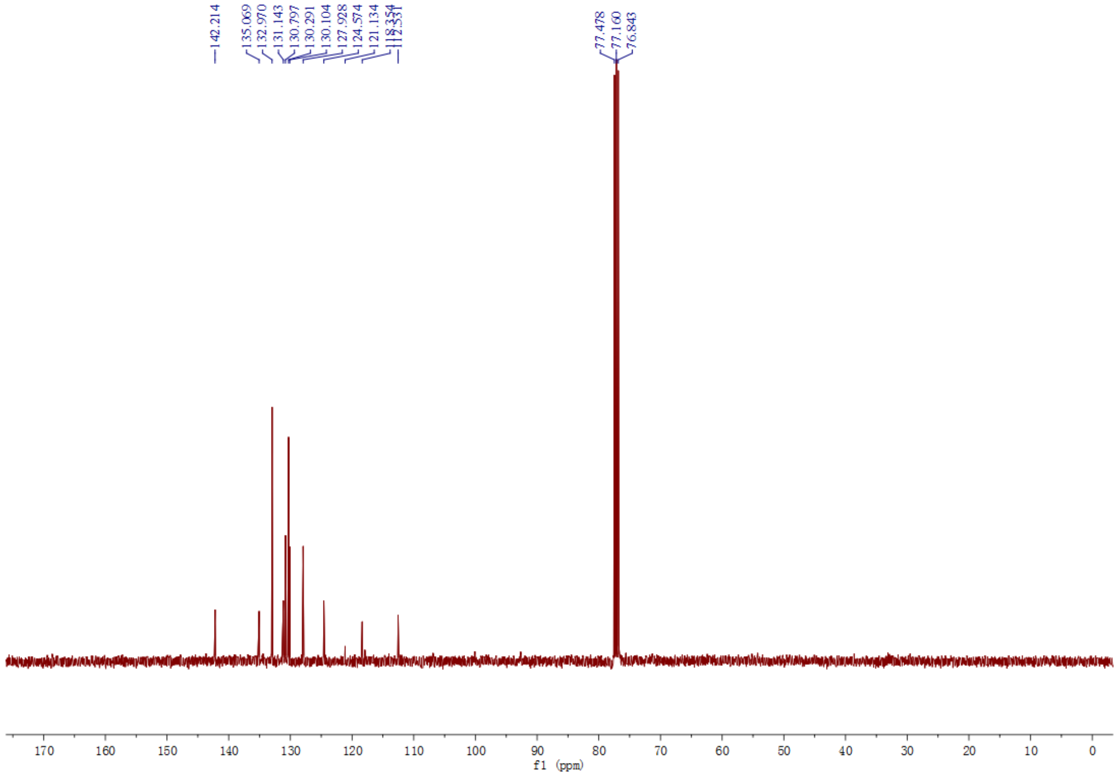

^{13}C NMR Spectrum for **3t** ($CDCl_3$, 100 MHz)

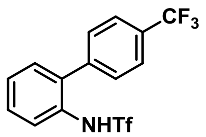

3u

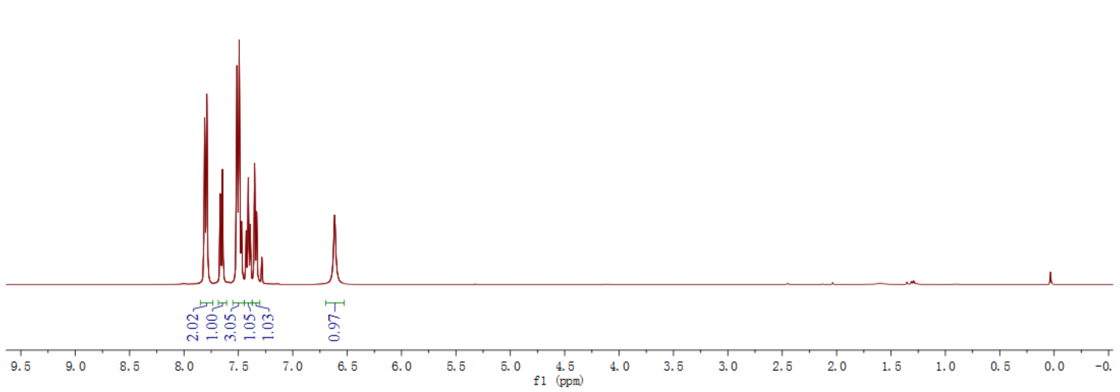

^{1}H NMR Spectrum for **3u** ($CDCl_3$, 400 MHz)

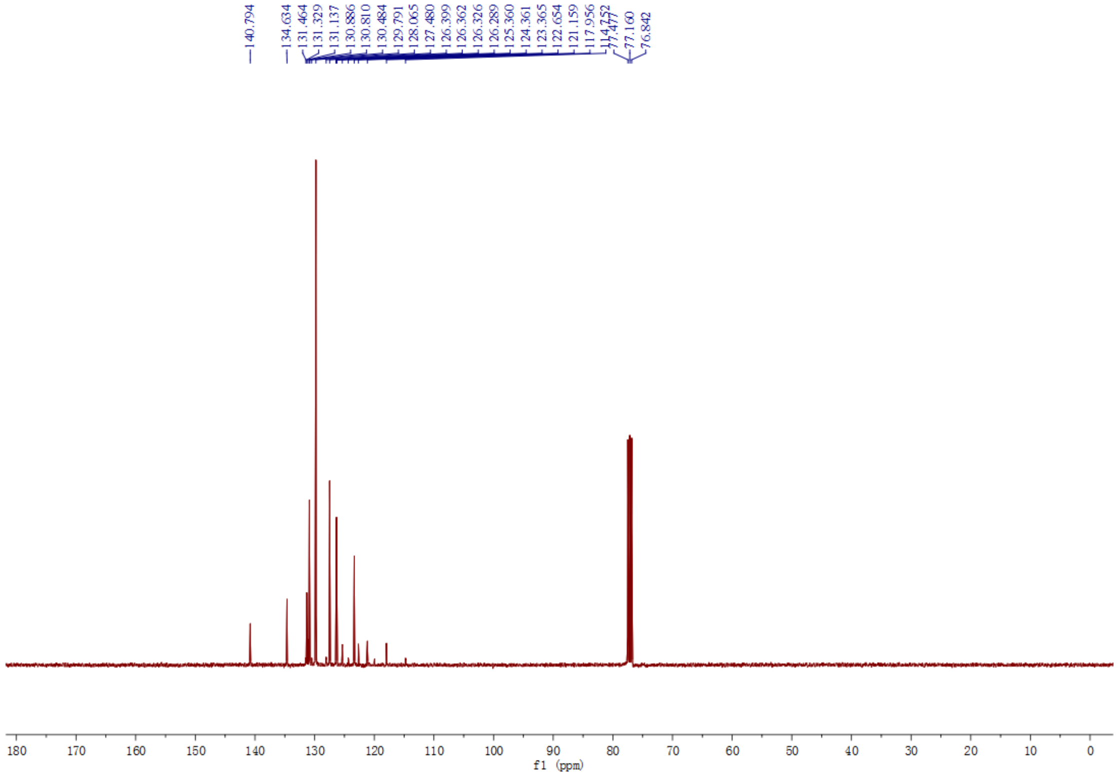

^{13}C NMR Spectrum for **3u** ($CDCl_3$, 100 MHz)

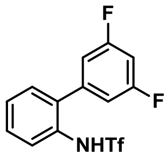

3v

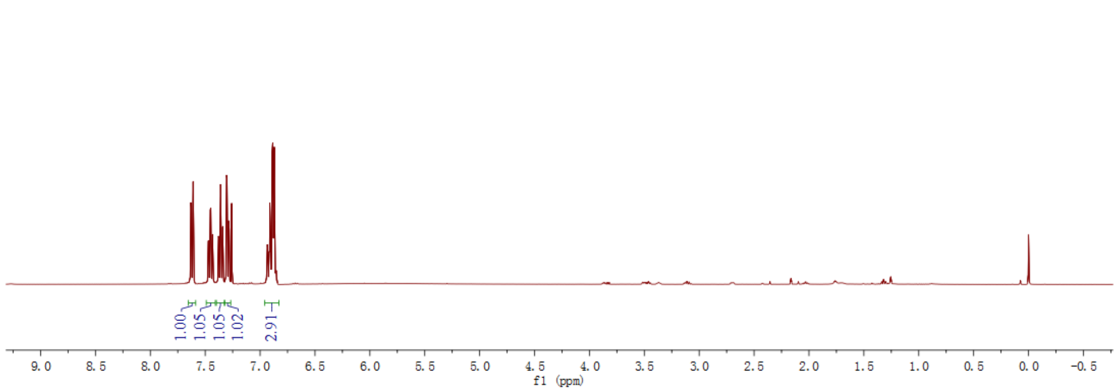

^{1}H NMR Spectrum for **3v** ($CDCl_3$, 400 MHz)

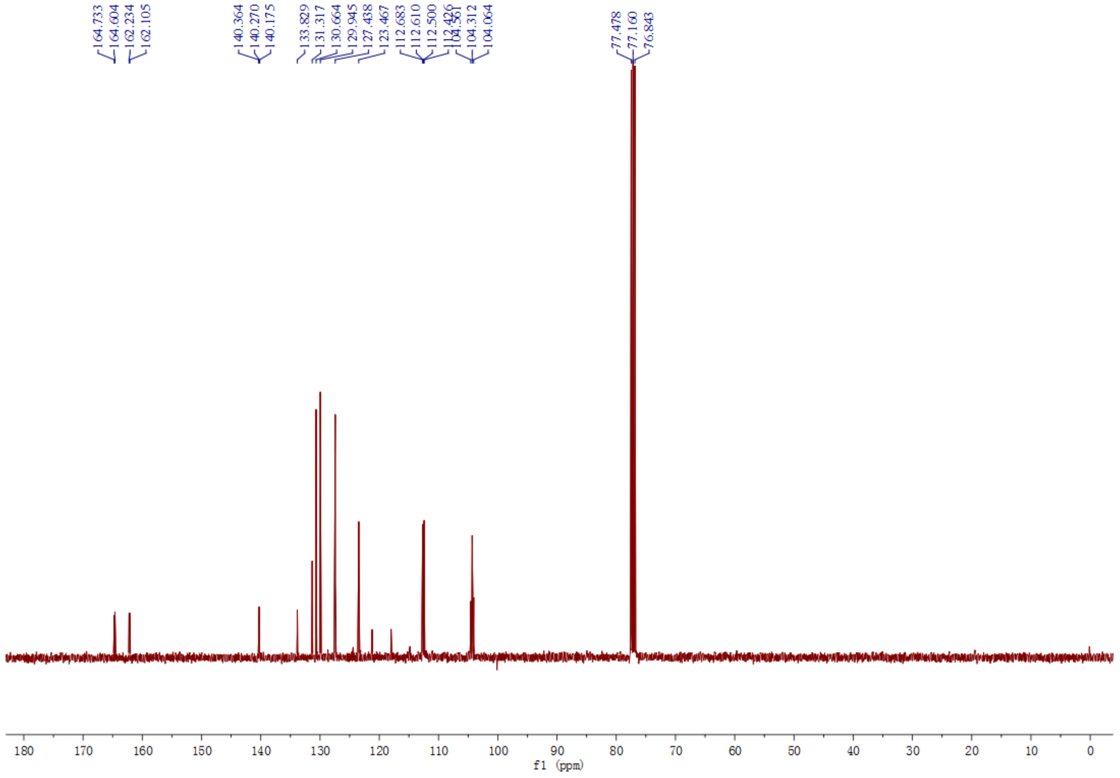

^{13}C NMR Spectrum for **3v** ($CDCl_3$, 100 MHz)

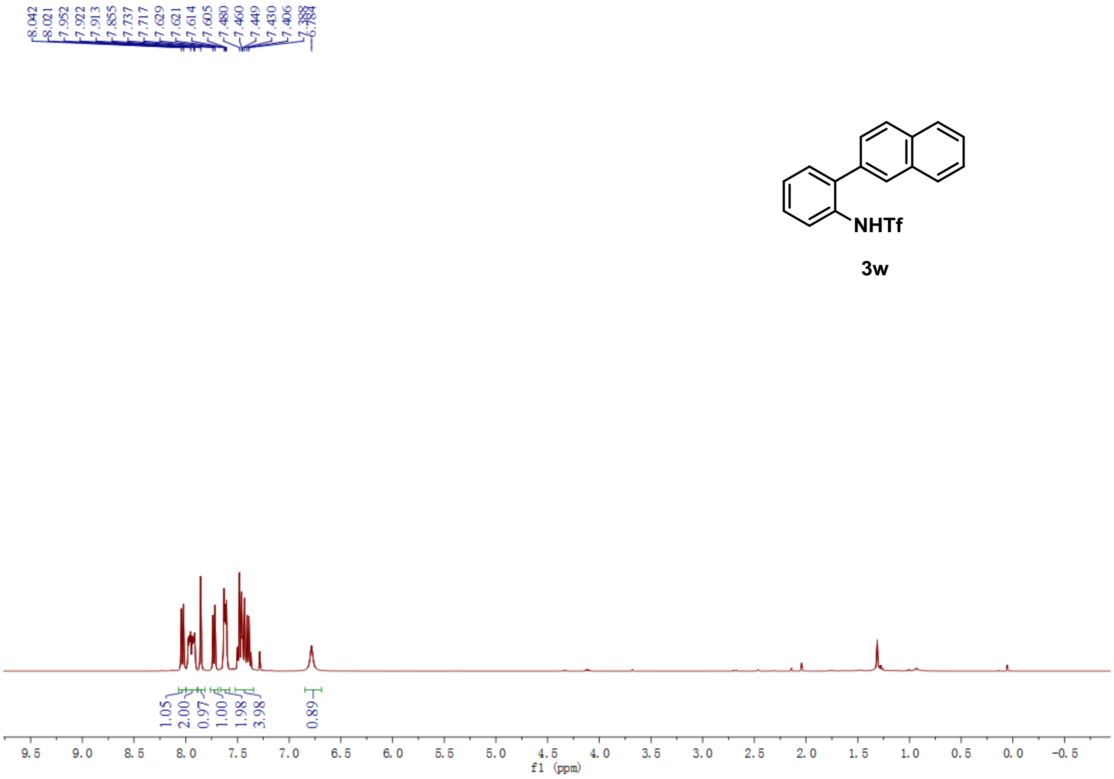

^{1}H NMR Spectrum for **3w** ($CDCl_3$, 400 MHz)

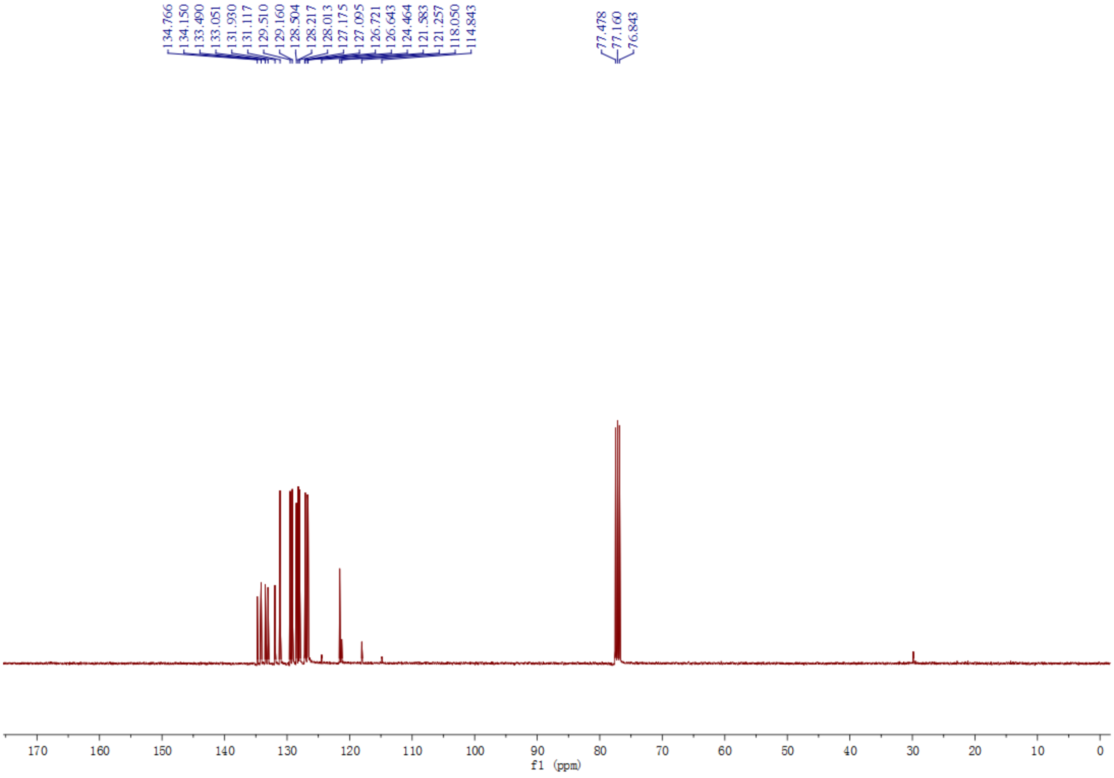

^{13}C NMR Spectrum for **3w** ($CDCl_3$, 100 MHz)

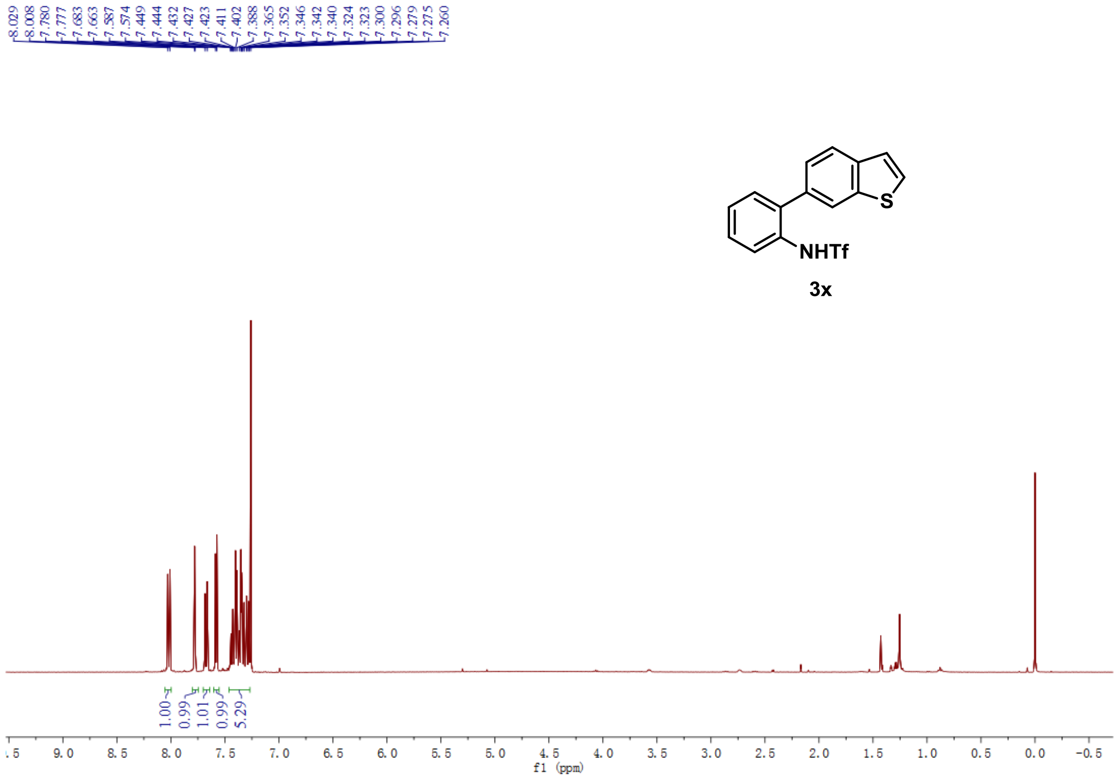

^{1}H NMR Spectrum for **3x** ($CDCl_3$, 400 MHz)

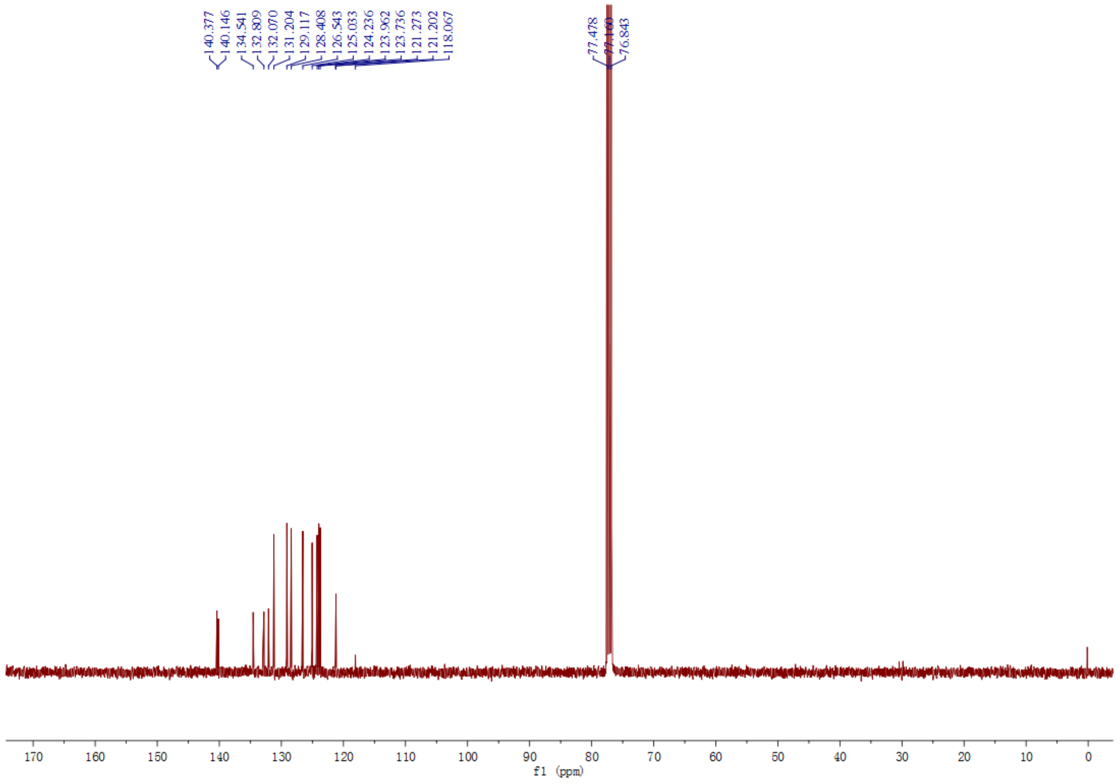

^{13}C NMR Spectrum for **3x** ($CDCl_3$, 100 MHz)

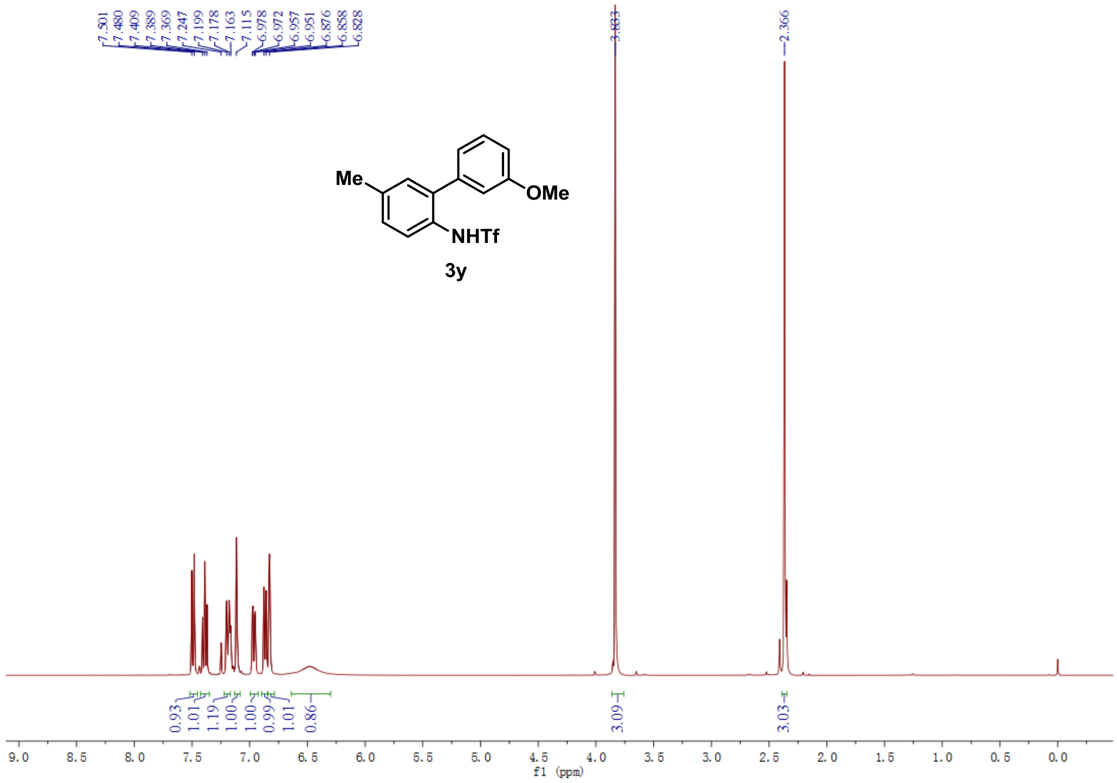

^{1}H NMR Spectrum for **3y** ($CDCl_3$, 400 MHz)

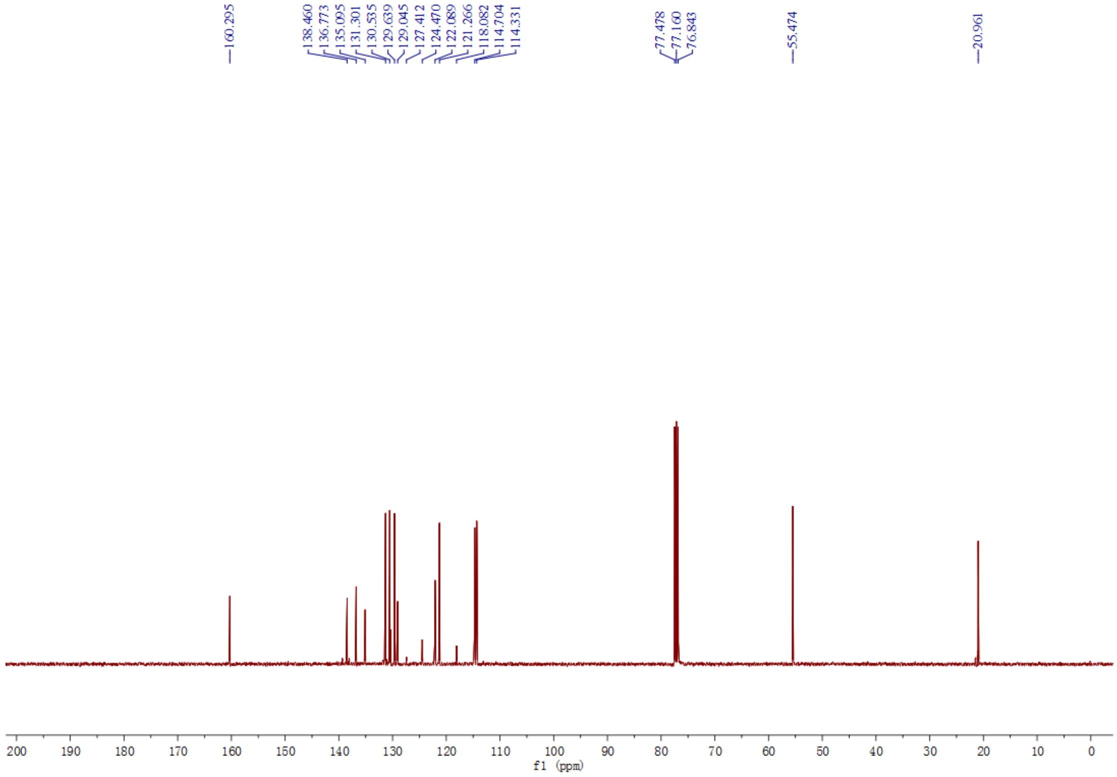

^{13}C NMR Spectrum for **3y** ($CDCl_3$, 100 MHz)

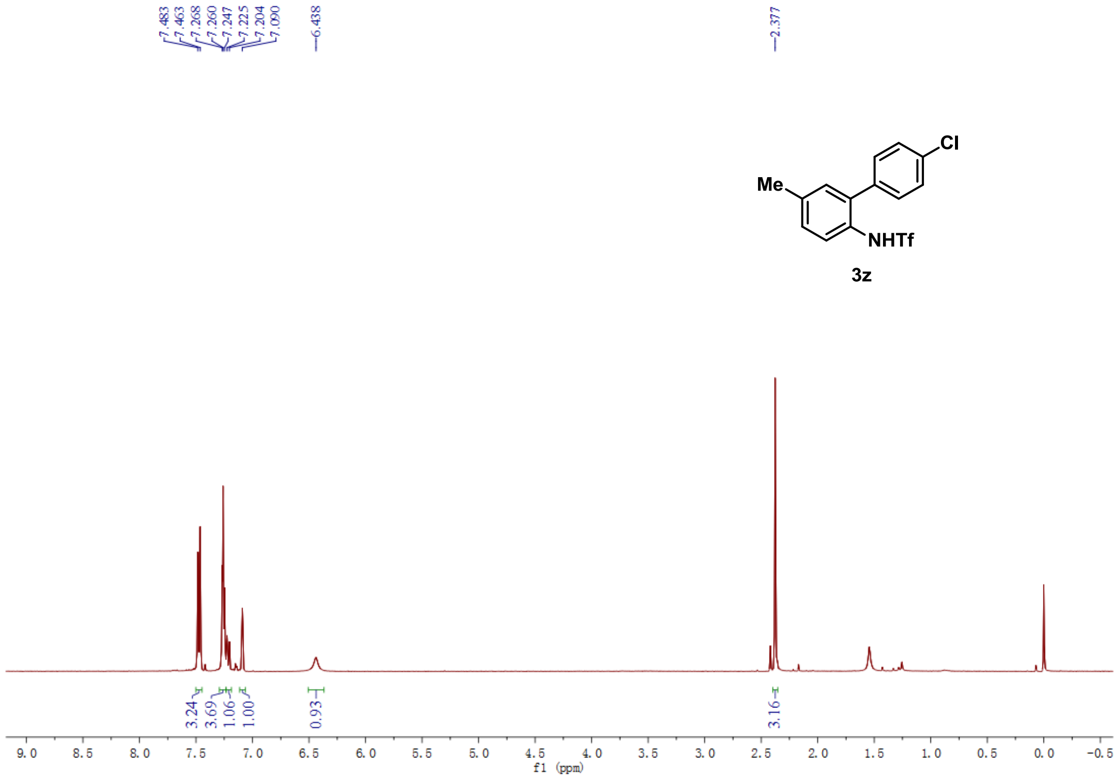

^{1}H NMR Spectrum for **3z** ($CDCl_3$, 400 MHz)

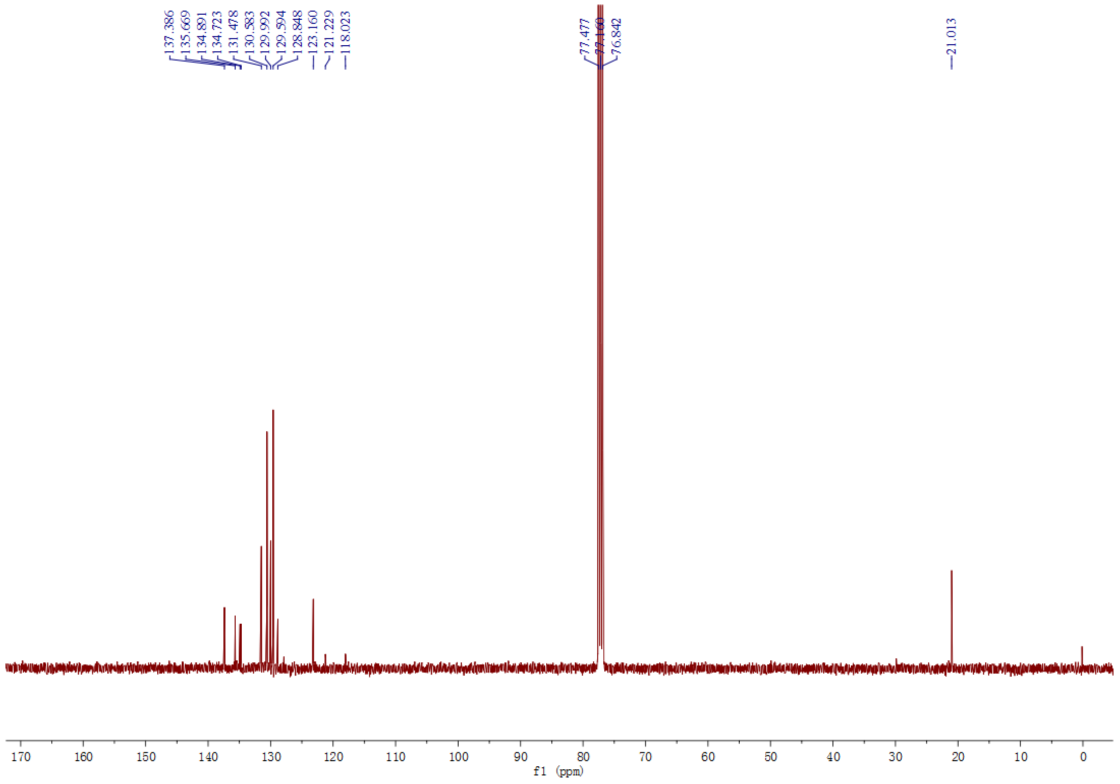

^{13}C NMR Spectrum for **3z** ($CDCl_3$, 100 MHz)

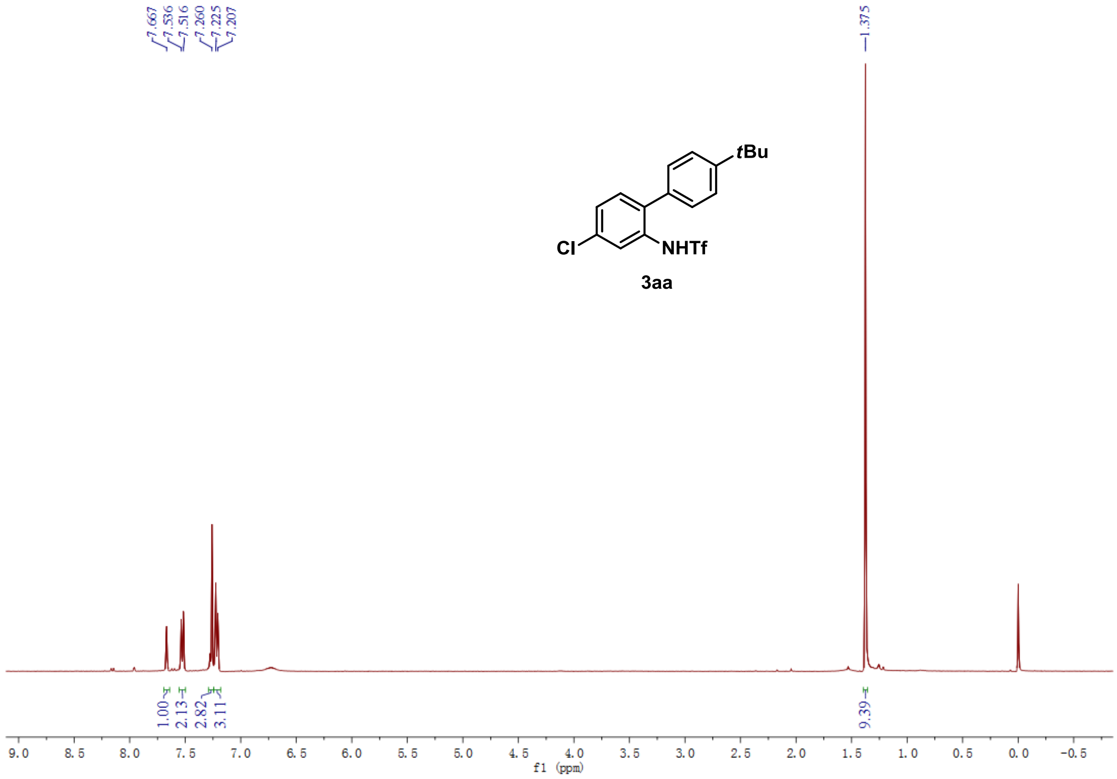

^{1}H NMR Spectrum for **3aa** ($CDCl_3$, 400 MHz)

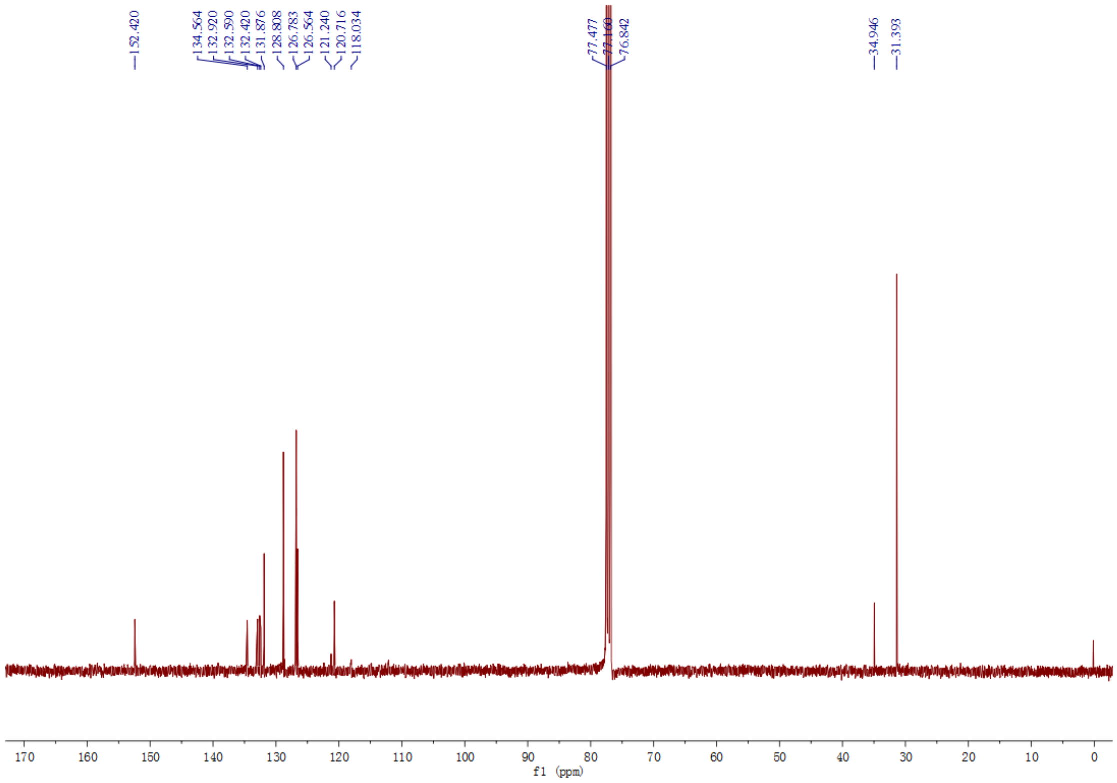

^{13}C NMR Spectrum for **3aa** ($CDCl_3$, 100 MHz)

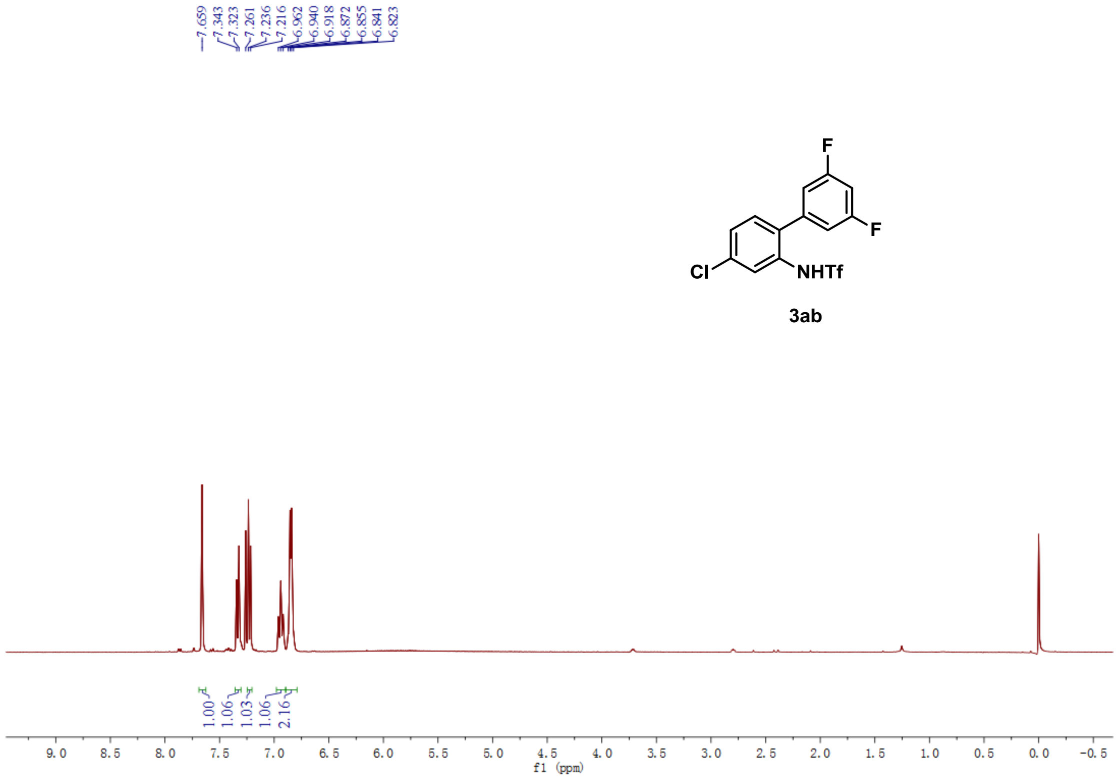

^{1}H NMR Spectrum for **3ab** ($CDCl_3$, 400 MHz)

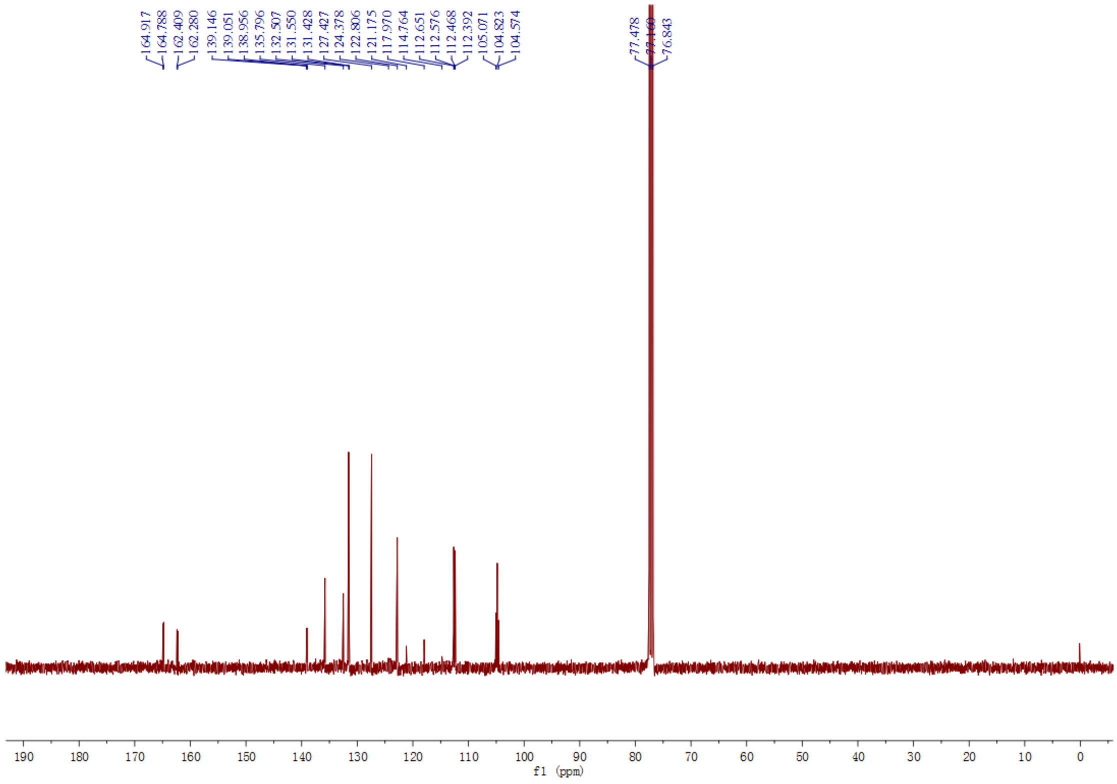

^{13}C NMR Spectrum for **3ab** ($CDCl_3$, 100 MHz)

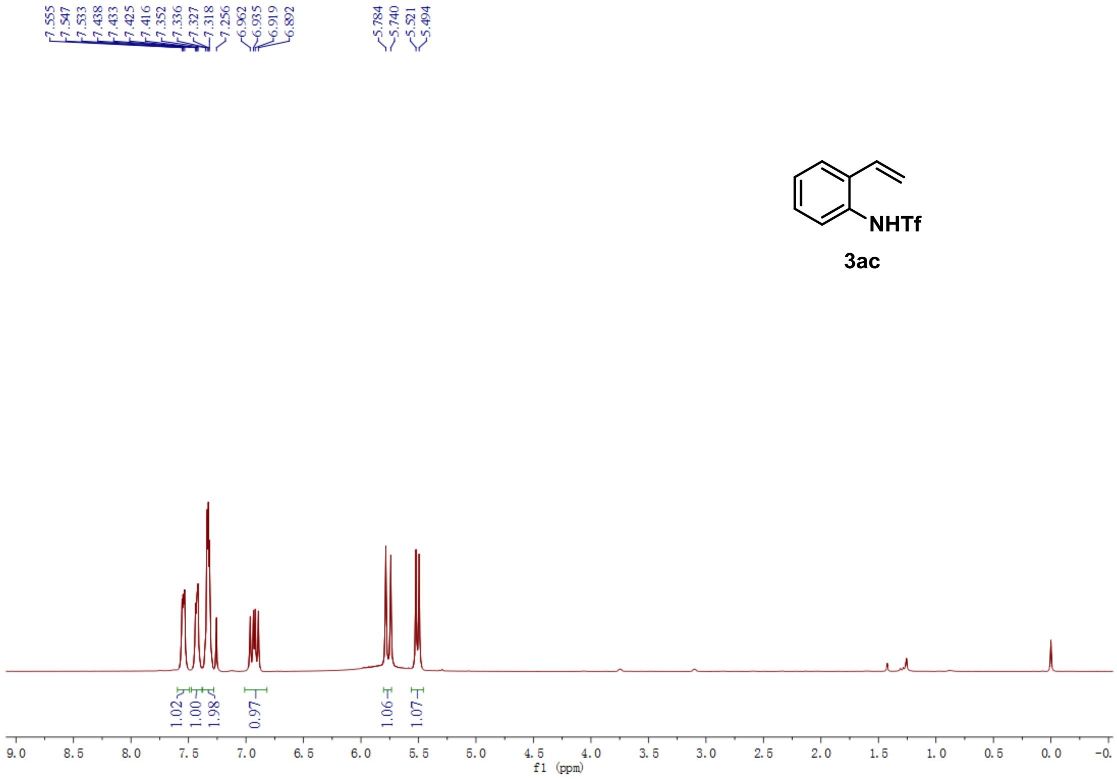

^{1}H NMR Spectrum for **3ac** ($CDCl_3$, 400 MHz)

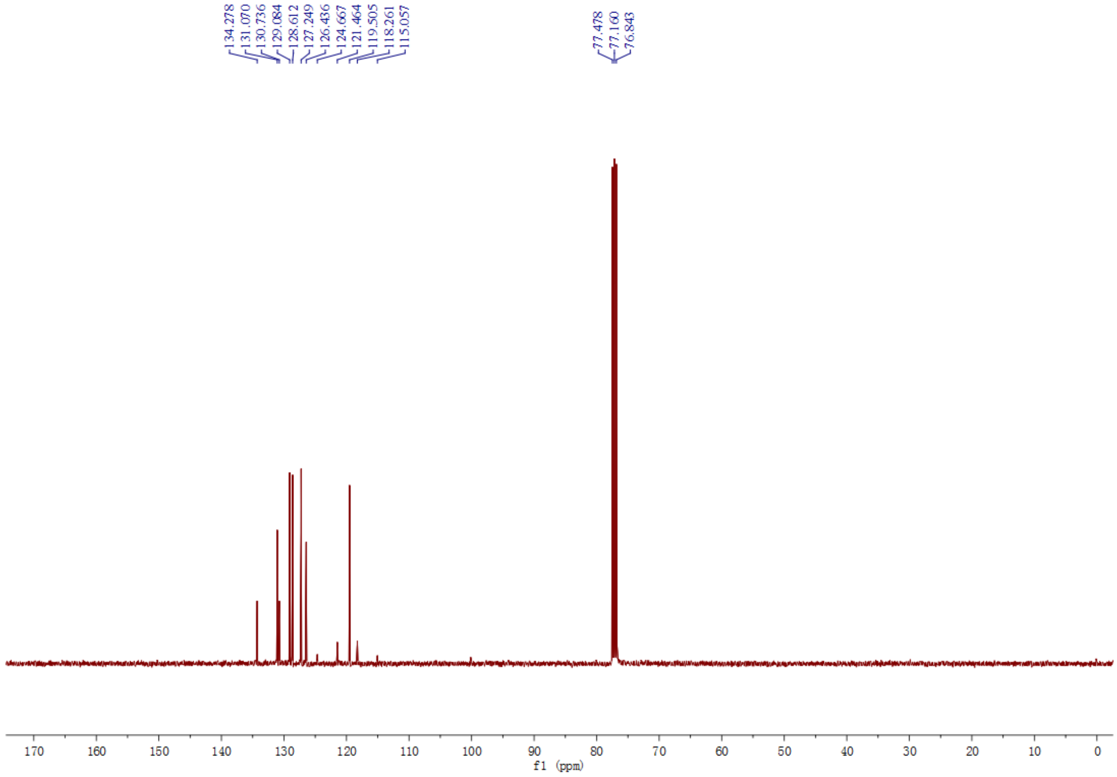

^{13}C NMR Spectrum for **3ac** ($CDCl_3$, 100 MHz)

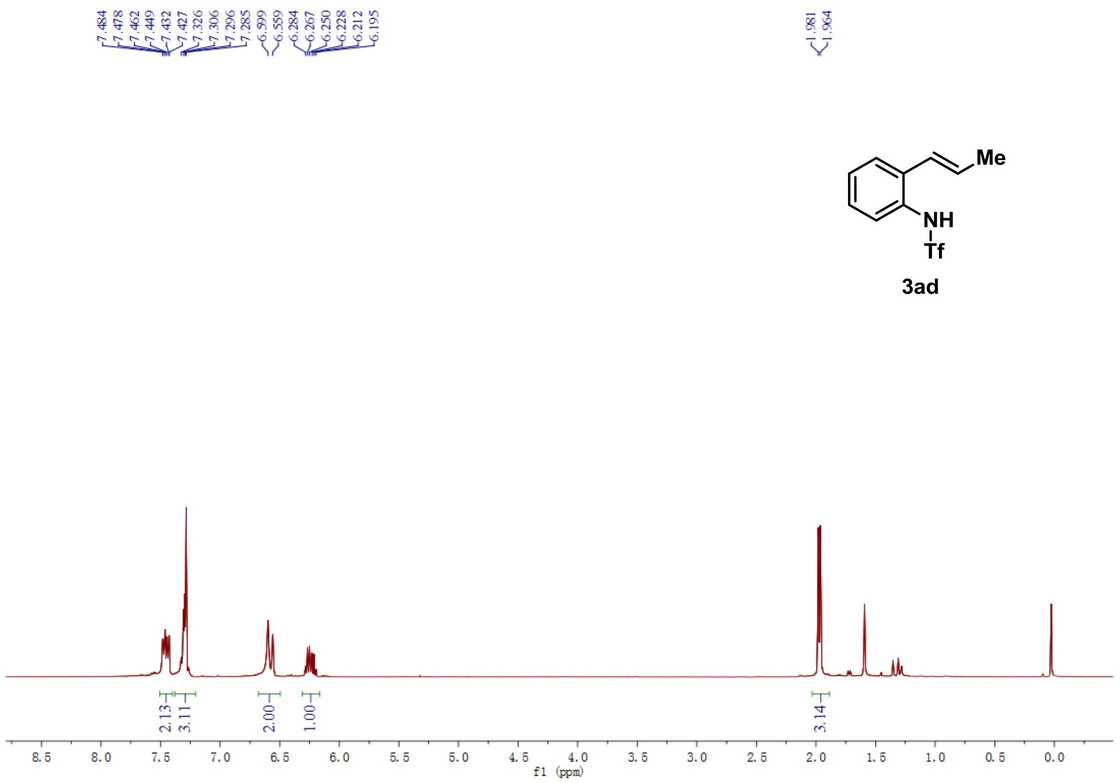

^{1}H NMR Spectrum for **3ad** ($CDCl_3$, 400 MHz)

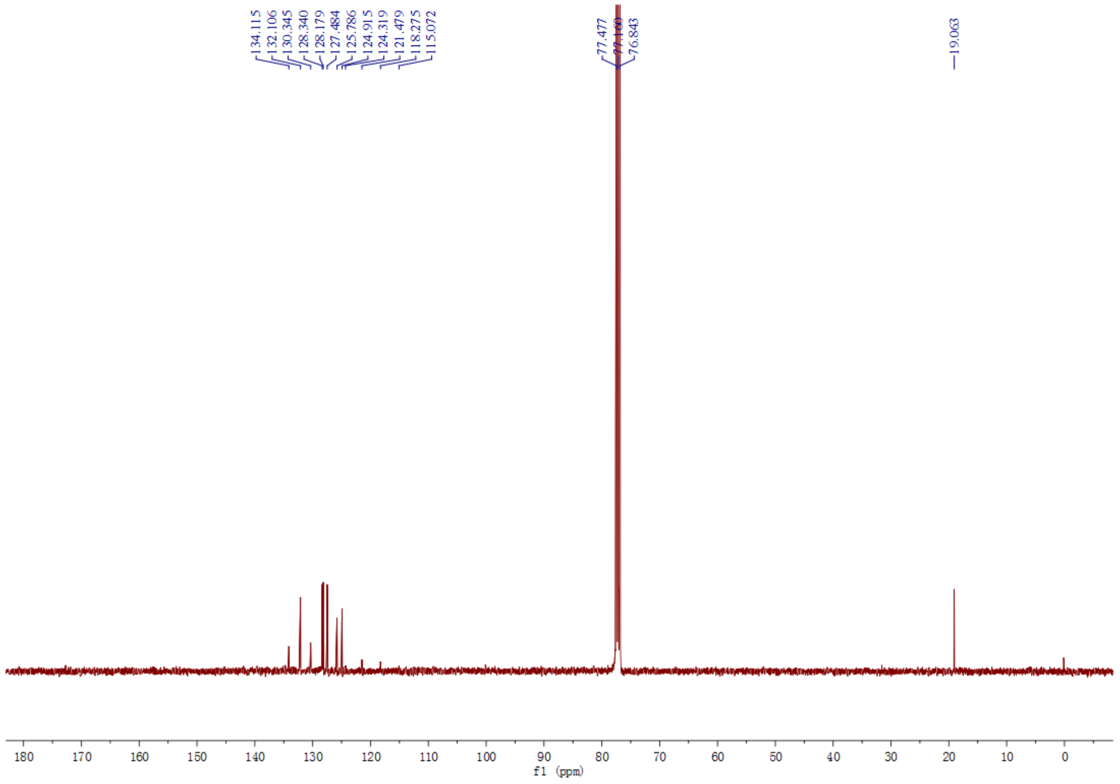

^{13}C NMR Spectrum for **3ad** ($CDCl_3$, 100 MHz)

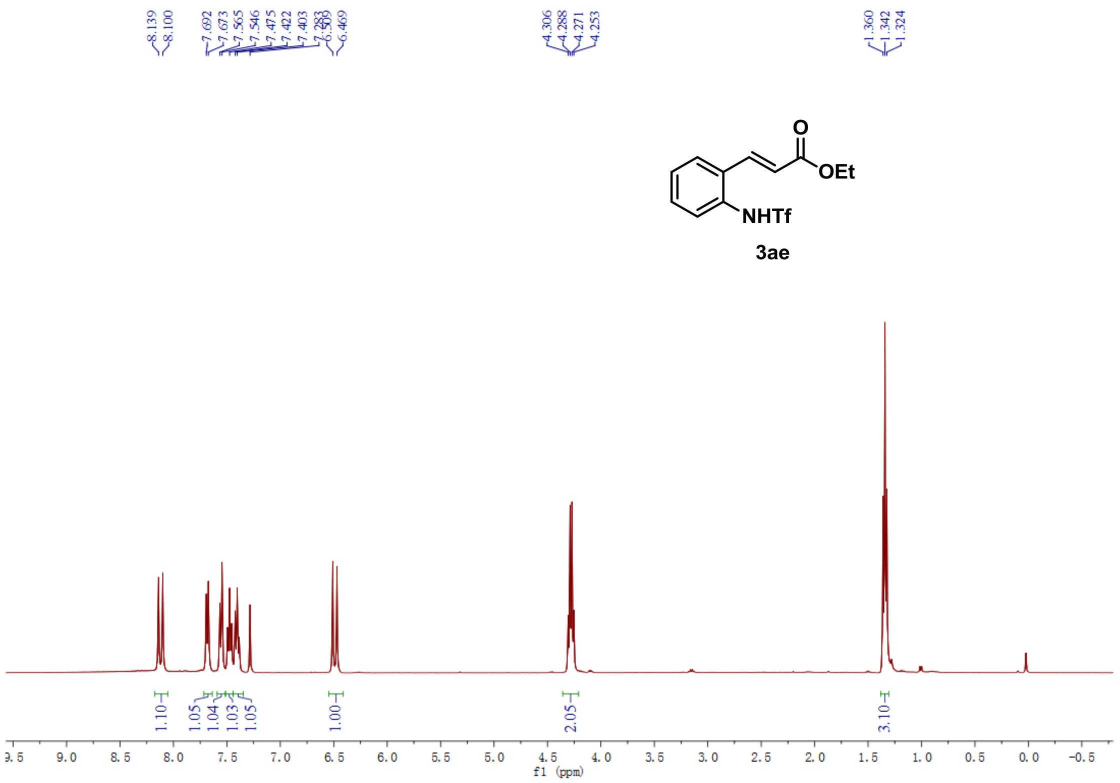

^{1}H NMR Spectrum for **3ae** ($CDCl_3$, 400 MHz)

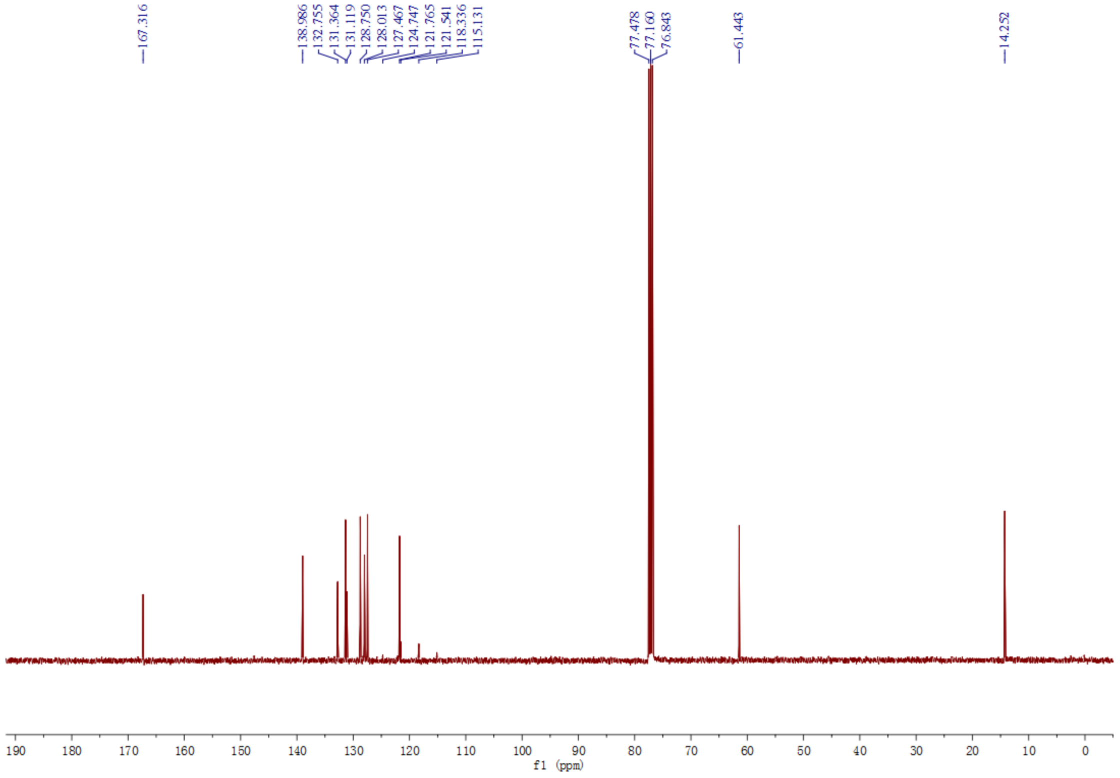

^{13}C NMR Spectrum for **3ae** ($CDCl_3$, 100 MHz)

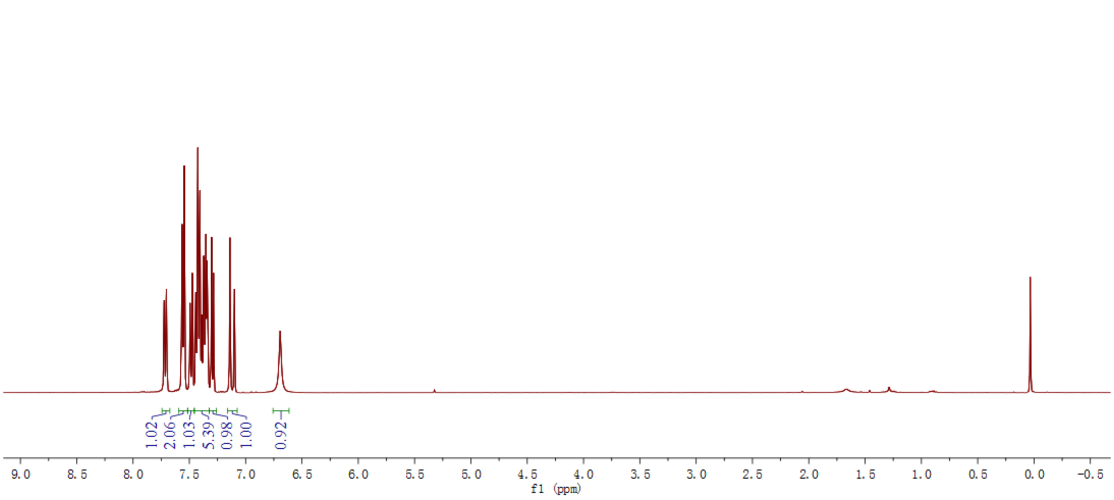

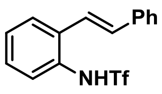

3af

^{1}H NMR Spectrum for **3af** ($CDCl_3$, 400 MHz)

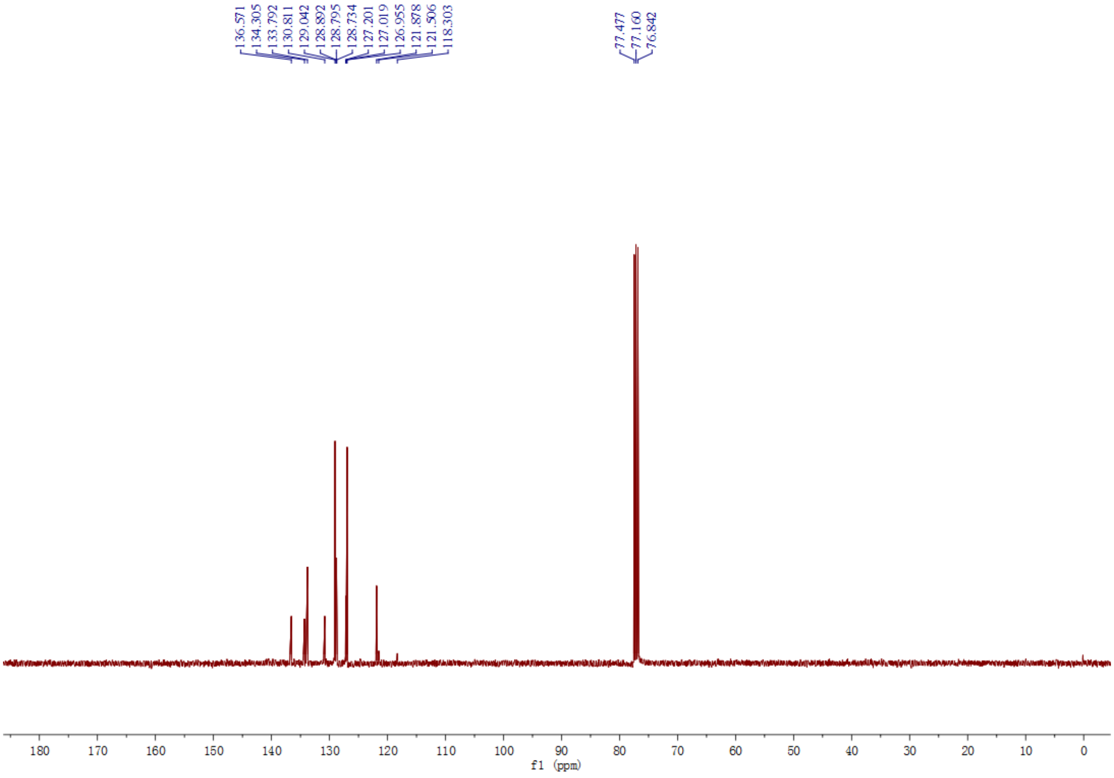

^{13}C NMR Spectrum for **3af** ($CDCl_3$, 100 MHz)

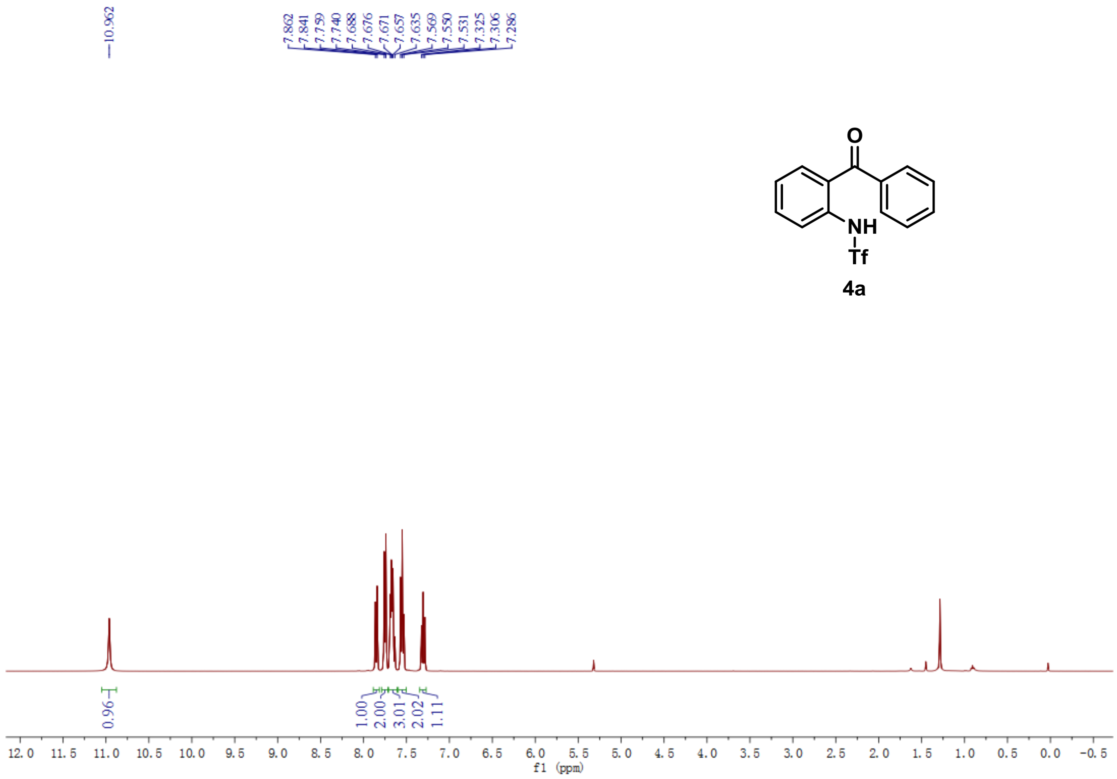

4a

^{1}H NMR Spectrum for **4a** ($CDCl_3$, 400 MHz)

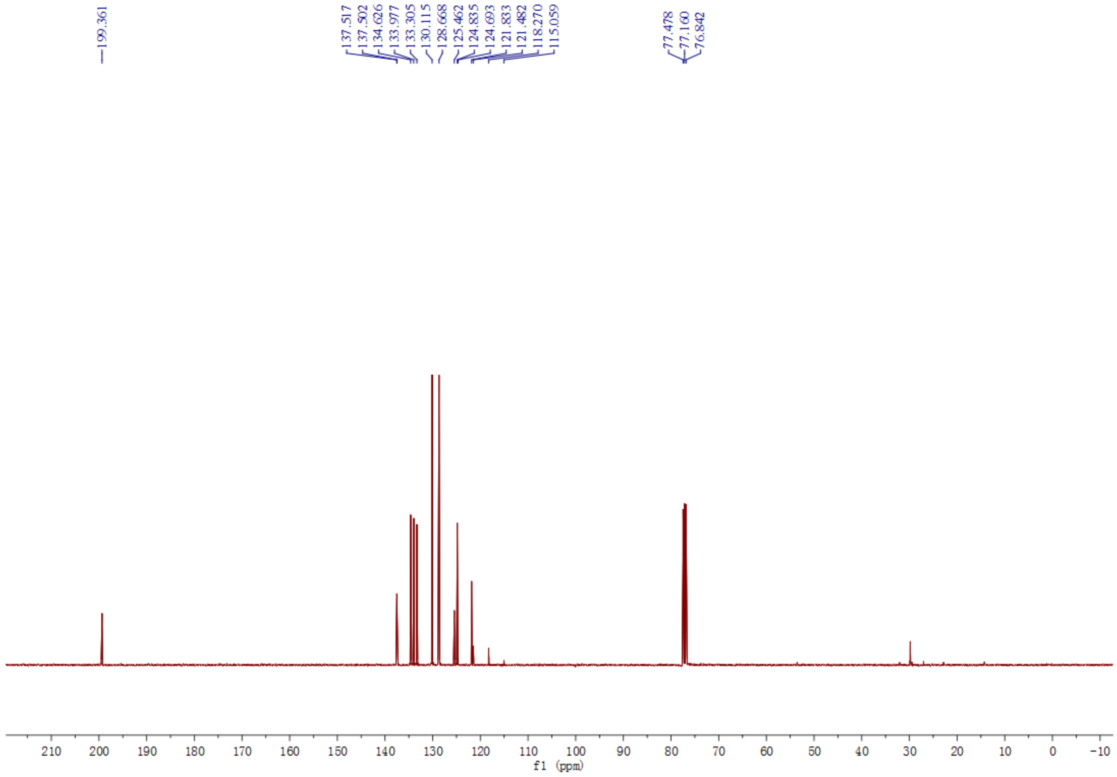

^{13}C NMR Spectrum for **4a** ($CDCl_3$, 100 MHz)

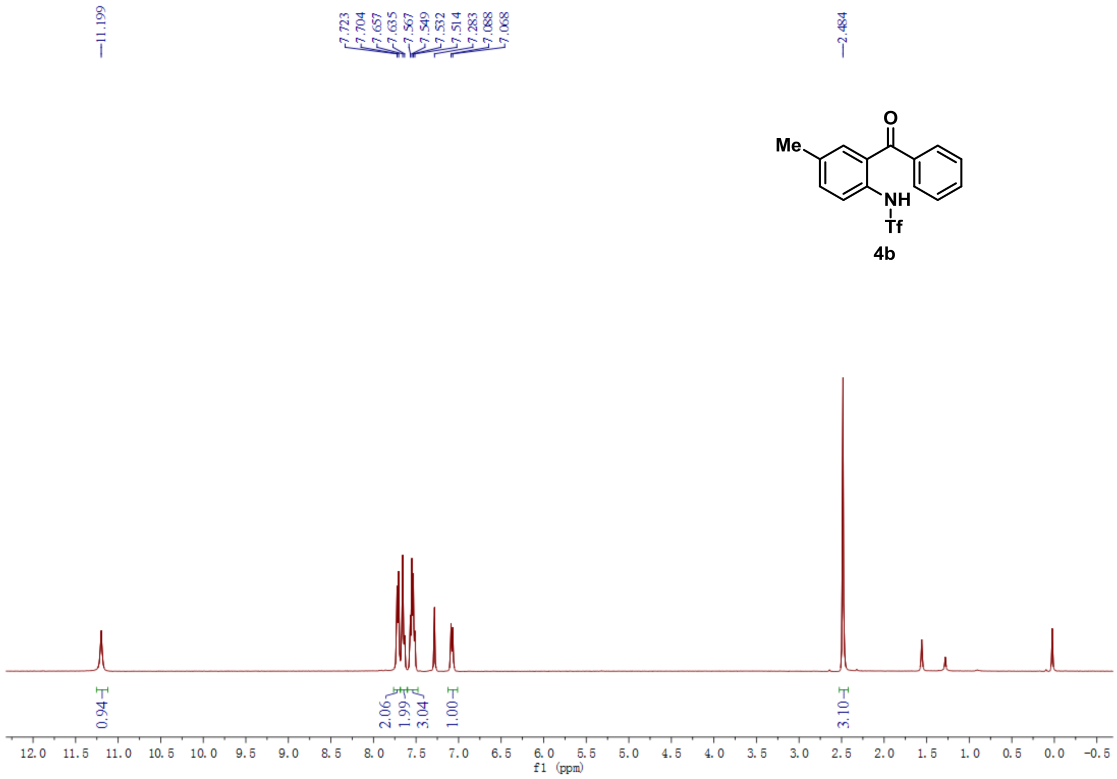

^{1}H NMR Spectrum for **4b** ($CDCl_3$, 400 MHz)

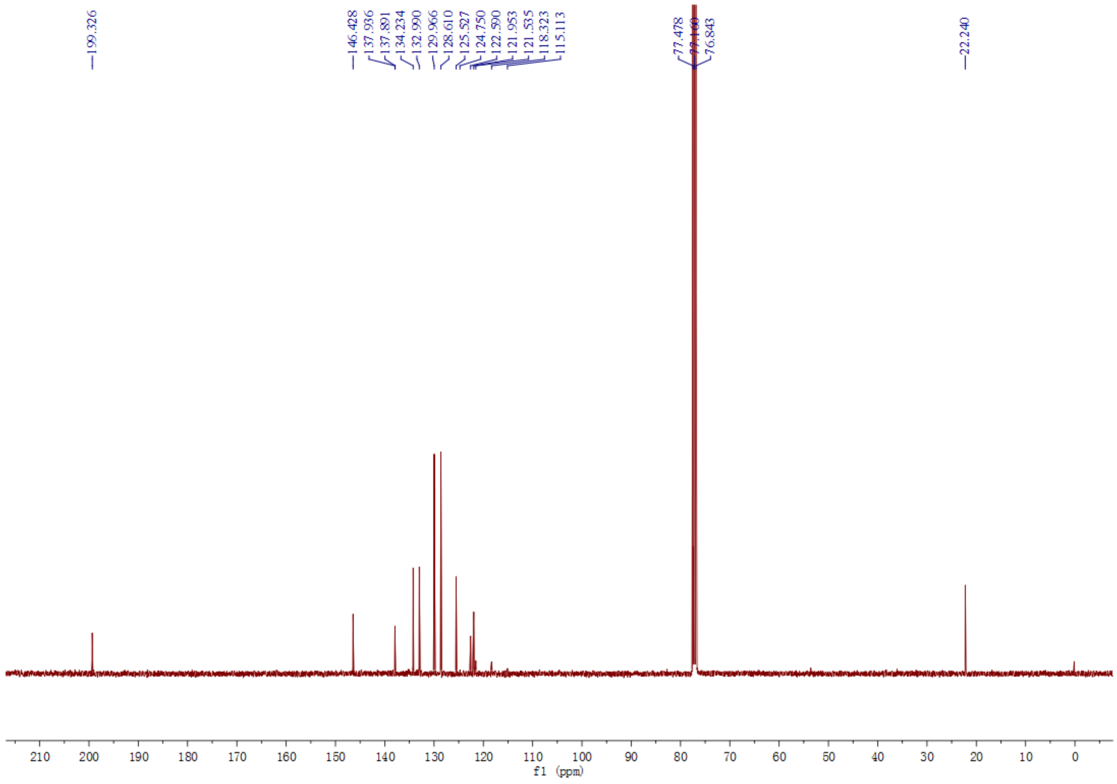

^{13}C NMR Spectrum for **4b** ($CDCl_3$, 100 MHz)

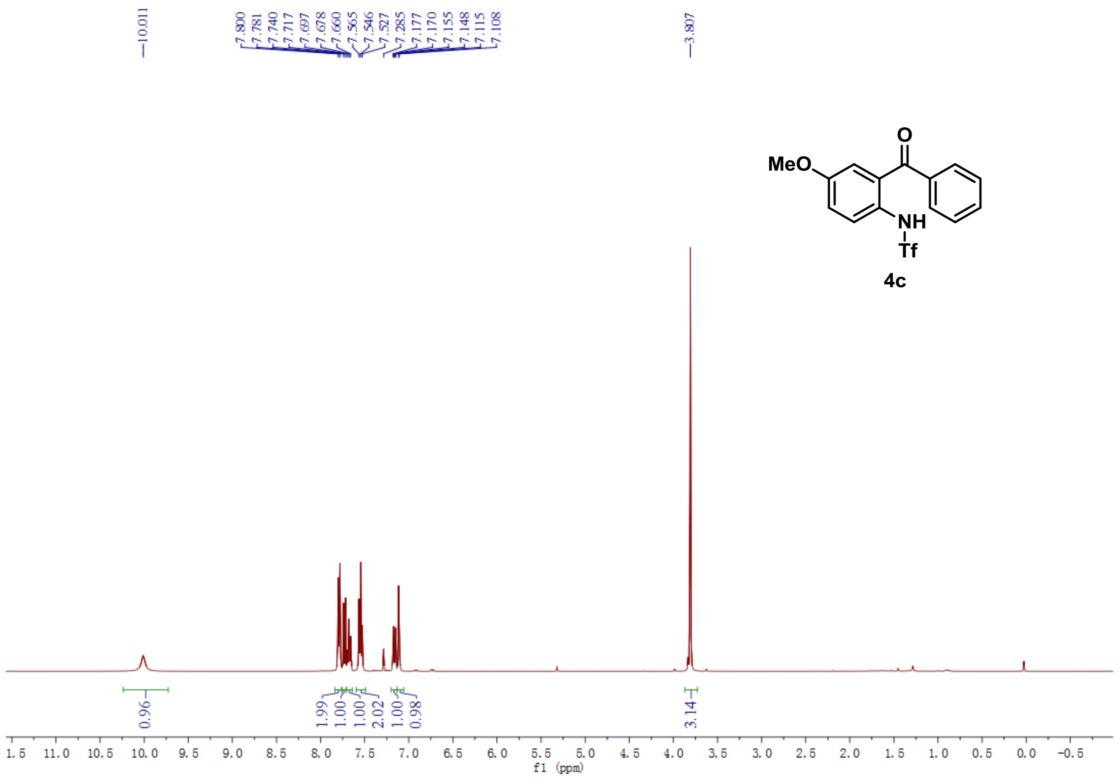

^{1}H NMR Spectrum for **4c** ($CDCl_3$, 400 MHz)

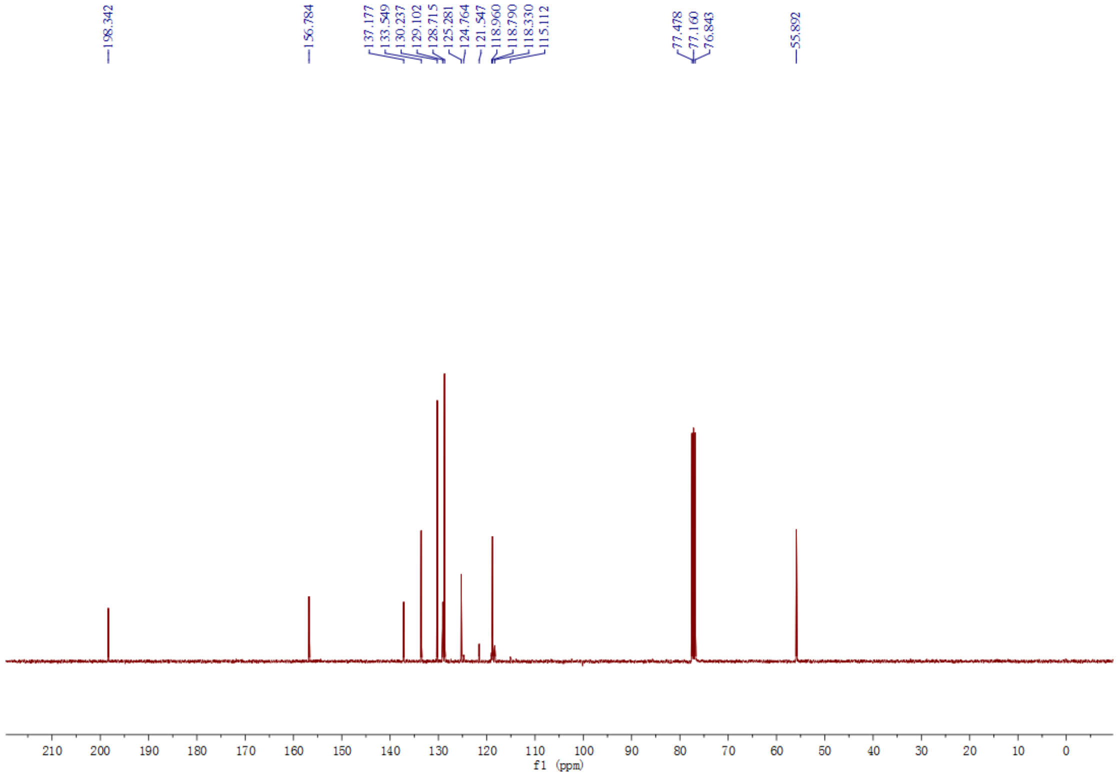

^{13}C NMR Spectrum for **4c** ($CDCl_3$, 100 MHz)

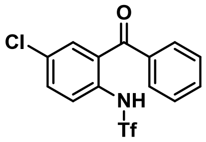

4d

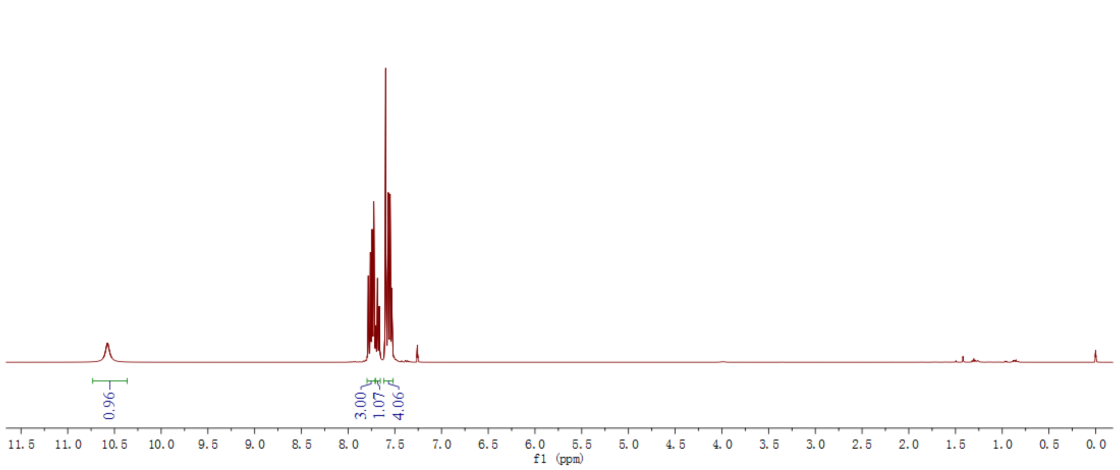

^{1}H NMR Spectrum for **4d** ($CDCl_3$, 400 MHz)

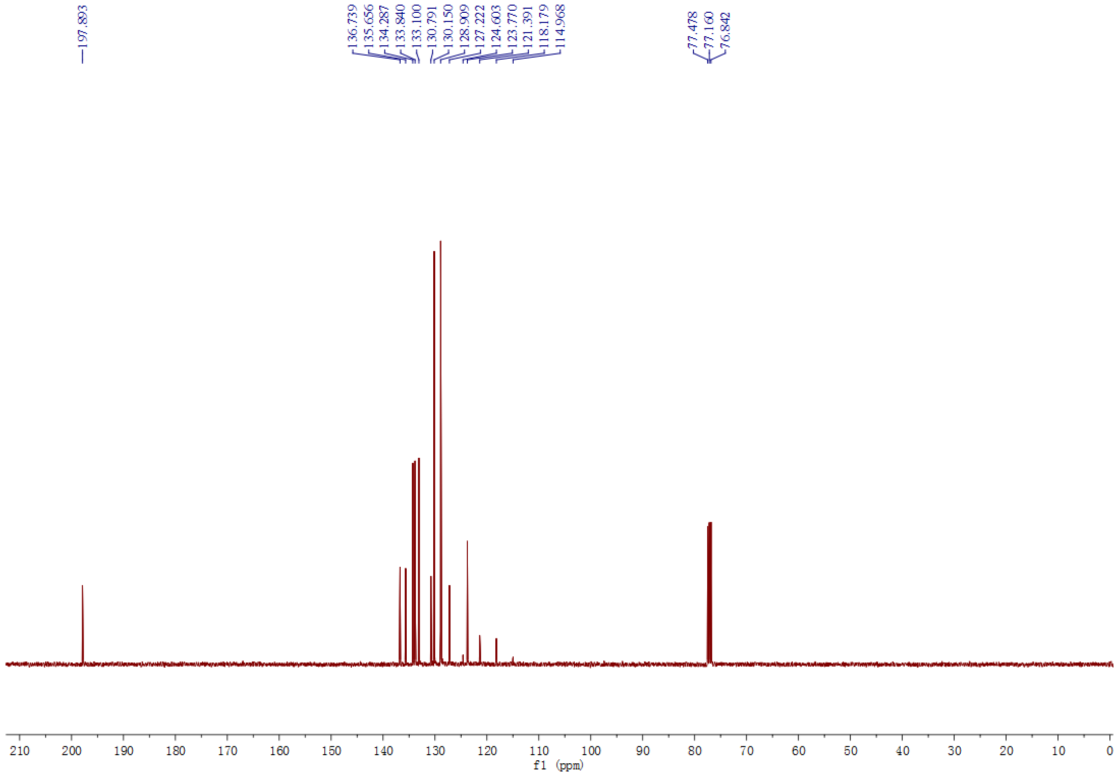

^{13}C NMR Spectrum for **4d** ($CDCl_3$, 100 MHz)

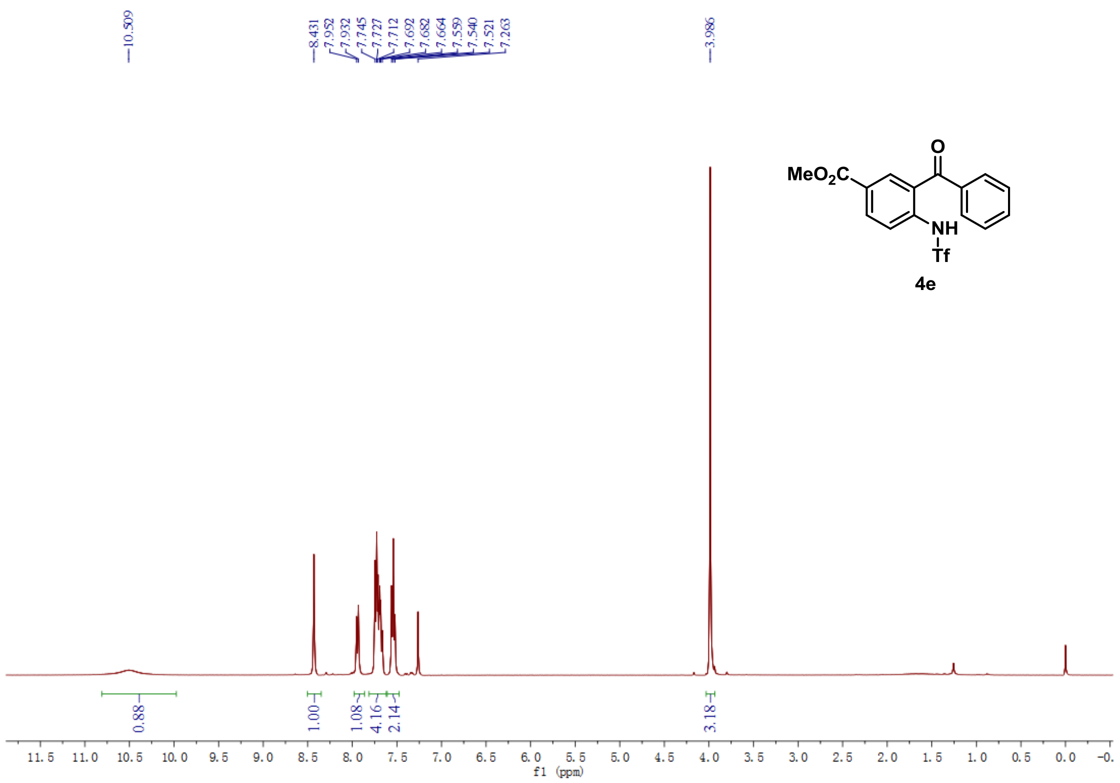

^{1}H NMR Spectrum for **4e** ($CDCl_3$, 400 MHz)

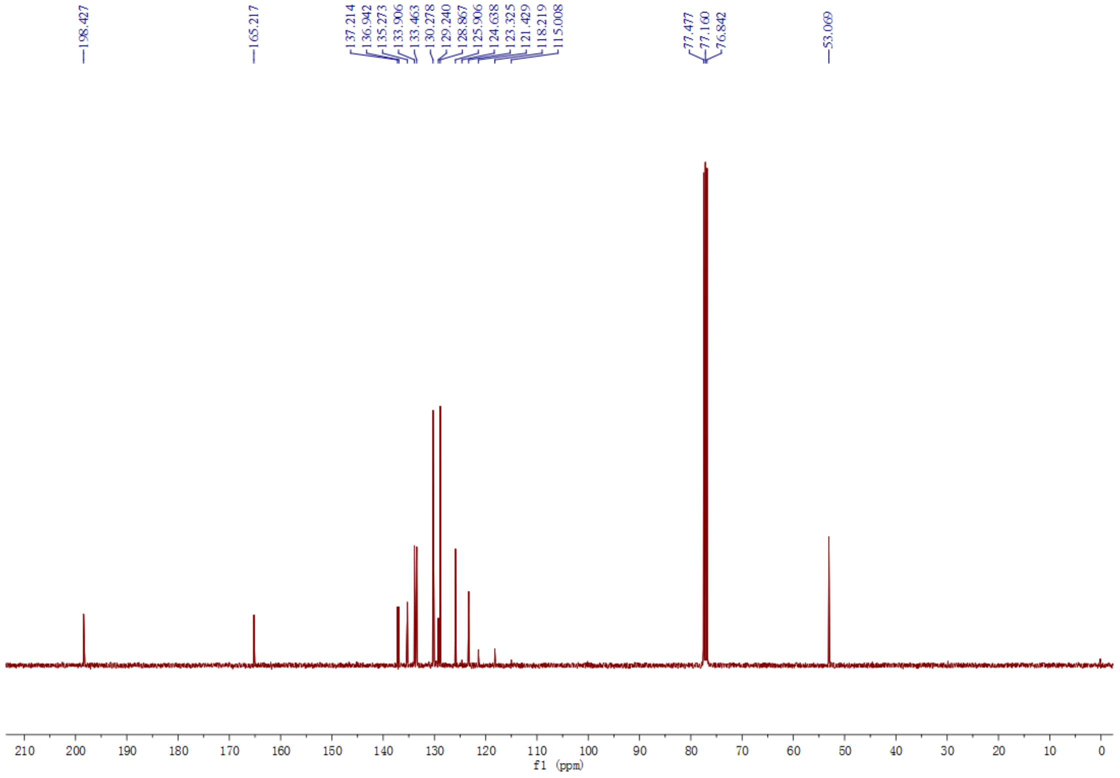

^{13}C NMR Spectrum for **4e** ($CDCl_3$, 100 MHz)

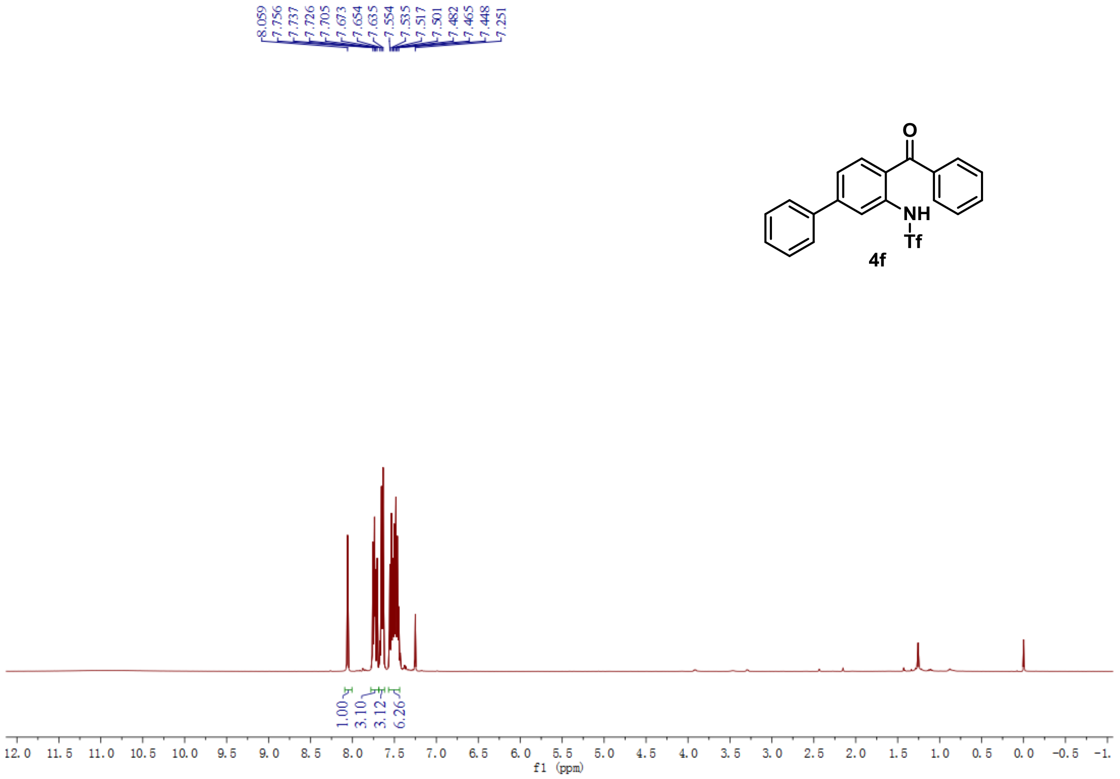

^{1}H NMR Spectrum for **4f** ($CDCl_3$, 400 MHz)

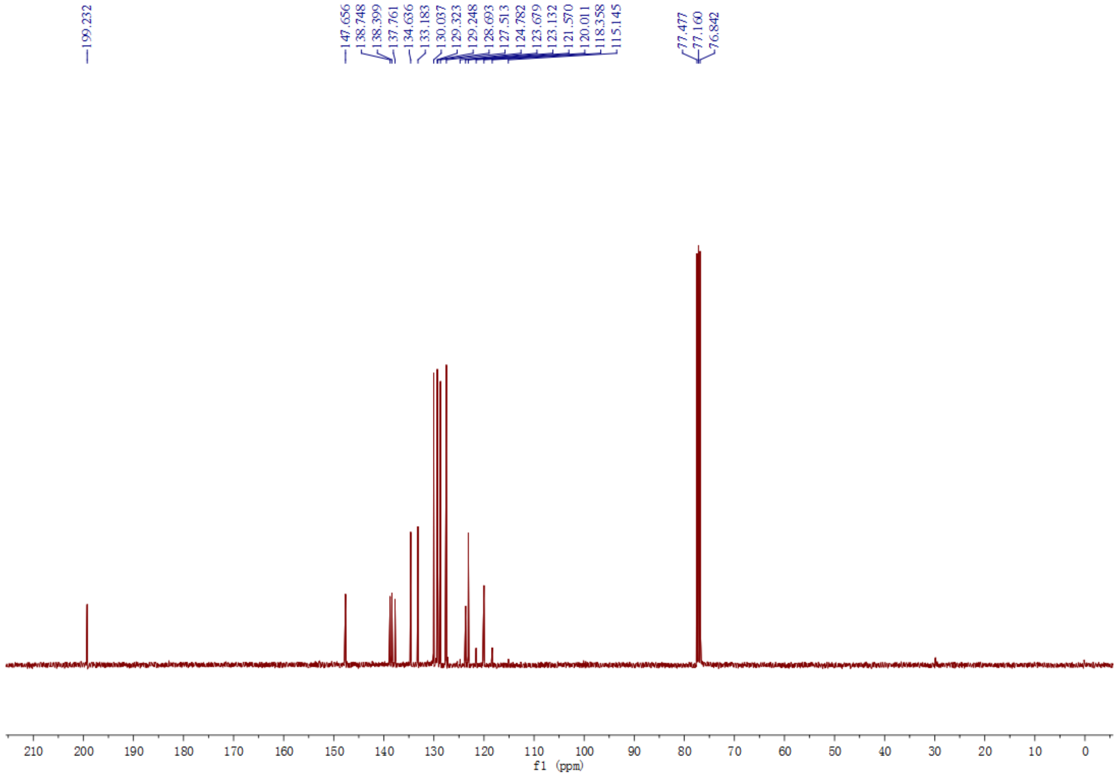

^{13}C NMR Spectrum for **4f** ($CDCl_3$, 100 MHz)

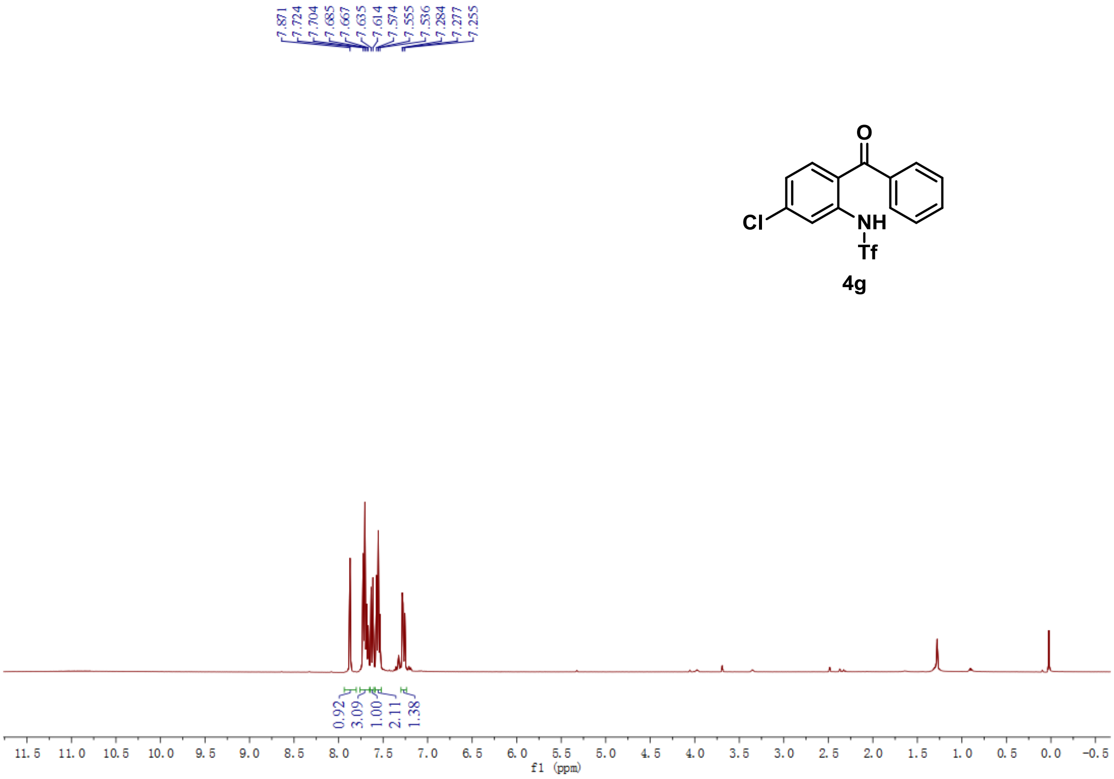

^{1}H NMR Spectrum for **4g** ($CDCl_3$, 400 MHz)

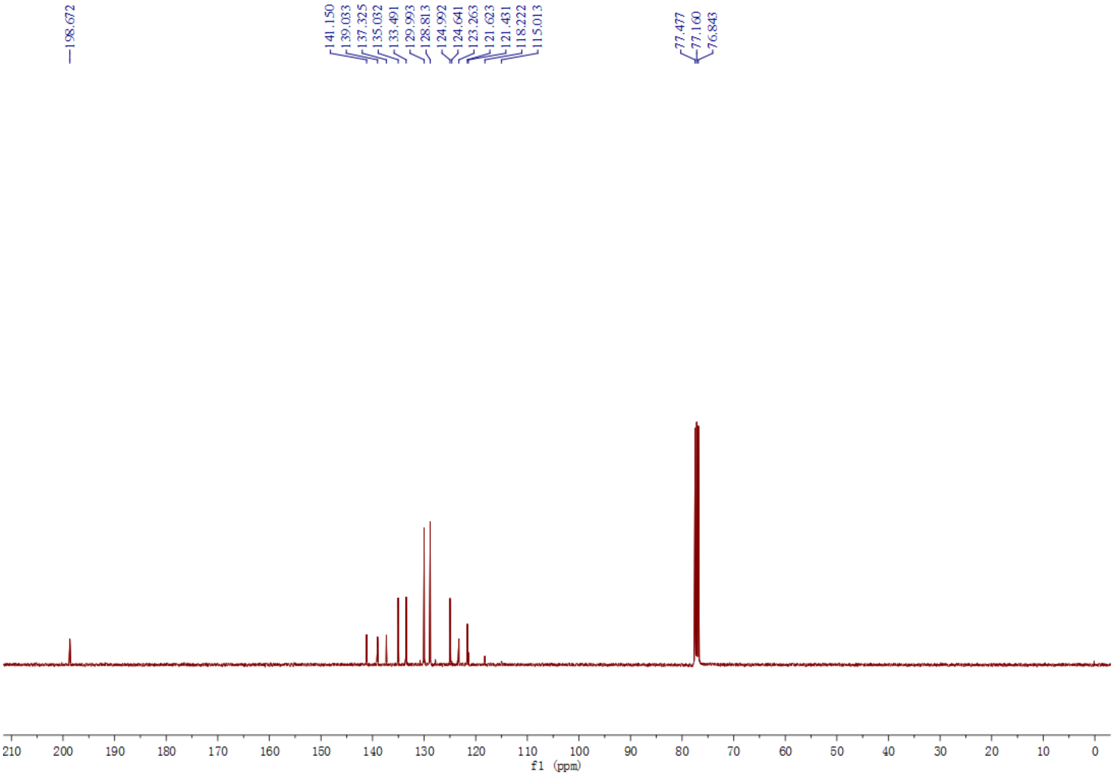

^{13}C NMR Spectrum for **4g** ($CDCl_3$, 100 MHz)

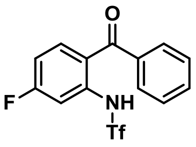

4h

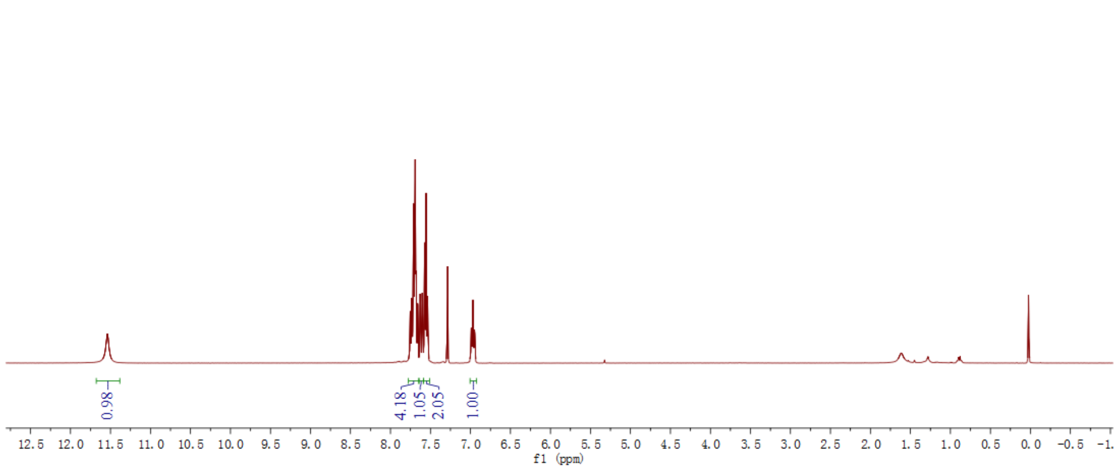

^{1}H NMR Spectrum for **4h** ($CDCl_3$, 400 MHz)

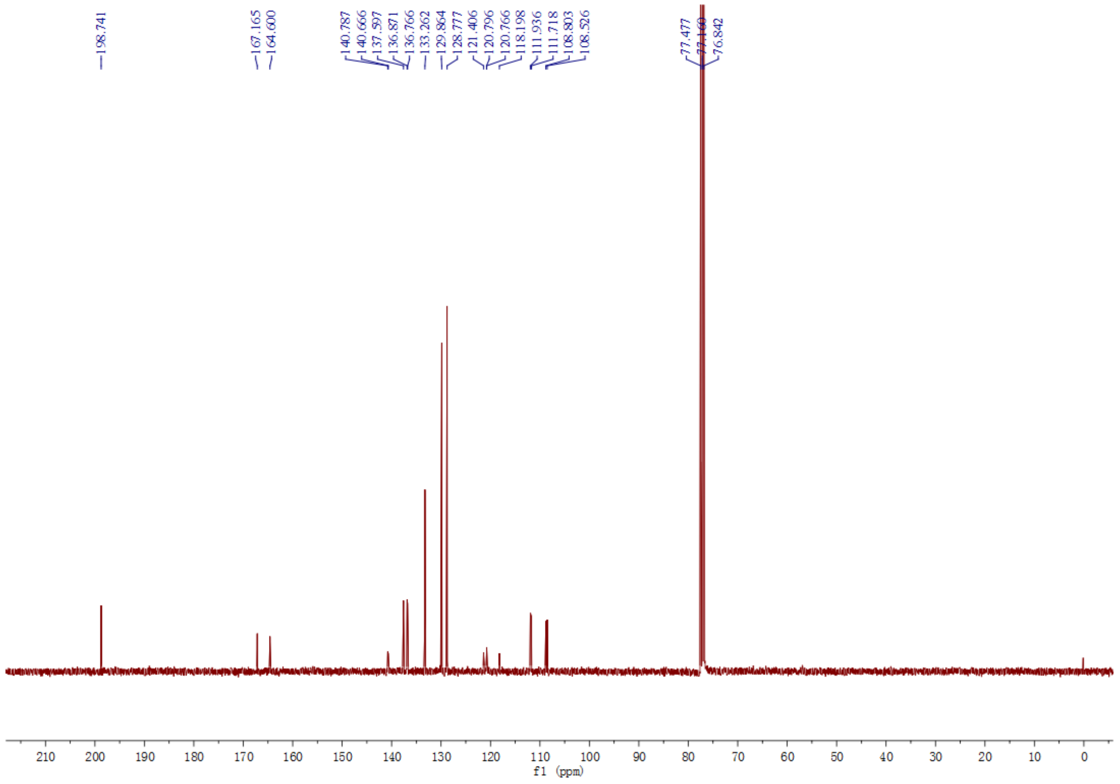

^{13}C NMR Spectrum for **4h** ($CDCl_3$, 100 MHz)

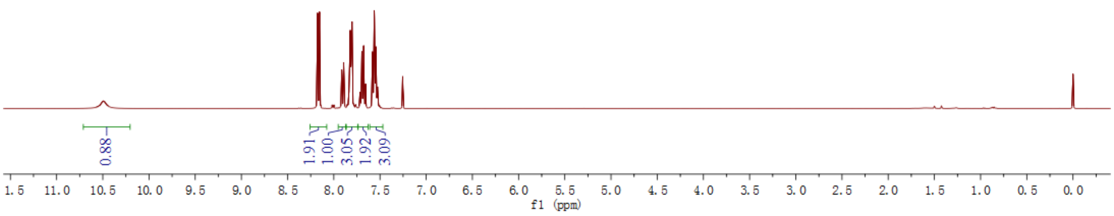

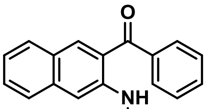

4i

^{1}H NMR Spectrum for **4i** ($CDCl_3$, 400 MHz)

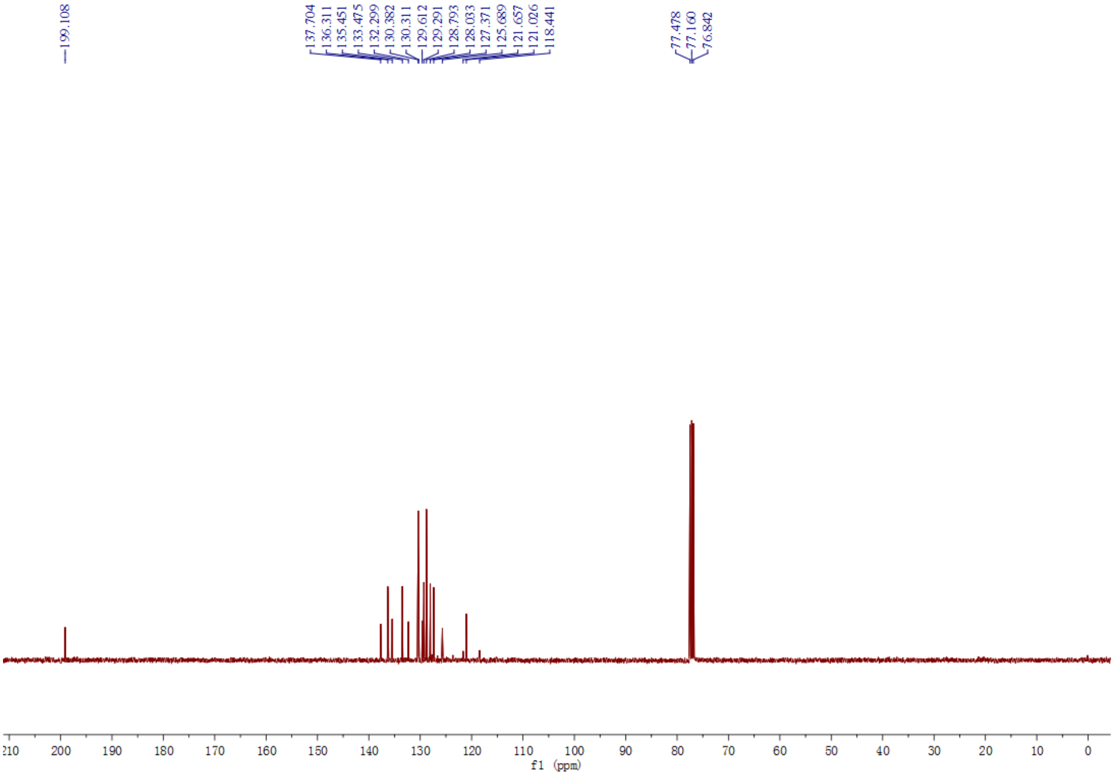

^{13}C NMR Spectrum for **4i** ($CDCl_3$, 100 MHz)

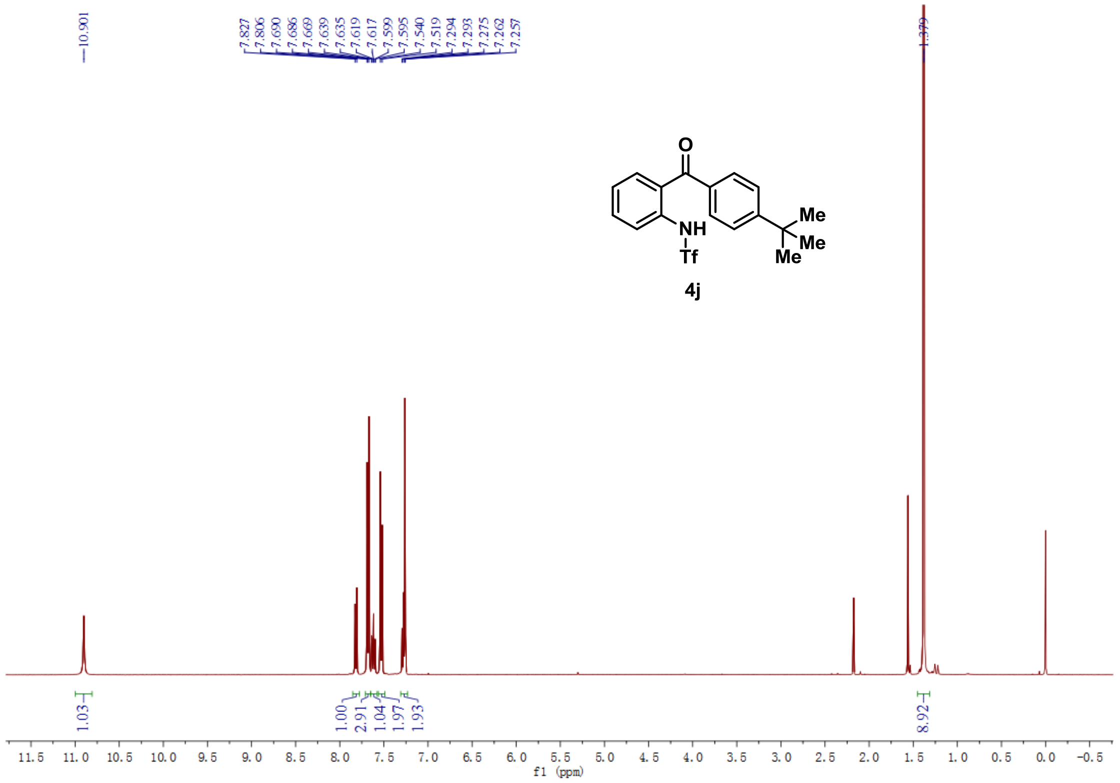

^{1}H NMR Spectrum for **4j** ($CDCl_3$, 400 MHz)

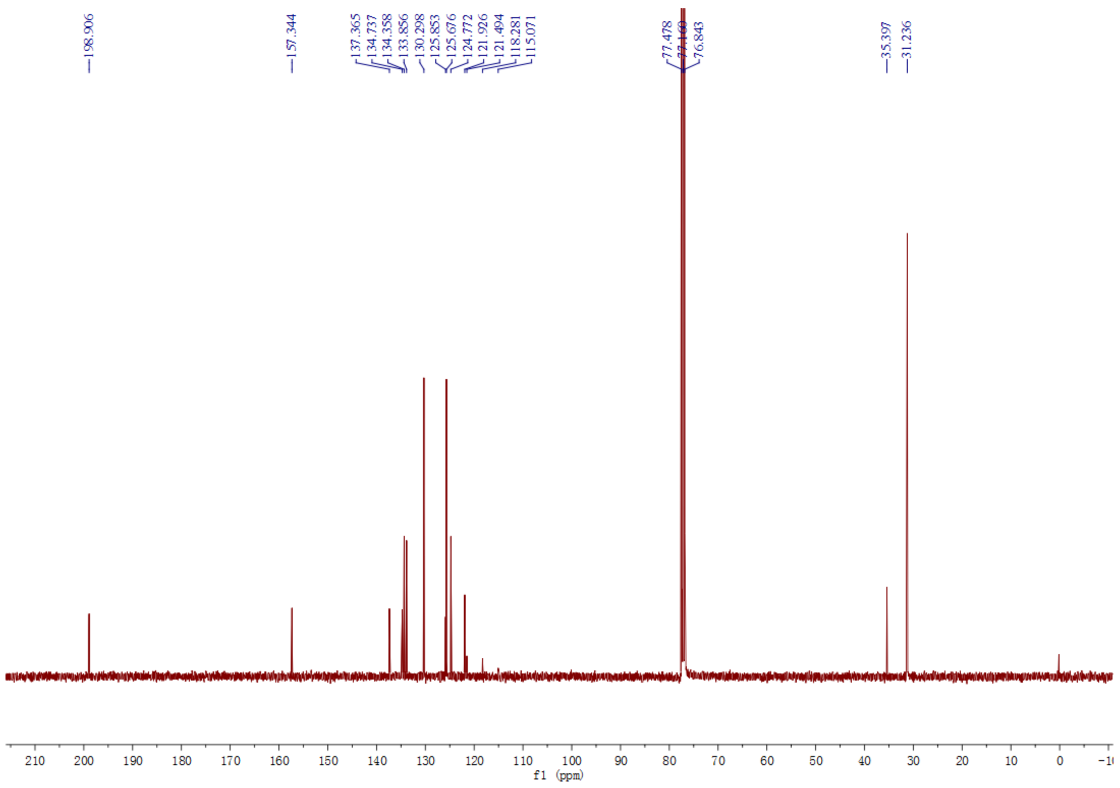

^{13}C NMR Spectrum for **4j** ($CDCl_3$, 100 MHz)

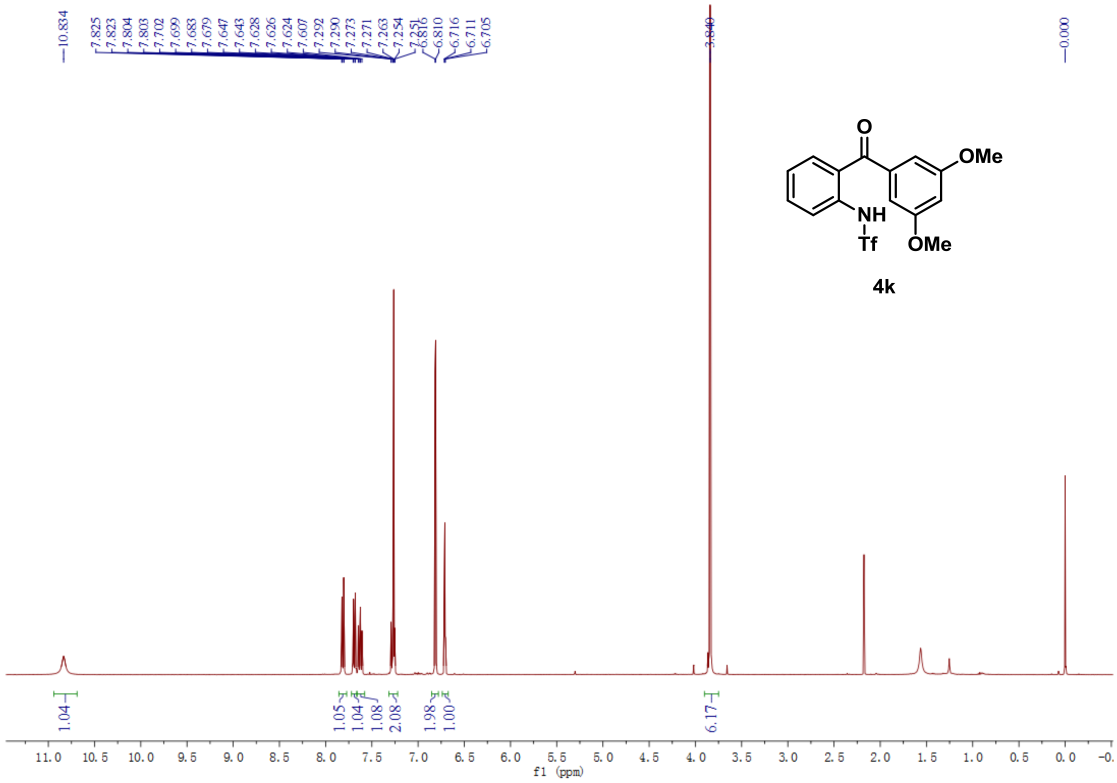

^{1}H NMR Spectrum for **4k** ($CDCl_3$, 400 MHz)

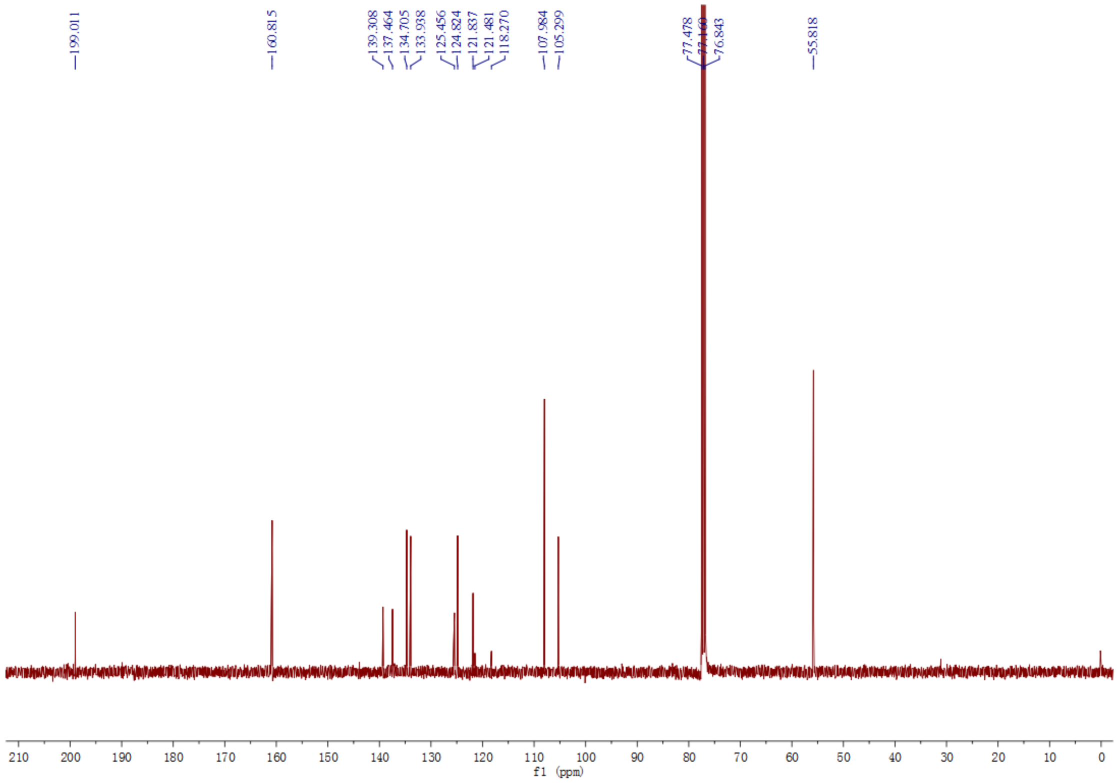

^{13}C NMR Spectrum for **4k** ($CDCl_3$, 100 MHz)

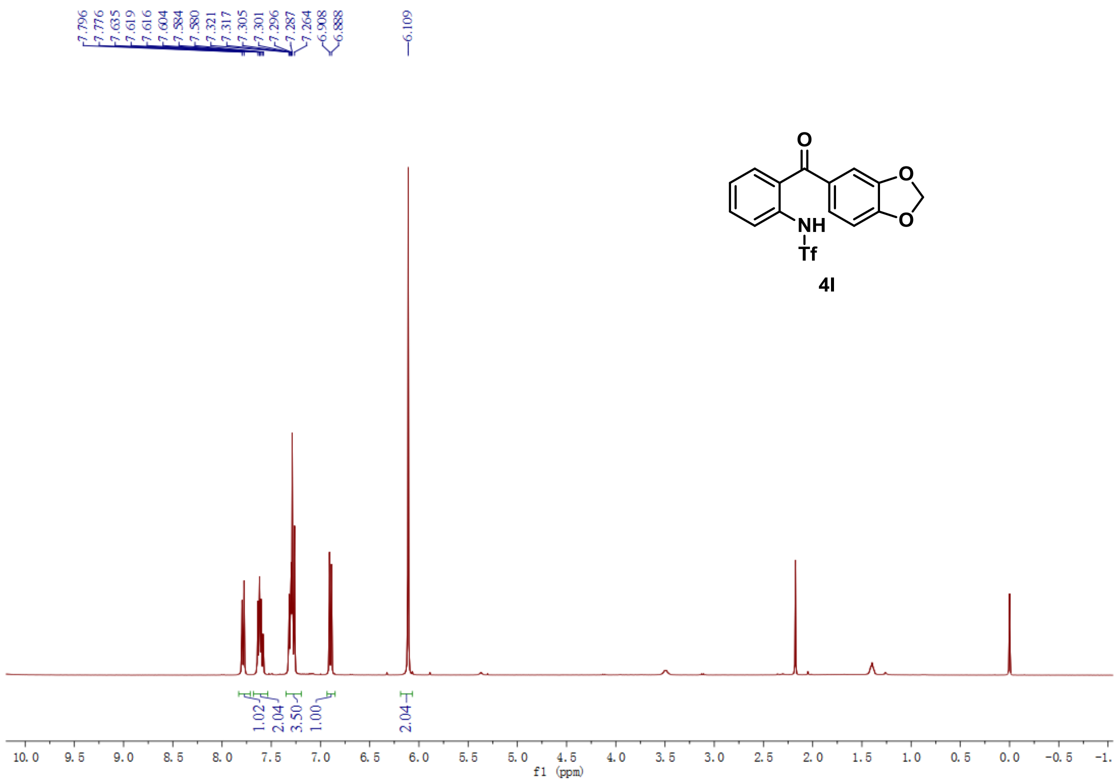

^{1}H NMR Spectrum for **4l** ($CDCl_3$, 400 MHz)

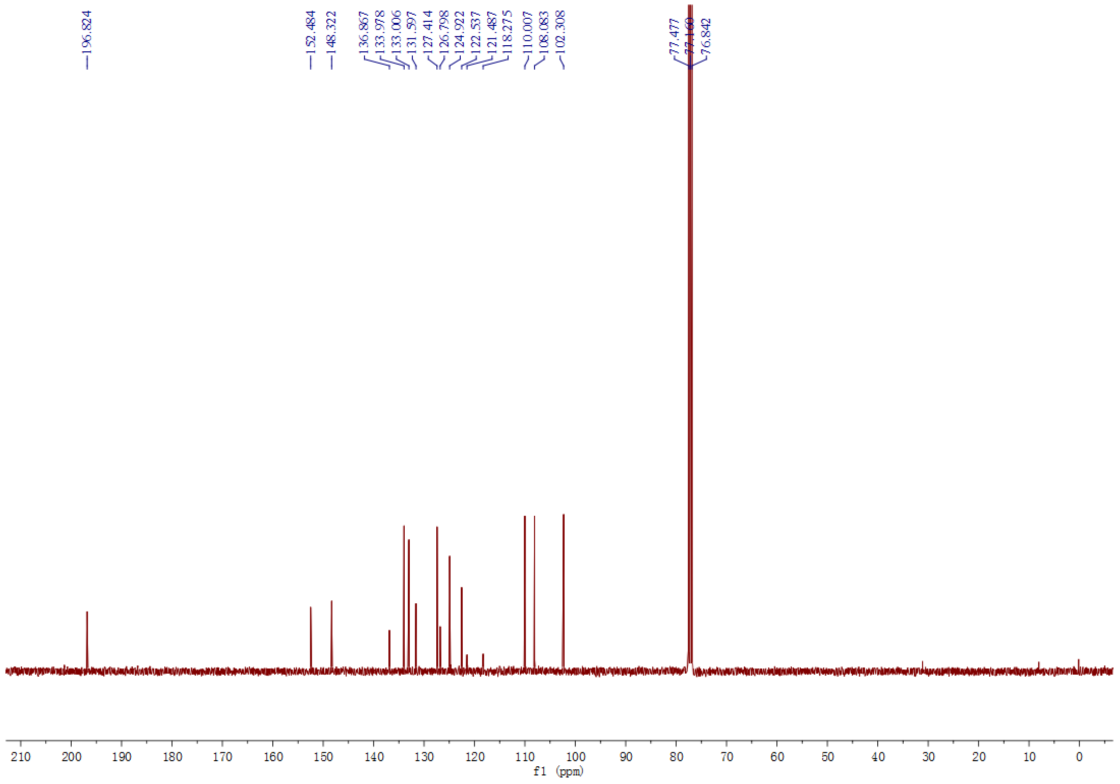

^{13}C NMR Spectrum for **4l** ($CDCl_3$, 100 MHz)

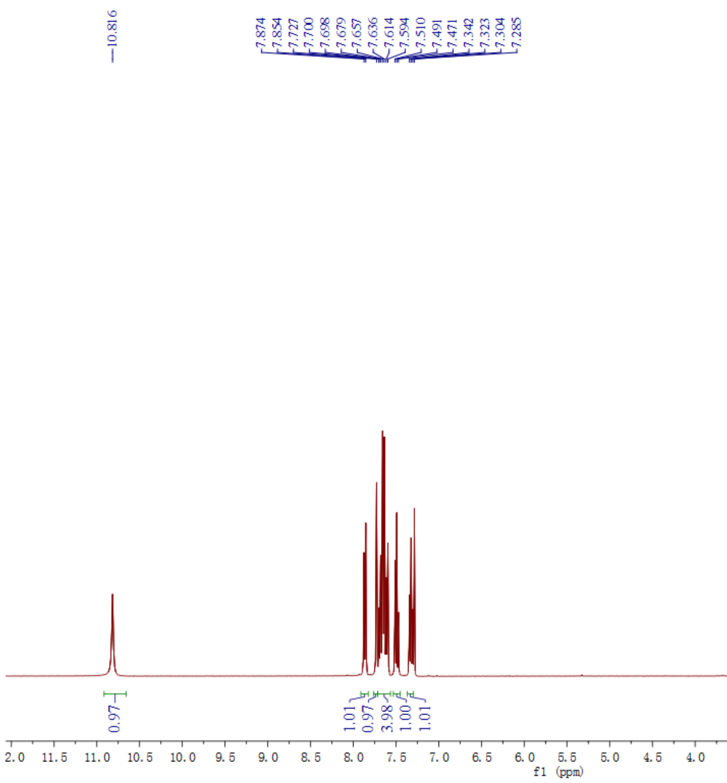

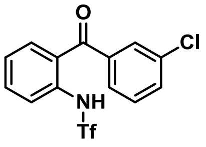

4m

^{1}H NMR Spectrum for **4m** ($CDCl_3$, 400 MHz)

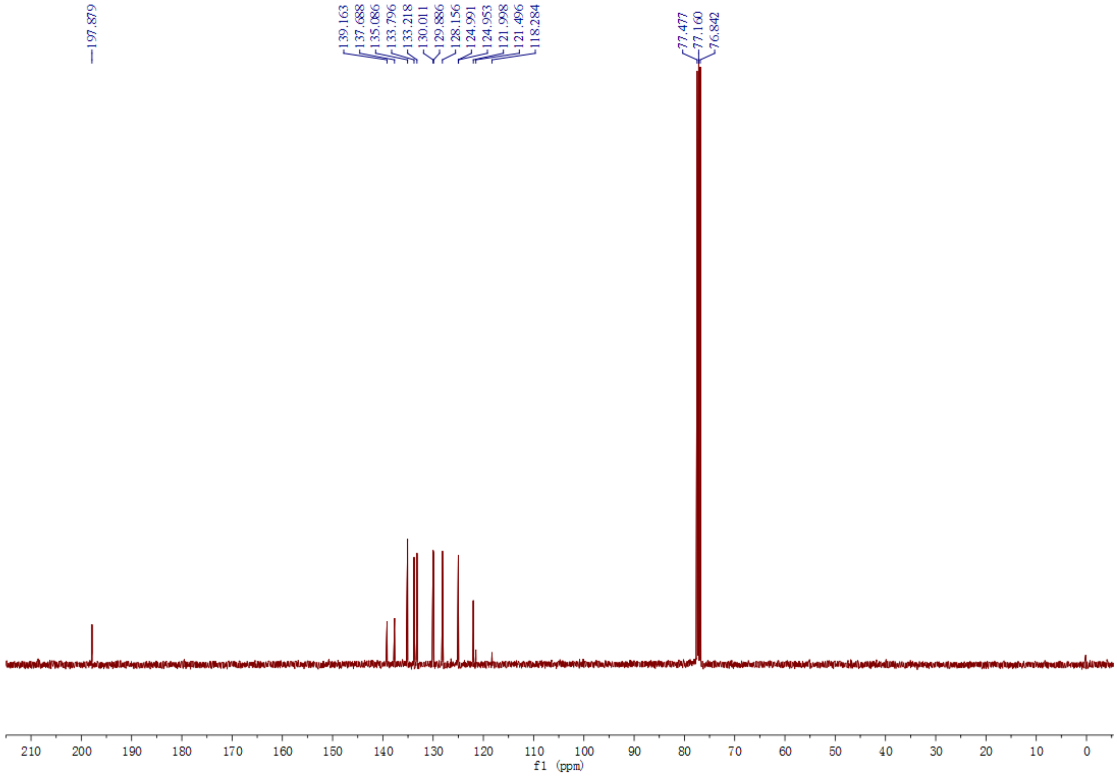

^{13}C NMR Spectrum for **4m** ($CDCl_3$, 100 MHz)

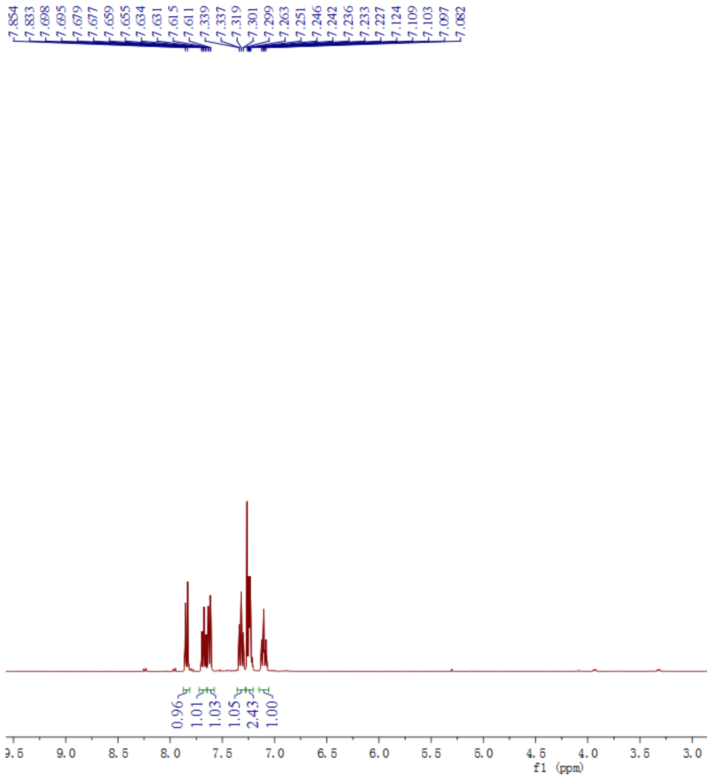

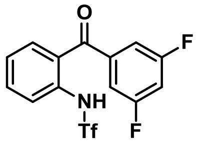

4n

^{1}H NMR Spectrum for **4n** ($CDCl_3$, 400 MHz)

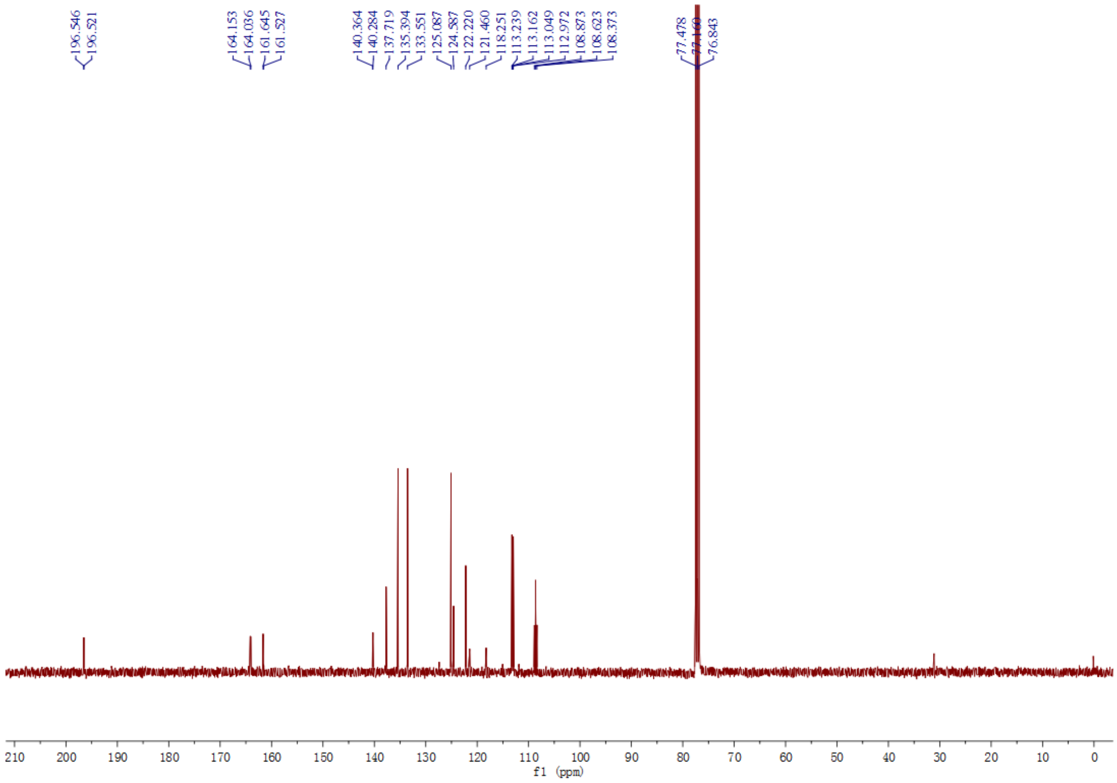

^{13}C NMR Spectrum for **4n** ($CDCl_3$, 100 MHz)

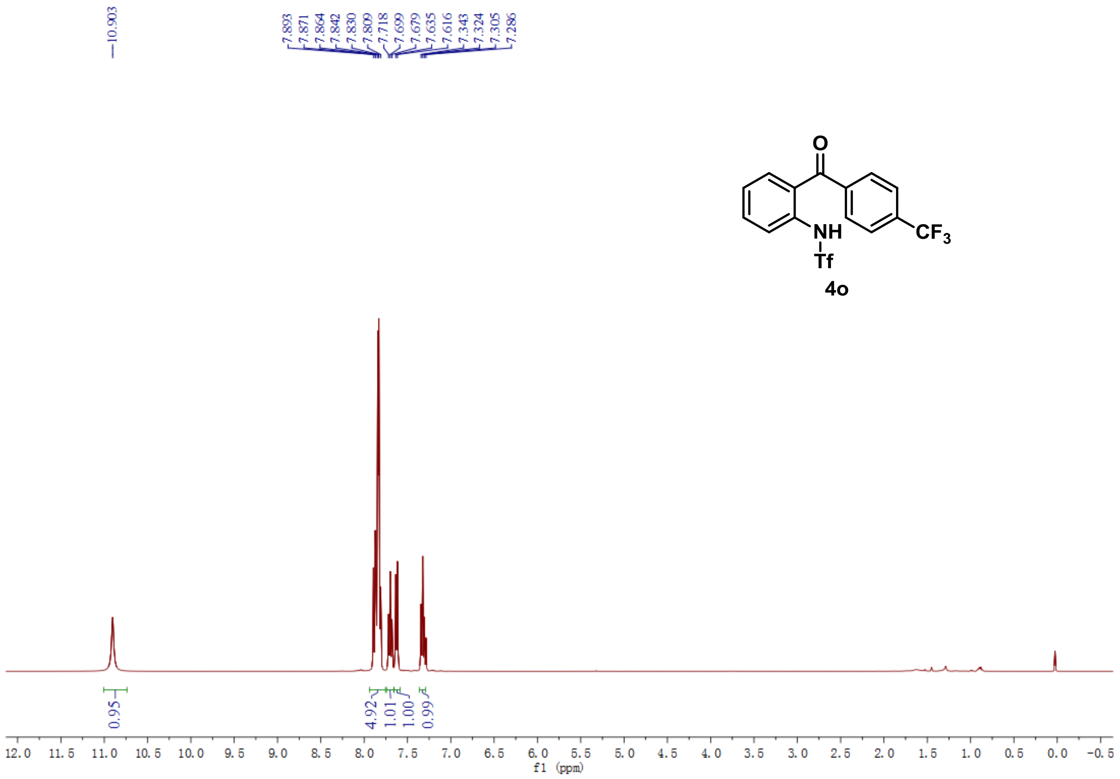

^{1}H NMR Spectrum for **4o** ($CDCl_3$, 400 MHz)

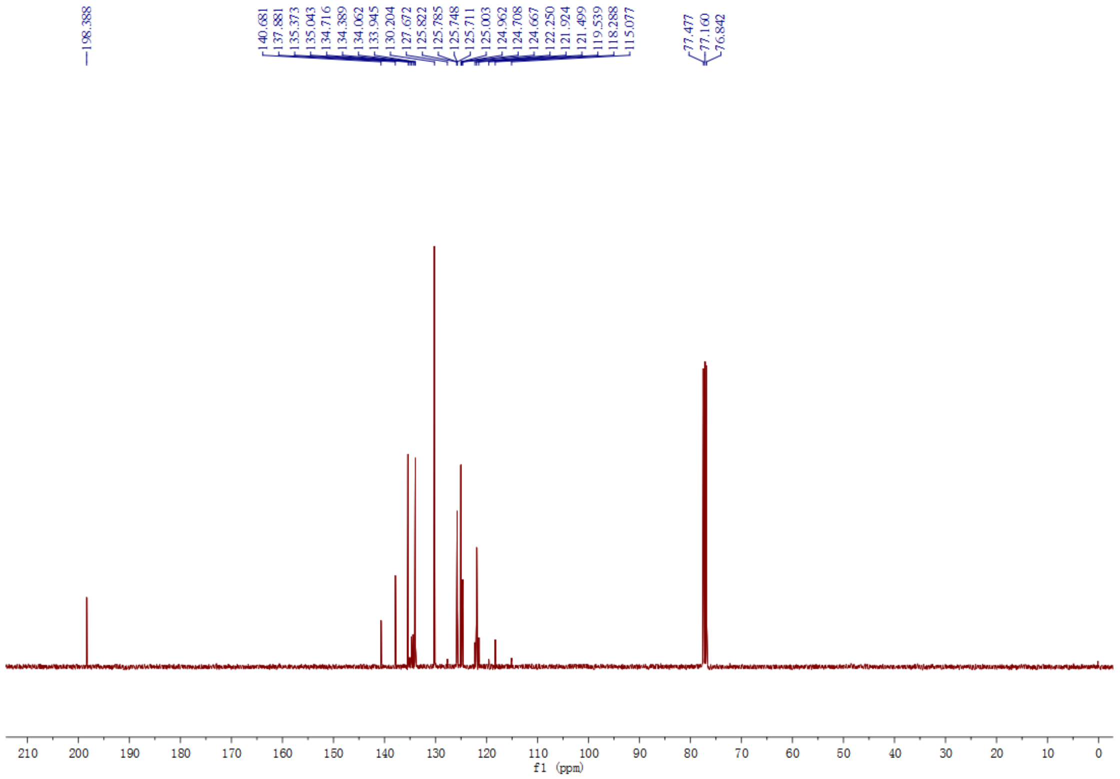

^{13}C NMR Spectrum for **4o** ($CDCl_3$, 100 MHz)

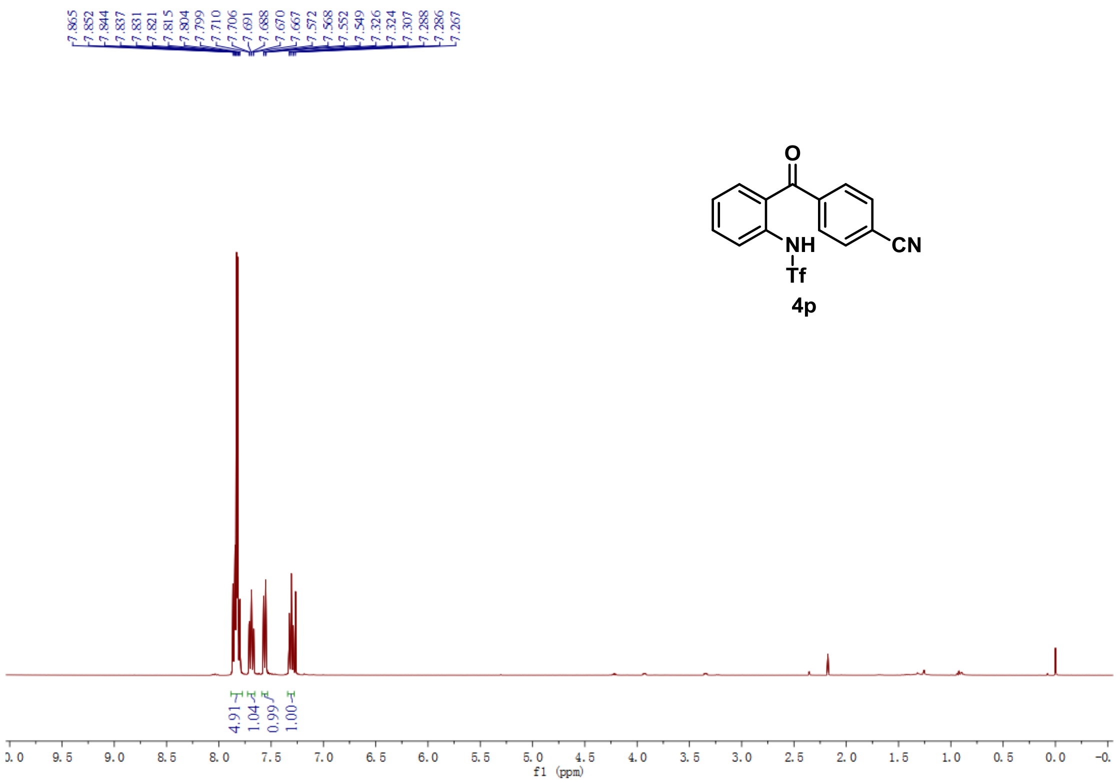

^{1}H NMR Spectrum for **4p** ($CDCl_3$, 400 MHz)

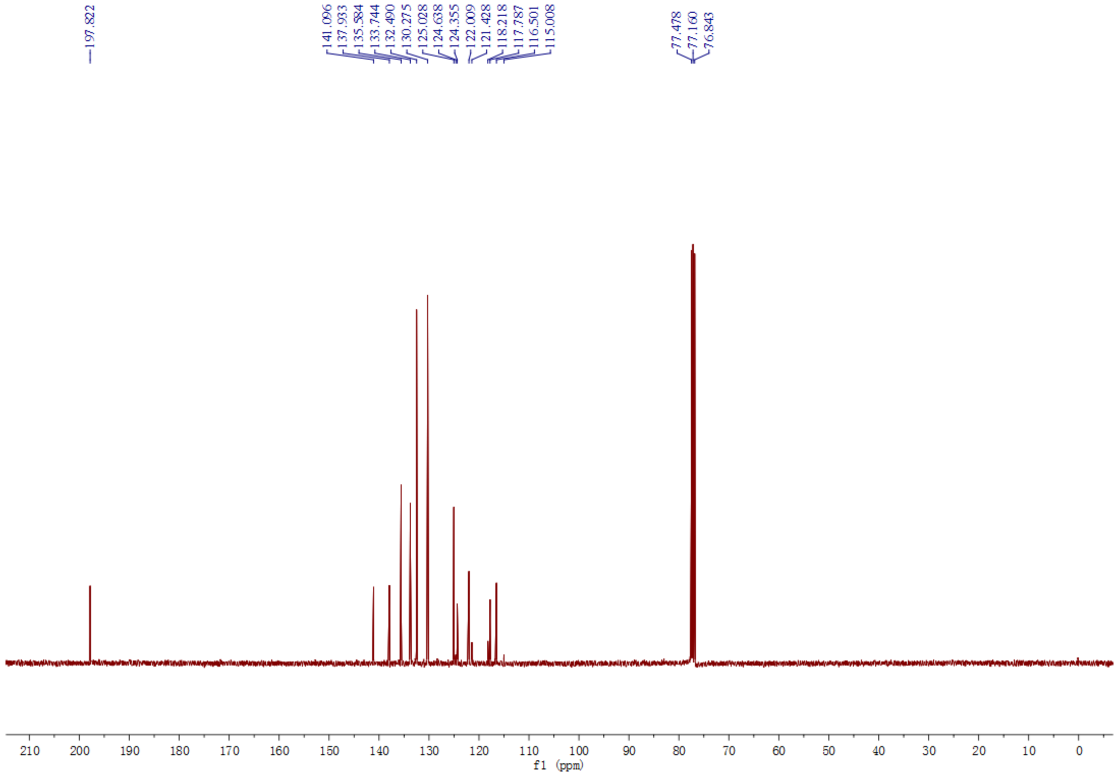

^{13}C NMR Spectrum for **4p** ($CDCl_3$, 100 MHz)

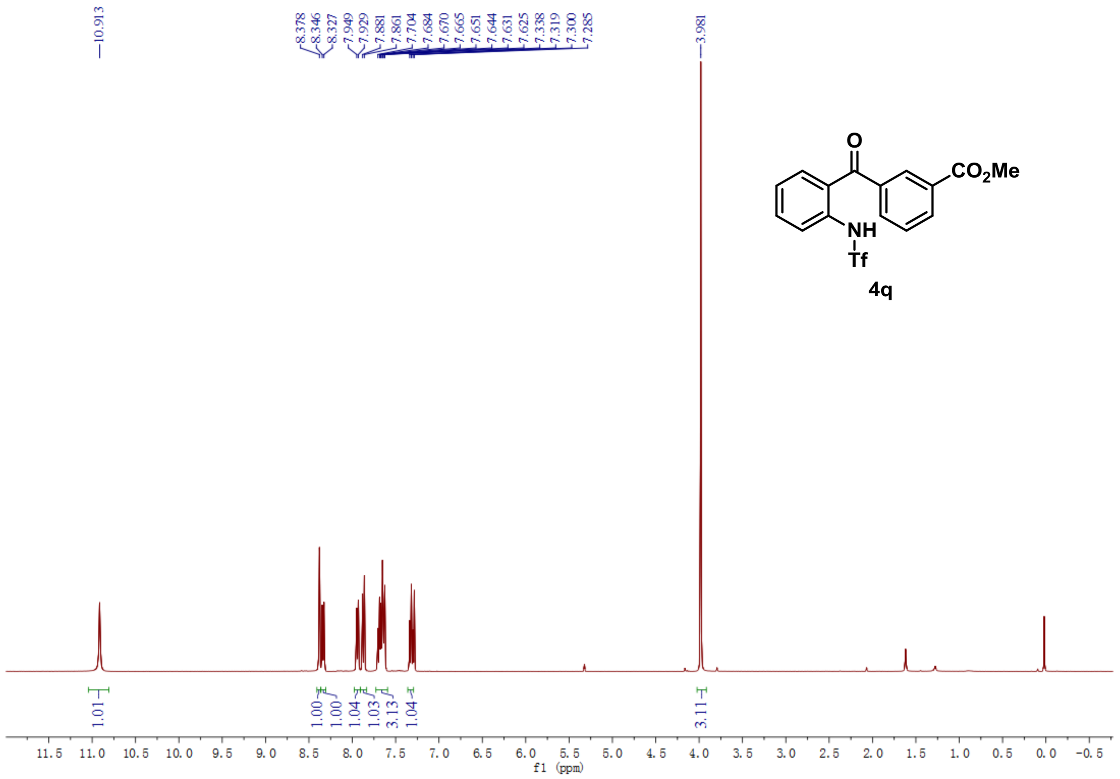

^{1}H NMR Spectrum for **4q** ($CDCl_3$, 400 MHz)

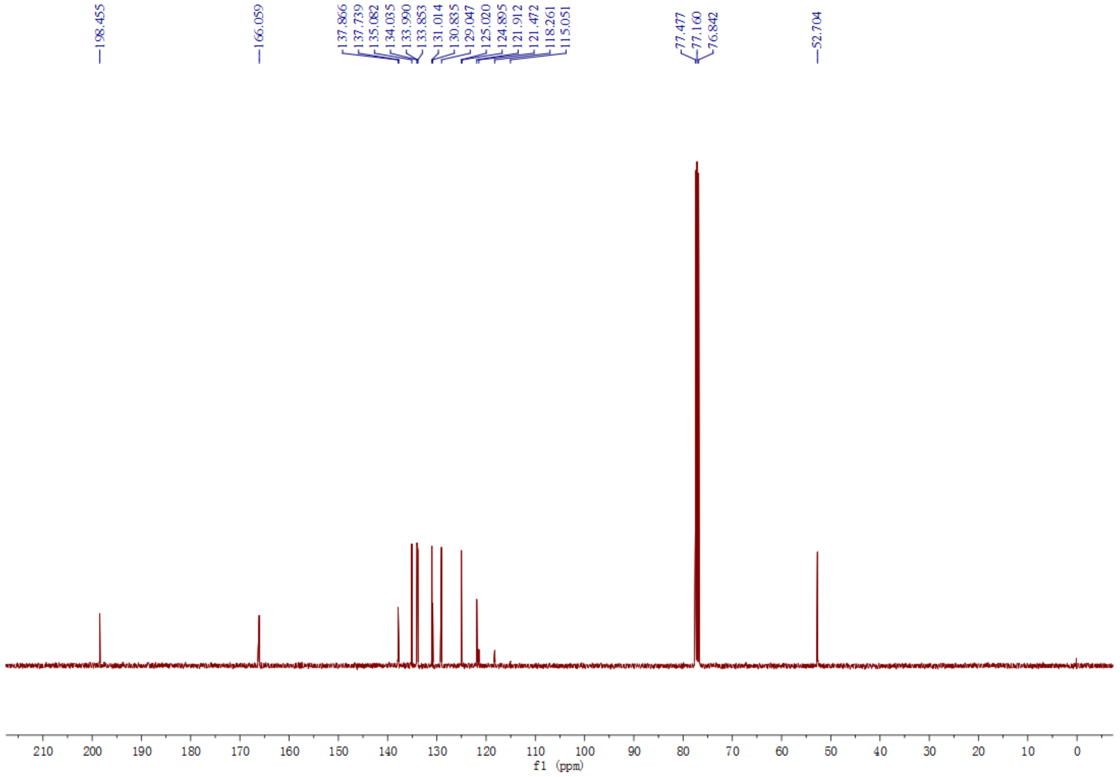

^{13}C NMR Spectrum for **4q** ($CDCl_3$, 100 MHz)

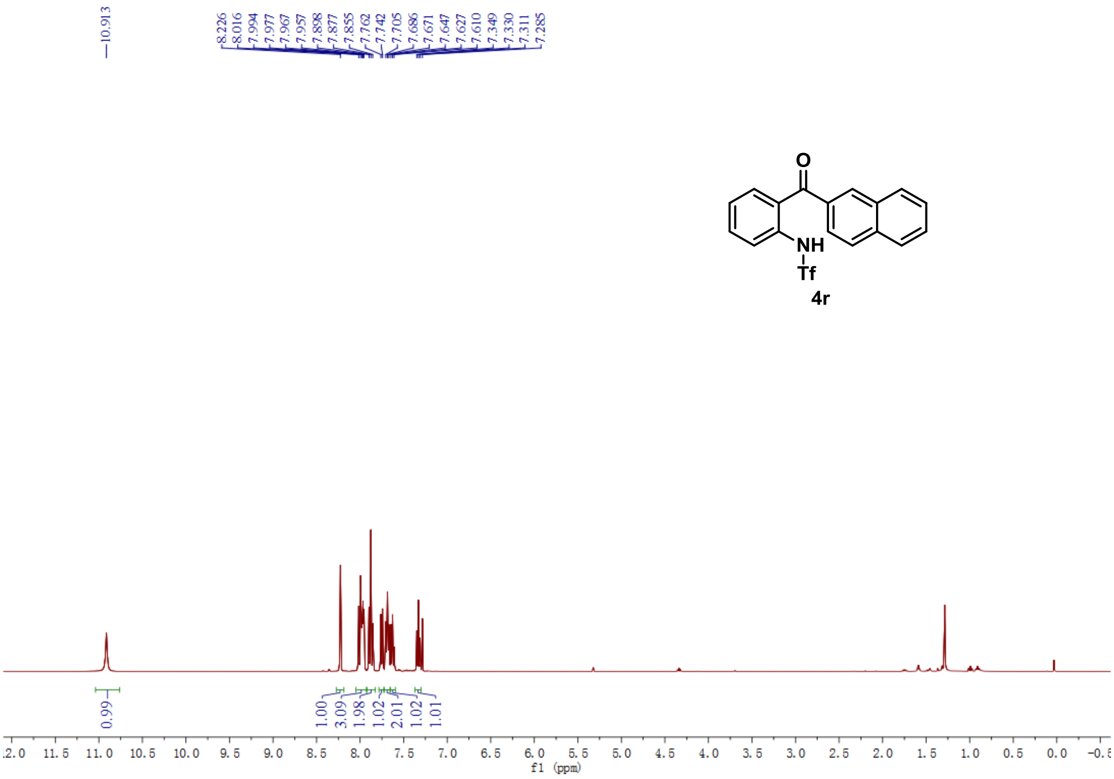

4r

^{1}H NMR Spectrum for **4r** ($CDCl_3$, 400 MHz)

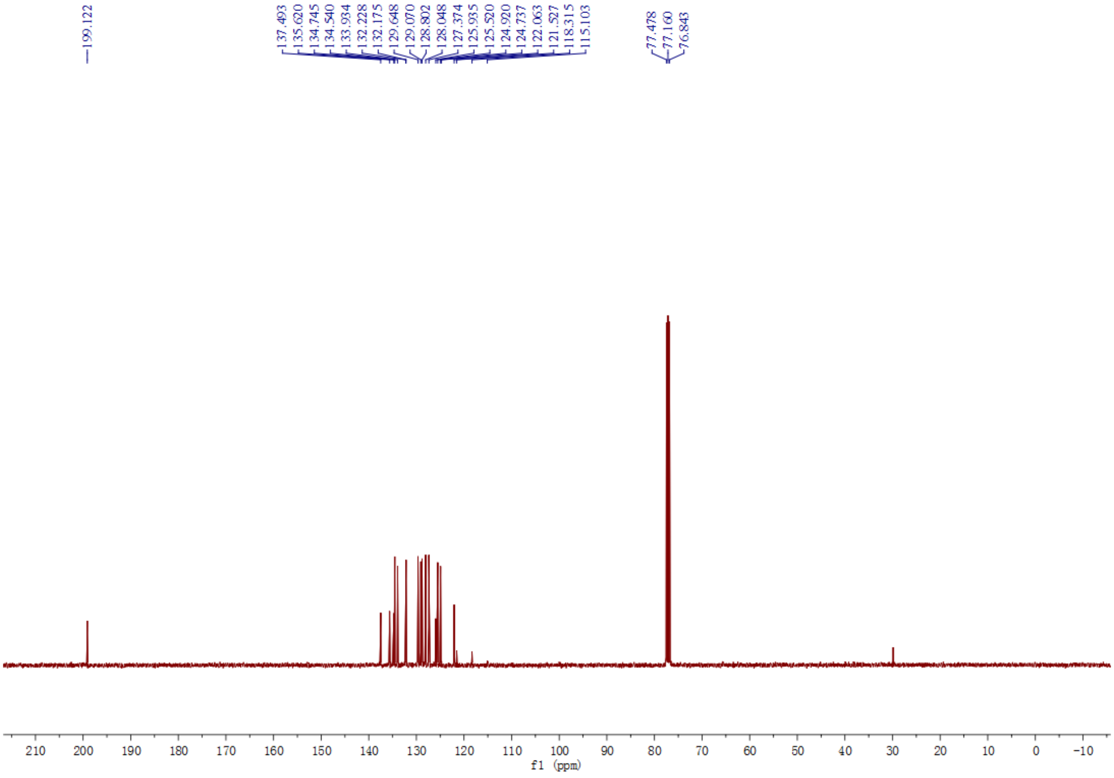

^{13}C NMR Spectrum for **4r** ($CDCl_3$, 100 MHz)

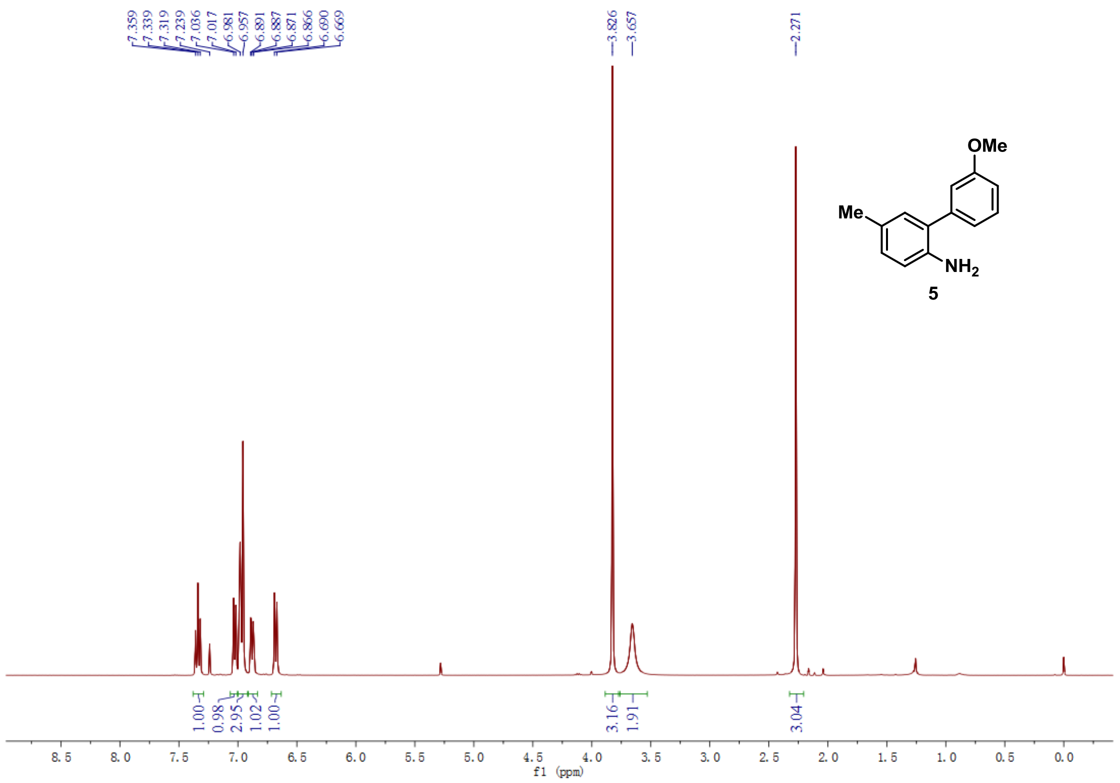

^{1}H NMR Spectrum for **5** ($CDCl_3$, 400 MHz)

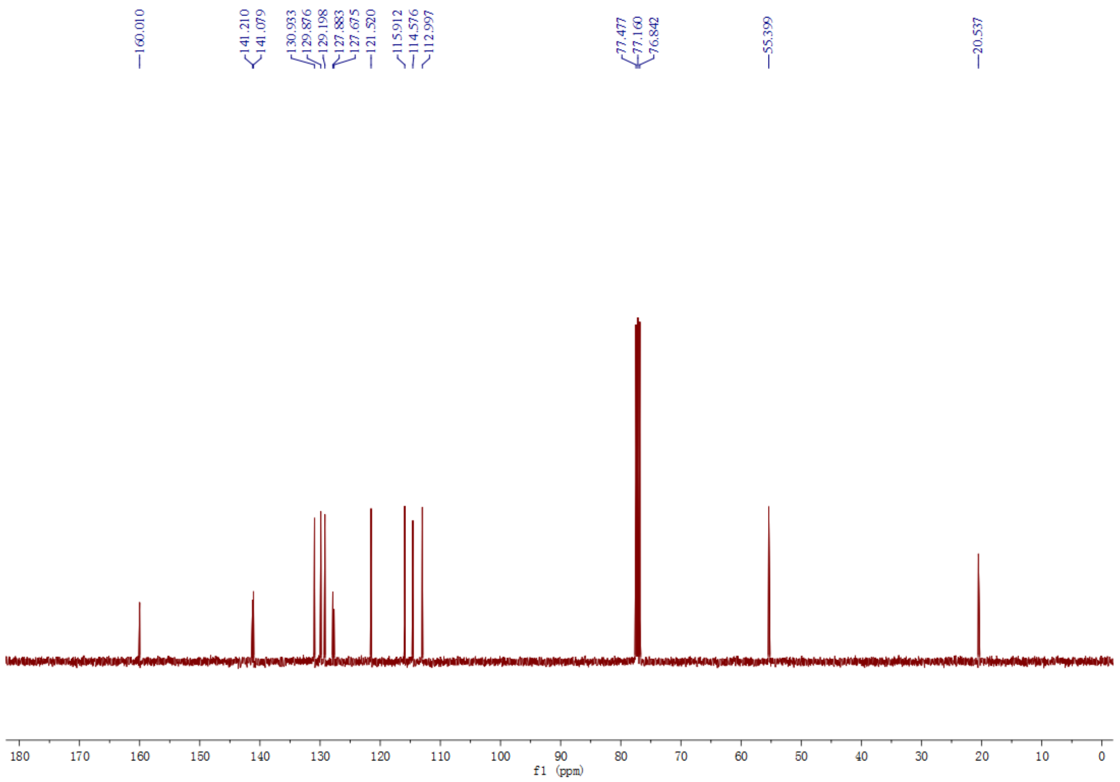

^{13}C NMR Spectrum for **5** ($CDCl_3$, 100 MHz)

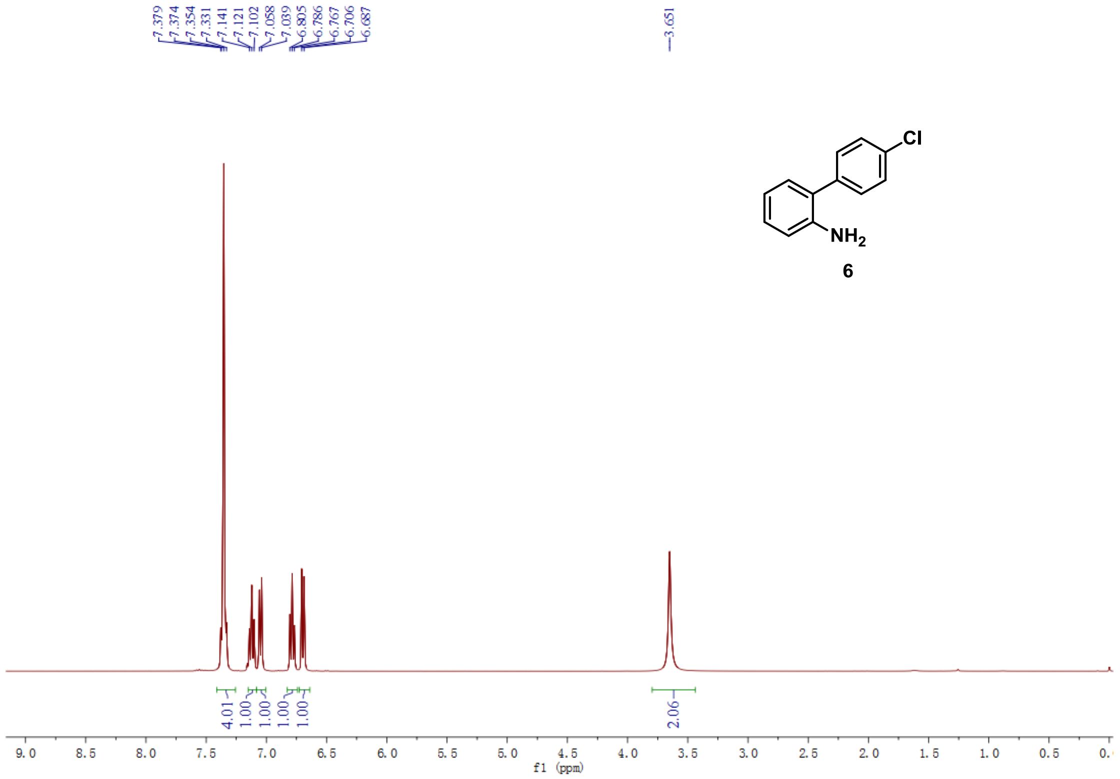

^{1}H NMR Spectrum for **6** ($CDCl_3$, 400 MHz)

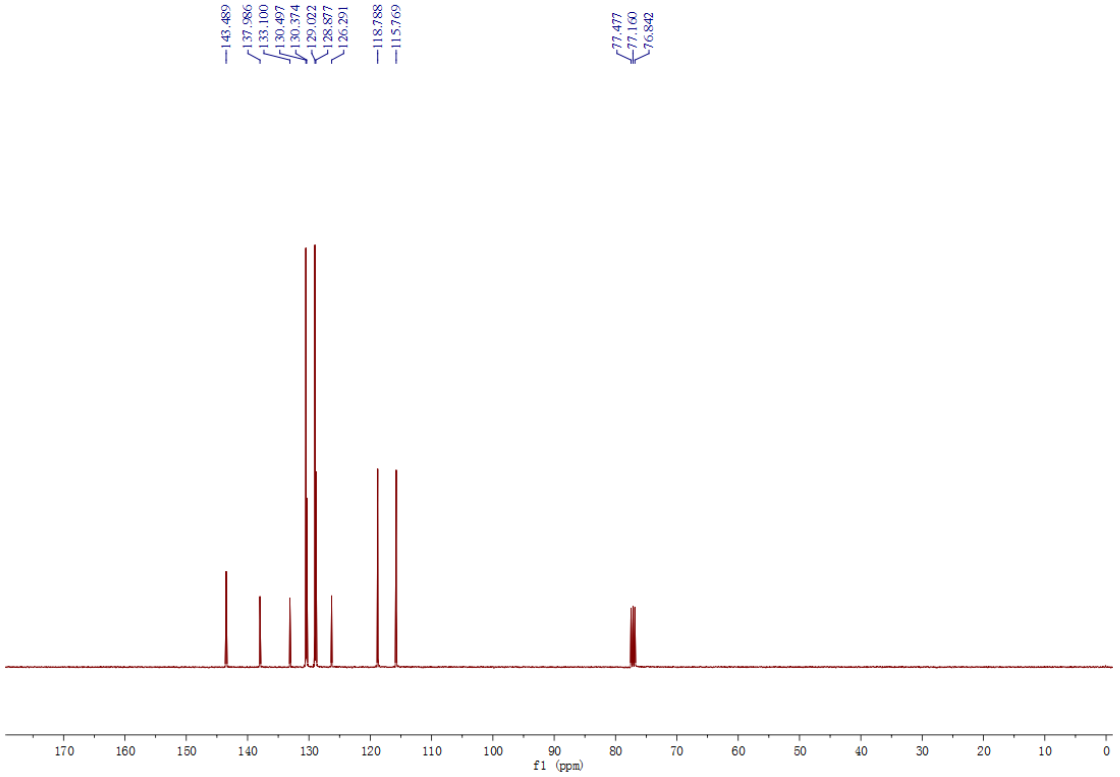

^{13}C NMR Spectrum for **6** ($CDCl_3$, 100 MHz)

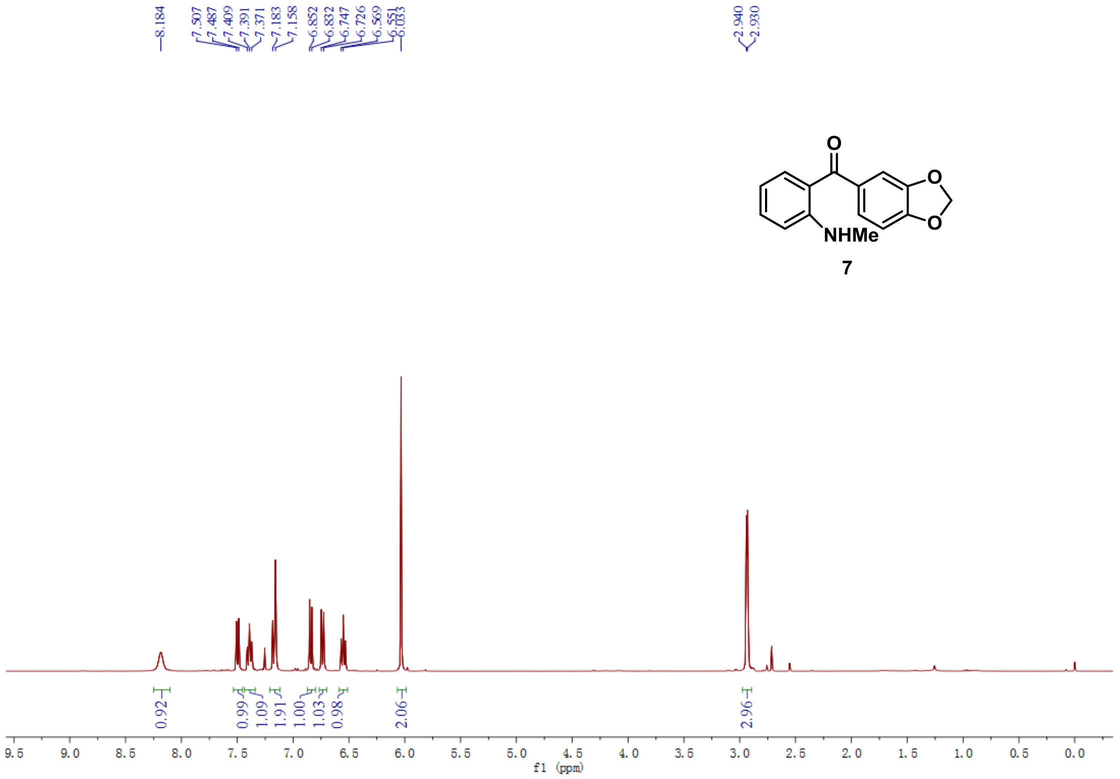

^{1}H NMR Spectrum for **7** ($CDCl_3$, 400 MHz)

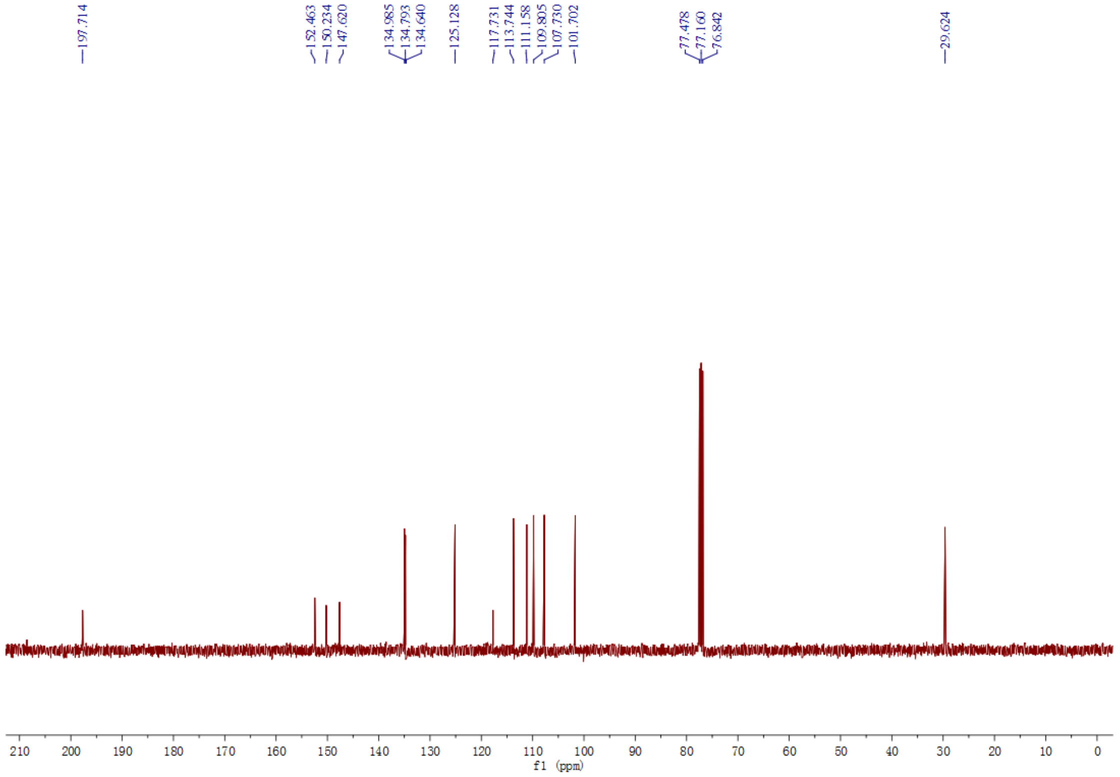

^{13}C NMR Spectrum for **7** ($CDCl_3$, 100 MHz)

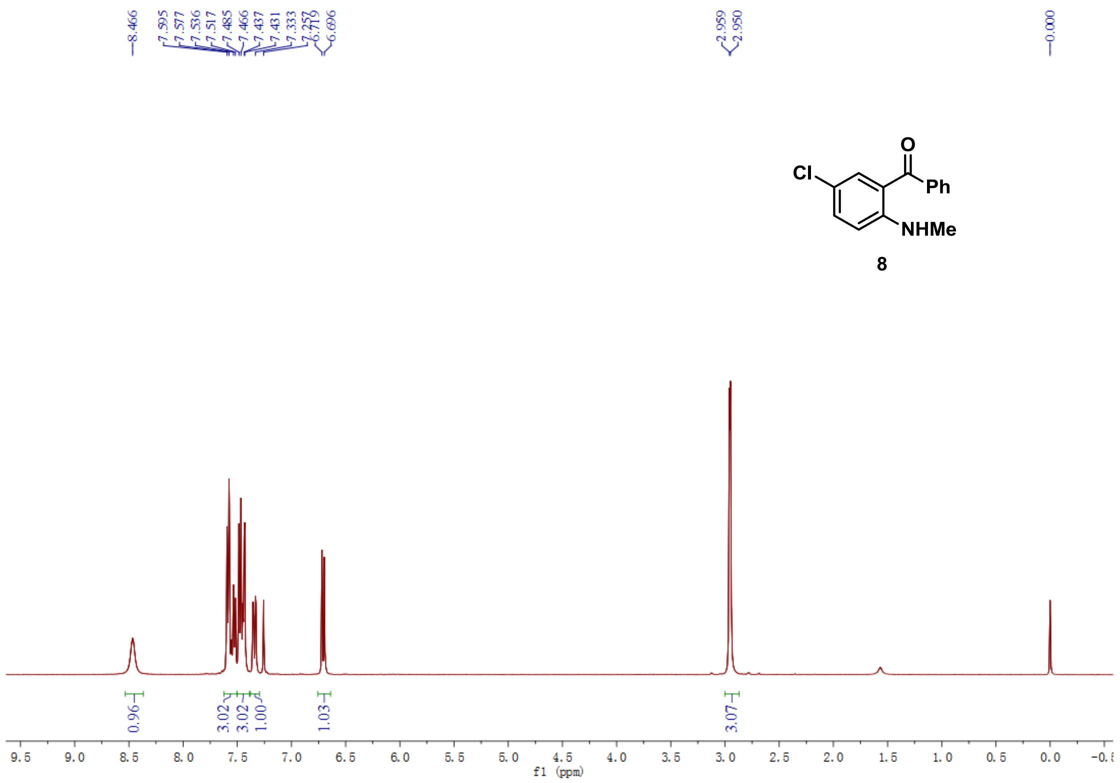

^{1}H NMR Spectrum for **8** ($CDCl_3$, 400 MHz)

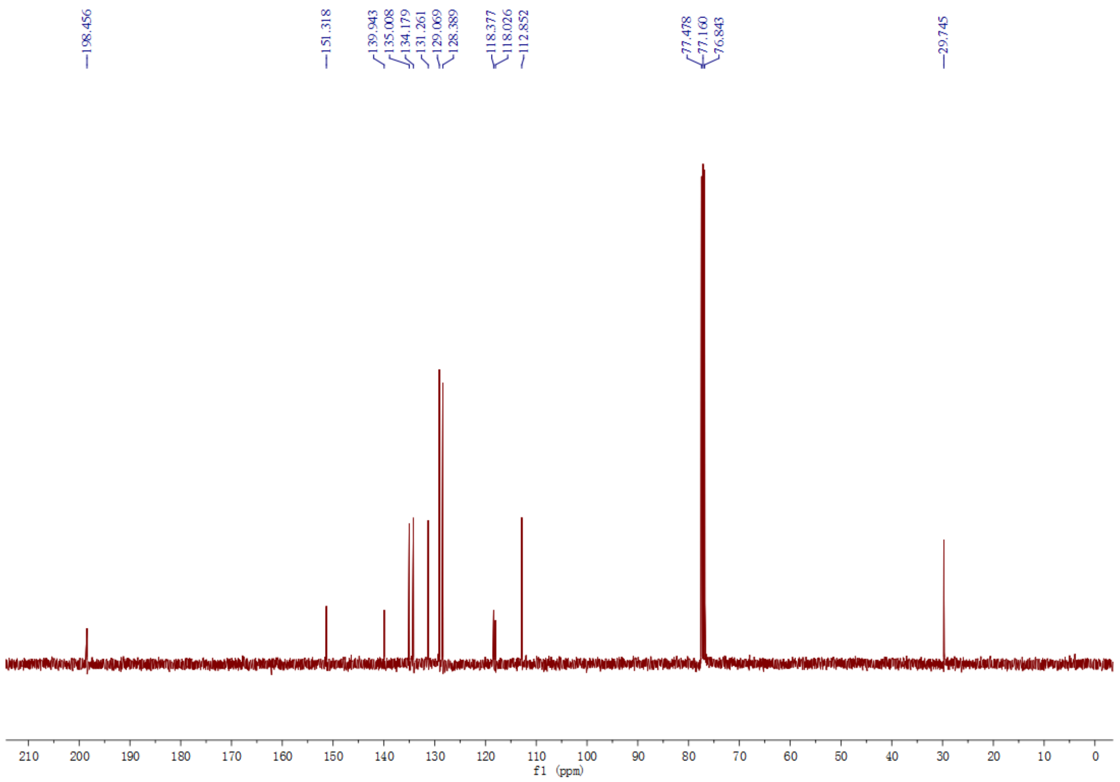

^{13}C NMR Spectrum for **8** ($CDCl_3$, 100 MHz)

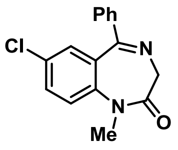

Diazepam

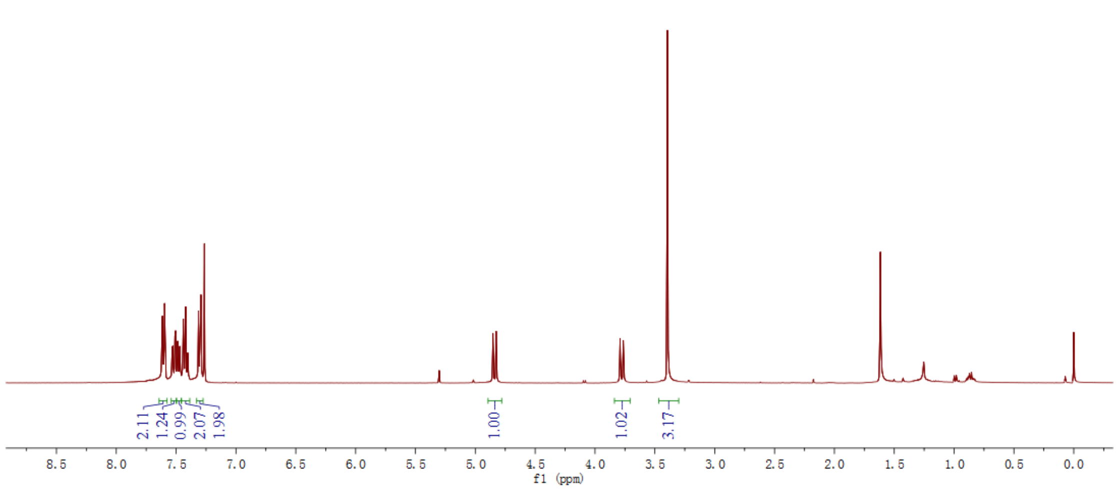

^{1}H NMR Spectrum for **Diazepam** ($CDCl_3$, 400 MHz)

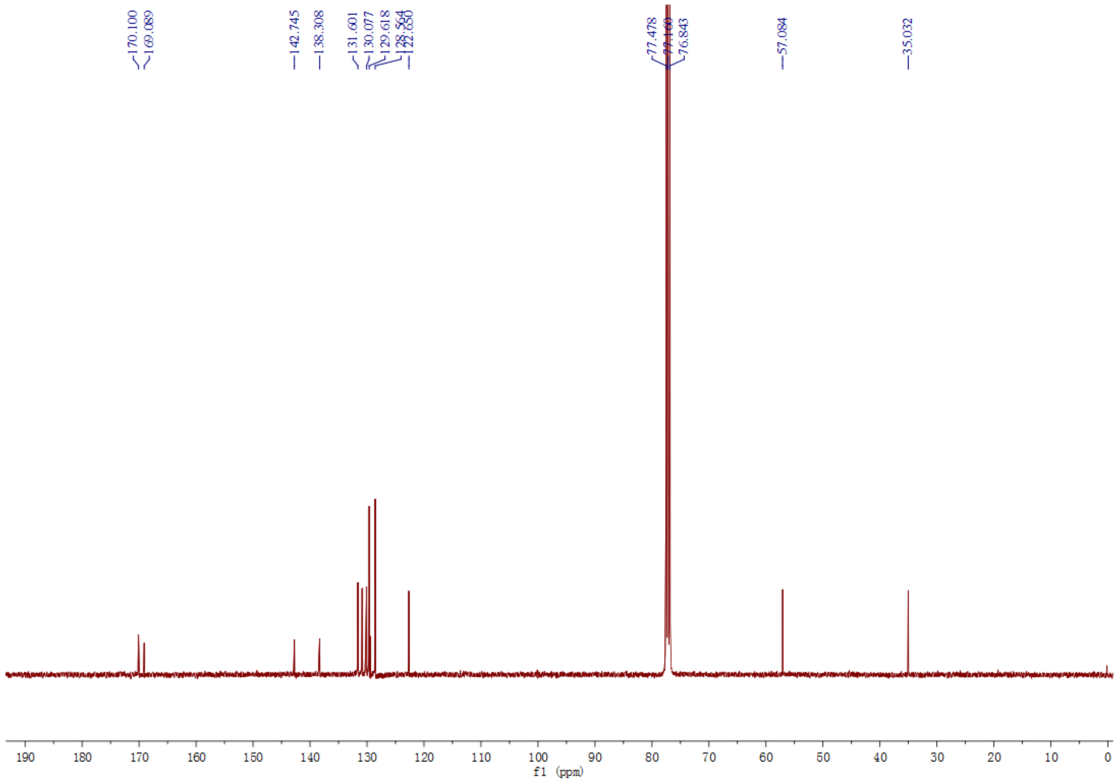

^{13}C NMR Spectrum for **Diazepam** ($CDCl_3$, 100 MHz)

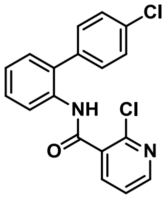

Boscalid

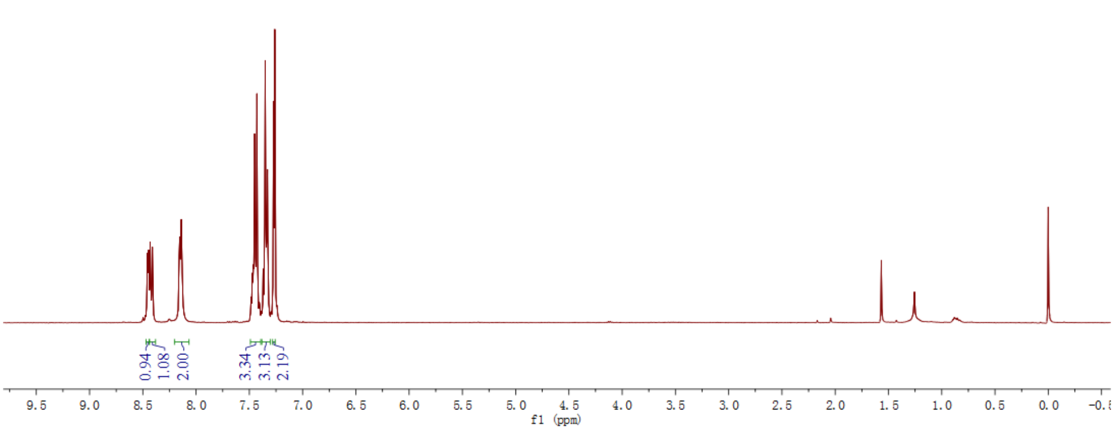

^{1}H NMR Spectrum for **Boscalid** ($CDCl_3$, 400 MHz)

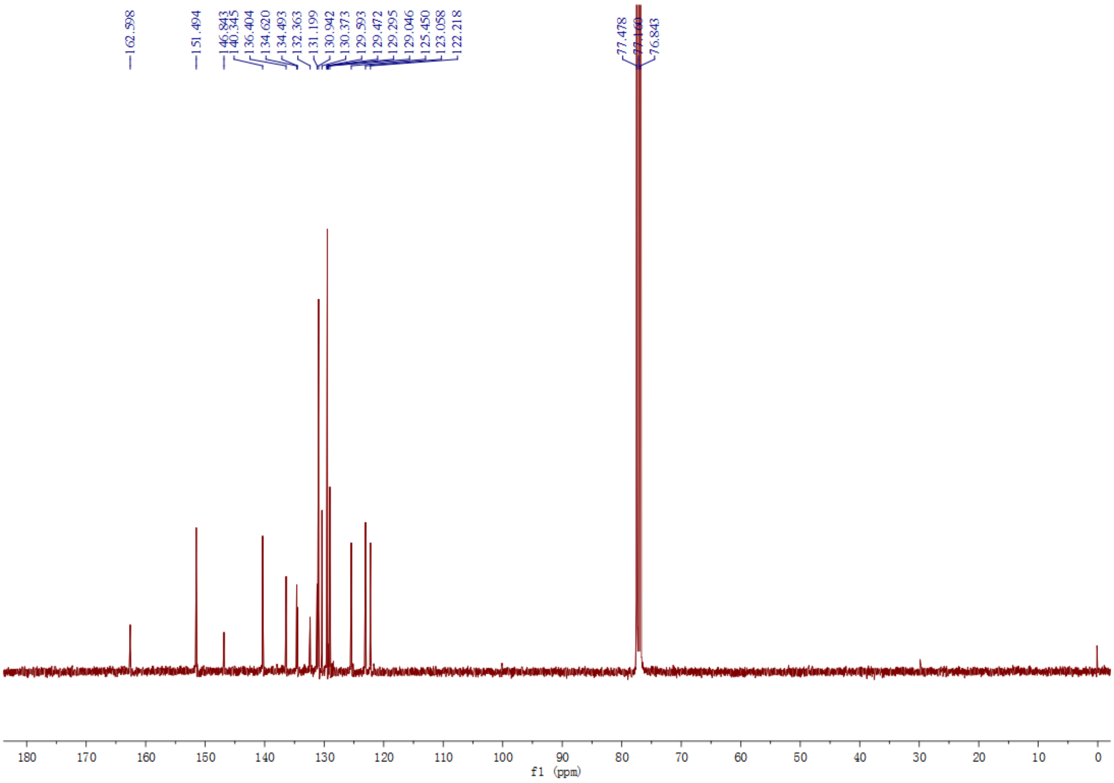

^{13}C NMR Spectrum for **Boscalid** ($CDCl_3$, 100 MHz)

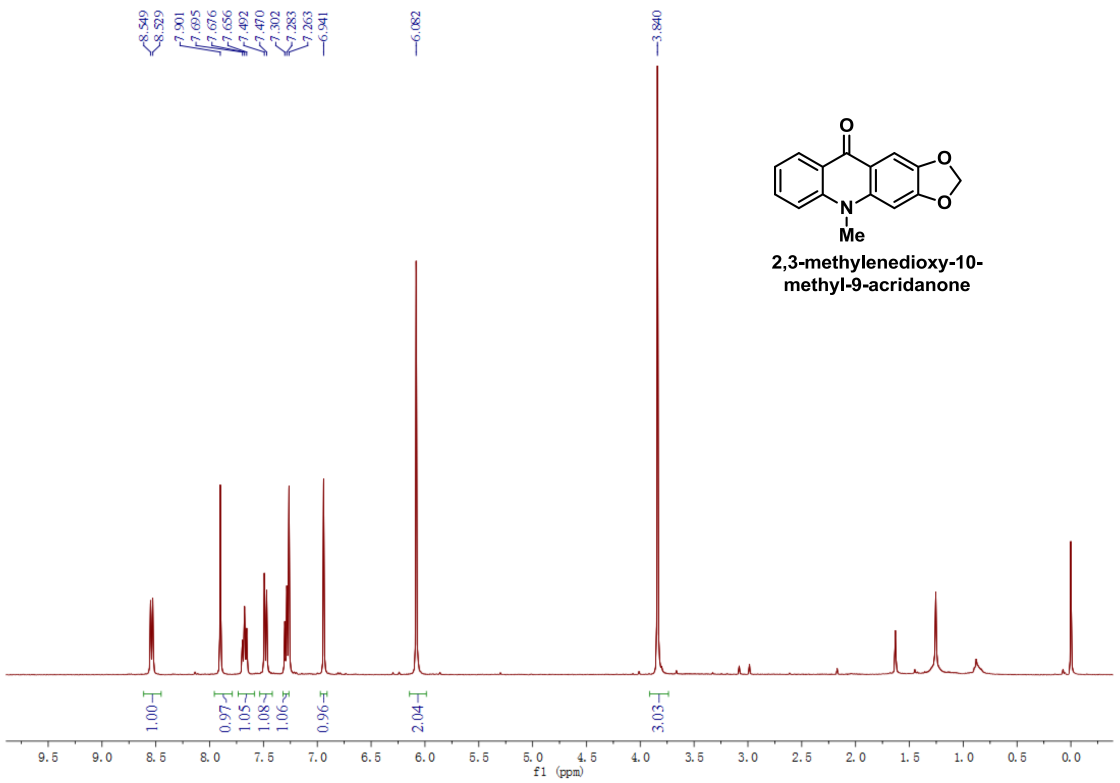

^{1}H NMR Spectrum for **2,3-methylenedioxy-10-methyl-9-acridanone** ($CDCl_3$, 400 MHz)

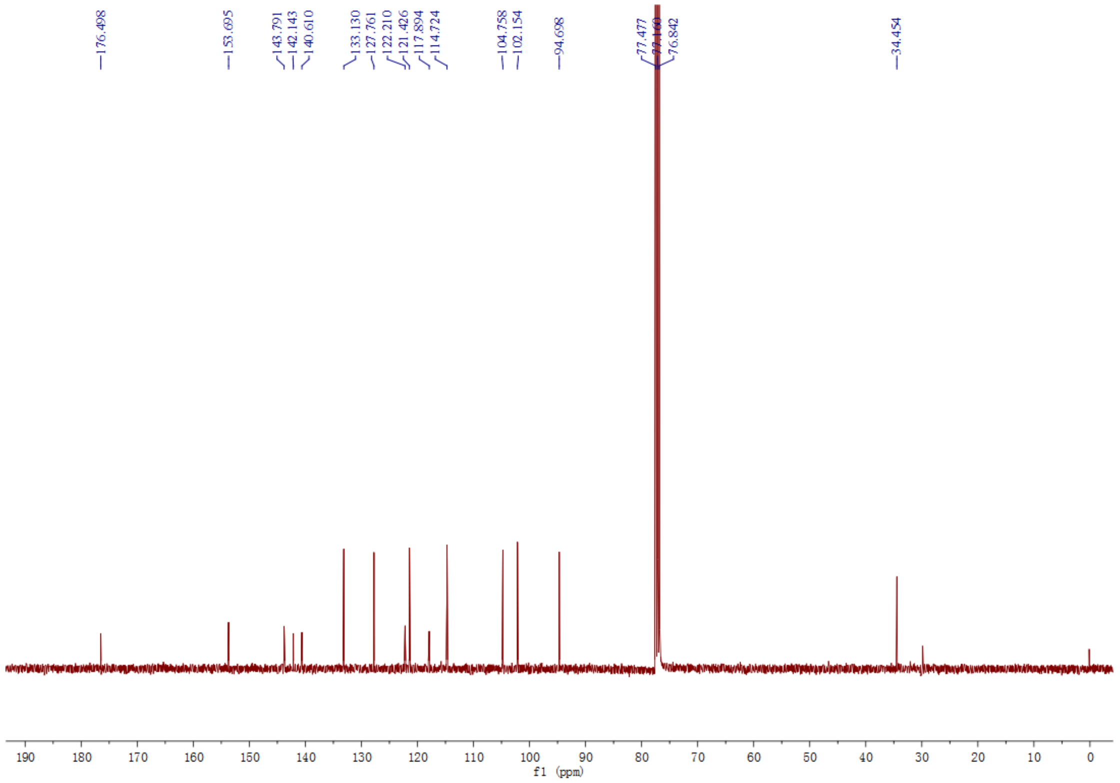

^{13}C NMR Spectrum for **2,3-methylenedioxy-10-methyl-9-acridanone** ($CDCl_3$, 100 MHz)

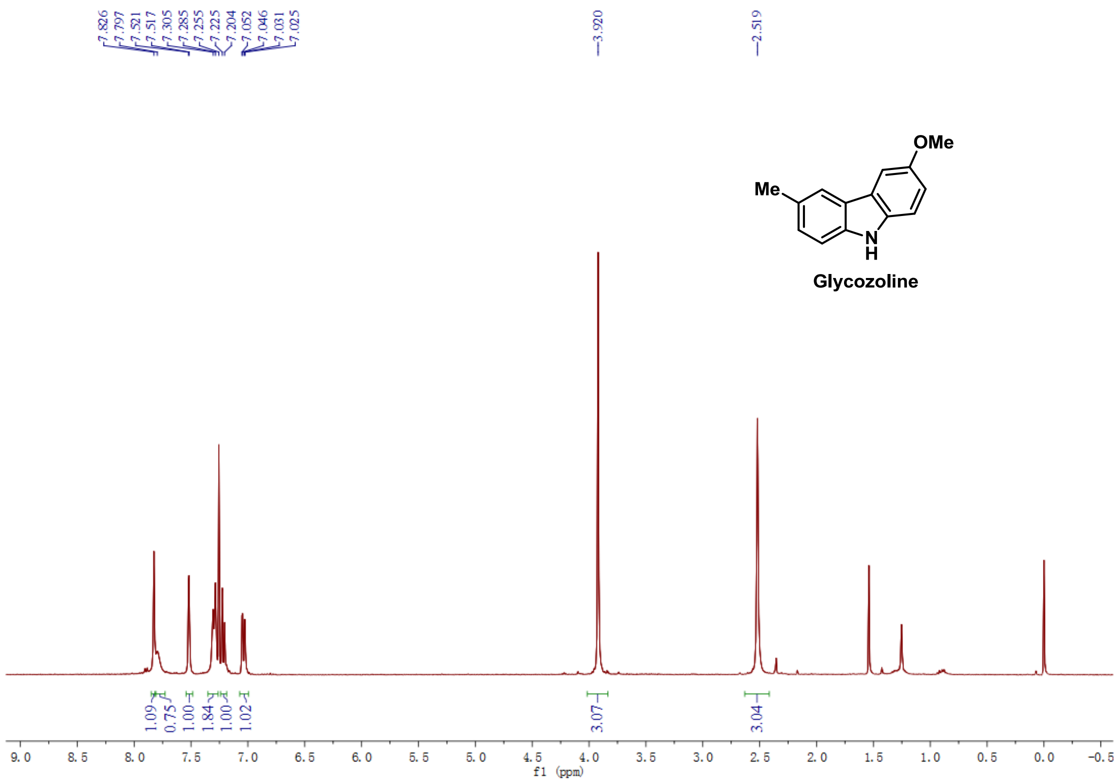

^{1}H NMR Spectrum for **Glycozoline** ($CDCl_3$, 400 MHz)

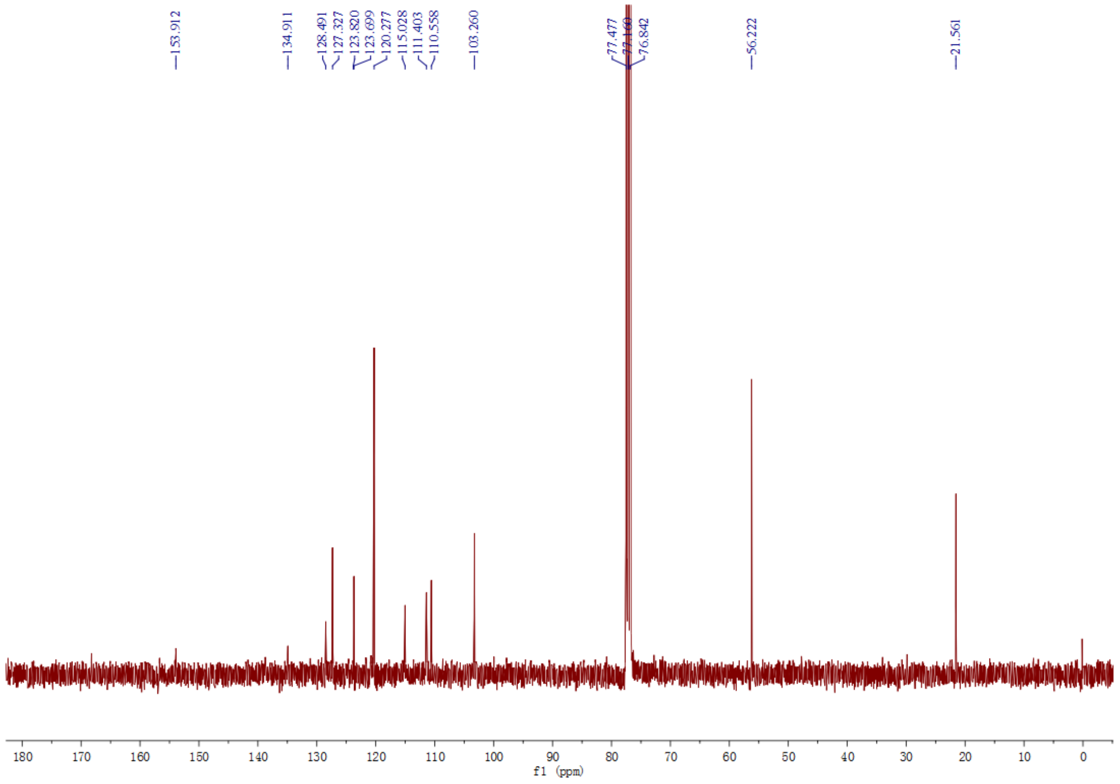

^{13}C NMR Spectrum for **Glycozoline** ($CDCl_3$, 100 MHz)

9. Computational Studies

1) Computational methods.

Calculation for organic compounds were performed with the Gaussian 09 program package[9] and ORCA 3.0.3 program[10]. The geometry optimizations of all structures were performed using M06-2X functional[11] corrected by DFT-D3 with Becke-Johnson damping[12]. For the basis set, the ma-TZVP basis set[13] was used for all nitrogen atoms in the substrate and fluorine atoms in BF_4^- anion if present; TZVP basis set[14] without diffusion functions was used for other non-metal atoms; LanL2TZ(f) basis and corresponding pseudopotential[15] was applied to silver atom. Higher level of single point electronic energy was calculated at PWPB95-D3/def2-QZVPP[16] level with minimal augmentation functions also added to the nitrogen atoms in the substrate and fluorine atoms on BF_4^- anion if present. Solvation effects was computed by SMD model[17] in toluene at M05-2X/6-31G(d) level[18] with the exception of LanL2TZ(f) basis and pseudopotential for Ag atom. The vibrational harmonic frequencies and thermal corrections were computed using the same level as the optimization; the former confirmed the optimized geometrical structures are the minima of PES, and transition states, the first order saddle points. All energies discussed in the paper are Gibbs free energies in toluene (ΔG_{sol}, $\Delta G_{sol}^{\neq}$).

2) Energy and Geometries.

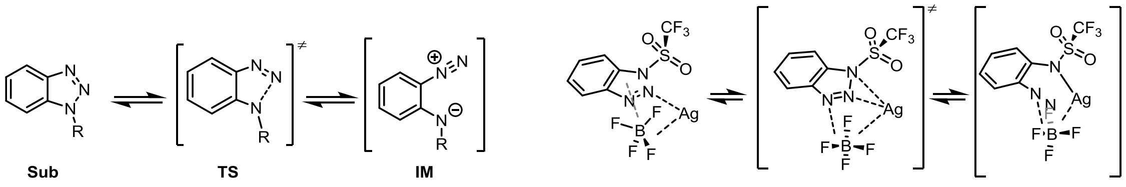

---

[9] *Gaussian 09, Revision D.01*, M. J. Frisch, G. W. Trucks, H. B. Schlegel, G. E. Scuseria, M. A. Robb, J. R. Cheeseman, G. Scalmani, V. Barone, B. Mennucci, G. A. Petersson, H. Nakatsuji, M. Caricato, X. Li, H. P. Hratchian, A. F. Izmaylov, J. Bloino, G. Zheng, J. L. Sonnenberg, M. Hada, M. Ehara, K. Toyota, R. Fukuda, J. Hasegawa, M. Ishida, T. Nakajima, Y. Honda, O. Kitao, H. Nakai, T. Vreven, J. A. Montgomery, Jr., J. E. Peralta, F. Ogliaro, M. Bearpark, J. J. Heyd, E. Brothers, K. N. Kudin, V. N. Staroverov, T. Keith, R. Kobayashi, J. Normand, K. Raghavachari, A. Rendell, J. C. Burant, S. S. Iyengar, J. Tomasi, M. Cossi, N. Rega, J. M. Millam, M. Klene, J. E. Knox, J. B. Cross, V. Bakken, C. Adamo, J. Jaramillo, R. Gomperts, R. E. Stratmann, O. Yazyev, A. J. Austin, R. Cammi, C. Pomelli, J. W. Ochterski, R. L. Martin, K. Morokuma, V. G. Zakrzewski, G. A. Voth, P. Salvador, J. J. Dannenberg, S. Dapprich, A. D. Daniels, O. Farkas, J. B. Foresman, J. V. Ortiz, J. Cioslowski, and D. J. Fox, Gaussian, Inc., Wallingford CT, **2013**.

[10] Frank Neese, WIREs Comput. Mol. Sci. **2012**, *2*, 73-78.

[11] Y. Zhao and D. G. Truhlar, *Theor. Chem. Acc.*, **2008**, 120, 215-241.

[12] S. Grimme, S. Ehrlich, L. Goerigk, *J. Comp. Chem*. **2011**, 32, 1456-1465.

[13] J. Zheng, X. Xu, D. Truhlar, *Theor Chem Acc* **2011**, 128, 295-305.

[14] A. Schaefer, C. Huber, and R. Ahlrichs, *J. Chem. Phys.*, **1994**, 100, 5829-5835.

[15] (a) L. Roy, J. Hay and R. Martin, *Chem. Theory Comput.*, **2008**, *4* (7), 1029–1031; (b) A.W. Ehlers, M. Böhme, S. Dapprich, A. Gobbi, A. Höllwarth, V. Jonas, K.F. Köhler, R. Stegmann, A. Veldkamp, G. Frenking, *Chem. Phys. Lett.* **1993**, 208, 111-114.

[16] L. Goerigk, S. Grimme, *J. Chem. Theory Comput.*, **2011**, *7* (2), 291–309.

[17] A. V. Marenich, C. J. Cramer, and D. G. Truhlar, *J. Phys. Chem. B,* **2009**, 113, 6378-6396.

[18] Y. Zhao, N. E. Schultz, and D. G. Truhlar, *J. Chem. Theory and Comput.*, **2006**, 2, 364-382.

Species	R = Bz			R = Ts		
	TS	Sub	IM	TS	Sub	IM
**E_{elec} at optimization level (kJ/mol)**	-1943264.64	-1943388.81	-1943284.01	-3085924.50	-3086033.35	-3085949.00
**E_{elec} at single point level(kJ/mol)**	-1943286.69	-1943403.56	-1943296.64	-3086216.32	-3086314.20	-3086230.12
**Imaginaries**	1	0	0	1	0	0
**H correction (kJ/mol)**	559.25	567.13	563.12	564.71	572.05	568.67
**G correction (kJ/mol)**	520.96	508.38	547.21	558.37	554.39	578.74
**S correction (J/mol K)**	375.27	387.59	369.87	367.52	376.27	364.29
**Solvent**	Toluene	Toluene	Toluene	Toluene	Toluene	Toluene
**Solvation Gibbs Free Energy (kJ/mol)**	-39.55	-41.39	-42.26	-46.70	-45.19	-51.75
**Solvated Gibbs Free Energy (kJ/mol)**	-1942950.97	-1943057.36	-1942969.02	-3085895.49	-3085983.13	-3085917.57

Species	R = Tf			R = Tf with $AgBF_4$		
	TS	Sub	IM	TS	Sub	IM
**E_{elec} at optimization level (kJ/mol)**	-3364297.07	-3364372.82	-3364323.81	-4861417.47	-4861471.37	-4861481.10
**E_{elec} at single point level(kJ/mol)**	-3364678.95	-3364746.19	-3364694.92	-4865092.53	-4865136.79	-4865142.77
**Imaginaries**	1	0	0	1	0	0
**H correction (kJ/mol)**	359.95	366.27	363.95	431.01	437.68	435.47
**G correction (kJ/mol)**	523.70	523.12	545.61	748.52	726.68	751.84
**S correction (J/mol K)**	175.00	181.53	171.27	166.67	181.06	169.95
**Solvent**	Toluene	Toluene	Toluene	Toluene	Toluene	Toluene
**Solvation Gibbs Free Energy (kJ/mol)**	-24.96	-20.89	-32.58	-55.23	-61.80	-55.04
**Solvated Gibbs Free Energy (kJ/mol)**	-3364528.91	-3364585.56	-3364556.24	-4864981.09	-4865017.54	-4865027.86

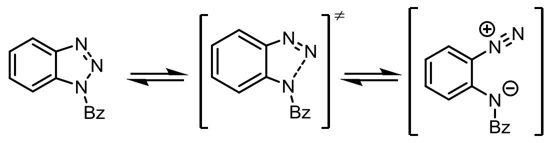

Sub **TS** **IM**

TS

C	3.778745	-1.021409	0.152735
C	2.697486	-1.879373	-0.146527
C	1.404803	-1.442115	-0.284387
C	1.077920	-0.060189	-0.137681
C	2.224963	0.756956	0.168045
C	3.542073	0.316239	0.313358
N	1.812321	2.053331	0.300600
N	-0.079279	0.572576	-0.263356
N	0.859196	2.638494	0.235232
H	4.778654	-1.418743	0.254105
H	2.895580	-2.937284	-0.271828

H	0.602348	-2.129282	-0.499782
H	4.325218	1.025752	0.544064
C	-1.283490	-0.073947	-0.383650
O	-1.452075	-1.276800	-0.247409
C	-2.450960	0.822467	-0.682508
C	-2.294174	2.152443	-1.066661
C	-3.732573	0.282758	-0.586291
C	-3.407699	2.932554	-1.348102
H	-1.300386	2.568824	-1.150636
C	-4.844258	1.064731	-0.860347
H	-3.838394	-0.754050	-0.294130
C	-4.683258	2.391984	-1.242263
H	-3.279645	3.964434	-1.650770
H	-5.837103	0.640273	-0.778057
H	-5.550776	3.003157	-1.459335

Sub

C	4.214704	-1.472465	0.545995
C	3.289492	-2.432050	0.089228
C	1.949259	-2.146369	-0.080194
C	1.560679	-0.841137	0.225978
C	2.469840	0.113341	0.679784
C	3.821563	-0.186797	0.848365
N	1.786156	1.298389	0.901556
N	0.368823	-0.150667	0.179813
N	0.567001	1.135170	0.612610
H	5.252340	-1.757536	0.660397
H	3.641955	-3.431321	-0.133148
H	1.240694	-2.885656	-0.420569
H	4.515957	0.564179	1.200931
C	-0.910872	-0.666163	-0.129208
O	-1.041295	-1.861264	-0.200988
C	-2.018886	0.296419	-0.356268
C	-1.840340	1.533885	-0.972847
C	-3.298475	-0.134204	-0.006658
C	-2.942750	2.335545	-1.229114
H	-0.853536	1.864336	-1.264181
C	-4.392042	0.681940	-0.244280
H	-3.422082	-1.106527	0.452951
C	-4.213989	1.917115	-0.856824
H	-2.806909	3.291732	-1.717754
H	-5.382802	0.353890	0.042651
H	-5.069159	2.553075	-1.049138

IM

C	4.136109	-1.626596	0.549452
C	3.025006	-2.497310	0.388931
C	1.741904	-2.058669	0.226014
C	1.433351	-0.654523	0.204719
C	2.602087	0.174361	0.369833
C	3.929380	-0.283124	0.540334
N	2.361446	1.498249	0.353840
N	0.282719	-0.024680	0.046262
N	2.104254	2.569988	0.332343
H	5.130899	-2.029296	0.676830
H	3.204943	-3.565906	0.396365
H	0.928208	-2.755437	0.113984
H	4.732851	0.432573	0.656138
C	-0.903621	-0.702119	-0.072838
O	-1.069853	-1.911206	0.024905
C	-2.085716	0.191310	-0.340478
C	-1.960451	1.574177	-0.461045
C	-3.342798	-0.395805	-0.474886
C	-3.078603	2.357728	-0.712982
H	-0.982916	2.024318	-0.354767
C	-4.459505	0.387103	-0.726250
H	-3.424988	-1.470803	-0.378815
C	-4.329066	1.766353	-0.846047
H	-2.974796	3.431852	-0.805513
H	-5.432771	-0.076648	-0.829306
H	-5.200645	2.378766	-1.042675

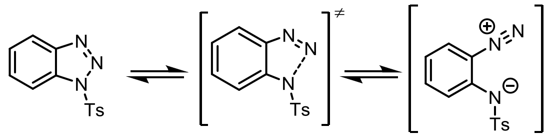

Sub TS IM

TS

C	3.612325	-1.190001	0.347943
C	2.470610	-1.918838	-0.048853
C	1.265840	-1.329582	-0.337939
C	1.113174	0.082134	-0.244409
C	2.307895	0.768777	0.161131

C	3.534852	0.173071	0.452094
N	2.029818	2.111169	0.217464
N	0.064289	0.865195	-0.457126
N	1.116184	2.741497	0.030908
S	-1.431398	0.315194	-0.871207
O	-1.342255	-0.757765	-1.872103
O	-2.224436	1.506636	-1.167911
H	4.538355	-1.704473	0.561461
H	2.547371	-2.995943	-0.135331
H	0.424664	-1.922336	-0.666803
H	4.375989	0.785548	0.747322
C	-2.054063	-0.415199	0.644037
C	-2.255312	-1.784014	0.712166
C	-2.309778	0.420219	1.724064
C	-2.728671	-2.331542	1.899520
H	-2.062191	-2.402071	-0.155138
C	-2.779102	-0.138126	2.902592
H	-2.151308	1.488050	1.634477
C	-2.985249	-1.512010	2.989720
H	-2.897914	-3.398484	1.968038
H	-2.987602	0.498054	3.753197
H	-3.354116	-1.942751	3.912148

Sub

C	4.193049	-1.049866	-0.231824
C	3.267497	-1.764816	-1.020419
C	1.971141	-1.335417	-1.212112
C	1.620907	-0.147817	-0.565139
C	2.529377	0.567462	0.218794
C	3.841938	0.123871	0.395368
N	1.893866	1.687442	0.723522
N	0.486544	0.629196	-0.499600
N	0.696302	1.705029	0.310653
S	-1.126242	0.284633	-1.002347
O	-0.966833	-0.838748	-1.914020
O	-1.691881	1.552890	-1.417337
H	5.198253	-1.435992	-0.125229
H	3.589265	-2.681226	-1.498722
H	1.269178	-1.877087	-1.828195
H	4.538666	0.689614	0.999523
C	-1.896438	-0.256882	0.501855
C	-1.976709	-1.618218	0.755938
C	-2.375022	0.704455	1.381722
C	-2.566089	-2.028103	1.943485
H	-1.596183	-2.334186	0.038985
C	-2.959736	0.274193	2.562854
H	-2.290898	1.757281	1.144533
C	-3.052805	-1.085285	2.840699
H	-2.646734	-3.084285	2.164466
H	-3.342462	1.001844	3.266236
H	-3.511026	-1.412564	3.765390

IM

C	3.812795	-1.070321	0.111816
C	2.640625	-1.799786	-0.224998
C	1.450417	-1.206247	-0.527521
C	1.306715	0.222638	-0.513108
C	2.527132	0.906772	-0.172428
C	3.760875	0.286977	0.136517
N	2.440579	2.249953	-0.154593
N	0.250984	0.979557	-0.745094
N	2.312389	3.344359	-0.154771
S	-1.205179	0.321857	-1.148349
O	-1.072693	-0.767810	-2.131213
O	-2.076032	1.453076	-1.472674
H	4.732022	-1.590548	0.339735
H	2.695282	-2.881610	-0.248195
H	0.598560	-1.808824	-0.803569
H	4.619033	0.899478	0.380089
C	-1.803281	-0.410684	0.379902
C	-2.024111	-1.776476	0.446780
C	-2.044926	0.423793	1.464164
C	-2.495915	-2.322349	1.635527
H	-1.843029	-2.394003	-0.423879
C	-2.510640	-0.132172	2.645483
H	-1.875221	1.489980	1.376611
C	-2.734545	-1.503361	2.730382
H	-2.679351	-3.387198	1.701436
H	-2.705354	0.504198	3.499323
H	-3.102112	-1.932102	3.654298

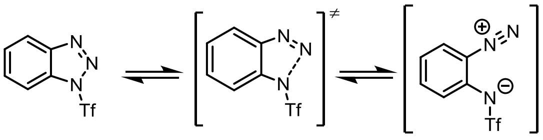

Sub **TS** **IM**

TS

C	3.783711	-1.128158	0.174224
C	2.687468	-1.902444	-0.244460
C	1.427859	-1.380036	-0.436489
C	1.193659	-0.005208	-0.215259
C	2.338665	0.733166	0.207542
C	3.613467	0.214624	0.404636
N	1.965466	2.054547	0.381350
N	0.079230	0.739381	-0.320556
N	0.980391	2.587045	0.251817
S	-1.366248	0.238795	-0.829147
O	-1.300220	-0.914698	-1.724333
O	-2.169998	1.401921	-1.165914
C	-2.112121	-0.434357	0.750669
F	-2.195768	0.516627	1.667051
F	-3.320812	-0.912890	0.507899
F	-1.343674	-1.413493	1.219237
H	4.752661	-1.586860	0.310780
H	2.834726	-2.960286	-0.424130
H	0.612724	-2.006054	-0.769047
H	4.418669	0.860810	0.726480

Sub

C	3.910804	-1.091599	0.177354
C	2.932143	-1.942947	-0.371003
C	1.641446	-1.522610	-0.625031
C	1.364076	-0.196652	-0.300036
C	2.323021	0.654475	0.247762
C	3.624445	0.218291	0.493663
N	1.745960	1.897272	0.468075
N	0.249739	0.620628	-0.388622
N	0.541354	1.880838	0.110207
S	-1.331285	0.247610	-0.836112
O	-1.255800	-0.968148	-1.617847
O	-1.987270	1.459067	-1.259278
C	-2.051291	-0.224795	0.832815
F	-1.960870	0.796032	1.657104
F	-3.312802	-0.558795	0.657218
F	-1.375075	-1.251178	1.313354
H	4.905764	-1.479969	0.349897
H	3.201290	-2.965083	-0.604516
H	0.897441	-2.177978	-1.053502
H	4.360674	0.889242	0.915367

IM

C	3.796478	-1.120146	0.161081
C	2.683409	-1.892065	-0.247553
C	1.435880	-1.364021	-0.448108
C	1.190319	0.025433	-0.250746
C	2.348207	0.755363	0.161278
C	3.634883	0.216228	0.368039
N	2.149767	2.082706	0.357077
N	0.068883	0.745042	-0.374925
N	1.946027	3.152189	0.503772
S	-1.330495	0.126812	-0.874877
O	-1.220280	-1.046189	-1.747129
O	-2.217630	1.220458	-1.246081
C	-2.055143	-0.552168	0.714532
F	-2.208435	0.409838	1.612585
F	-3.231756	-1.109999	0.475665
F	-1.237768	-1.475655	1.220264
H	4.760937	-1.585463	0.305387
H	2.821083	-2.954292	-0.408784
H	0.622852	-1.996082	-0.771189
H	4.444537	0.862230	0.680214

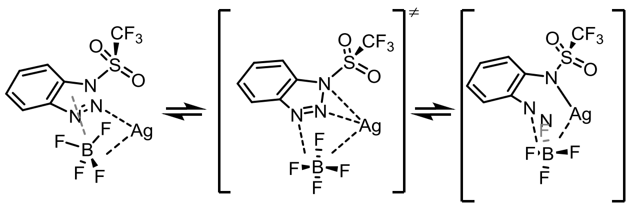

R = Tf with AgBF$_4$

Sub **TS** **IM**

TS

C	0.051068	4.400778	-0.226334
C	0.867519	3.853590	-1.223504
C	1.171964	2.504603	-1.285281
C	0.651703	1.659728	-0.302922
C	-0.154534	2.247445	0.698074

C	-0.479734	3.591508	0.756800
N	-0.556133	1.254608	1.590783
N	0.733099	0.306236	-0.136696
N	-0.254711	0.149141	1.496179
S	1.876451	-0.700377	-0.657744
O	2.523449	-0.274467	-1.887490
O	1.295742	-2.049454	-0.533745
C	3.192548	-0.633501	0.673280
F	2.660897	-0.960277	1.836752
F	4.164586	-1.470666	0.374412
F	3.666543	0.599672	0.736901
Ag	-1.109761	-1.896648	-0.345465
H	-0.169756	5.458972	-0.231504
H	1.270105	4.506868	-1.987306
H	1.785568	2.101915	-2.078351
H	-1.124841	3.963312	1.540788
B	-3.349860	0.116927	0.082753
F	-4.505636	0.152521	-0.655677
F	-3.332882	1.055676	1.095345
F	-3.166542	-1.191100	0.640564
F	-2.215420	0.311357	-0.759532

Sub

C	0.218455	4.622664	0.039666
C	0.905670	4.020974	-1.034520
C	1.118280	2.660012	-1.111173
C	0.607217	1.914872	-0.052487
C	-0.072026	2.497037	1.019374
C	-0.284509	3.875038	1.079377
N	-0.469906	1.501863	1.890241
N	0.572753	0.569440	0.254592
N	-0.084595	0.384556	1.455145
S	1.357839	-0.730376	-0.475021
O	1.411376	-0.487562	-1.894369
O	0.861799	-1.939490	0.157771
C	3.096135	-0.489054	0.196516
F	3.049527	-0.489350	1.512708
F	3.846616	-1.479525	-0.232911
F	3.568421	0.661924	-0.237131
Ag	-1.323396	-1.749281	1.545090
H	0.077382	5.695167	0.033164
H	1.272980	4.650061	-1.835054
H	1.627903	2.203068	-1.947414
H	-0.826525	4.314955	1.905285
B	-2.747152	-0.134710	-0.594878
F	-3.733615	-0.290896	-1.534189
F	-2.258770	1.161070	-0.543590
F	-3.231896	-0.501606	0.698407
F	-1.660516	-1.022228	-0.854227

IM

C	0.038128	4.382131	-0.277229
C	0.950658	3.827807	-1.189062
C	1.289454	2.491173	-1.192746
C	0.721852	1.609674	-0.253622
C	-0.158197	2.225401	0.670353
C	-0.534506	3.574780	0.668734
N	-0.667680	1.406124	1.643216
N	0.861610	0.254602	-0.138452
N	-1.030586	0.730878	2.424729
S	2.057626	-0.593292	-0.788292
O	2.764413	-0.009993	-1.924707
O	1.554097	-1.977804	-0.875380
C	3.325778	-0.646780	0.585252
F	2.799652	-1.190791	1.672008
F	4.375858	-1.352218	0.207883
F	3.708688	0.592784	0.867638
Ag	-0.875523	-1.507331	-0.323254
H	-0.217298	5.430923	-0.325551
H	1.400074	4.471736	-1.934809
H	1.972053	2.103586	-1.933476
H	-1.251146	3.934120	1.394828
B	-3.328755	0.292735	0.127999
F	-4.521002	0.110300	-0.520994
F	-3.377731	1.336004	1.045623
F	-2.944098	-0.907378	0.806593
F	-2.280027	0.544684	-0.796638
